# Mendelian randomization analysis does not reveal a causal influence of mental diseases on osteoporosis

**DOI:** 10.3389/fendo.2023.1125427

**Published:** 2023-04-20

**Authors:** Fen Tang, Sheng Wang, Hongxia Zhao, Demeng Xia, Xin Dong

**Affiliations:** ^1^ School of Medicine, Shanghai University, Shanghai, China; ^2^ Department of Emergency, Changhai Hospital, Naval Military Medical University, Shanghai, China; ^3^ Clinical Research Institute of Zhanjiang, Central People’s Hospital of Zhanjiang, Zhanjiang, China; ^4^ Luodian Clinical Drug Research Center, Shanghai Baoshan Luodian Hospital, Shanghai University, Shanghai, China; ^5^ Institute of Translational Medicine, Shanghai University, Shanghai, China

**Keywords:** mental diseases, osteoporosis, osteoporosis fracture, Mendelian randomization, genome-wide association study

## Abstract

**Introduction:**

Osteoporosis (OP) is primarily diagnosed through bone mineral density (BMD) measurements, and it often leads to fracture. Observational studies suggest that several mental diseases (MDs) may be linked to OP, but the causal direction of these associations remain unclear. This study aims to explore the potential causal association between five MDs (Schizophrenia, Depression, Alzheimer's disease, Parkinson's disease, and Epilepsy) and the risk of OP.

**Methods:**

First, single-nucleotide polymorphisms (SNPs) were filtered from summary-level genome-wide association studies using quality control measures. Subsequently, we employed two-sample Mendelian randomization (MR) analysis to indirectly analyze the causal effect of MDs on the risk of OP through bone mineral density (in total body, femoral neck, lumbar spine, forearm, and heel) and fractures (in leg, arm, heel, spine, and osteoporotic fractures). Lastly, the causal effect of the MDs on the risk of OP was evaluated directly through OP. MR analysis was performed using several methods, including inverse variance weighting (IVW)-random effects, IVW-fixed effects, maximum likelihood, weighted median, MR-Egger regression, and penalized weighted median.

**Results:**

The results did not show any evidence of a causal relationship between MDs and the risk of OP (with almost all P values > 0.05). The robustness of the above results was proved to be good.

**Discussion:**

In conclusion, this study did not find evidence supporting the claim that MDs have a definitive impact on the risk of OP, which contradicts many existing observational reports. Further studies are needed to determine the potential mechanisms of the associations observed in observational studies.

## 1 Introduction

Osteoporosis (OP) is the most common systemic bone disease, characterized by a decrease in bone mineral density (BMD) and brittle fractures caused by deterioration of bone microstructure (1), which can easily lead to disability or even death in elderly patients (2). The standard diagnostic method for OP involves measuring BMD through dual-energy x-ray absorptiometry at the same skeletal site from childhood to old age. The femoral neck, lumbar spine, and forearm are the most commonly used skeletal sites for diagnosing OP (3, 4). Recently, the heel site has also been used to estimate OP (5). Moreover, total body BMD (TB-BMD) measurement is also an appropriate method for an unbiased assessment of BMD. Fractures are another feature of OP (6–8), with the leg, arm, heel, and spine being the most representative. According to the latest report of the International Osteoporosis Foundation, one in three women and one in five men over the age of 50 will experience OP worldwide (6–9). This disease not only impacts the patient’s quality of life but also poses a significant burden on public health and the national economy.

Mental diseases (MDs) are becoming increasingly prevalent in modern populations and can be classified into primary and secondary psychosis. Primary psychosis includes schizophrenia (SCH), depression (MDD), mood disorders, split personality, and other related conditions. Secondary psychosis, on the other hand, is caused by somatic organ diseases, neurological diseases, or substance abuse, and includes conditions such as Alzheimer’s disease (AD), stroke, Parkinson’s disease (PD), and epilepsy (EP) (10). As chronic diseases, MDs have been linked to abnormal bone metabolism, with patients suffering from some of the most common psychiatric disorders such as SCH (9), MDD (11), AD (12), PD (13), or EP (14) being more likely to have lower BMD and fracture risk, including OP, compared to the general population. However, most of these reports are observational in nature and may be subject to confounding factors, making it difficult to establish a definitive etiological link between MDs and OP.

Mendelian randomization (MR) analysis, the mimic design of randomized control trials, is an epidemiological research method that uses genetic variants (typically single-nucleotide polymorphisms, SNPs) to assess the causal association between modifiable exposures (or risk factors) and outcome (15, 16). MR analysis has advantages over clinical trials in terms of financial resources, material resources, and time. It is extensively applied in various studies (17, 18), particularly in research related to COVID-19 (19–21).

The aim of this study is to explore the potential causal relationship between MDs and the risk of OP by leveraging genetic variation through the use of two-sample MR analysis (22). Our investigation seeks to contribute novel insights and empirical evidence to the field of research on the association between MDs and OP.

## 2 Method

### 2.1 Outcome genome-wide association studies summary statistics

In this study, we estimated the causal effect of MDs on BMD using the five genome-wide association studies (GWAS) summary data (TB-BMD; femoral neck-BMD, FN-BMD; lumbar spine-BMD, LS-BMD; forearm-BMD, FA-BMD; and heel-BMD, eBMD), as well as the causal effect of MDs on fracture using an additional five GWAS summary data (leg fracture, LF; arm fracture, AF; heel fracture, HF; spine fracture, SF; and osteoporotic fractures, OPF). These data indirectly evaluated the causal impact of MDs on the risk of OP *via* MR analysis. At the end of those parts, we directly assessed the causal effect of MDs on the risk of OP by utilizing GWAS summary data of OP.

GWAS summary statistics for BMDs were downloaded from the GEnetic Factors for osteoporosis Consortium website (GEFOS, http://www.gefos.org/). GWAS summary statistics for fracture were downloaded from the Medical Research Council Integrative Epidemiology Unit website (MRC-IEU, http://www.bristol.ac.uk/integrative-epidemiology/). GWAS summary statistics for OPF and OP were downloaded from the FinnGen website (https://www.finngen.fi/en/access_results). In addition, GWAS summary statistics for MDs, BMD, fractures, and OP were downloaded from the GWAS catalog website (https://www.ebi.ac.uk/gwas/downloads/summary-statistics). All study participants were of European descent. More detailed information can be found in **Table 1**.

**Table 1:** The detailed data information of the Mendelian randomization study on the association of MDs with the risk of OP.

Type	Study/Consortium	Population	Sample size	Sample control	Datasets in the GWAS
Exposure					
Schizophrenia	PGC	European	82,315	46,839	ieu-a-22
Depression	UK Biobank	European	322,580	208,811	ebi-a-GCST005902
Alzheimer’s Disease	IGAP	European	74,046	48,466	ieu-a-298
Parkinson	LNG	European	5,691	3,978	ieu-b-7
Epilepsy	ILNE	European	33,446	29,677	ieu-b-9
Outcome					
FA-BMD	GEFOS	European	8,143	NA	ieu-a-977
LS-BMD	GEFOS	European	28,489	NA	ieu-a-982
FN-BMD	GEFOS	European	32,735	NA	ieu-a-980
eBMD	MRC-IEU	European	265,627	NA	ukb-b-8875
TB-BMD	GEFOS	European	56,284	NA	ebi-a-GCST005348
AF	MRC-IEU	European	460,340	455,626	ukb-b-19255
SF	MRC-IEU	European	460,340	459,304	ukb-b-873
LF	MRC-IEU	European	460,340	457,352	ukb-b-3798
HF	MRC-IEU	European	306,379	NA	ukb-b-18389
OPF	The FinnGen consortium	European	173,619	NA	finn-b-OSTEOPORO SIS_FRACTURE_FG
OP	The FinnGen consortium	European	212,778	209,575	finn-b-M13_OSTEOP OROSIS

TB-BMD, total body bone mineral density; FN-BMD, femur neck bone mineral density; LS-BMD, lumbar spine bone mineral density; FA-BMD, forearm bone mineral density; eBMD, heel bone mineral density; LF, leg fractures; AF, arm fractures; HF, heel fractures; SF, spine fractures; OPF, osteoporosis fracture; OP, osteoporosis; PGC, the Psychiatric Genomics Consortium; UK Biobank, the UK Biobank Psychiatric Genetics Group; IGAP, the French National Foundation on Alzheimer’s Disease and Related Disorder; LNG, US National Institute of Neurological Disorders and Stroke (NINDS) Human Genetics Resource Center DNA and Cell Line Repository; ILNE, The International League Against Epilepsy Consortium on Complex Epilepsies; GEFOS, the Genetic Factors for osteoporosis Consortium; MRC-IEU, the Medical Research Council Integrative Epidemiology Unit.

To derive a reliable and valid inference regarding the correlation between MDs and OP, we opted for the most substantial GWAS database of MDs, encompassing SCH (*N* = 35,476 cases; 46,839 controls) (23) from the Psychiatric Genomics Consortium, MDD (*N* = 113,769 cases; 208,811 controls) (24) from the UK Biobank Psychiatric Genetics Group, AD (*N* = 25,580 cases; 48,466 controls) (25) from the French National Foundation on Alzheimer’s Disease and Related Disorder, PD (*N* = 1,713 cases; 3,978 controls) (26) from US National Institute of Neurological Disorders and Stroke (NINDS), and EP (*N* = 3,769 cases; 29,677 controls) (27) from The International League Against Epilepsy Consortium on Complex Epilepsies.

The GWAS database of TB-BMD (*N* = 66,628), FN-BMD (*N* = 32,735), LS-BMD (*N* = 28,498), and FA-BMD (*N* = 8,143) were downloaded from GEFOS (28, 29). Five separate GWAS summary statistics of eBMD (*N* = 265,627), AF (*N* = 4,714 cases; 455,626 controls), SF (*N* = 1,036 cases; 459,304 controls), LF (*N* = 2,988 cases; 457,352 controls), and HF (*N* = 306,379) were downloaded from MRC-IEU. The data of separate GWAS summary statistics of OPF (*N* = 173,619) and OP (*N* = 3,203 cases; 209,575 controls) were downloaded from the FinnGen consortium.

### 2.2 Selection of genetic instrumental variants

We employed stringent criteria to select SNPs as the genetic instrumental variables from the GWAS summary data of MDs, including SCH, MDD, AD, PD, and EP. Initially, SNPs with genome-wide significance (*p* < 5 × 10^−8^, *R* ^2^ < 0.001, kb = 10,000) of MDs were selected. Subsequently, the clumping process (*R* ^2^ > 0.001, window size = 10,000 kb) was executed to ensure that all the SNPs were not in linkage disequilibrium (LD) with the clump data function. Thirdly, if an SNP was not present in the outcome GWAS during the R calculation process, it would also be excluded. Fourthly, any ambiguous or palindromic SNPs that were ambiguous with non-concordant alleles (e.g., A/G vs. A/C) or with an ambiguous strand (i.e., A/T or G/C) were excluded. Finally, using the PhenoScanner tool (http://www.phenoscanner.medschl.cam.ac.uk/) (30–32), we excluded any SNPs associated with the confounding factor of the outcome, and we used the *F*-statistic to indicate the strength of the genetic instrumental variants.

### 2.3 Two-sample MR analysis

**Figure 1:**
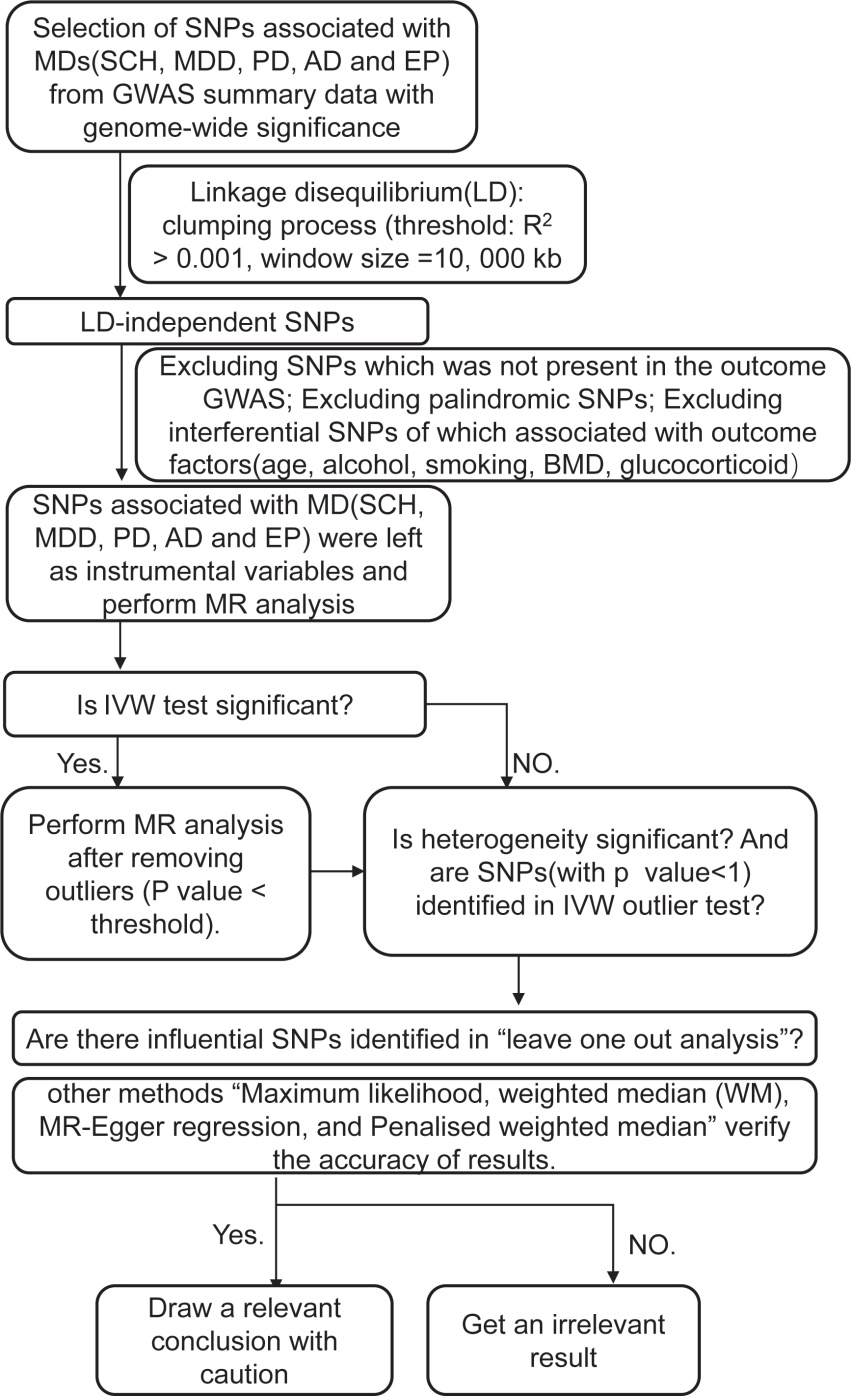
A step-by-step illustration of the study design and workflow. SNP, single-nucleotide polymorphism; MDs, mental diseases; SCH, schizophrenia; MDD, depression; AD, Alzheimer’s disease; PD, Parkinson’s disease; EP, epilepsy; GWAS, genome-wide association studies; BMD, bone mineral density; MR analysis, Mendelian randomization analysis; IVX, inverse variance weighting.

The instrumental SNPs were utilized to carry out a two-sample MR analysis for the purpose of evaluating the causal effect of MDs on the risk of OP. The summary statistics (OR and standard error) of BMD and fracture enabled the indirect assessment of the causal association between MDs and the risk of OP, whereas the summary statistics of OP facilitated a direct evaluation. Detailed methods of MR analysis included inverse variance weighting (IVW)-random effects, IVW-fixed meta-analysis, maximum likelihood, weighted median (WM), and MR-Egger regression, and penalized weighted median was applied to estimate the effects. Bonferroni correction (*p*-value = 0.05/11 outcomes) was used to adjust for multiple testing (*p* = 0.0045) in this MR. All of these analyses were conducted in R V.4.2.0 by using R packages of “Two-Sample MR” (https://mrcieu.github.io/TwoSampleMR/reference/clump_data.html) (33) and *p*-values < 0.05 were considered statistically significant. The detailed steps are shown in the flowchart (**Figure 1**).

### 2.4 Robust analysis

IVW (random effect and fixed effect) and MR Egger regression were used to assess the potential horizontal pleiotropic effects of the SNPs. Cochran *Q*-test statistics were used to quantify heterogeneities. Furthermore, we performed a “leave-one-out” sensitivity analysis to identify potentially influential SNPs. In this method, we excluded each SNP in turn and checked whether it was responsible for the association. We also applied the MR Steiger filtering method to verify the causality between MDs and OP.

## 3 Results

### 3.1 Selection of SNPs: Instrumental variables

We obtained 83 SNPs in SHC, 14 SNPs in MDD, 20 SNPs in AD, 23 SNPs in PD, and 11 SNPs in EP, which met the generally accepted genome-wide significance threshold (*p* < 5 × 10^−8^, *r* ^2^ < 0.001, kb = 10,000) for exposure. Some SNPs strongly associated with confounding factors such as alcohol (34), glucocorticoid use (35), BMD (3, 4), and age (36) were eliminated. The removed SNPs are as follows: seven SNPs (rs11210892, rs2909457, rs2851447, rs13217619, rs7405404, rs4129585, and rs12887734) in SHC, one SNP (rs9530139) in MDD, two SNPs (rs10838725 and rs983392) in AD, two SNPs (rs35265698 and rs58879558) in PD, and one SNP (rs1402398) in EP. Moreover, all *F*-statistics > 10 indicated no weak instrument bias, the Bonferroni test verified the multiple testing *p*-value precision (*p*-value > 0.0045), and the directionality test conducted by MR Steiger confirmed our estimation of potential causal direction (*p* < 0.001). The detailed information is listed in **Supplementary Material 1**.

### 3.2 Two-sample MR analysis for association between MDs and the risk of OP

**Figure 2:**
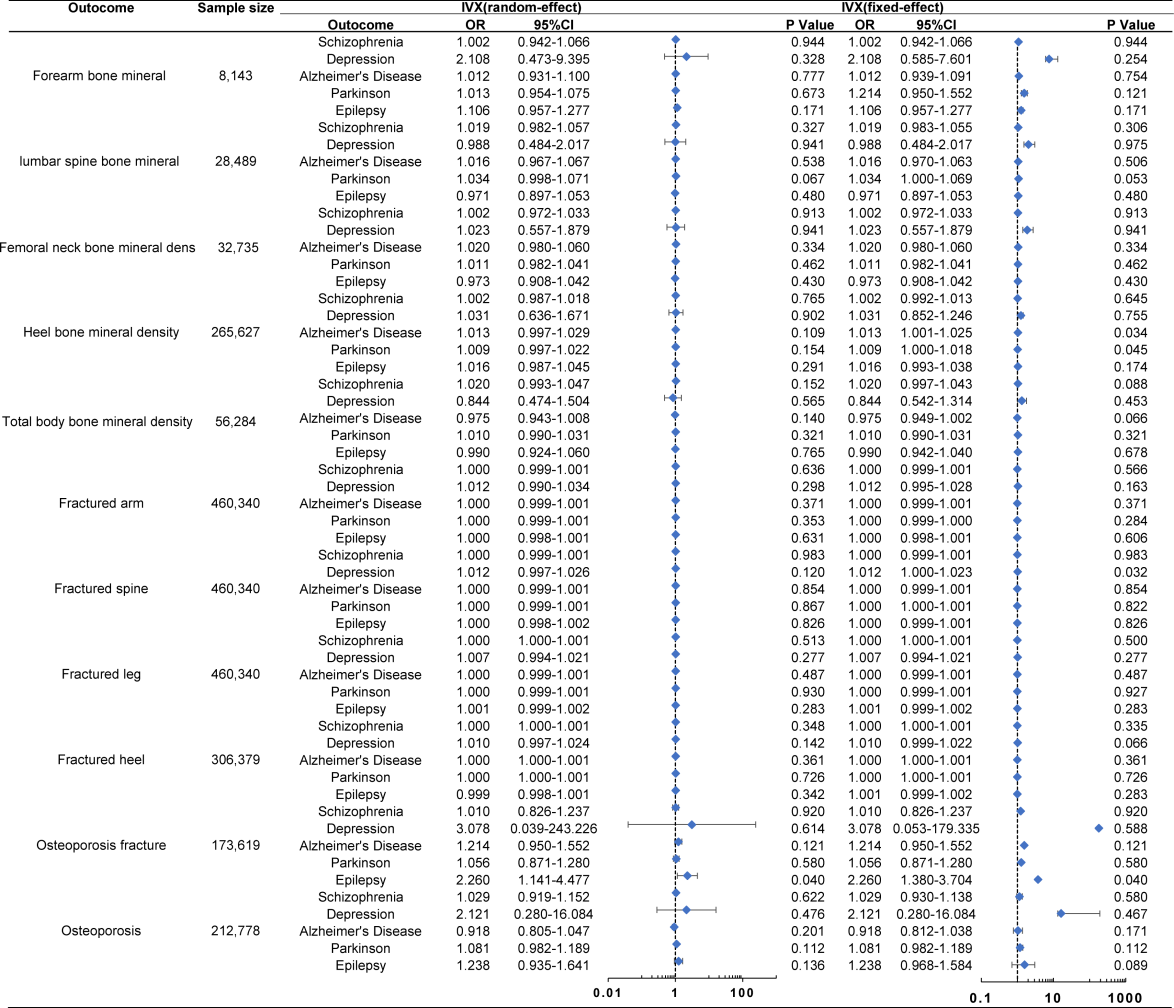
Associations of genetically predicted MDs with the risk of OP. The Forest plot of causal relationship between MDs and the risk of OP using the IVX. All *p*-values > 0.05 in the IVW random method. OR, odds ratio; IVX, inverse variance weighting; CI, confidence interval.

The MR analysis did not reveal any significant causal association between MDs and the risk of OP, whether analyzed indirectly from the causal effect of MDs on BMD (TB-BMD, FN-BMD, LS-BMD, FA-BMD, and eBMD) and fractures (LF, AF, SF, HF, and OPF), or directly from the causal effect of the MDs on OP (all *p* > 0.05) (**Figure 2**). The other methods including IVW-fixed effects, maximum likelihood, WM, MR-Egger regression, and penalized weighted median also verified that there was no significant association between MDs and the risk of OP (all *p* > 0.05). The results of those verified methods were consistent with the result in the MR analysis (**Supplementary Material 2**). The effect size of each SNP on the BMD, fracture, and OP is shown in **Supplementary Material 3**-**7**.

#### 3.2.1 Causal effect of SCH with the risk of OP

The MR analysis presented in **Supplementary Material 2** estimated the causal effect of SCH on the risk of OP through indirect means such as BMD and fractures, as well as direct means such as OP. In the primary IVW analyses, SCH showed no MR association with BMD [FA-BMD: OR (95% CI) 1.002 (0.942–1.066), *p* = 0.944; LS-BMD: OR (95% CI) 1.019 (0.982–1.057), *p* = 0.327; FN-BMD: OR (95% CI) 1.002 (0.972–1.033), *p* = 0.913; eBMD: OR (95% CI) 1.002 (0.987–1.018), *p* = 0.765; and TB-BMD: OR (95% CI) 1.020 (0.993–1.047), *p* = 0.152], fracture [AF: OR (95% CI) 1.000 (0.999–1.001), *p* = 0.636; SF: OR (95% CI) 1.000 (0.999–1.001), *p* = 0.983; LF: OR (95% CI) 1.000 (1.000–1.001), *p* = 0.513; HF: OR (95% CI) 1.000 (1.000–1.001), *p* = 0.348; and OPF: OR (95% CI) 1.010 (0.826–1.237), *p* = 0.920], and OP (OP: OR (95% CI) 1.029 (0.919–1.152), *p* = 0.622] (**Figure 2**). These results were corroborated by other methods, indicating that SCH had no MR association with the risk of OP through either indirect (BMD and fractures) or direct (OP) pathways, with all *p*-values greater than 0.05 (**Supplemental Materials 2**, **3**).

#### 3.2.2 Causal effect of MDD with the risk of OP

The MR analysis conducted to estimate the causal effect of MDD on the risk of OP is outlined in **Supplementary Material 2**. In the primary IVW analyses, MDD showed no MR association with BMD [FA-BMD: OR (95% CI) 2.108 (0.473–9.395), *p* = 0.328; LS-BMD: OR (95% CI) 0.988 (0.484–2.017), *p* = 0.975; FN-BMD: OR (95% CI) 1.023 (0.557–1.879), *p* = 0.941; eBMD: OR (95% CI) 1.031 (0.636–1.671), *p* = 0.902; and TB-BMD: OR (95% CI) 0.844 (0.474–1.504), *p* = 0.565], fracture [AF: OR (95% CI) 1.012 (0.990–1.034), *p* = 0.298; SF: OR (95% CI) 1.012 (0.997–1.026), *p* = 0.120; LF: OR (95% CI) 1.007 (0.994–1.021), *p* = 0.277; HF: OR (95% CI) 1.010 (0.997–1.024), *p* = 0.142; and OPF: OR (95% CI) 3.078 (0.039–243.226), *p* = 0.614], and OP [OP: OR (95% CI) 2.121 (0.280–16.084), *p* = 0.476] (**Figure 2**). Additional methods also confirmed that MDD was not associated with the risk of OP using both indirect (BMD and fractures) and direct (OP) approaches (all *p* > 0.05) (**Supplementary Material 2**, **4**).

#### 3.2.3 Causal effect of AD with the risk of OP

**Supplementary Material 2**, which includes MR estimates obtained from various methods of assessing the causal effect of AD on the risk of OP, indicated that AD demonstrated no association with the risk of OP. In the primary IVW analyses, AD exhibited no MR association with BMD [FA-BMD: OR (95% CI) 1.012 (0.931–1.100), *p* = 0.777; LS-BMD: OR (95% CI) 1.016 (0.967–1.067), *p* = 0.538; FN-BMD: OR (95% CI) 1.020 (0.980–1.060), *p* = 0.334; eBMD: OR (95% CI) 1.013 (0.997–1.029), *p* = 0.109, and TB-BMD: OR (95% CI) 0.975 (0.943–1.008), *p* = 0.140], fracture [AF: OR (95% CI) 1.000 (0.999–1.001), *p* = 0.371; SF: OR (95% CI) 1.000 (0.999–1.001), *p* = 0.854; LF: OR (95% CI) 1.000 (0.999–1.001), *p* = 0.487; HF: OR (95% CI) 1.000 (1.000–1.001), *p* = 0.361; and OPF: OR (95% CI) 1.214 (0.950–1.552), *p* = 0.121], and OP [OP: OR (95% CI) 0.918 (0.805–1.047), *p* = 0.201] (**Figure 2**). The results from other methods also supported the null association between AD and the risk of OP (all *p* > 0.05) (**Supplementary Material 2**, **5**).

#### 3.2.4 Causal effect of PD with the risk of OP

In the primary IVW analyses, PD was found to have no association with the risk of OP. In terms of its indirect aspect, PD was also found to have no MR association with BMD [FA-BMD: OR (95% CI) 1.013 (0.954–1.075), *p* = 0.673; LS-BMD: OR (95% CI) 1.034 (0.998–1.071), *p* = 0.067; FN-BMD: OR (95% CI) 1.011 (0.982–1.041), *p* = 0.462; eBMD: OR (95% CI) 1.009 (0.997–1.022), *p* = 0.154; and TB-BMD: OR (95% CI) 1.010 (0.990–1.031), *p* = 0.321] and fracture [AF: OR (95% CI) 1.000 (0.999–1.000), *p* = 0.353; SF: OR (95% CI) 1.000 (0.999–1.000), *p* = 0.867; LF: OR (95% CI) 1.000 (0.999–1.000), *p* = 0.930; HF: OR (95% CI) 1.000 (0.999–1.000), *p* = 0.726; and OPF: OR (95% CI) 1.056 (0.871–1.280), *p* = 0.580]. In the direct aspect, PD showed no MR association with OP [OP: OR (95% CI) 1.081 (0.982–1.189), *p* = 0.112] (**Figure 2**). Additional methods also demonstrated that PD had no MR association with the risk of OP (all *p* > 0.05) (**Supplementary Material 2**, **6**).

#### 3.2.5 Causal effect of EP with the risk of OP

We estimated the EP’s causal effect on the risk of OP. In the primary IVW analyses, EP showed no MR association with BMD [FA-BMD: OR (95% CI) 1.106 (0.957–1.277), *p* = 0.171; LS-BMD: OR (95% CI) 0.971 (0.897–1.053), *p* = 0.480; FN-BMD: OR (95% CI) 0.973 (0.908–1.042), *p* = 0.430; eBMD: OR (95% CI) 1.016 (0.987–1.045), *p* = 0.291; and TB-BMD: OR (95% CI) 0.990 (0.924–1.060), *p* = 0.765], fracture [AF: OR (95% CI) 1.000 (0.998–1.001), *p* = 0.631; SF: OR (95% CI) 1.000 (0.998–1.002), *p* = 0.868; LF: OR (95% CI) 1.001 (0.999–1.002), *p* = 0.283; HF: OR (95% CI) 0.999 (0.998–1.001), *p* = 0.342; and OPF: OR (95% CI) 2.260 (1.141–4.477), *p* = 0.137], and OP [OP: OR (95% CI) 1.238 (0.935–1.641), *p* = 0.136] (**Figure 2**). Other methods also indicated that EP had no MR association with the risk of OP (all *p* > 0.05) (**Supplementary Material 2**, **7**).

### 3.3 Robustness

Cochran’s *Q*-test did not reveal any sign of heterogeneity during the sensitivity analyses (all *p* > 0.05) (**Supplementary Material 8**). The MR-Egger regression method examined the possibility of horizontal pleiotropy between SNPs and outcome, and the results indicated no evidence of such pleiotropy (all *p* > 0.05) (**Supplementary Material 8**). Additionally, the funnel plots suggested no observable horizontal pleiotropy for any of the outcomes (**Supplementary Material 3**-**7**). Furthermore, the leave-one-out sensitivity analysis plots demonstrated that no single SNP was likely to have influenced the causal association and that our conclusions were therefore robust (**Supplementary Material 3**-**7**). Taken together, all of the findings suggest that the null association between genetic predisposition to MDs and the risk of OP was not significantly impacted by any individual SNP (**Supplementary Material 3**-**7**).

## 4 Discussion

To our knowledge, this is the first study to evaluate the causal association between five kinds of MDs and the risk of OP using two-sample MR analysis. This study extensively mined the largest database, GWAS, and other relevant databases to investigate the causal association between the five most prevalent MDs (SCH, MDD, AD, PD, and EP) and the risk of OP, as well as its clinical manifestations (BMD and fracture). Our findings suggest that there is no clear causal relationship between mental disorders and the risk of OP.

Existing reports are limited to observational studies. These studies have consistently found that MDs increase the risk of OP (11, 13, 37–39). Specifically, Gomez and Stubbs discovered that individuals with SCH and MDD tend to have lower BMD at the hip and lumbar spine compared to healthy individuals (37, 40). Liu et al. studied PD and observed that PD patients have lower BMD at the total body, arm, and femur neck (13). Zhao et al. investigated the link between MDs and OP, and found that individuals with AD have a greater propensity for hip fractures (38). It is important to note, however, that these were all observational studies.

The aforementioned findings are in contrast to our own results, which may be attributed to the limitations of observational studies. Firstly, the studies cited were all based on case–control and cross-sectional designs, leaving it uncertain whether MDs are prospectively linked to an increased risk of OP. Secondly, many patients with MDs take long-term medications such as corticosteroids, which could potentially introduce bias in the results. Thirdly, some of the original studies lacked access to raw data, which could affect the accuracy of the findings. Lastly, certain assessment criteria may lower the reliability of the results, and BMD and fracture risk of multiple body parts should be taken into account in the investigation.

Due to the limitations of conventional observational study (41), we used “two-sample MR analysis” (16, 42) to ascertain the causal effects between MDs and the risk of OP, proceeding from the indirect aspect (BMD and fracture) to the direct aspect (OP). As we all know, MDs are frequently accompanied by other illnesses and substance abuse, resulting in the traditional misconception that psychiatric disorders can result in OP. However, which returns to the genetic level of analysis, indicates that MDs cannot lead to OP directly. Meanwhile, the randomness and fixedness of alleles preclude the reverse causation bias (43). The use of heterogeneity and sensitivity analysis in our analysis, with various Mendelian tools, augments the result’s stability, and the extensive sample size and singular population distribution diminish the bias of population stratification, ensuring an accurate causal effect between MDs and the risk of OP.

Undoubtedly, this study is not exempt from some constraints. First and foremost, the study’s participants are exclusively of European descent, necessitating further data collection and analysis to determine whether the findings apply to other populations. Second, the absence of publicly available source data precludes us from determining the potential sample overlap bias. Finally, even though we took measures to eliminate any confounding factors, we cannot fully rule out the potential influence of horizontal pleiotropy on our findings.

Moreover, given the constraints of our current research, our findings should be viewed as preliminary and warrant further investigation. Additionally, given the intricate nature of confounding factors, it is still advisable to consider various intervention methods and prevention strategies (44) for patients with MDs. These methods could include modifications to their lifestyle, such as promoting physical activity and improving their diet and exercise regime, increasing their intake of calcium and vitamin D (45), and minimizing the risk of falls.

## 5 Conclusion

The results of this study did not provide conclusive evidence to support the notion that MDs (including SCH, MDD, AD, PD, and EP) have a direct causal effect on the risk of OP. This is in contrast to the findings of numerous observational studies. It is possible that the relationship between MDs and OP risk reported in these observational studies is confounded by other risk factors. With the availability of more sophisticated approaches and a larger sample size of OP patients, the estimates can become less biased and the results can be more accurate. It is important to acknowledge that further research is needed to fully understand the relationship between MDs and OP risk.

## Data availability statement

The original contributions presented in the study are included in the article/**Supplementary Material**. Further inquiries can be directed to the corresponding authors. The R code to perform the MR analysis is detailed in the presentation 1 (**Supplementary Material 9**).

## Ethics statement

This study used publicly available GWAS summary databases. Thus, no ethical committee approval was required.

## Author contributions

XD, FT, DX, SW, and HZ conceived the idea for the study. FT obtained the genetic data and performed the data analyses. FT, DX, SW, and HZ interpreted the results of the data analyses. All authors contributed to the article and approved the submitted version.

## Supplementary material

Supplementary Material 1 Instrumental variables SNPs.

Supplementary Material 2 Other methods to assess the link of MDs with the risk of OP.

Supplementary Material 8 Heterogeneity, level pleiotropy test and the power of Mendelian randomization analyses.

Supplementary Material 9 The detailed R code perform the MR analysis.

## Glossary

**Table:**

MDs	mental diseases
OP	osteoporosis
MR	Mendelian randomization
WM	weighted median
BMD	bone mineral density
GWAS	genome-wide association studies
SCH	schizophrenia
MDD	depression
AD	Alzheimer’s disease
PD	Parkinson’s disease
EP	epilepsy
OPF	osteoporosis fracture
SNP	single-nucleotide polymorphism
OR	odds ratio
CI	confidence interval
TB-BMD	total body bone mineral density
FN-BMD	femur neck bone mineral density
LS-BMD	lumbar spine bone mineral density
FA-BMD	forearm bone mineral density
eBMD	heel bone mineral density
LF	leg fractures
AF	arm fractures
HF	heel fractures
SF	spine fractures
PGC	the Psychiatric Genomics Consortium
UK Biobank	the UK Biobank Psychiatric Genetics Group
IGAP	the French National Foundation on Alzheimer’s Disease and Related Disorder
LNG	US National Institute of Neurological Disorders and Stroke (NINDS) Human Genetics Resource Center DNA and Cell Line Repository
ILNE	The International League Against Epilepsy Consortium on Complex Epilepsies
GEFOS	the Genetic Factors for osteoporosis Consortium
MRC-IEU	the Medical Research Council Integrative Epidemiology Unit.
