# Supplementary material for: Mendelian randomization analysis does not reveal a causal influence of mental diseases on osteoporosis
Source: Front Endocrinol (Lausanne). 2023 Apr 20;14:1125427. doi: 10.3389/fendo.2023.1125427 (PMC10157183; doi:10.3389/fendo.2023.1125427)
Supplement: Supplementary Material 1 — Instrumental variables SNPs. [file DataSheet_1.zip › Supplementary Material/Supplementary Material 3.pdf]

Figure S1 Leave-one-out analysis, MR effect size and funnel plot for SHC on OP.

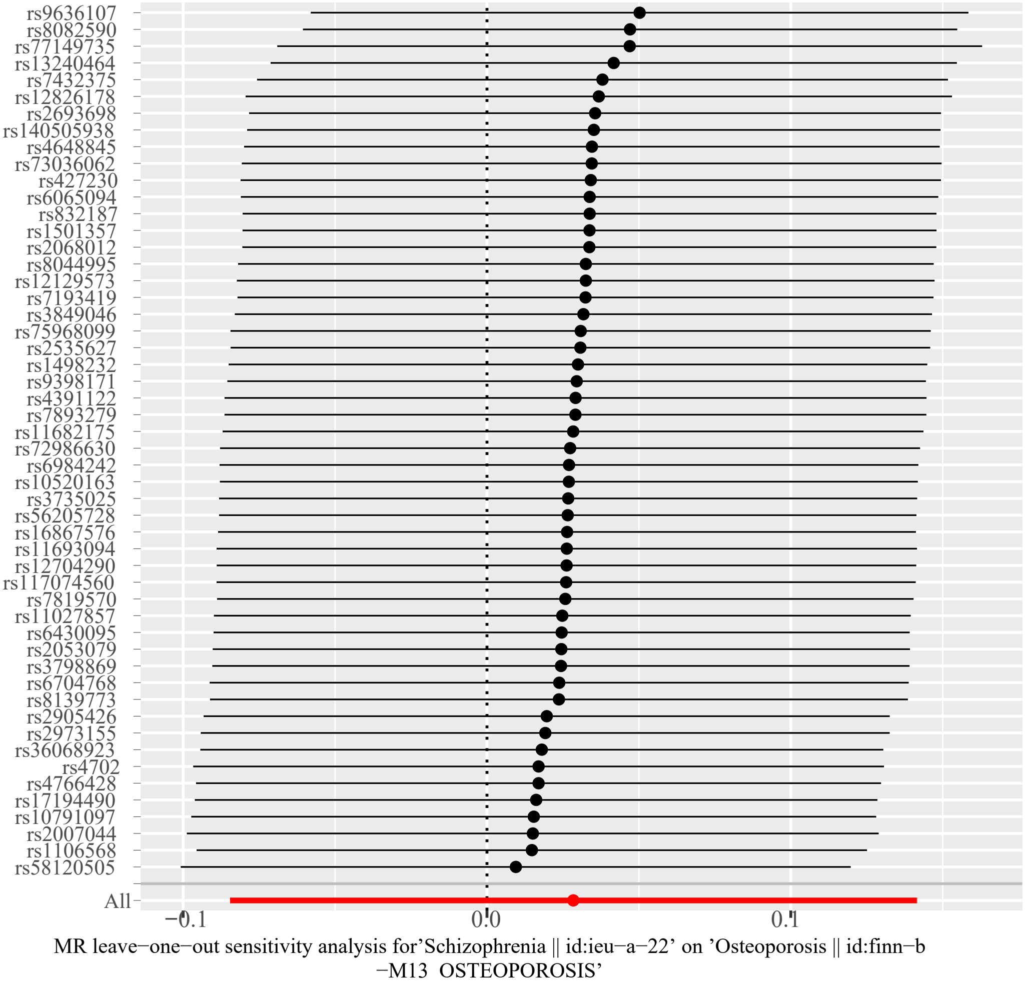

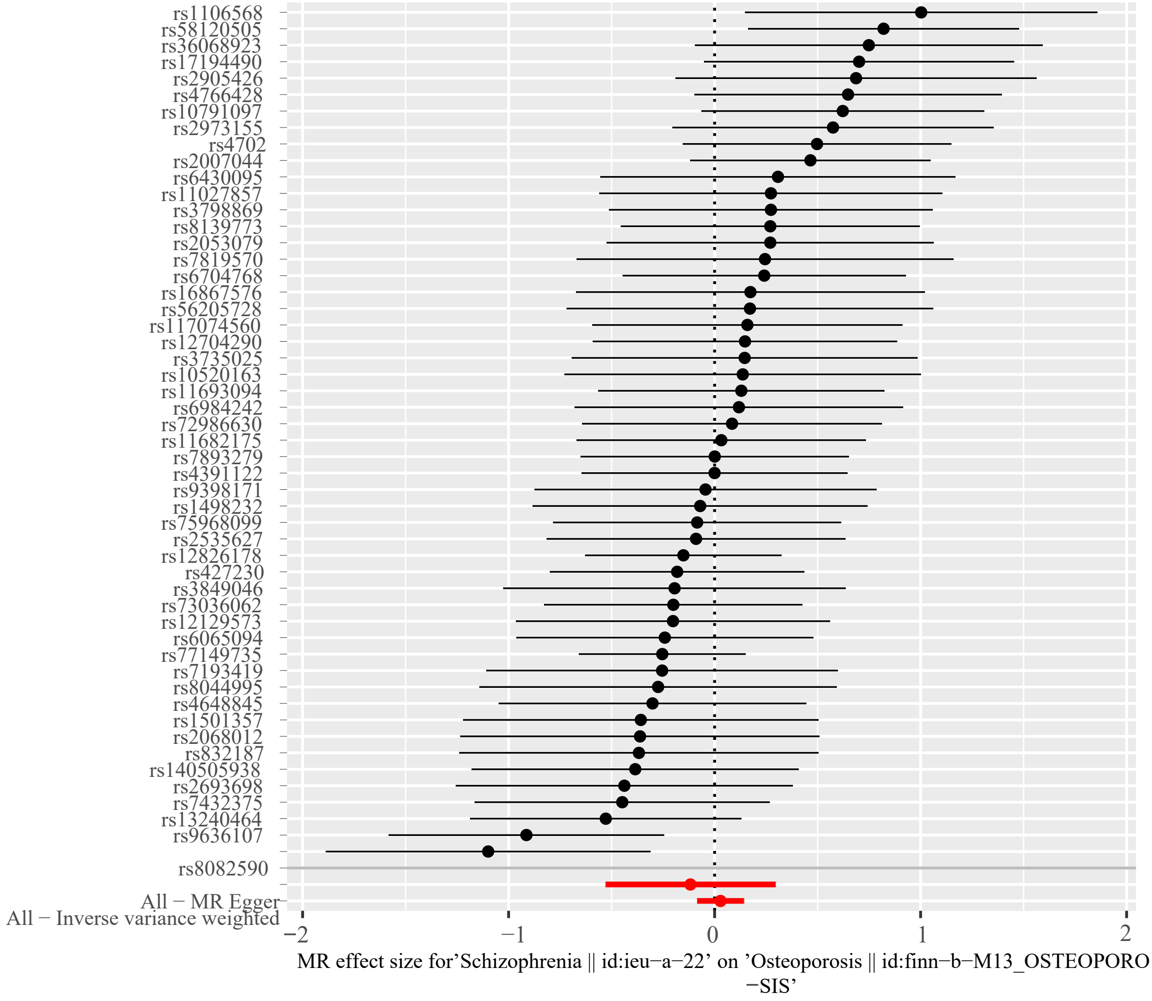
rs1106568
rs58120505
rs36068923
rs17194490
rs2905426
rs4766428
rs10791097
rs2973155
rs4702
rs2007044
rs6430095
rs11027857
rs3798869
rs8139773
rs2053079
rs7819570
rs6704768
rs16867576
rs56205728
rs117074560
rs12704290
rs3735025
rs10520163
rs11693094
rs6984242
rs72986630
rs11682175
rs7893279
rs4391122
rs9398171
rs1498232
rs75968099
rs2535627
rs12826178
rs427230
rs3849046
rs73036062
rs12129573
rs6065094
rs77149735
rs7193419
rs8044995
rs4648845
rs1501357
rs2068012
rs832187
rs140505938
rs2693698
rs7432375
rs13240464
rs9636107
rs8082590
All − MR Egger
All − Inverse variance weighted
−2
−1
0
1
2
MR effect size for'Schizophrenia || id:ieu−a−22' on 'Osteoporosis || id:finn−b−M13_OSTEOPORO
−SIS'

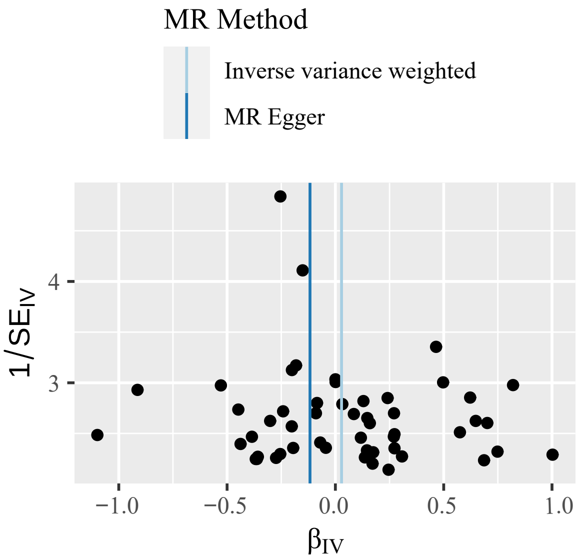

MR Method
Inverse variance weighted
MR Egger
4
3
$1/SE_{IV}$
−1.0
−0.5
0.0
0.5
1.0
β_{IV}

Figure S2. Leave-one-out analysis, MR effect size and funnel plot for SHC on OPF.

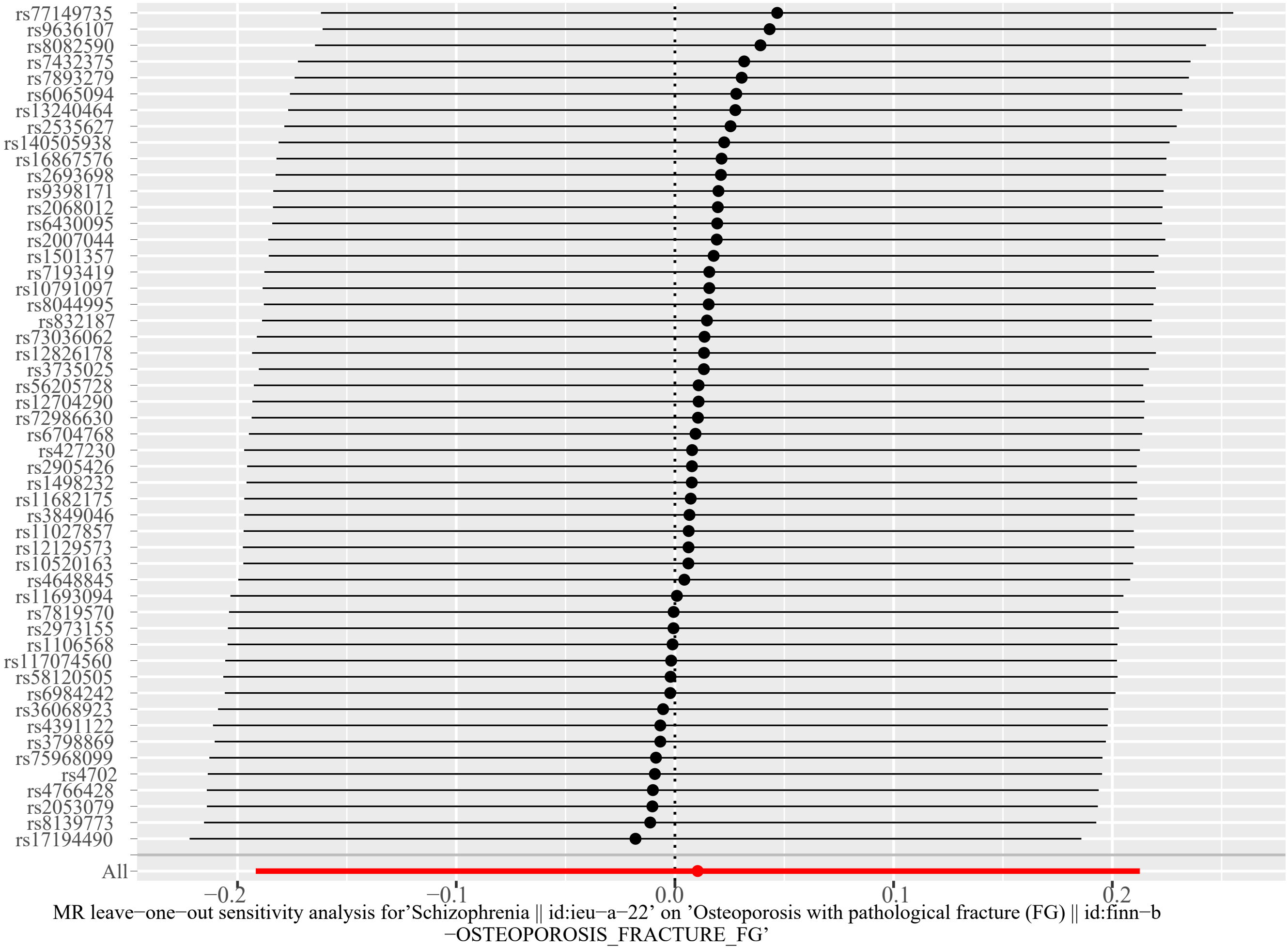

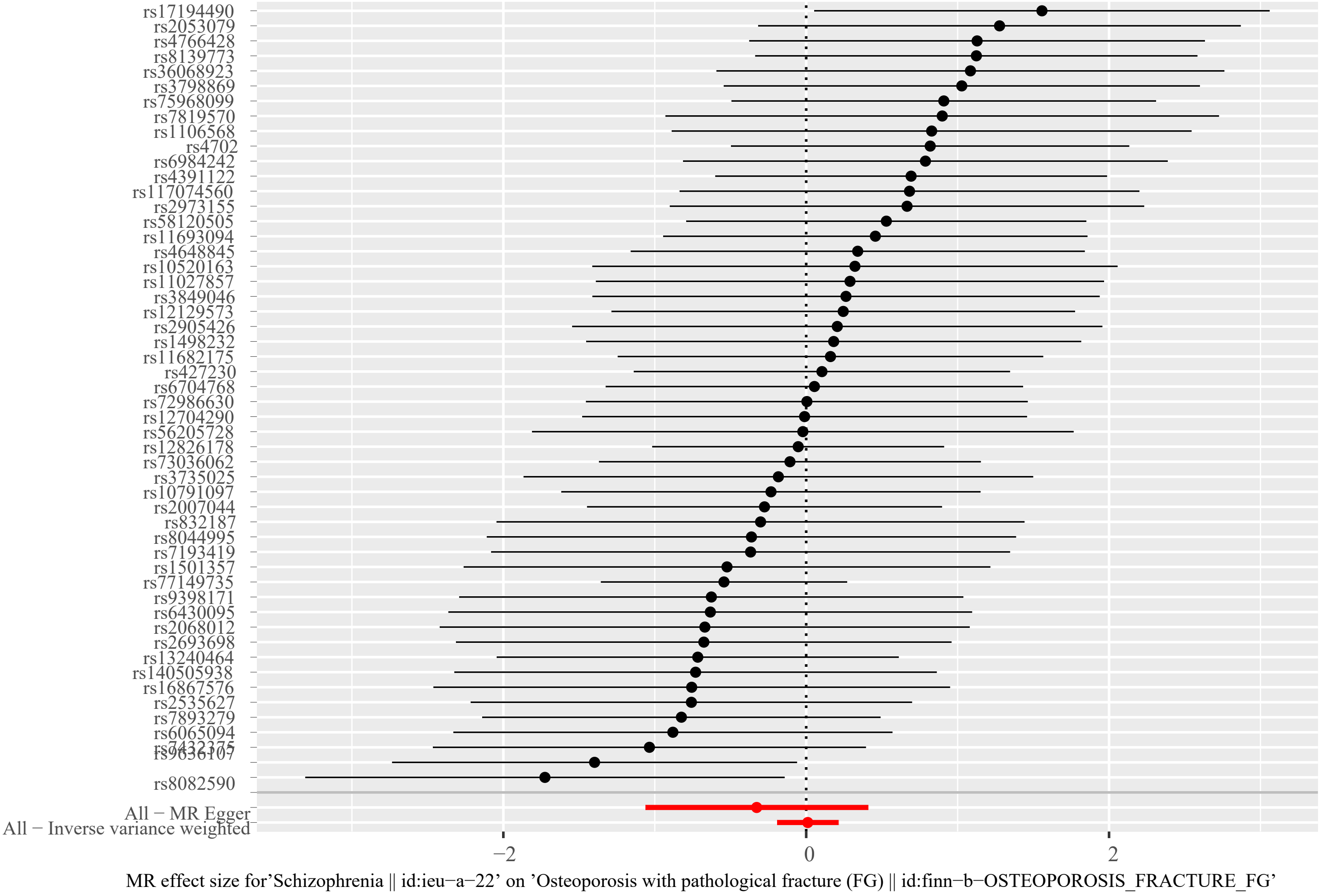

rs17194490
rs2053079
rs4766428
rs8139773
rs36068923
rs3798869
rs75968099
rs7819570
rs1106568
rs4702
rs6984242
rs4391122
rs117074560
rs2973155
rs58120505
rs11693094
rs4648845
rs10520163
rs11027857
rs3849046
rs12129573
rs2905426
rs1498232
rs11682175
rs427230
rs6704768
rs72986630
rs12704290
rs56205728
rs12826178
rs73036062
rs3735025
rs10791097
rs2007044
rs832187
rs8044995
rs7193419
rs1501357
rs77149735
rs9398171
rs6430095
rs2068012
rs2693698
rs13240464
rs140505938
rs16867576
rs2535627
rs7893279
rs6065094
rs7432375
rs9636107
rs8082590
All − MR Egger
All − Inverse variance weighted
−2
0
2
MR effect size for’Schizophrenia || id:ieu−a−22’ on ’Osteoporosis with pathological fracture (FG) || id:finn−b−OSTEOPOROSIS_FRACTURE_FG’

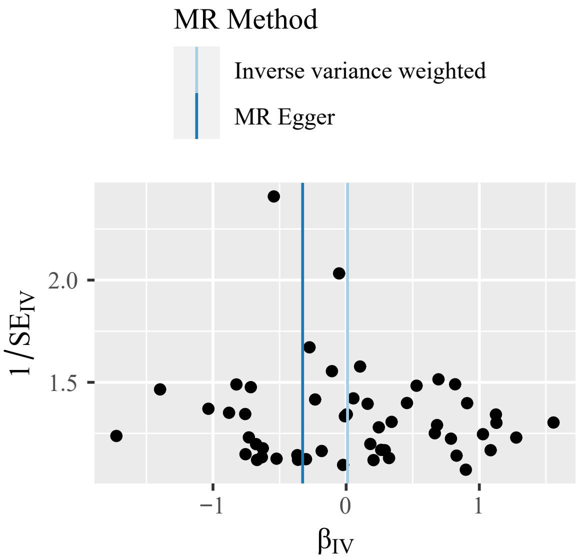
MR Method
Inverse variance weighted
MR Egger
2.0
1.5
$1/SE_{IV}$
−1
0
1
β_{IV}

Figure S3. Leave-one-out analysis, MR effect size and funnel plot for SHC on TB-BMD.

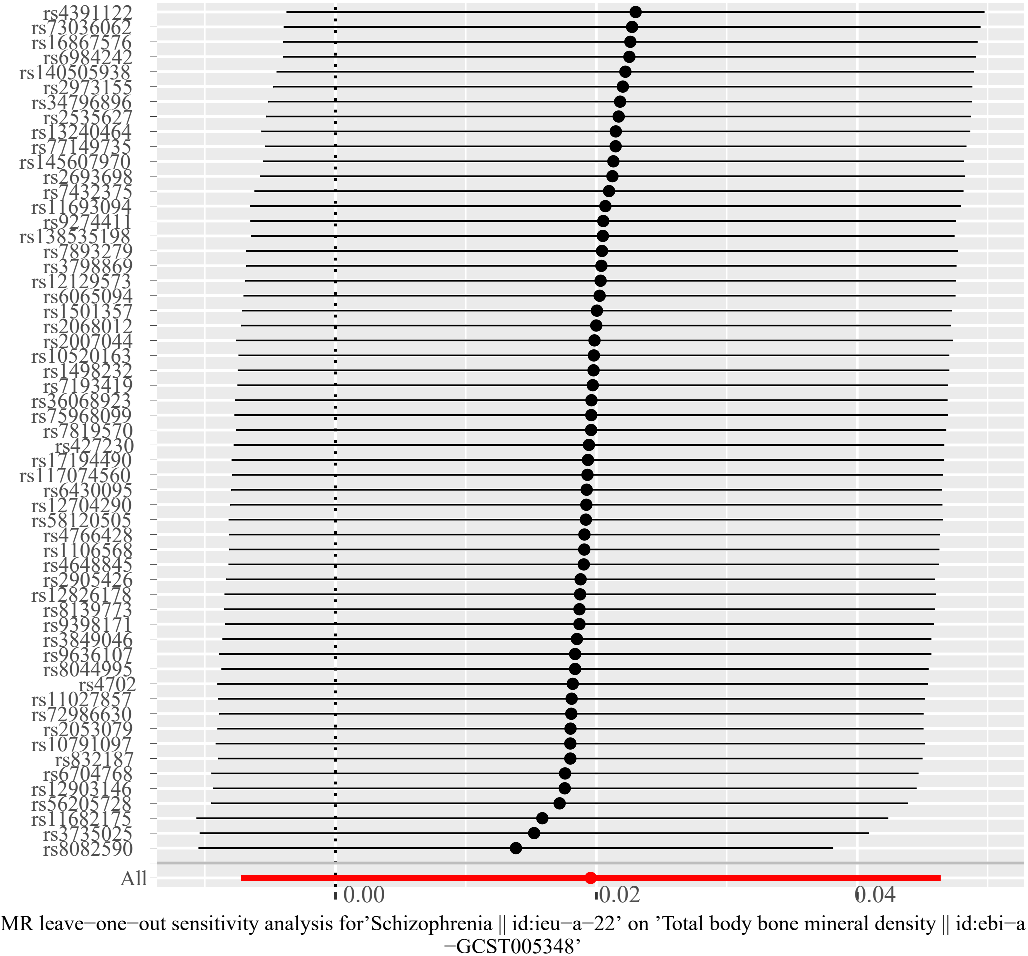

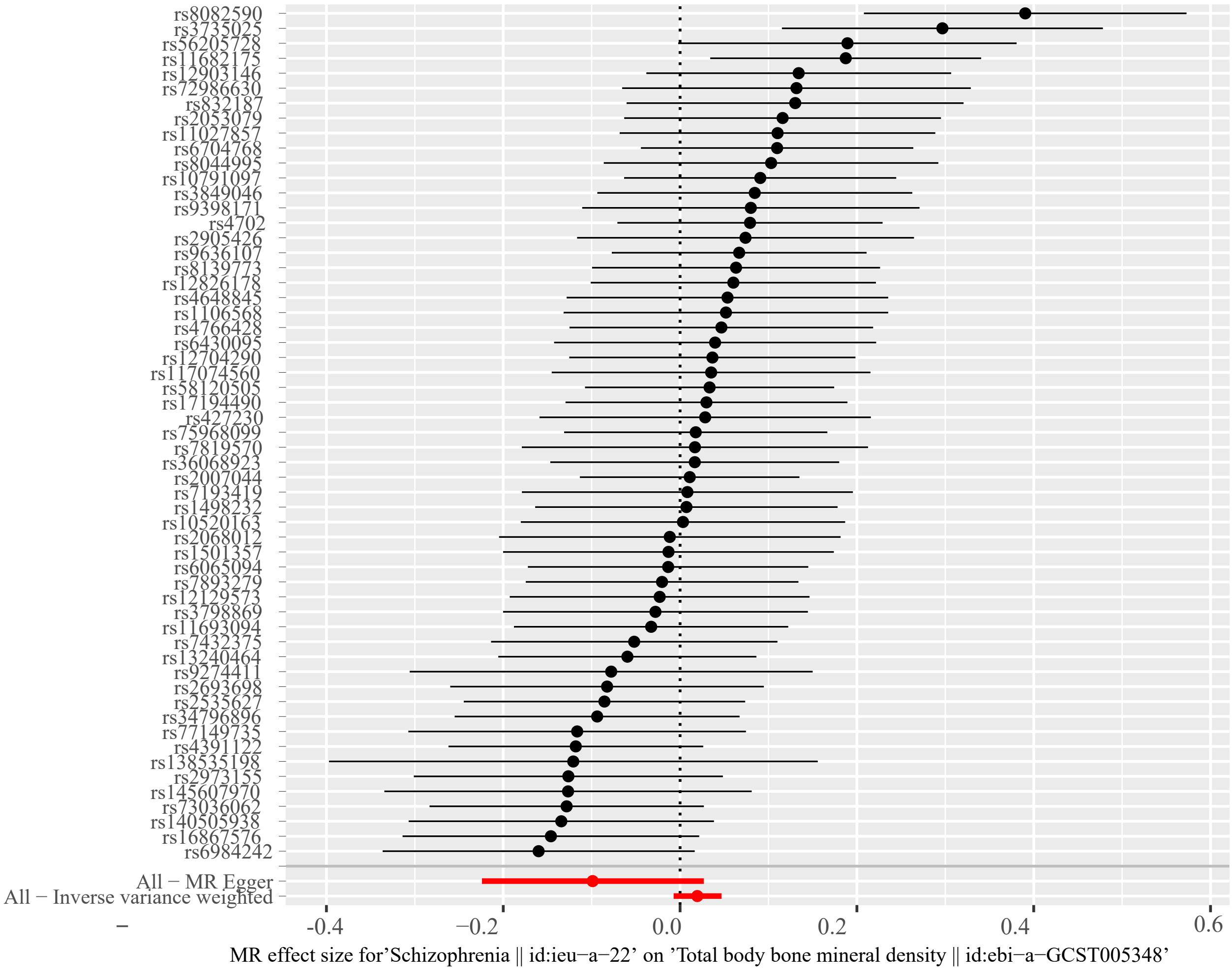
rs8082590
rs3735025
rs56205728
rs11682175
rs12903146
rs72986630
rs832187
rs2053079
rs11027857
rs6704768
rs8044995
rs10791097
rs3849046
rs9398171
rs4702
rs2905426
rs9636107
rs8139773
rs12826178
rs4648845
rs1106568
rs4766428
rs6430095
rs12704290
rs117074560
rs58120505
rs17194490
rs427230
rs75968099
rs7819570
rs36068923
rs2007044
rs7193419
rs1498232
rs10520163
rs2068012
rs1501357
rs6065094
rs7893279
rs12129573
rs3798869
rs11693094
rs7432375
rs13240464
rs9274411
rs2693698
rs2535627
rs34796896
rs77149735
rs4391122
rs138535198
rs2973155
rs145607970
rs73036062
rs140505938
rs16867576
rs6984242
All − MR Egger
All − Inverse variance weighted
−
-0.4
−0.2
0.0
0.2
0.4
0.6
MR effect size for’Schizophrenia || id:ieu−a−22’ on ’Total body bone mineral density || id:ebi−a−GCST005348’

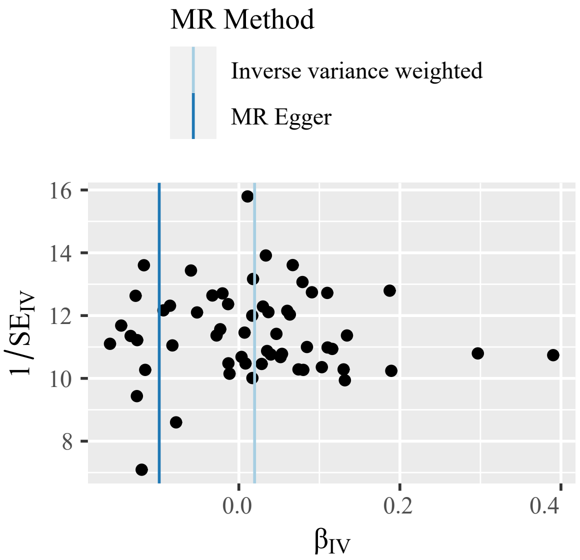
MR Method
Inverse variance weighted
MR Egger
16
14
12
10
8
$1/SE_{IV}$
0.0
0.2
0.4
β_{IV}

Figure S4. Leave-one-out analysis, MR effect size and funnel plot for SHC on FN-BMD.

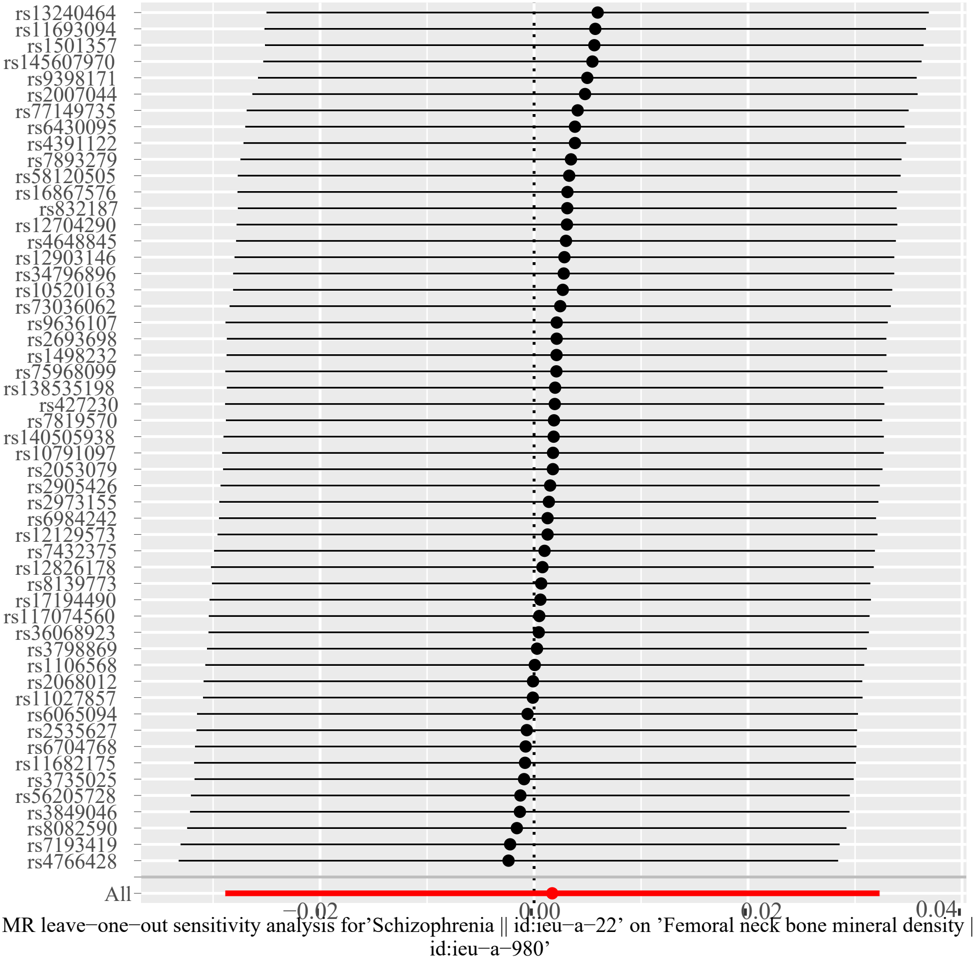

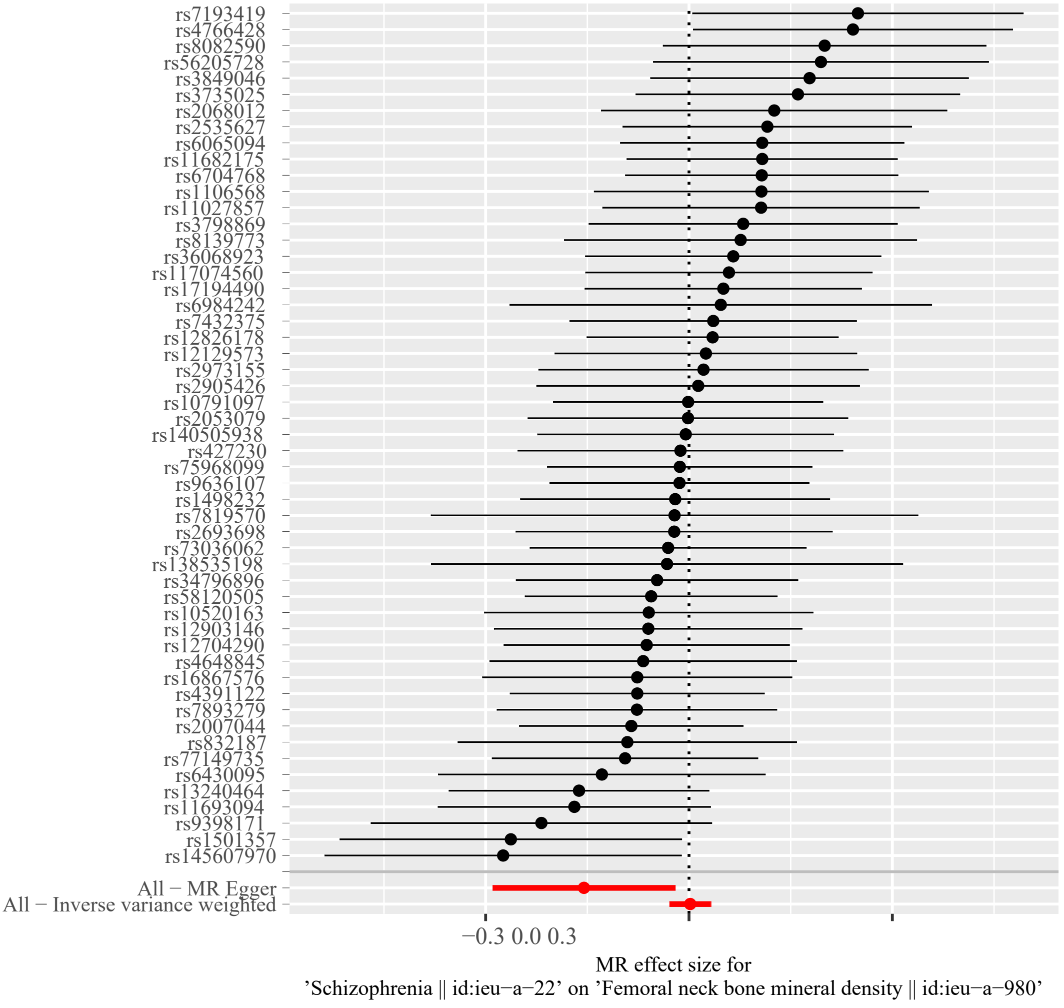

rs7193419
rs4766428
rs8082590
rs56205728
rs3849046
rs3735025
rs2068012
rs2535627
rs6065094
rs11682175
rs6704768
rs1106568
rs11027857
rs3798869
rs8139773
rs36068923
rs117074560
rs17194490
rs6984242
rs7432375
rs12826178
rs12129573
rs2973155
rs2905426
rs10791097
rs2053079
rs140505938
rs427230
rs75968099
rs9636107
rs1498232
rs7819570
rs2693698
rs73036062
rs138535198
rs34796896
rs58120505
rs10520163
rs12903146
rs12704290
rs4648845
rs16867576
rs4391122
rs7893279
rs2007044
rs832187
rs77149735
rs6430095
rs13240464
rs11693094
rs9398171
rs1501357
rs145607970
All − MR Egger
All − Inverse variance weighted
−0.3 0.0 0.3
MR effect size for
'Schizophrenia || id:ieu−a−22' on 'Femoral neck bone mineral density || id:ieu−a−980'

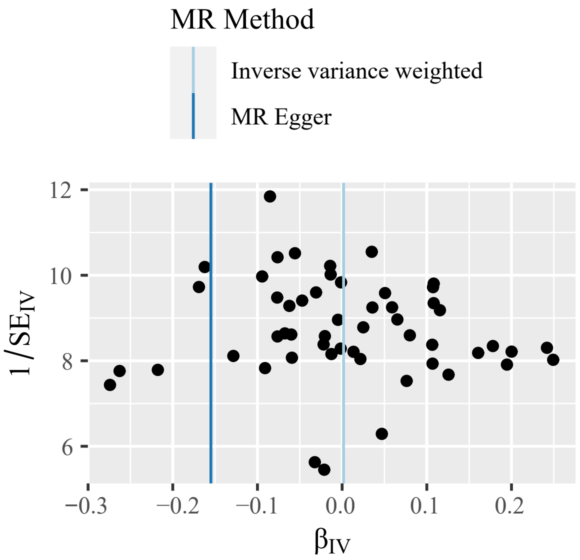

MR Method
Inverse variance weighted
MR Egger
12
10
8
6
1/SE_IV
−0.3
−0.2
−0.1
0.0
0.1
0.2
β_IV

Figure S5. Leave-one-out analysis, MR effect size and funnel plot for SHC on LS-BMD.

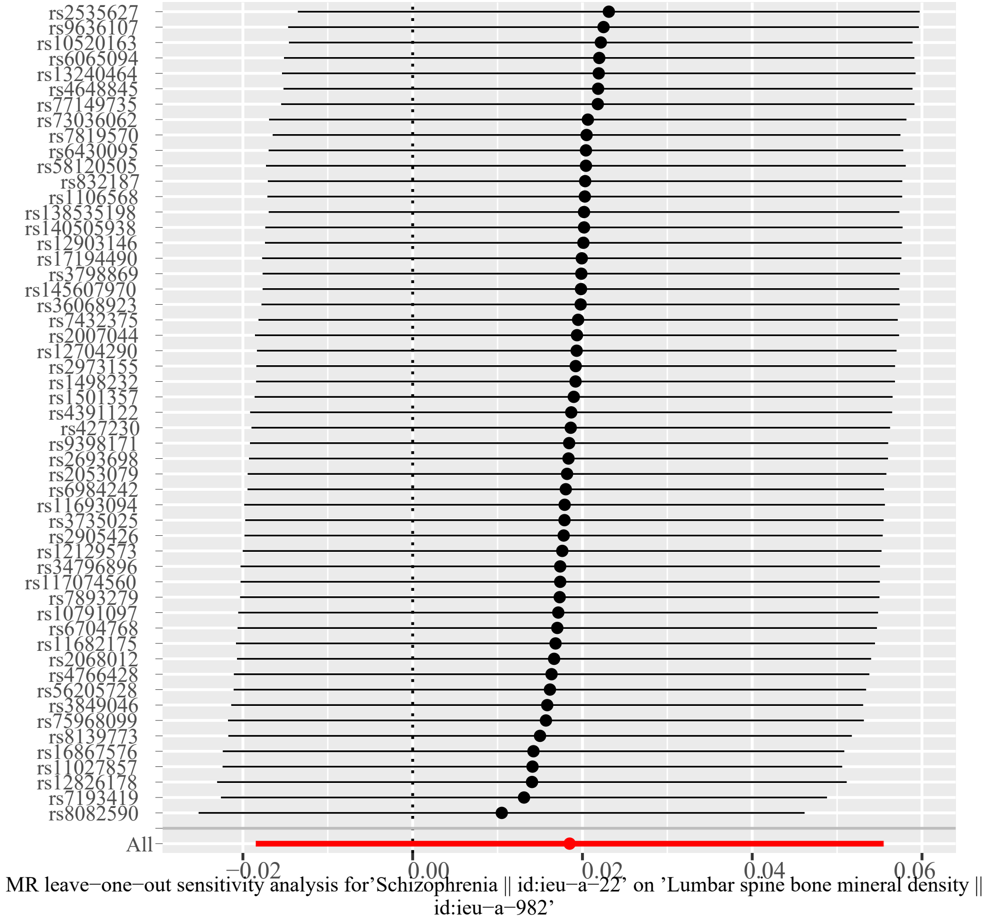

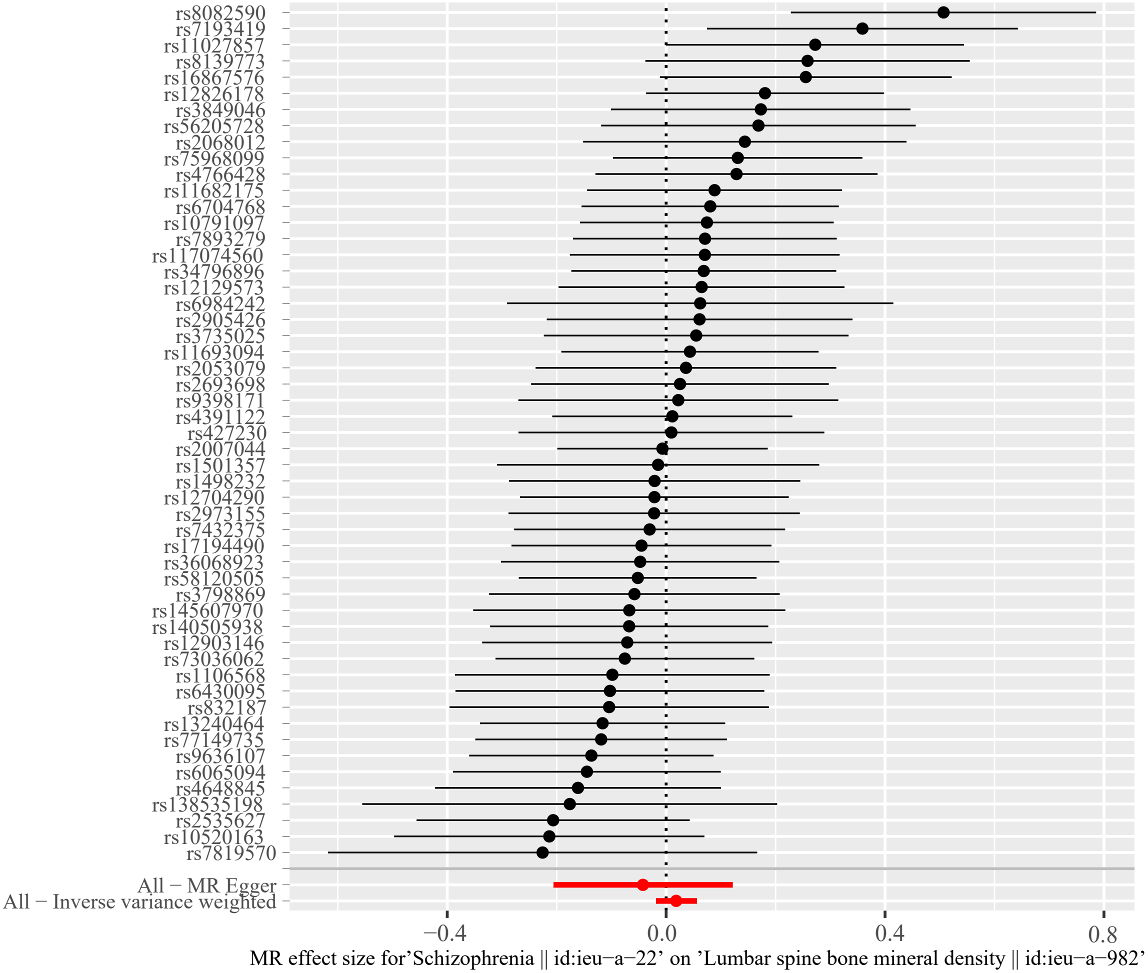
rs8082590
rs7193419
rs11027857
rs8139773
rs16867576
rs12826178
rs3849046
rs56205728
rs2068012
rs75968099
rs4766428
rs11682175
rs6704768
rs10791097
rs7893279
rs117074560
rs34796896
rs12129573
rs6984242
rs2905426
rs3735025
rs11693094
rs2053079
rs2693698
rs9398171
rs4391122
rs427230
rs2007044
rs1501357
rs1498232
rs12704290
rs2973155
rs7432375
rs17194490
rs36068923
rs58120505
rs3798869
rs145607970
rs140505938
rs12903146
rs73036062
rs1106568
rs6430095
rs832187
rs13240464
rs77149735
rs9636107
rs6065094
rs4648845
rs138535198
rs2535627
rs10520163
rs7819570
All − MR Egger
All − Inverse variance weighted
−0.4
0.0
0.4
0.8
MR effect size for'Schizophrenia || id:ieu−a−22' on 'Lumbar spine bone mineral density || id:ieu−a−982'

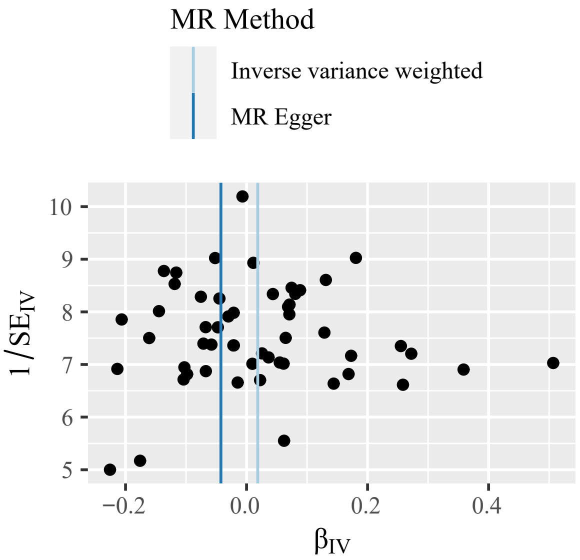
MR Method
Inverse variance weighted
MR Egger
10
9
8
7
6
5
1/SE_IV
−0.2
0.0
0.2
0.4
β_IV

Figure S6. Leave-one-out analysis, MR effect size and funnel plot for SHC on FA-BMD.

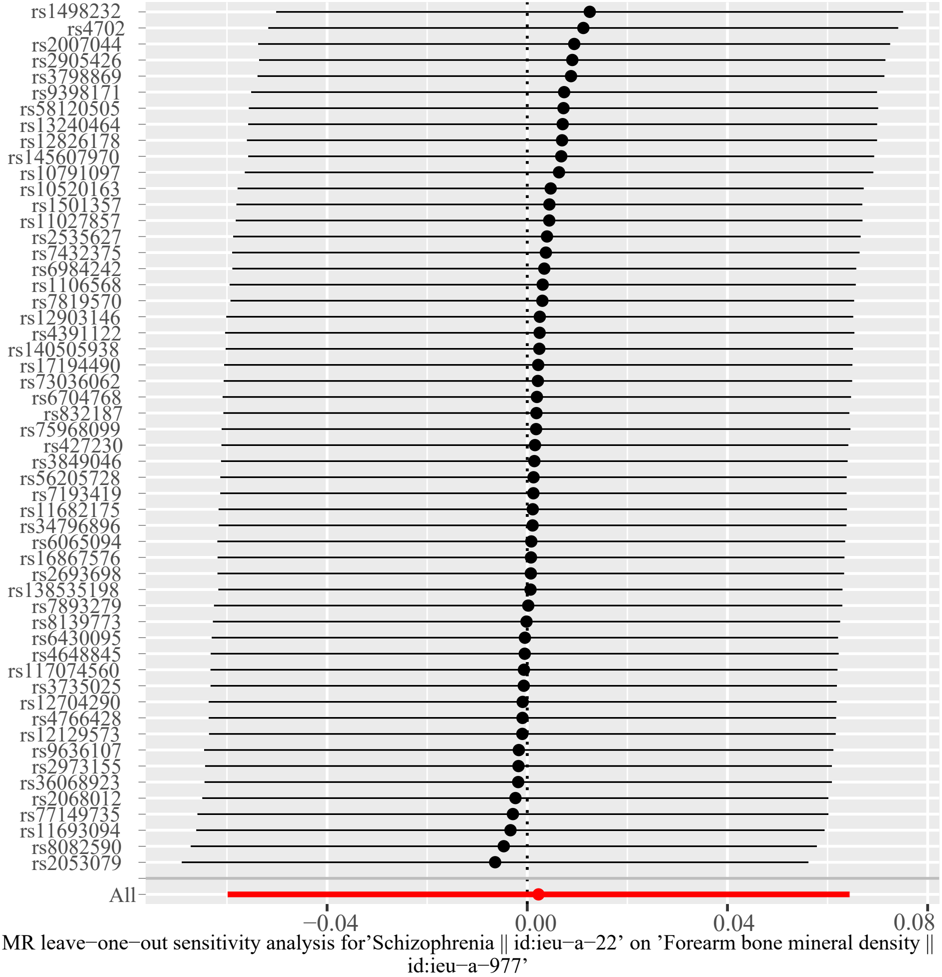

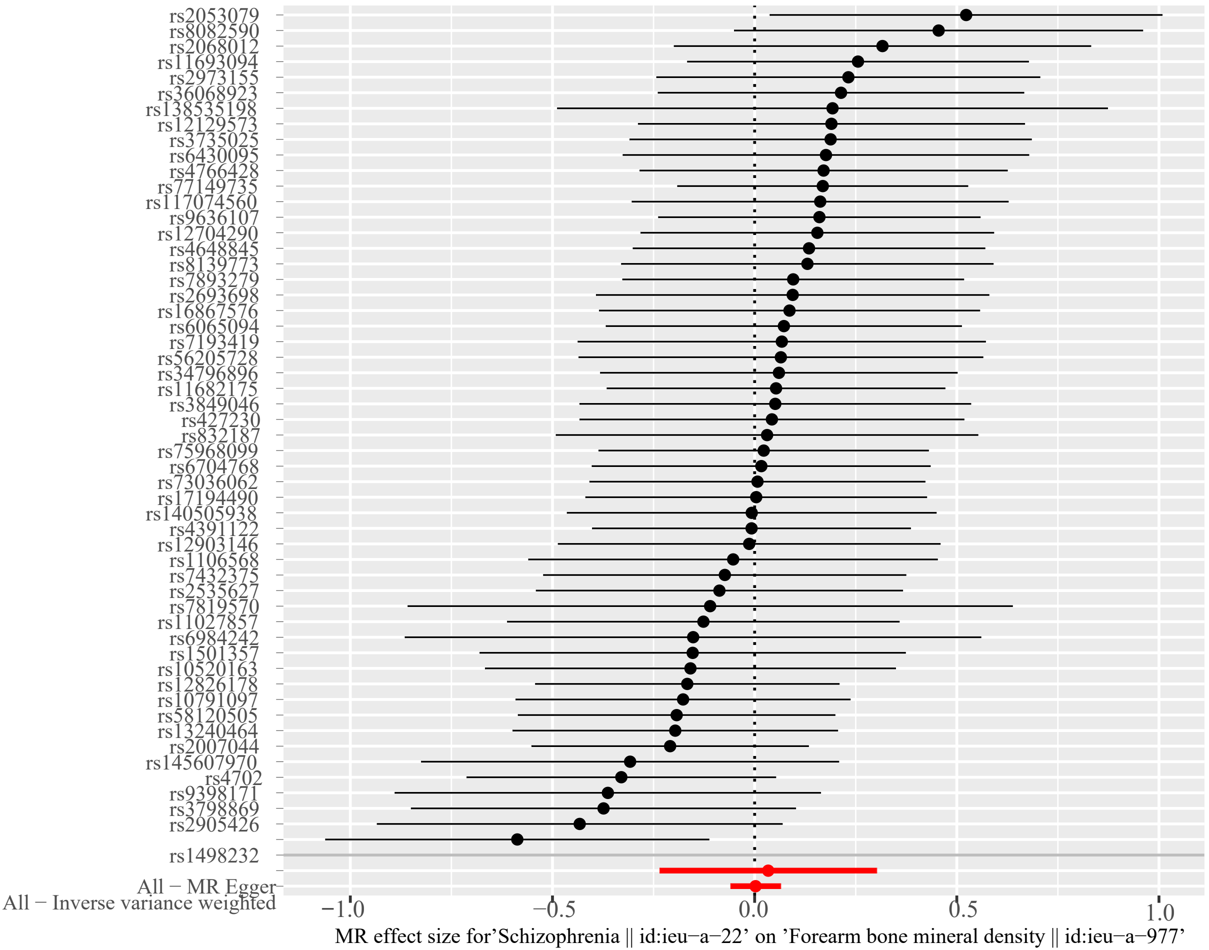
rs2053079
rs8082590
rs2068012
rs11693094
rs2973155
rs36068923
rs138535198
rs12129573
rs3735025
rs6430095
rs4766428
rs77149735
rs117074560
rs9636107
rs12704290
rs4648845
rs8139773
rs7893279
rs2693698
rs16867576
rs6065094
rs7193419
rs56205728
rs34796896
rs11682175
rs3849046
rs427230
rs832187
rs75968099
rs6704768
rs73036062
rs17194490
rs140505938
rs4391122
rs12903146
rs1106568
rs7432375
rs2535627
rs7819570
rs11027857
rs6984242
rs1501357
rs10520163
rs12826178
rs10791097
rs58120505
rs13240464
rs2007044
rs145607970
rs4702
rs9398171
rs3798869
rs2905426
rs1498232
All − MR Egger
All − Inverse variance weighted
−1.0
−0.5
0.0
0.5
1.0
MR effect size for'Schizophrenia || id:ieu−a−22' on 'Forearm bone mineral density || id:ieu−a−977'

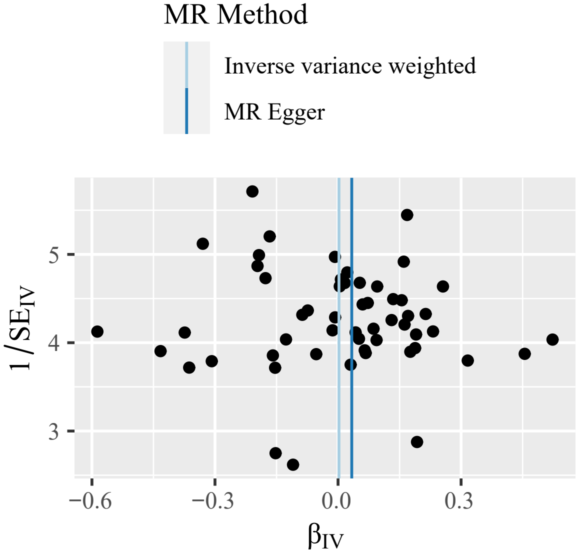
MR Method
Inverse variance weighted
MR Egger
1/SE_IV
β_IV
5
4
3
−0.6
−0.3
0.0
0.3

Figure S7. Leave-one-out analysis, MR effect size and funnel plot for SHC on eBMD.

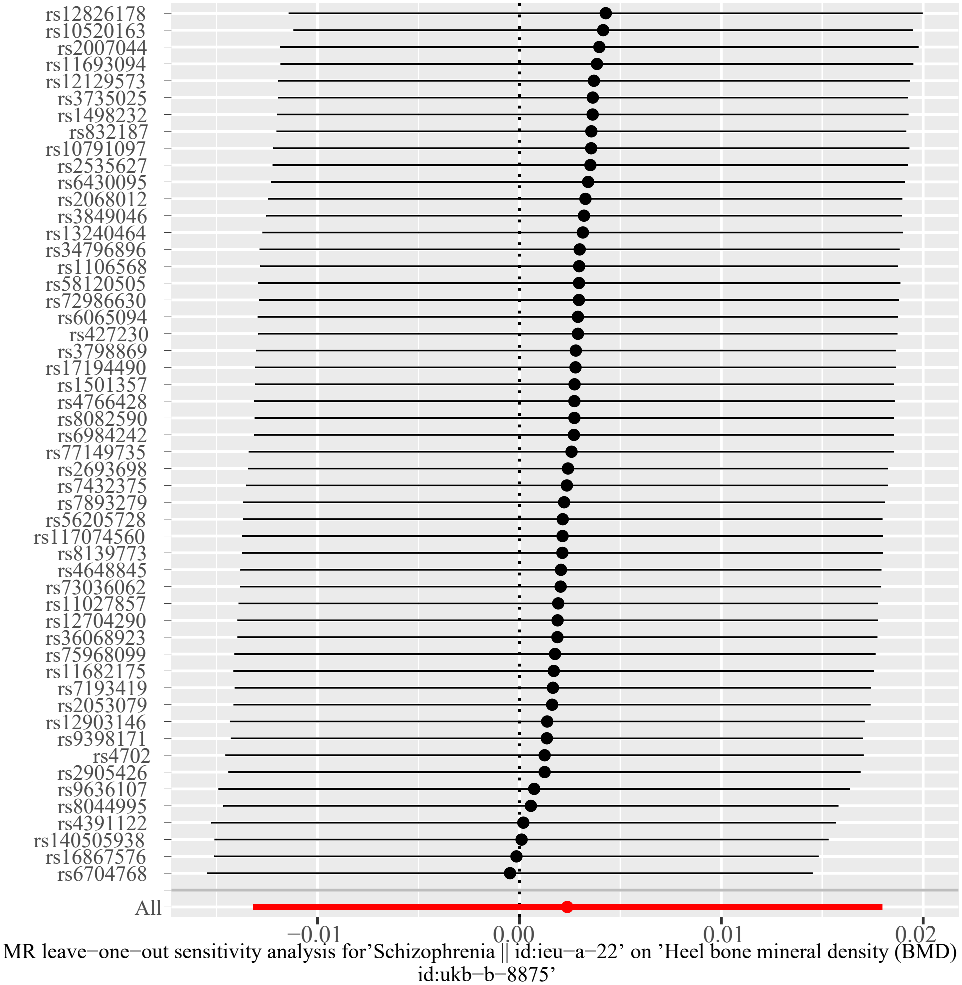

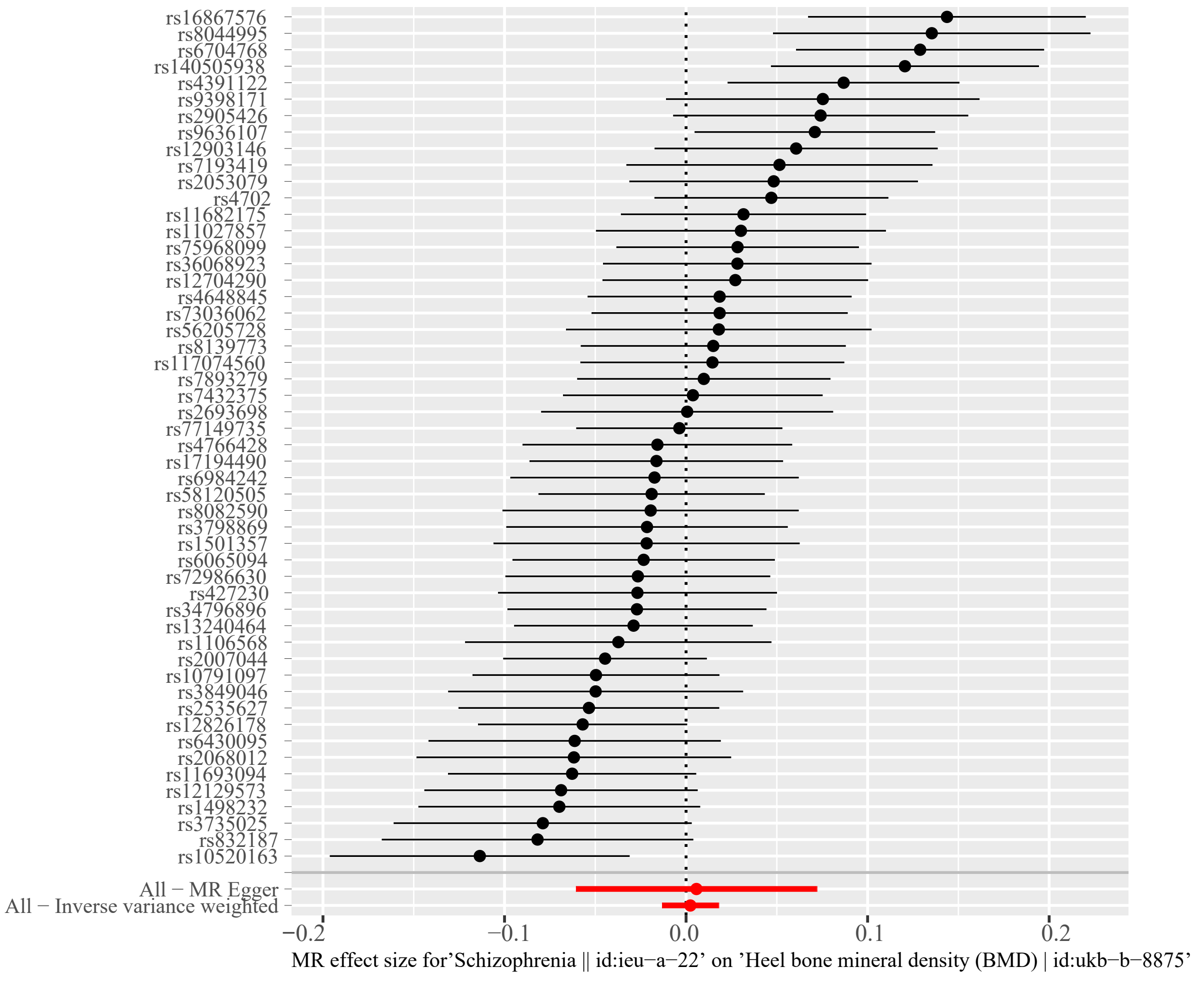
rs16867576
rs8044995
rs6704768
rs140505938
rs4391122
rs9398171
rs2905426
rs9636107
rs12903146
rs7193419
rs2053079
rs4702
rs11682175
rs11027857
rs75968099
rs36068923
rs12704290
rs4648845
rs73036062
rs56205728
rs8139773
rs117074560
rs7893279
rs7432375
rs2693698
rs77149735
rs4766428
rs17194490
rs6984242
rs58120505
rs8082590
rs3798869
rs1501357
rs6065094
rs72986630
rs427230
rs34796896
rs13240464
rs1106568
rs2007044
rs10791097
rs3849046
rs2535627
rs12826178
rs6430095
rs2068012
rs11693094
rs12129573
rs1498232
rs3735025
rs832187
rs10520163
All − MR Egger
All − Inverse variance weighted
−0.2
−0.1
0.0
0.1
0.2
MR effect size for'Schizophrenia || id:ieu−a−22' on 'Heel bone mineral density (BMD) | id:ukb−b−8875'

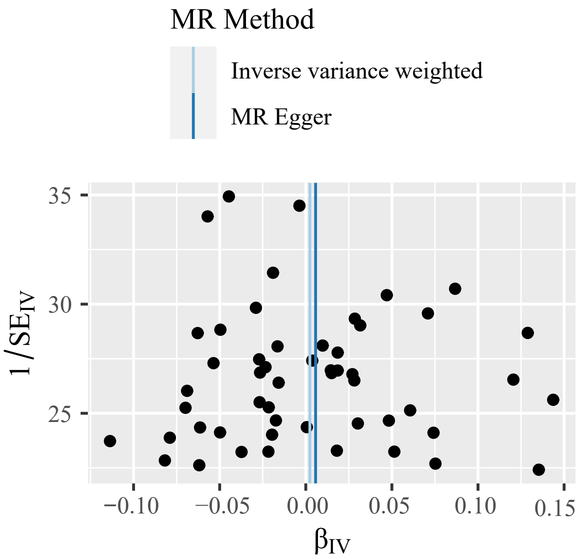
MR Method
Inverse variance weighted
MR Egger
35
30
25
1/SE_IV
−0.10
−0.05
0.00
0.05
0.10
0.15
β_IV

Figure S8. Leave-one-out analysis, MR effect size and funnel plot for SHC on LF.

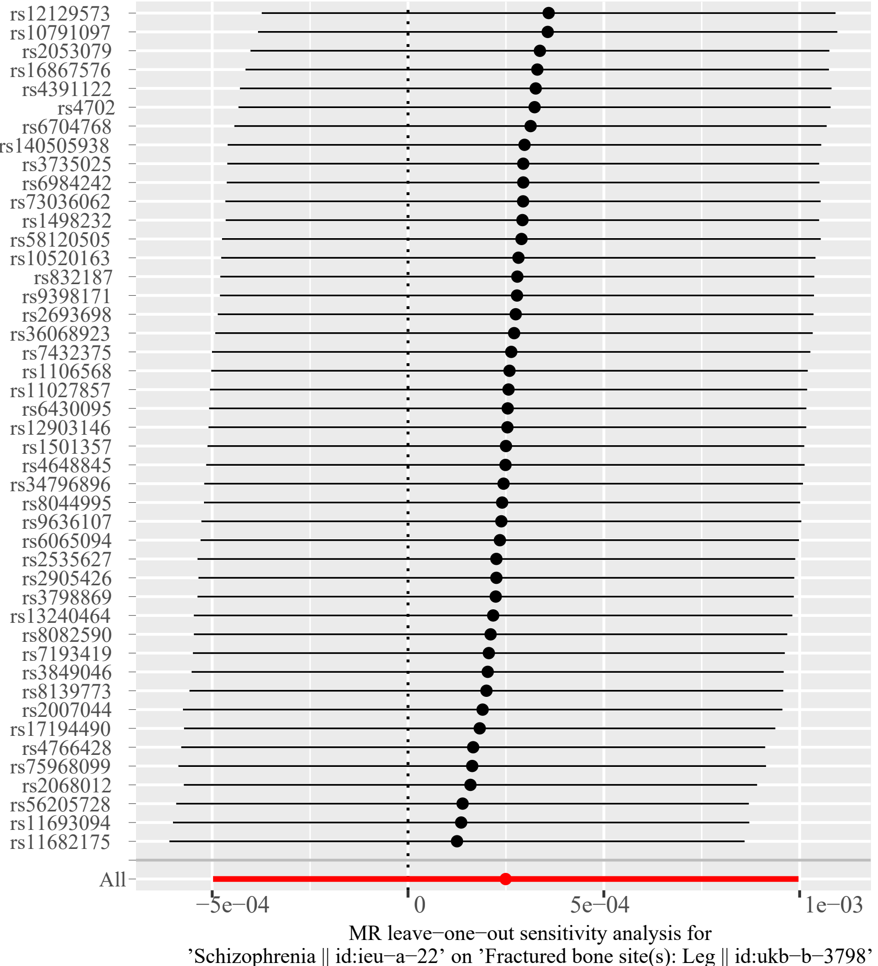

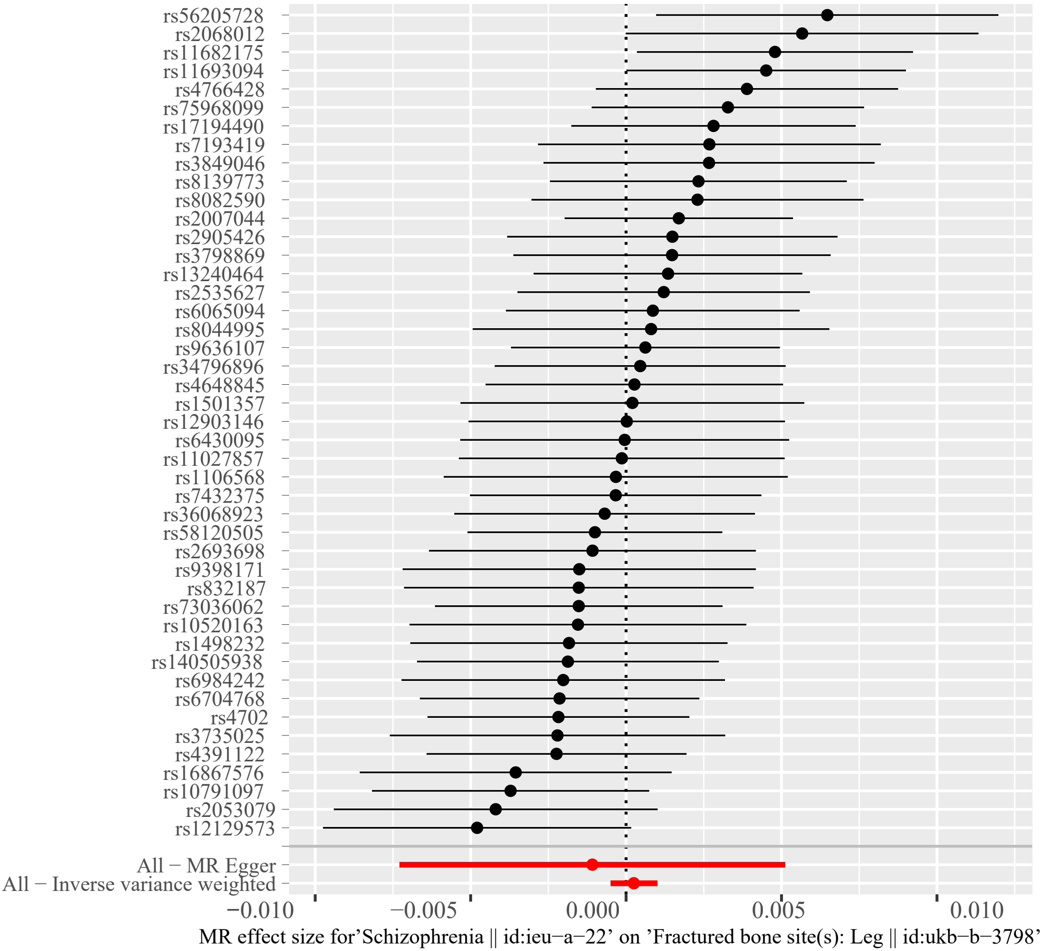

rs56205728
rs2068012
rs11682175
rs11693094
rs4766428
rs75968099
rs17194490
rs7193419
rs3849046
rs8139773
rs8082590
rs2007044
rs2905426
rs3798869
rs13240464
rs2535627
rs6065094
rs8044995
rs9636107
rs34796896
rs4648845
rs1501357
rs12903146
rs6430095
rs11027857
rs1106568
rs7432375
rs36068923
rs58120505
rs2693698
rs9398171
rs832187
rs73036062
rs10520163
rs1498232
rs140505938
rs6984242
rs6704768
rs4702
rs3735025
rs4391122
rs16867576
rs10791097
rs2053079
rs12129573
All − MR Egger
All − Inverse variance weighted
−0.010
−0.005
0.000
0.005
0.010
MR effect size for'Schizophrenia || id:ieu−a−22' on 'Fractured bone site(s): Leg || id:ukb−b−3798'

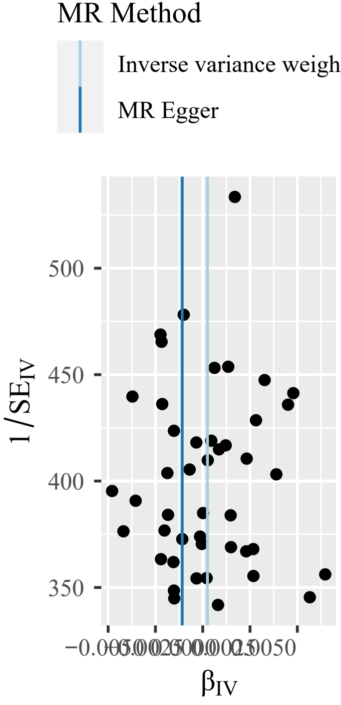
MR Method
Inverse variance weigh
MR Egger
500
450
400
350
1/SE_IV
β_IV

Figure S9. Leave-one-out analysis, MR effect size and funnel plot for SHC on AF.

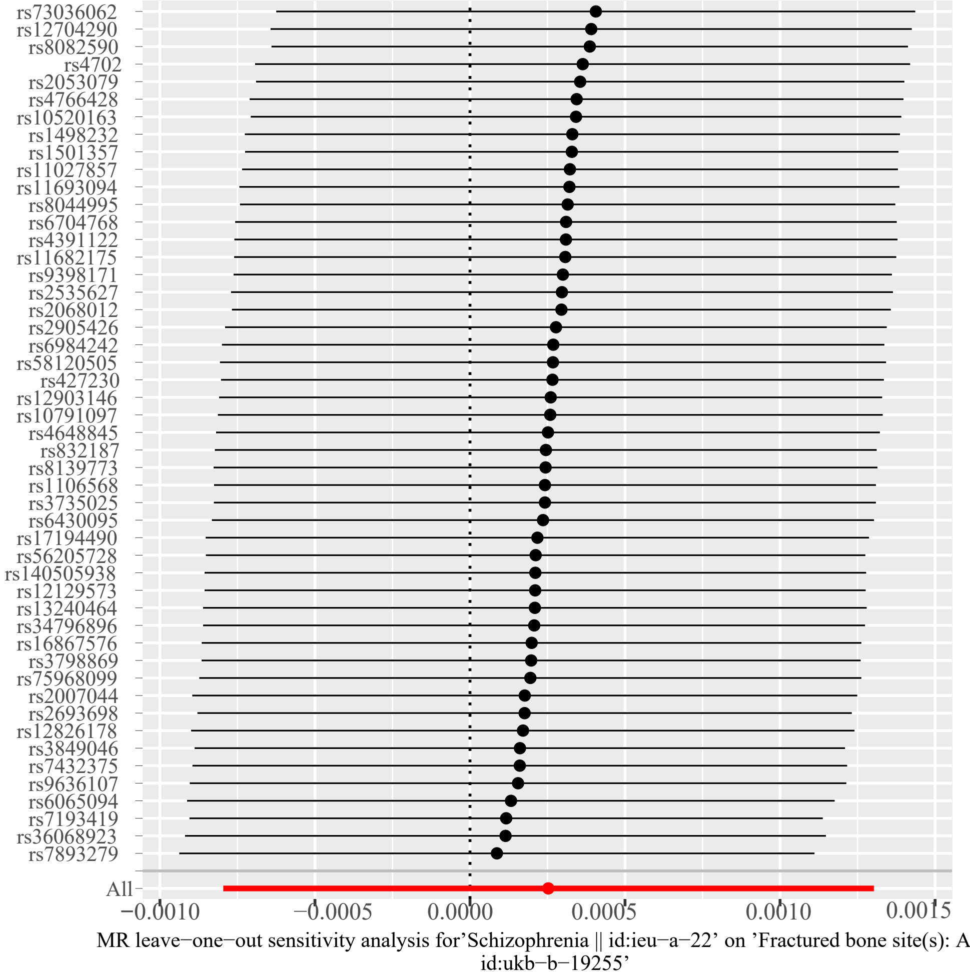

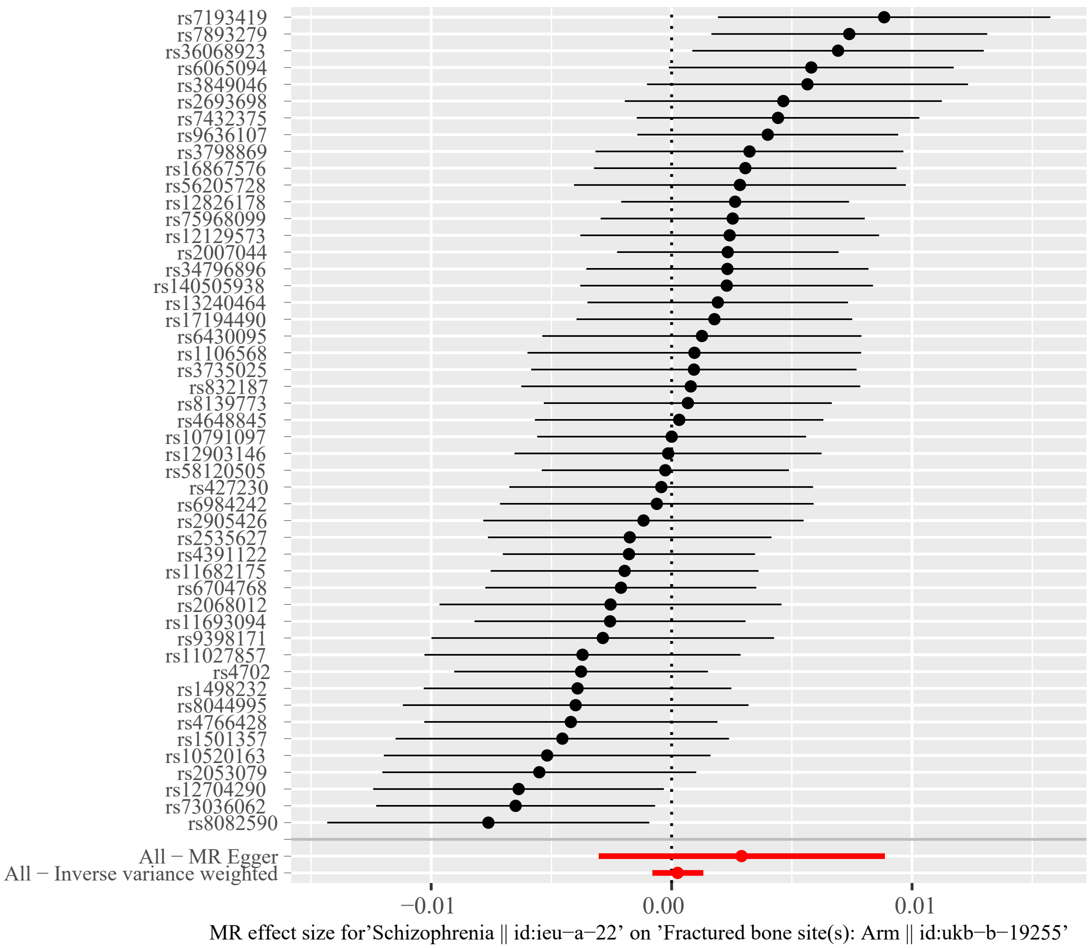

rs7193419
rs7893279
rs36068923
rs6065094
rs3849046
rs2693698
rs7432375
rs9636107
rs3798869
rs16867576
rs56205728
rs12826178
rs75968099
rs12129573
rs2007044
rs34796896
rs140505938
rs13240464
rs17194490
rs6430095
rs1106568
rs3735025
rs832187
rs8139773
rs4648845
rs10791097
rs12903146
rs58120505
rs427230
rs6984242
rs2905426
rs2535627
rs4391122
rs11682175
rs6704768
rs2068012
rs11693094
rs9398171
rs11027857
rs4702
rs1498232
rs8044995
rs4766428
rs1501357
rs10520163
rs2053079
rs12704290
rs73036062
rs8082590
All − MR Egger
All − Inverse variance weighted
−0.01
0.00
0.01
MR effect size for'Schizophrenia || id:ieu−a−22' on 'Fractured bone site(s): Arm || id:ukb−b−19255'

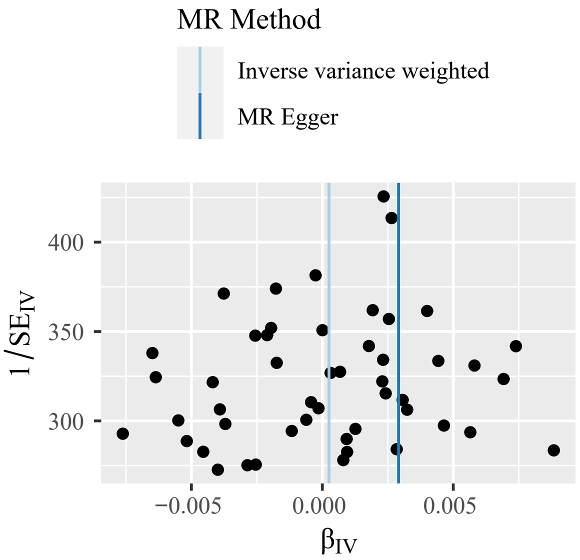
MR Method
Inverse variance weighted
MR Egger
400
350
300
1/SE_IV
−0.005
0.000
0.005
β_IV

Figure S10. Leave-one-out analysis, MR effect size and funnel plot for SHC on SF.

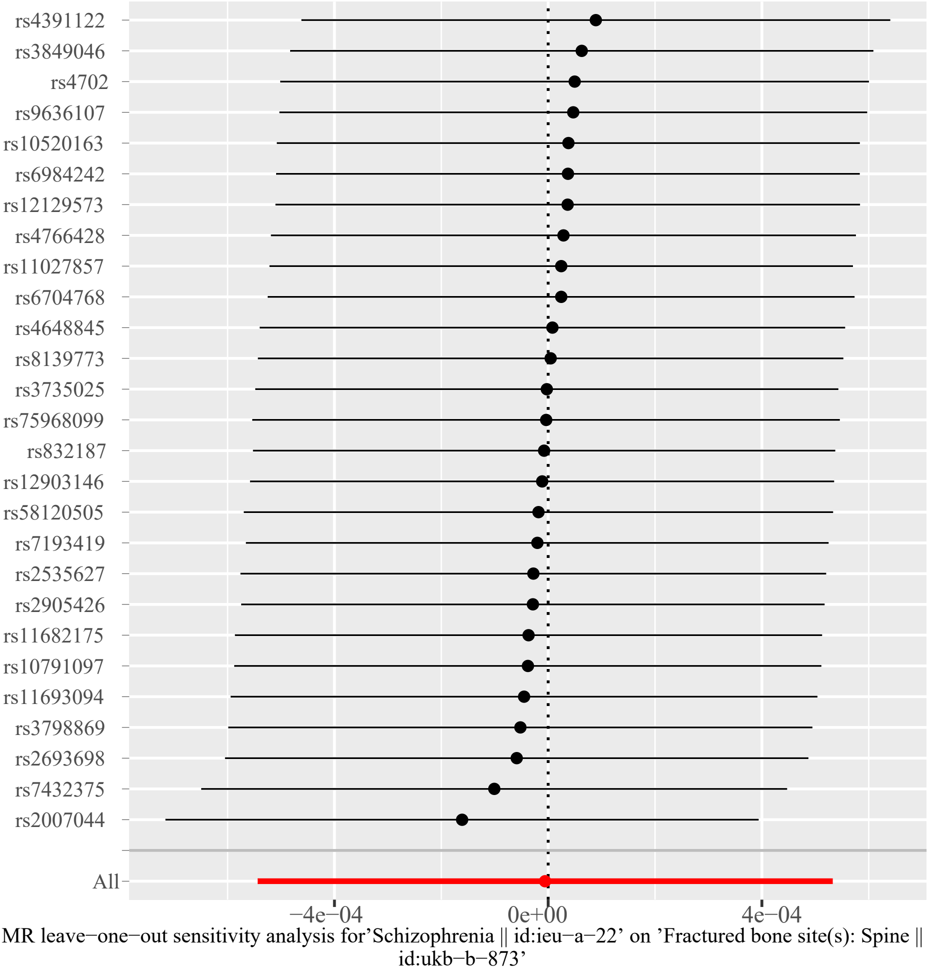

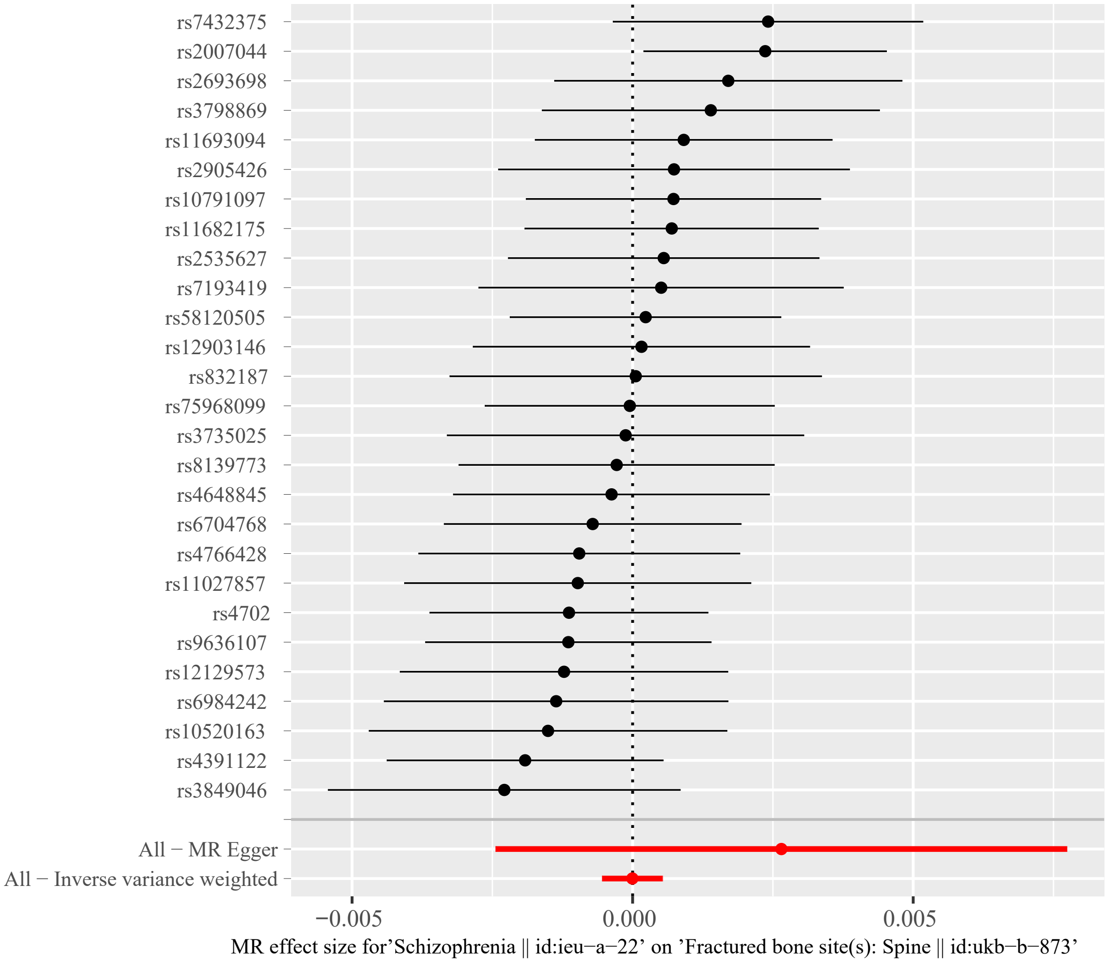
rs7432375
rs2007044
rs2693698
rs3798869
rs11693094
rs2905426
rs10791097
rs11682175
rs2535627
rs7193419
rs58120505
rs12903146
rs832187
rs75968099
rs3735025
rs8139773
rs4648845
rs6704768
rs4766428
rs11027857
rs4702
rs9636107
rs12129573
rs6984242
rs10520163
rs4391122
rs3849046
All − MR Egger
All − Inverse variance weighted
−0.005
0.000
0.005
MR effect size for'Schizophrenia || id:ieu−a−22' on 'Fractured bone site(s): Spine || id:ukb−b−873'

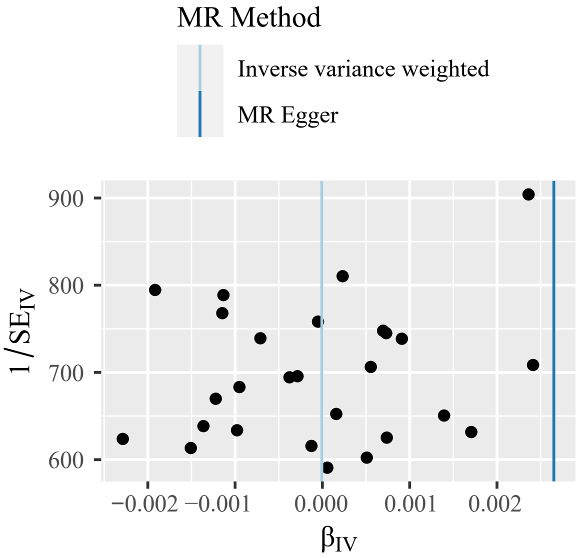
MR Method
Inverse variance weighted
MR Egger
900
800
700
600
1/SE_IV
−0.002
−0.001
0.000
0.001
0.002
β_IV

Figure S11. Leave-one-out analysis, MR effect size and funnel plot for SHC on HF.

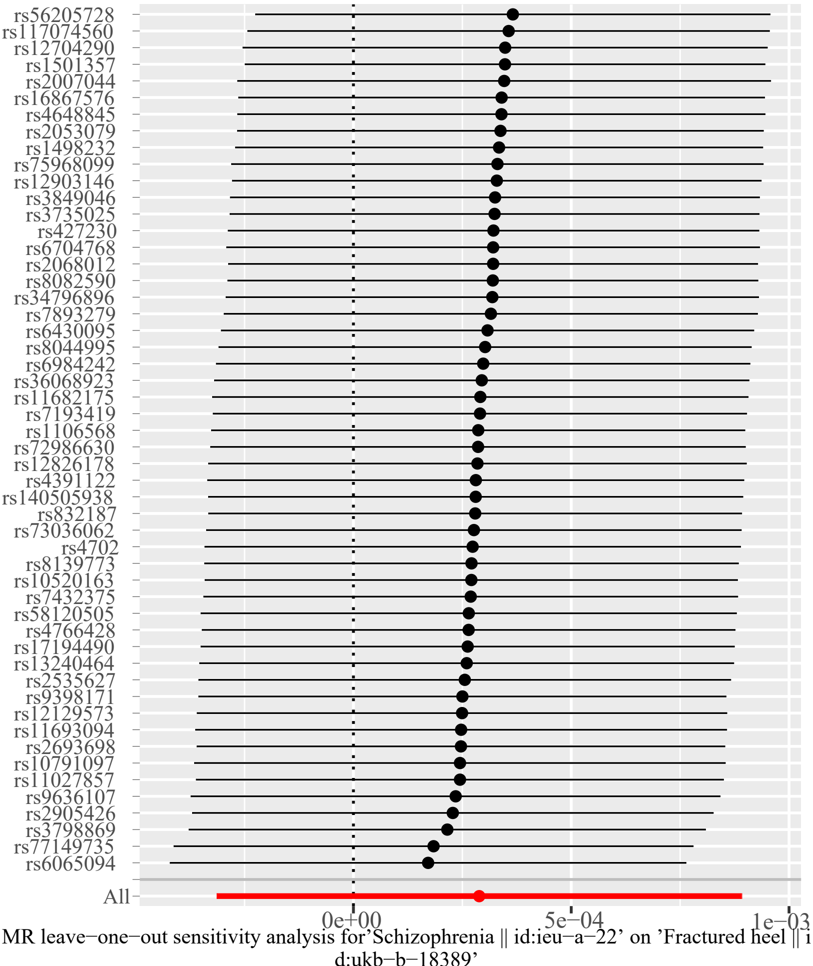

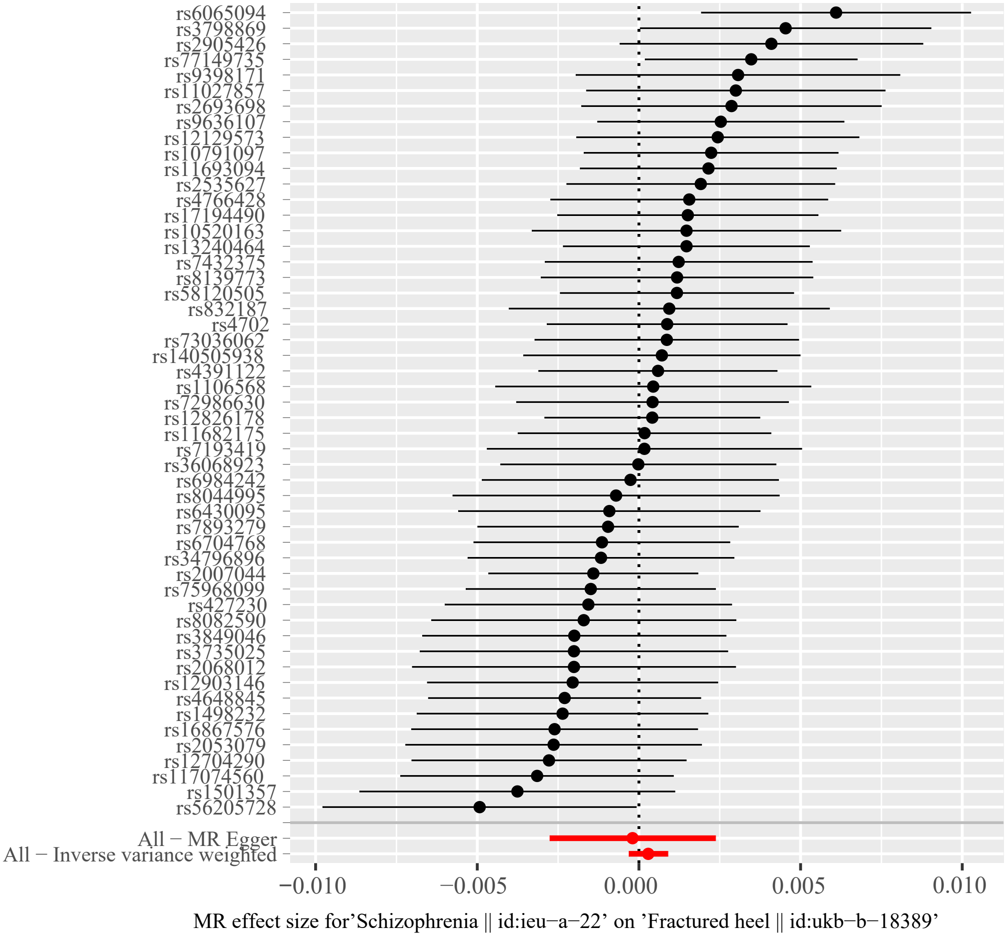

rs6065094
rs3798869
rs2905426
rs77149735
rs9398171
rs11027857
rs2693698
rs9636107
rs12129573
rs10791097
rs11693094
rs2535627
rs4766428
rs17194490
rs10520163
rs13240464
rs7432375
rs8139773
rs58120505
rs832187
rs4702
rs73036062
rs140505938
rs4391122
rs1106568
rs72986630
rs12826178
rs11682175
rs7193419
rs36068923
rs6984242
rs8044995
rs6430095
rs7893279
rs6704768
rs34796896
rs2007044
rs75968099
rs427230
rs8082590
rs3849046
rs3735025
rs2068012
rs12903146
rs4648845
rs1498232
rs16867576
rs2053079
rs12704290
rs117074560
rs1501357
rs56205728
All − MR Egger
All − Inverse variance weighted
−0.010
−0.005
0.000
0.005
0.010
MR effect size for’Schizophrenia || id:ieu−a−22’ on ’Fractured heel || id:ukb−b−18389’

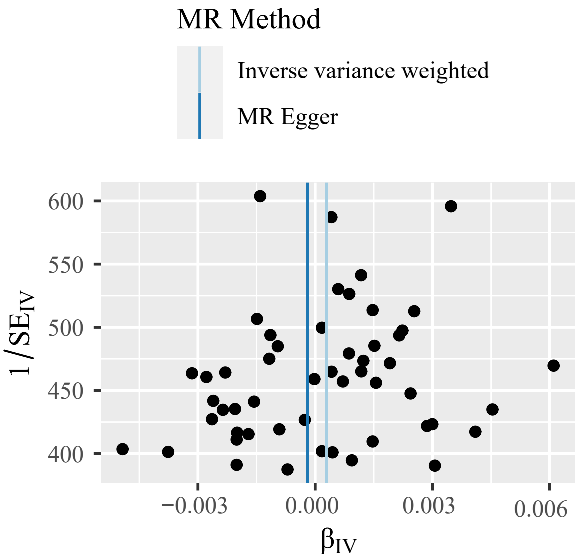
MR Method
Inverse variance weighted
MR Egger
600
550
500
450
400
1/SE$_{IV}$
−0.003
0.000
0.003
0.006
β$_{IV}$
