# Supplementary material for: Mendelian randomization analysis does not reveal a causal influence of mental diseases on osteoporosis
Source: Front Endocrinol (Lausanne). 2023 Apr 20;14:1125427. doi: 10.3389/fendo.2023.1125427 (PMC10157183; doi:10.3389/fendo.2023.1125427)
Supplement: Supplementary Material 1 — Instrumental variables SNPs. [file DataSheet_1.zip › Supplementary Material/Supplementary Material 4.pdf]

Figure S1 Leave-one-out analysis, MR effect size and funnel plot for MDD on OP.

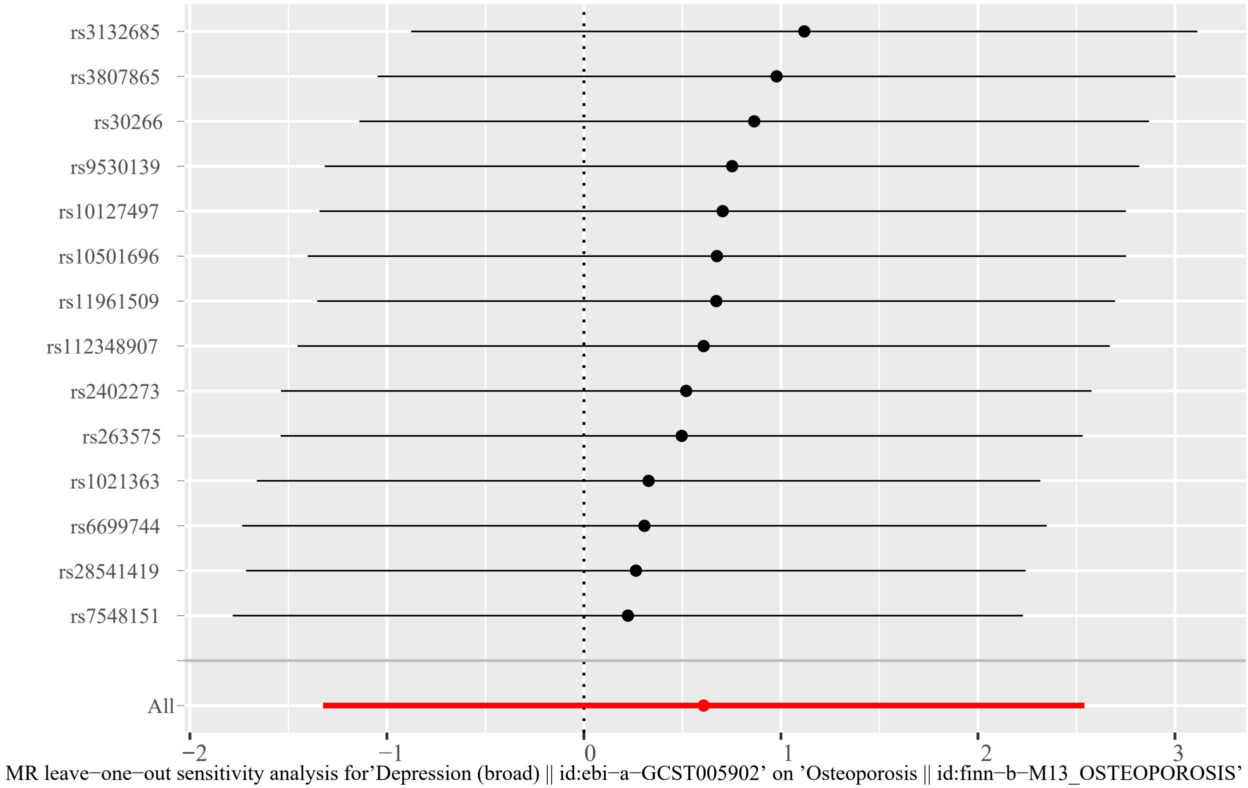

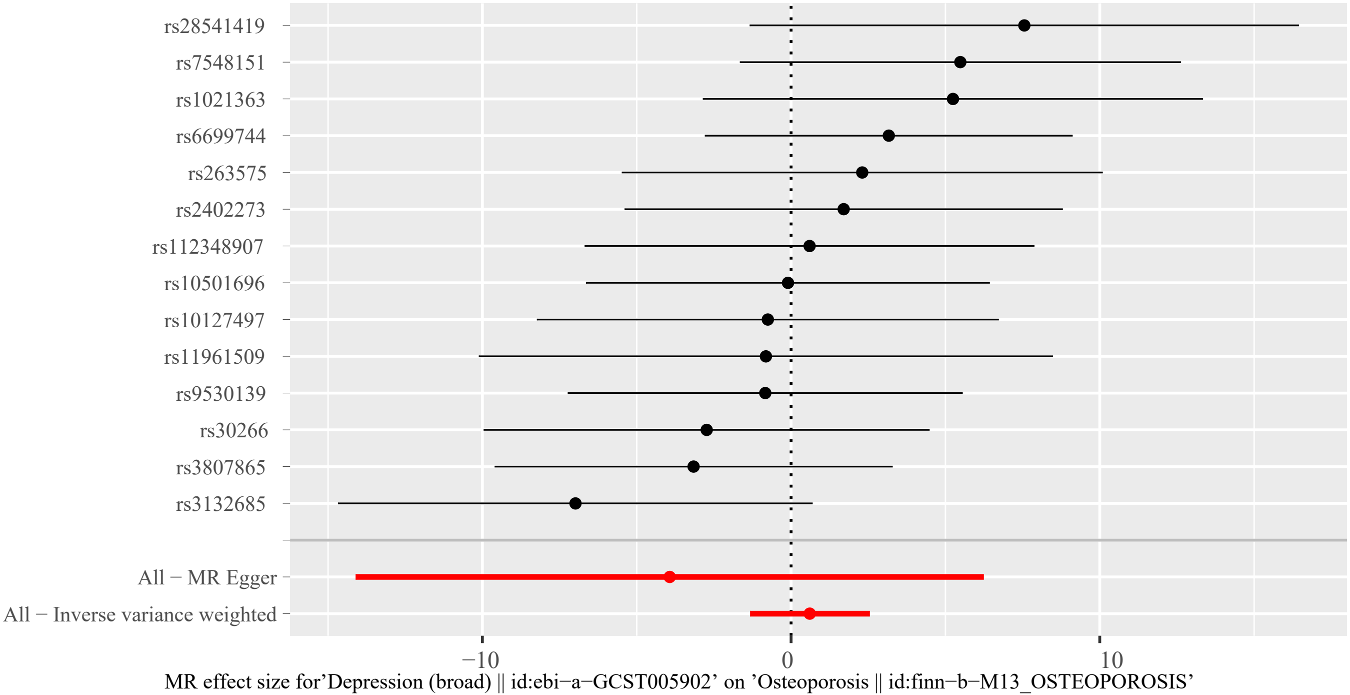
rs28541419
rs7548151
rs1021363
rs6699744
rs263575
rs2402273
rs112348907
rs10501696
rs10127497
rs11961509
rs9530139
rs30266
rs3807865
rs3132685
All − MR Egger
All − Inverse variance weighted
−10
0
10
MR effect size for'Depression (broad) || id:ebi−a−GCST005902' on 'Osteoporosis || id:finn−b−M13_OSTEOPOROSIS'

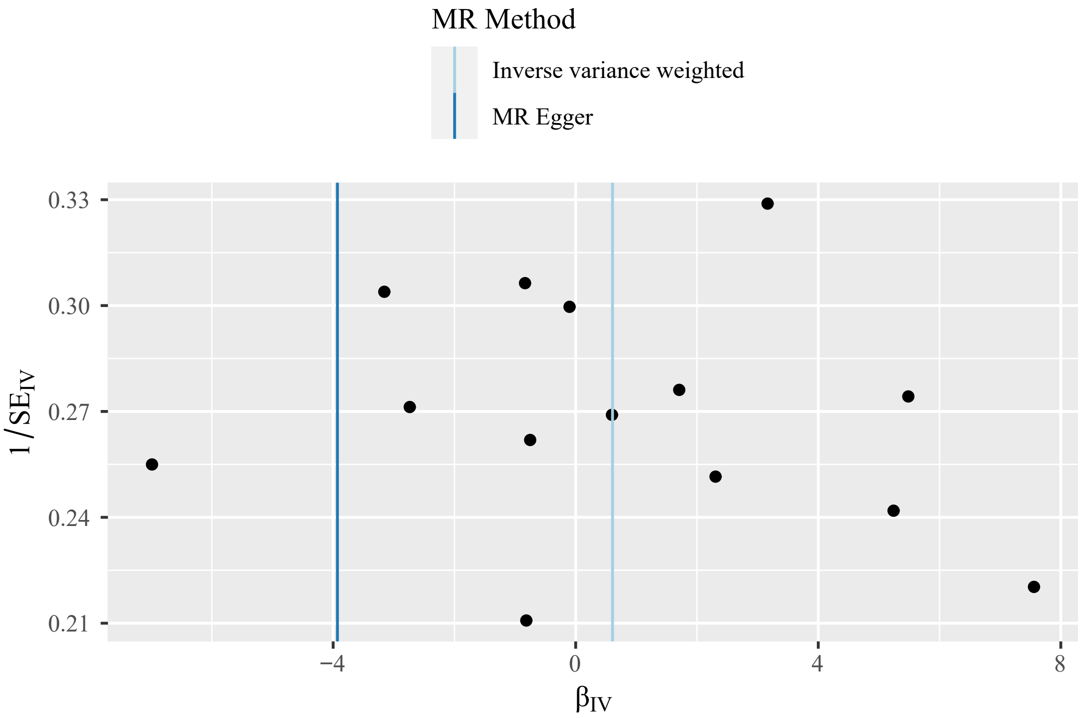

MR Method
Inverse variance weighted
MR Egger
0.33
0.30
0.27
0.24
0.21
1/SE_IV
−4
0
4
8
β_IV

Figure S2. Leave-one-out analysis, MR effect size and funnel plot for MDD on OPF.

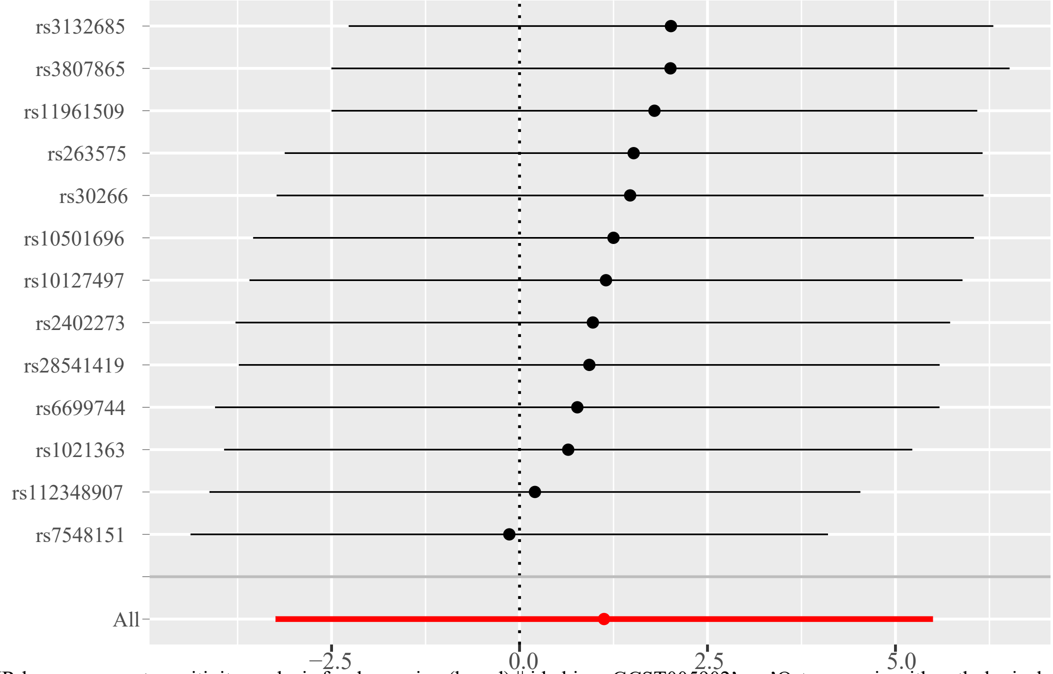

MR leave−one−out sensitivity analysis for depression (broad) || id:ebi−a−GCST005902' on 'Osteoporosis with pathological fracture (FG) || id:finn−b−OSTEOPOROS

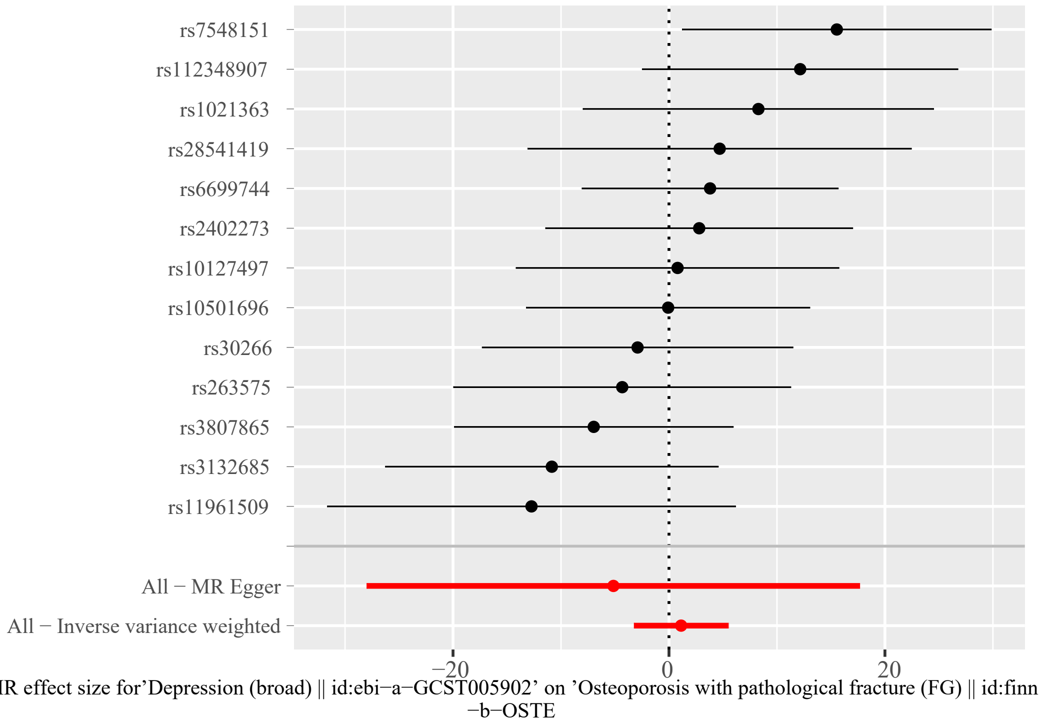

rs7548151
rs112348907
rs1021363
rs28541419
rs6699744
rs2402273
rs10127497
rs10501696
rs30266
rs263575
rs3807865
rs3132685
rs11961509
All − MR Egger
All − Inverse variance weighted
−20
0
20
MR effect size for'Depression (broad) || id:ebi−a−GCST005902' on 'Osteoporosis with pathological fracture (FG) || id:finn−b−OSTE

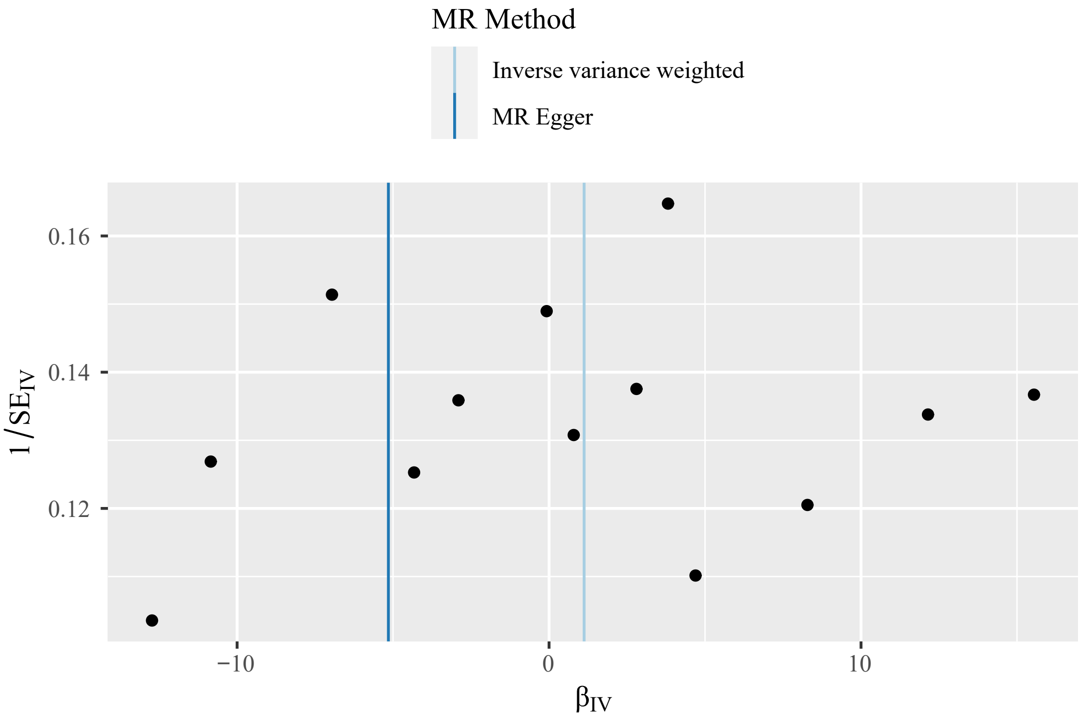

MR Method
Inverse variance weighted
MR Egger
0.16
0.14
0.12
$1/SE_{IV}$
−10
0
10
β_{IV}

Figure S3. Leave-one-out analysis, MR effect size and funnel plot for MDD on TB-BMD.

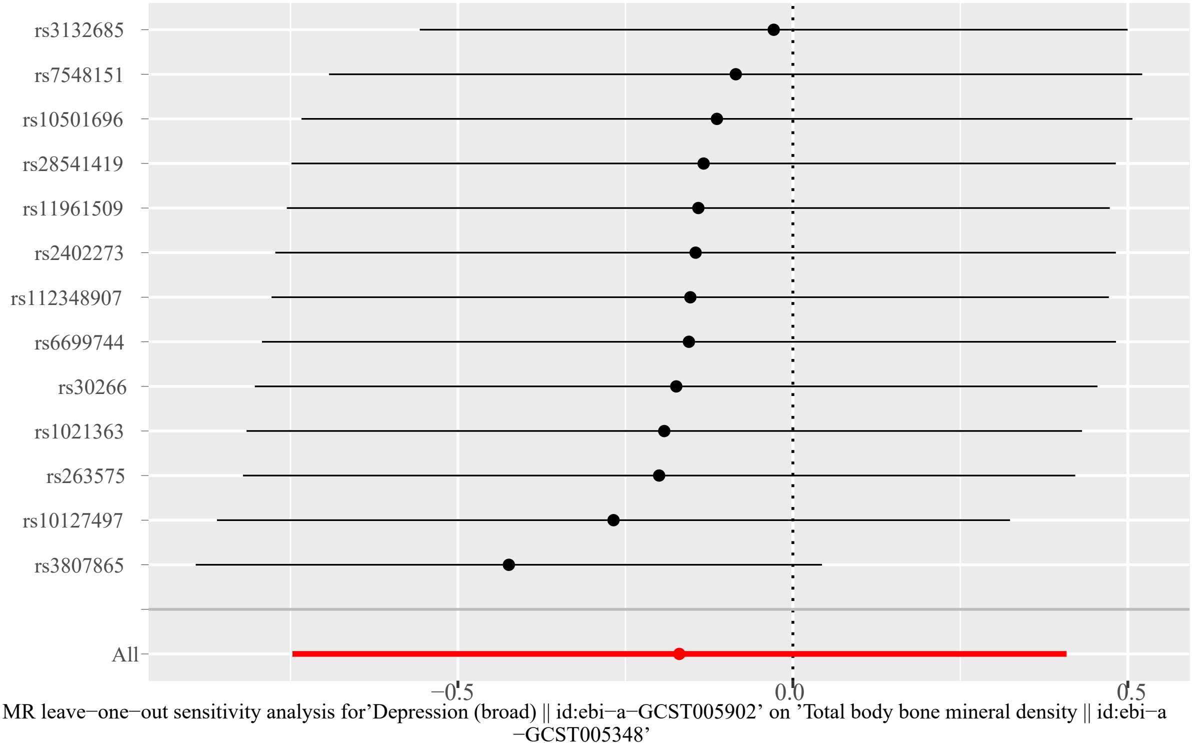

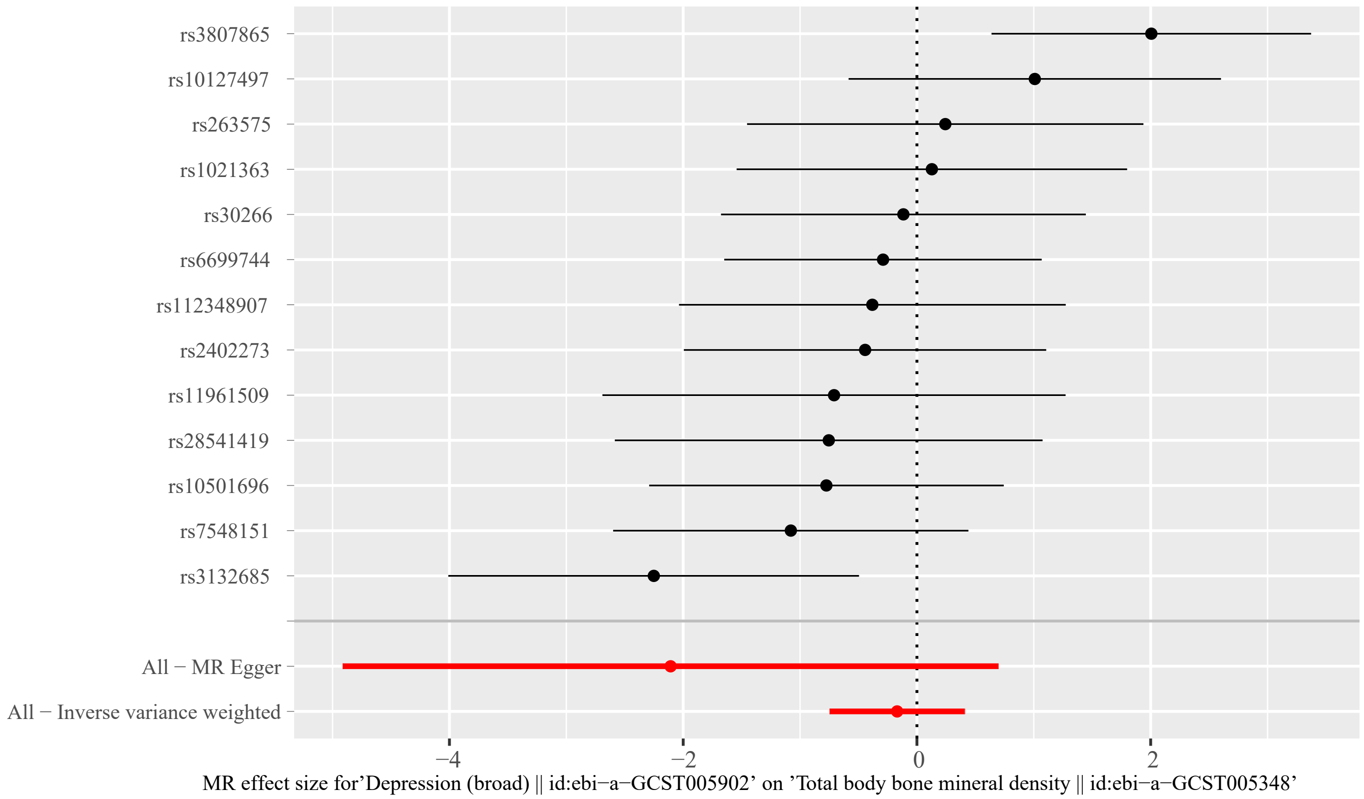
rs3807865
rs10127497
rs263575
rs1021363
rs30266
rs6699744
rs112348907
rs2402273
rs11961509
rs28541419
rs10501696
rs7548151
rs3132685
All − MR Egger
All − Inverse variance weighted
−4
−2
0
2
MR effect size for'Depression (broad) || id:ebi−a−GCST005902' on 'Total body bone mineral density || id:ebi−a−GCST005348'

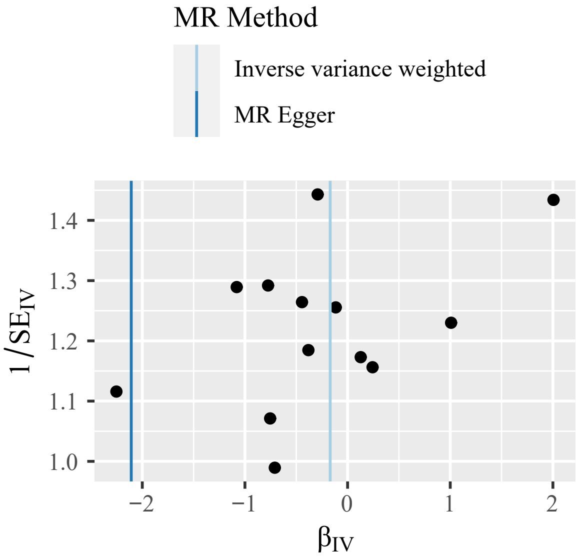

MR Method
Inverse variance weighted
MR Egger
1.4
1.3
1.2
1.1
1.0
1/SE_IV
−2
−1
0
1
2
β_IV

Figure S4. Leave-one-out analysis, MR effect size and funnel plot for MDD on FN-BMD.

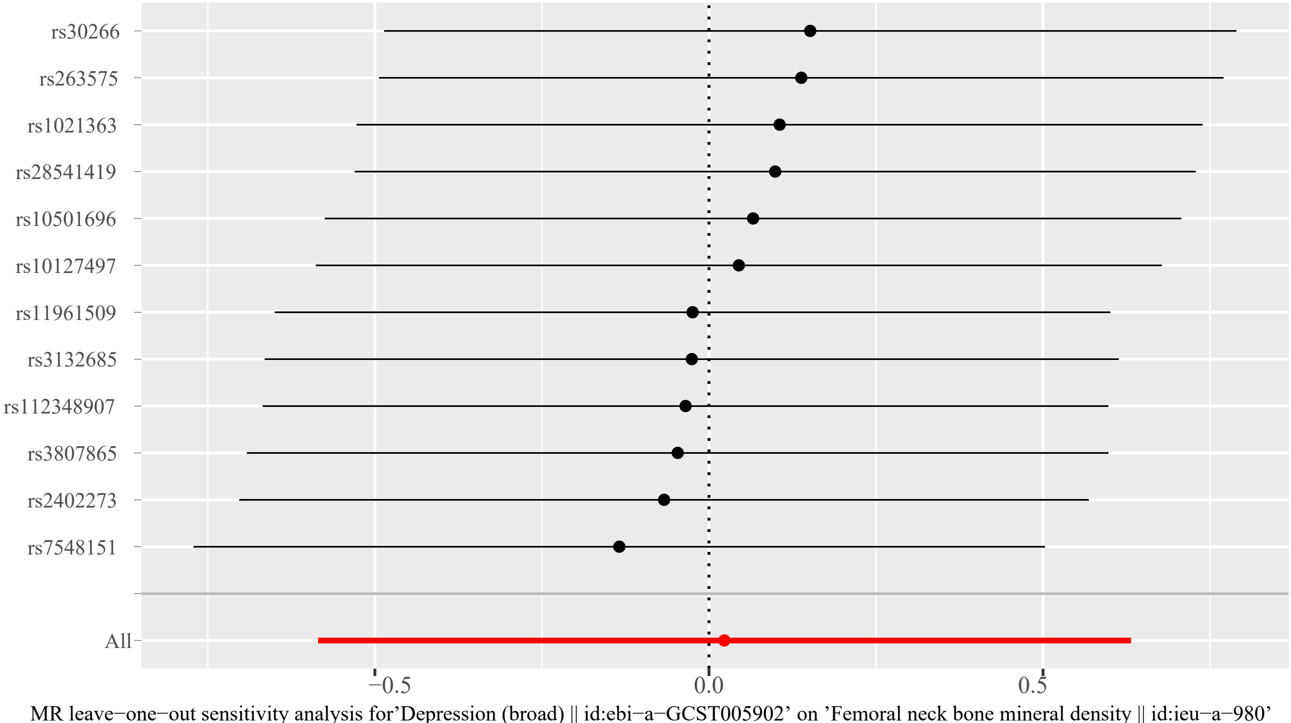

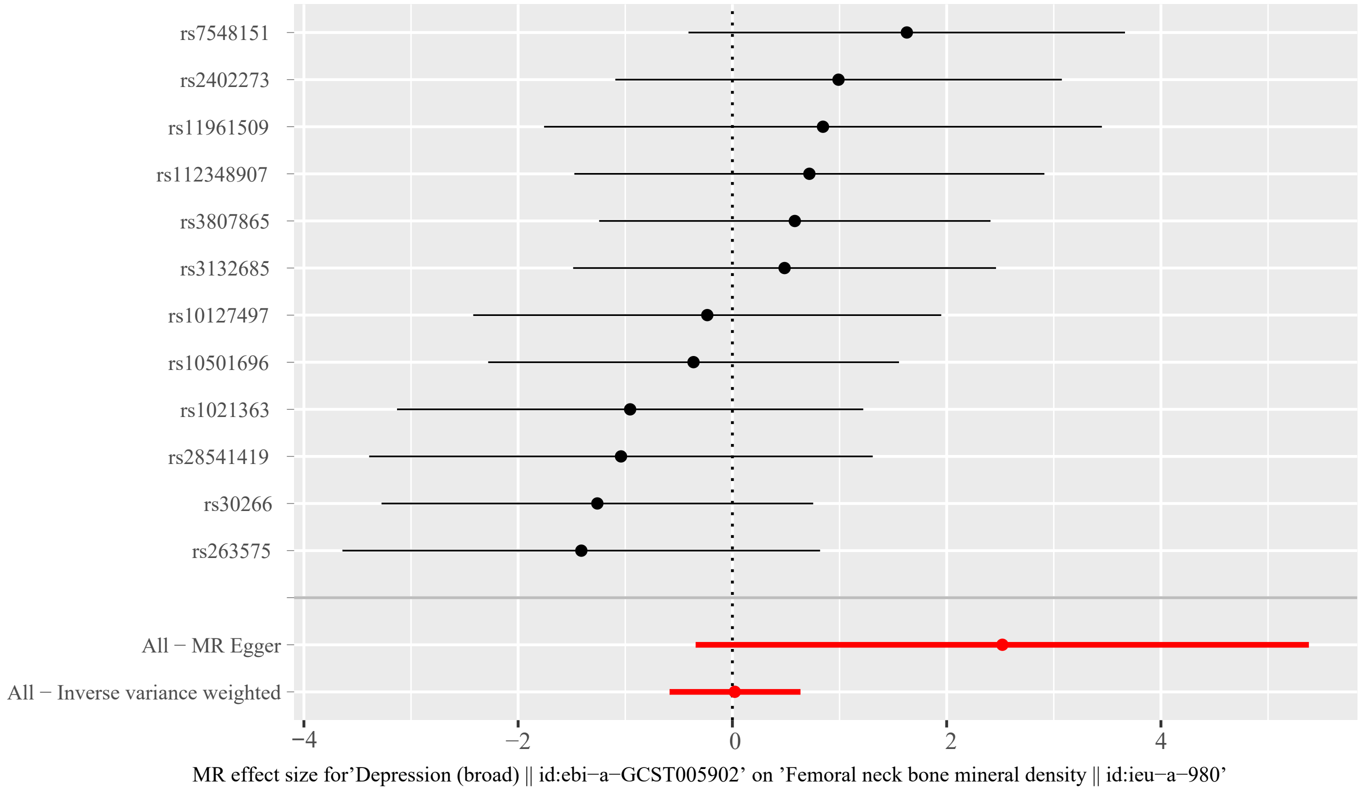
rs7548151
rs2402273
rs11961509
rs112348907
rs3807865
rs3132685
rs10127497
rs10501696
rs1021363
rs28541419
rs30266
rs263575
All − MR Egger
All − Inverse variance weighted
−4
−2
0
2
4
MR effect size for'Depression (broad) || id:ebi−a−GCST005902' on 'Femoral neck bone mineral density || id:ieu−a−980'

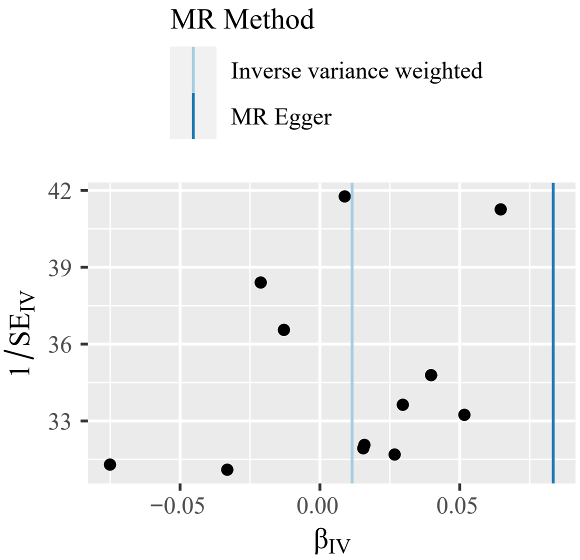

MR Method
Inverse variance weighted
MR Egger
42
39
36
33
1/SE$_{IV}$
−0.05
0.00
0.05
β$_{IV}$

Figure S5. Leave-one-out analysis, MR effect size and funnel plot for MDD on LS-BMD.

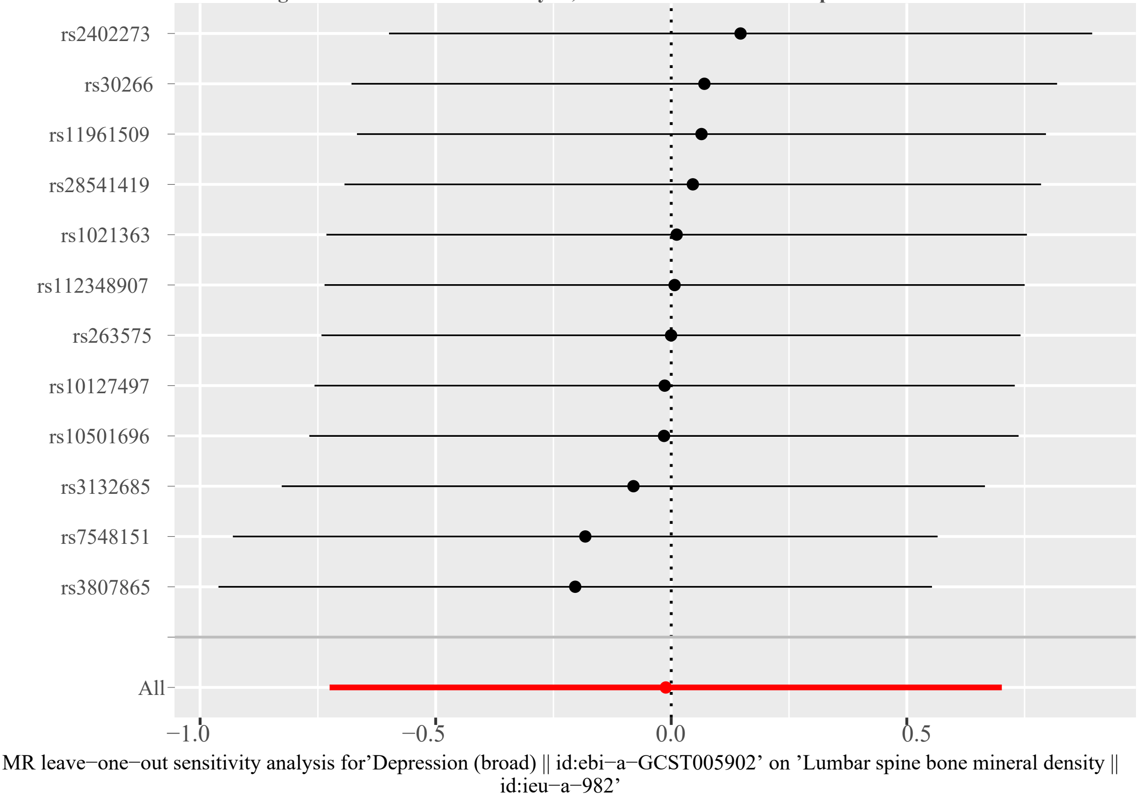

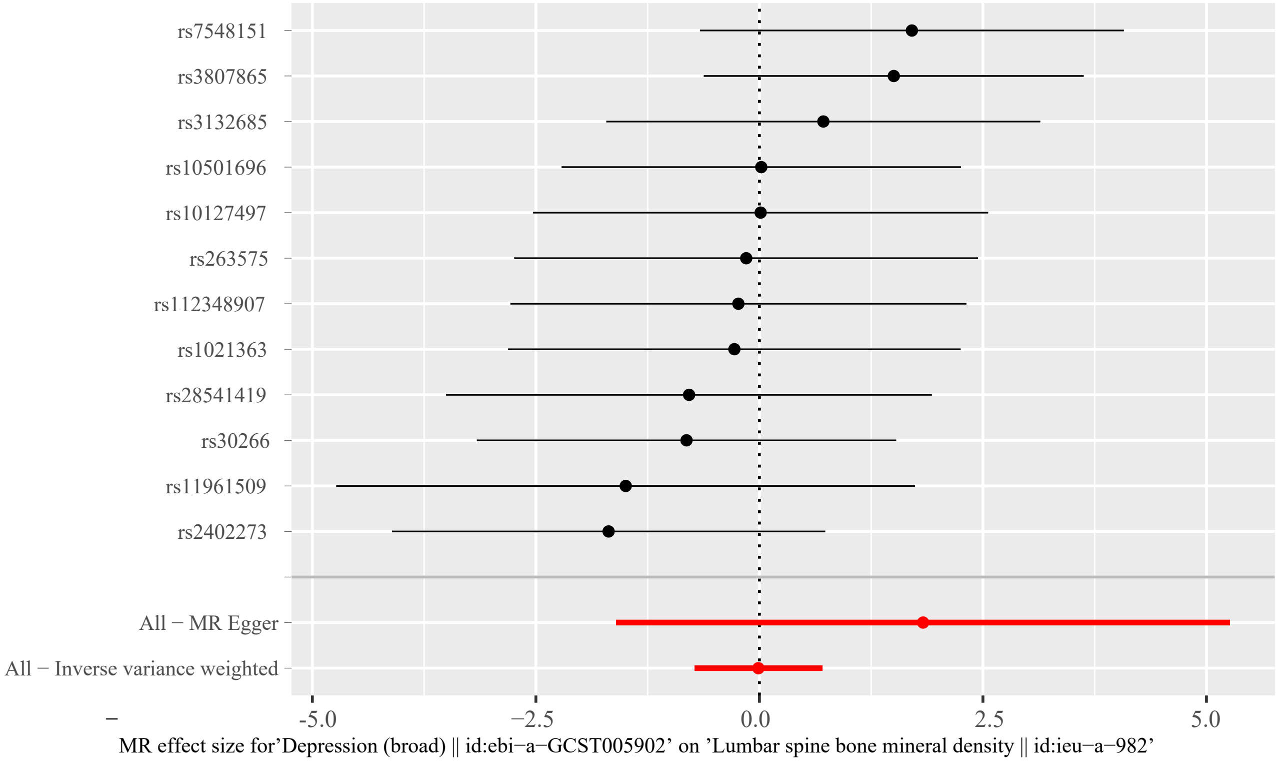
rs7548151
rs3807865
rs3132685
rs10501696
rs10127497
rs263575
rs112348907
rs1021363
rs28541419
rs30266
rs11961509
rs2402273
All − MR Egger
All − Inverse variance weighted
−5.0
−2.5
0.0
2.5
5.0
MR effect size for'Depression (broad) || id:ebi−a−GCST005902' on 'Lumbar spine bone mineral density || id:ieu−a−982'

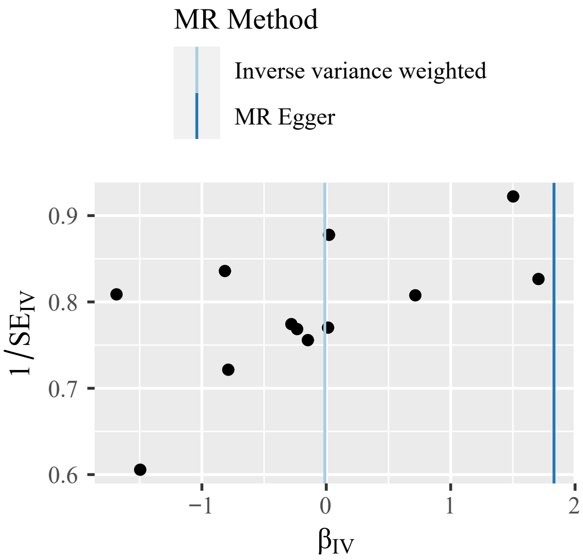
MR Method
Inverse variance weighted
MR Egger
0.9
0.8
0.7
0.6
1/SE_IV
−1
0
1
2
β_IV

Figure S6. Leave-one-out analysis, MR effect size and funnel plot for MDD on FA-BMD.

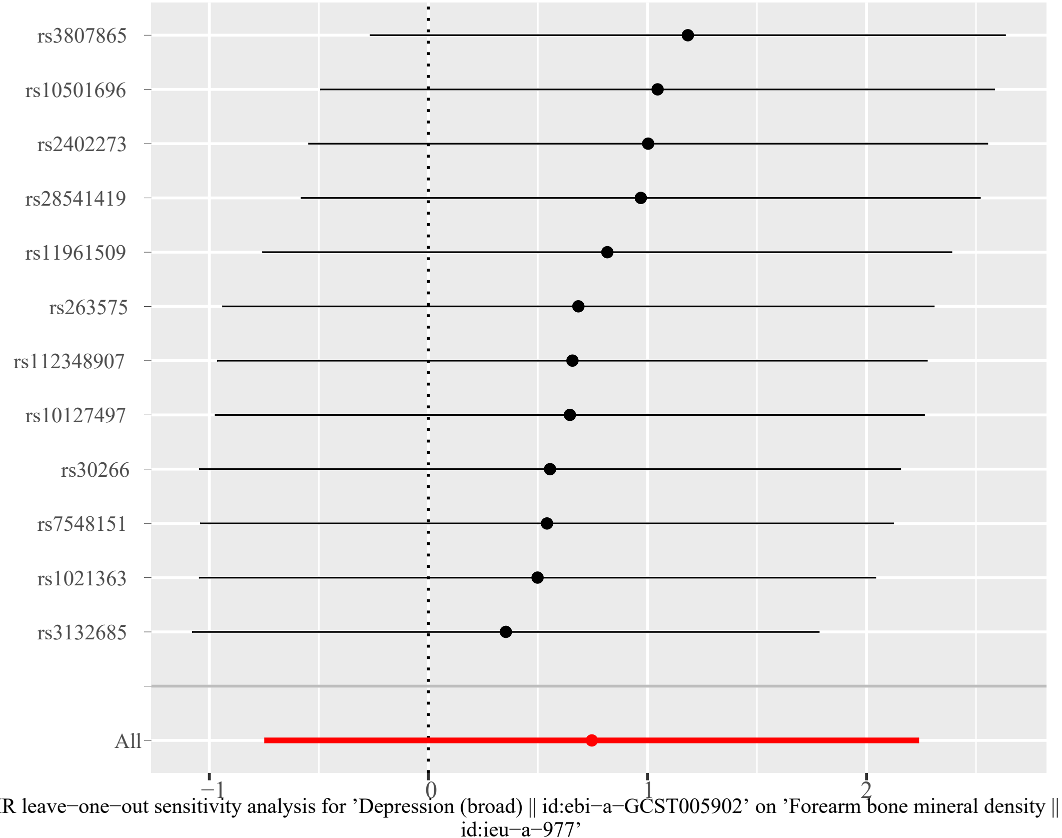

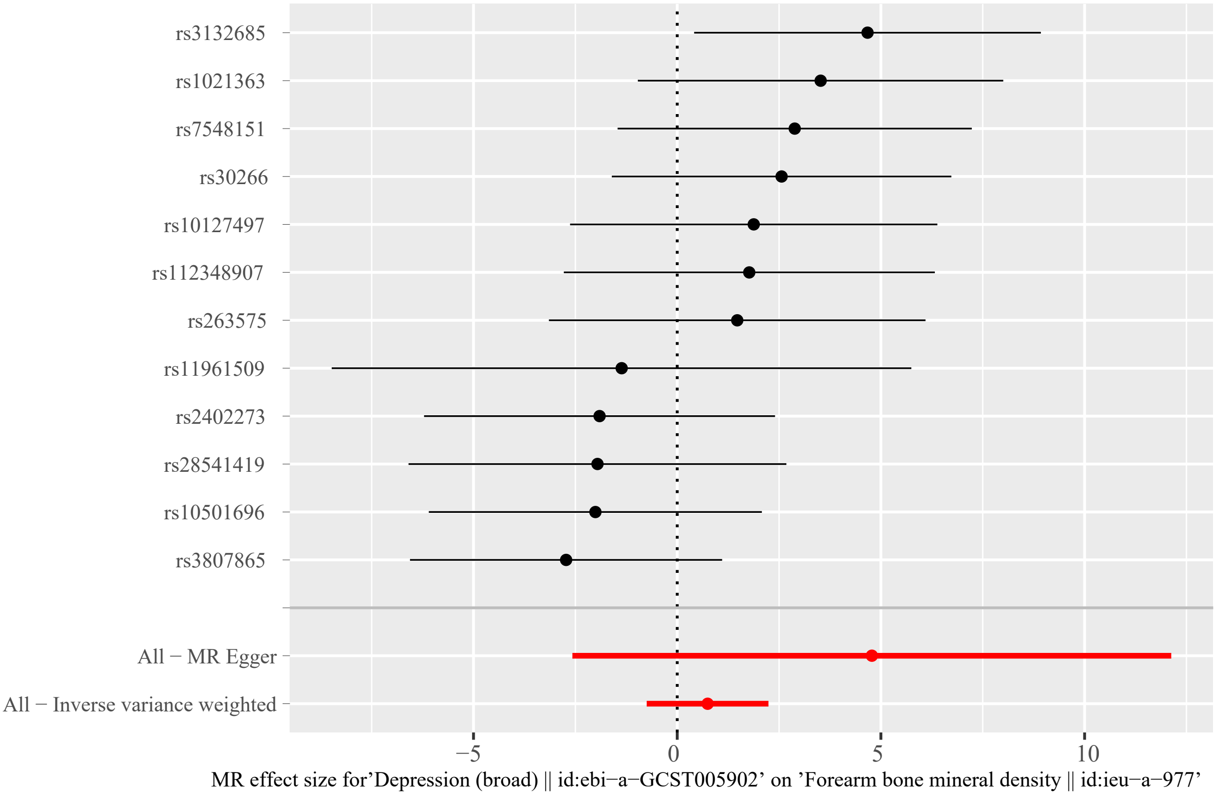
rs3132685
rs1021363
rs7548151
rs30266
rs10127497
rs112348907
rs263575
rs11961509
rs2402273
rs28541419
rs10501696
rs3807865
All − MR Egger
All − Inverse variance weighted
−5
0
5
10
MR effect size for'Depression (broad) || id:ebi−a−GCST005902' on 'Forearm bone mineral density || id:ieu−a−977'

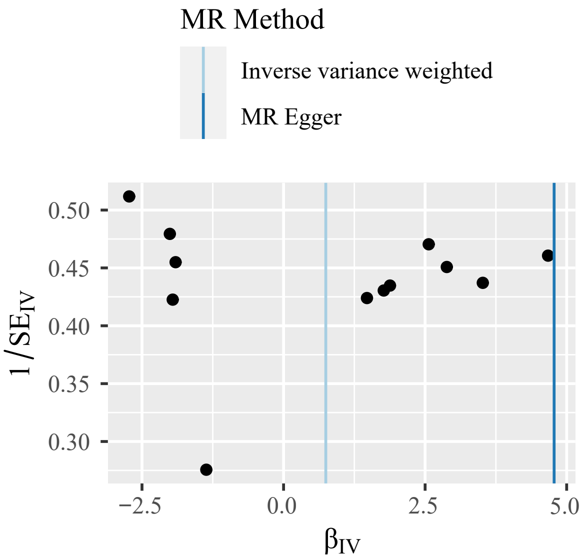
MR Method
Inverse variance weighted
MR Egger
0.50
0.45
0.40
0.35
0.30
$1/SE_{IV}$
−2.5
0.0
2.5
5.0
β_{IV}

Figure S7. Leave-one-out analysis, MR effect size and funnel plot for MDD on eBMD.

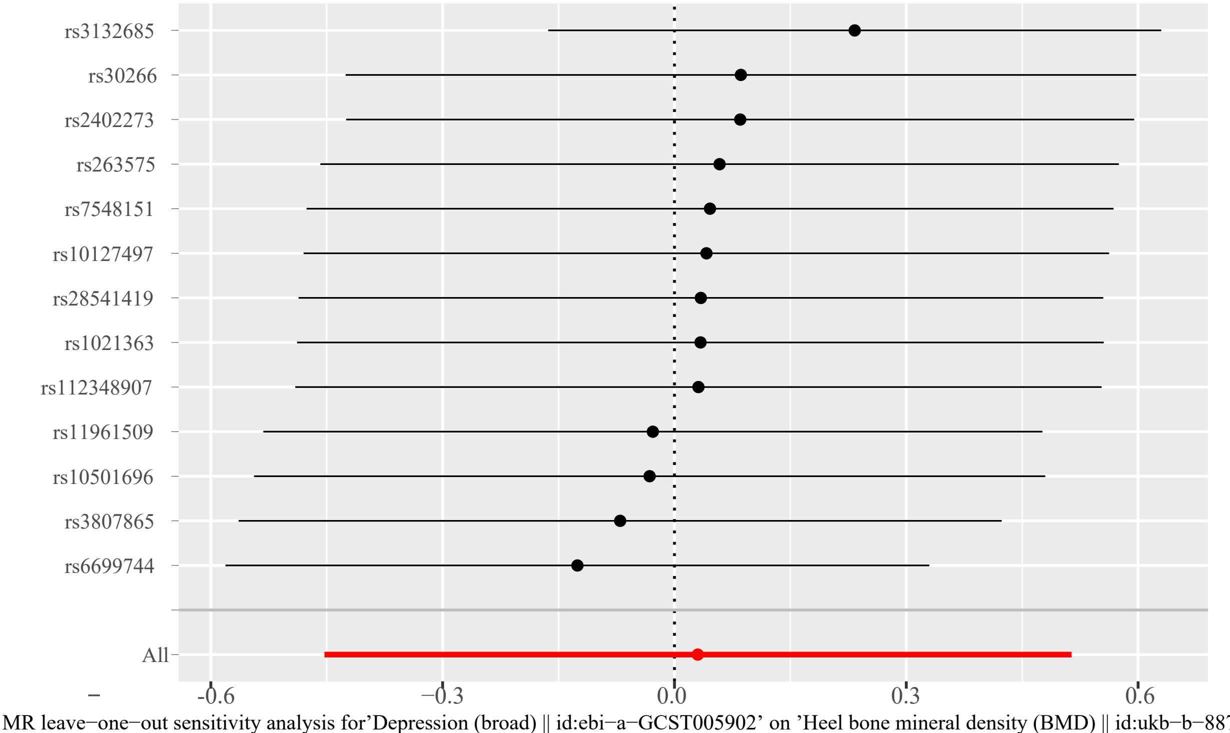

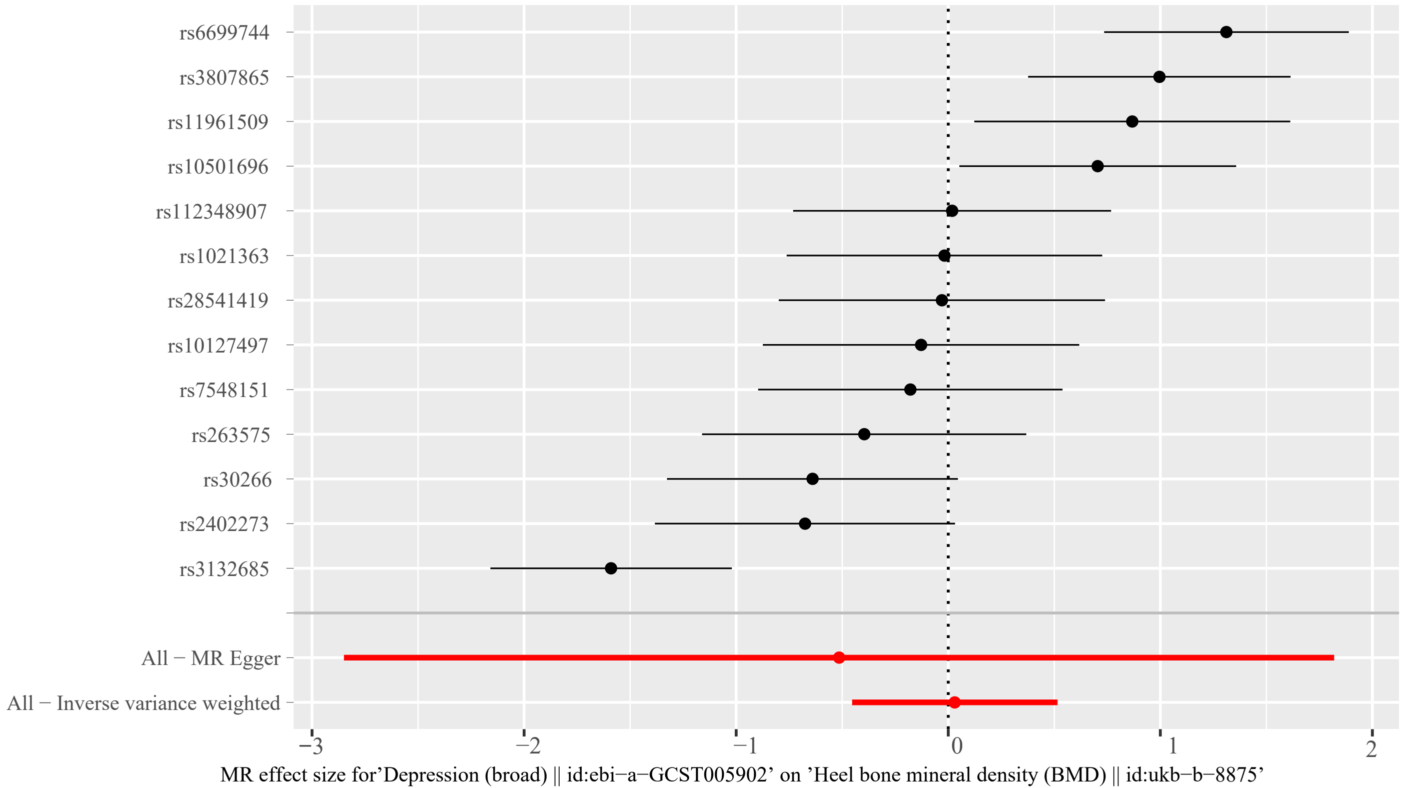

rs6699744
rs3807865
rs11961509
rs10501696
rs112348907
rs1021363
rs28541419
rs10127497
rs7548151
rs263575
rs30266
rs2402273
rs3132685
All − MR Egger
All − Inverse variance weighted
−3
−2
−1
0
1
2
MR effect size for'Depression (broad) || id:ebi−a−GCST005902' on 'Heel bone mineral density (BMD) || id:ukb−b−8875'

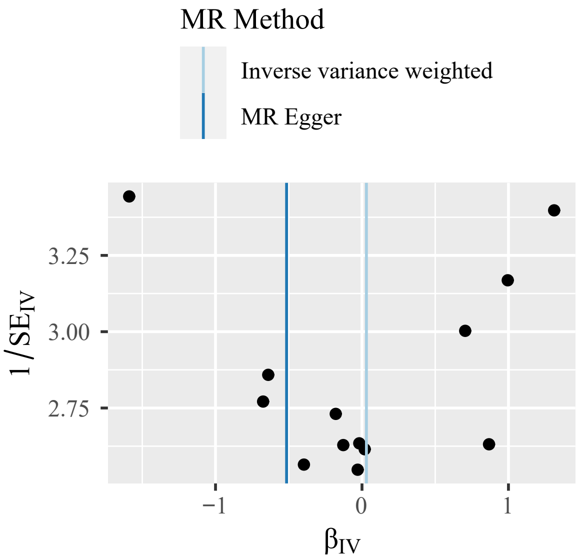
MR Method
Inverse variance weighted
MR Egger
3.25
3.00
2.75
−1
0
1
$1/SE_{IV}$
β_{IV}

Figure S8. Leave-one-out analysis, MR effect size and funnel plot for MDD on LF.

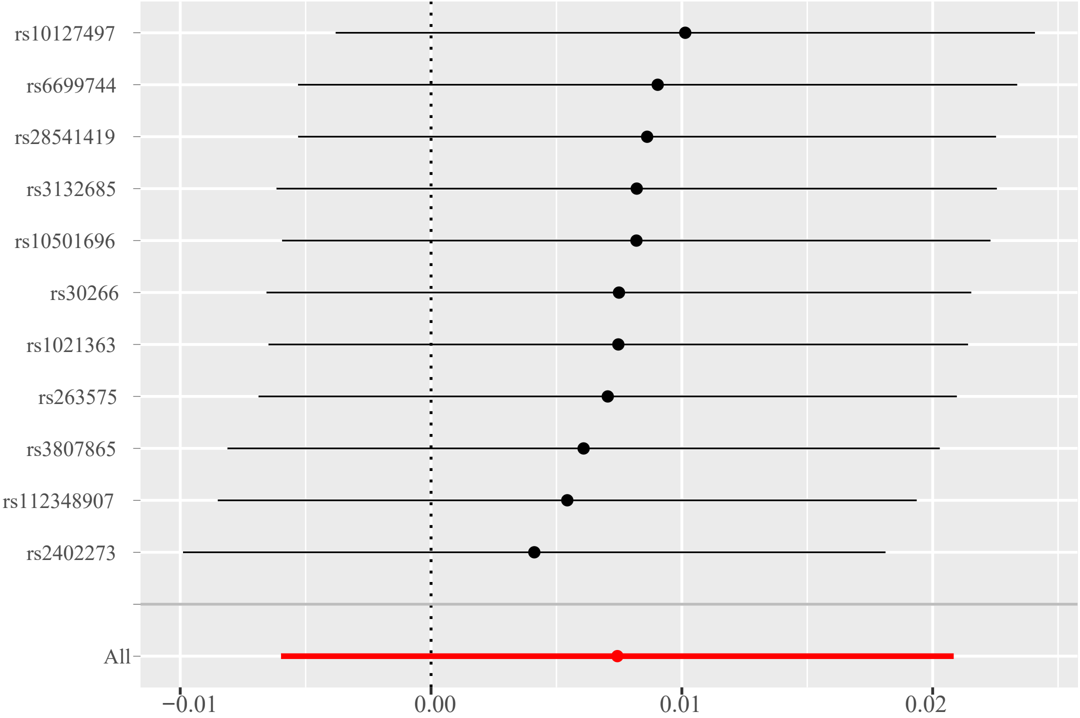

MR leave−one−out sensitivity analysis for'Depression (broad) || id:ebi−a−GCST005902' on 'Fractured bone site(s): Leg || id:ukb−b−3798'

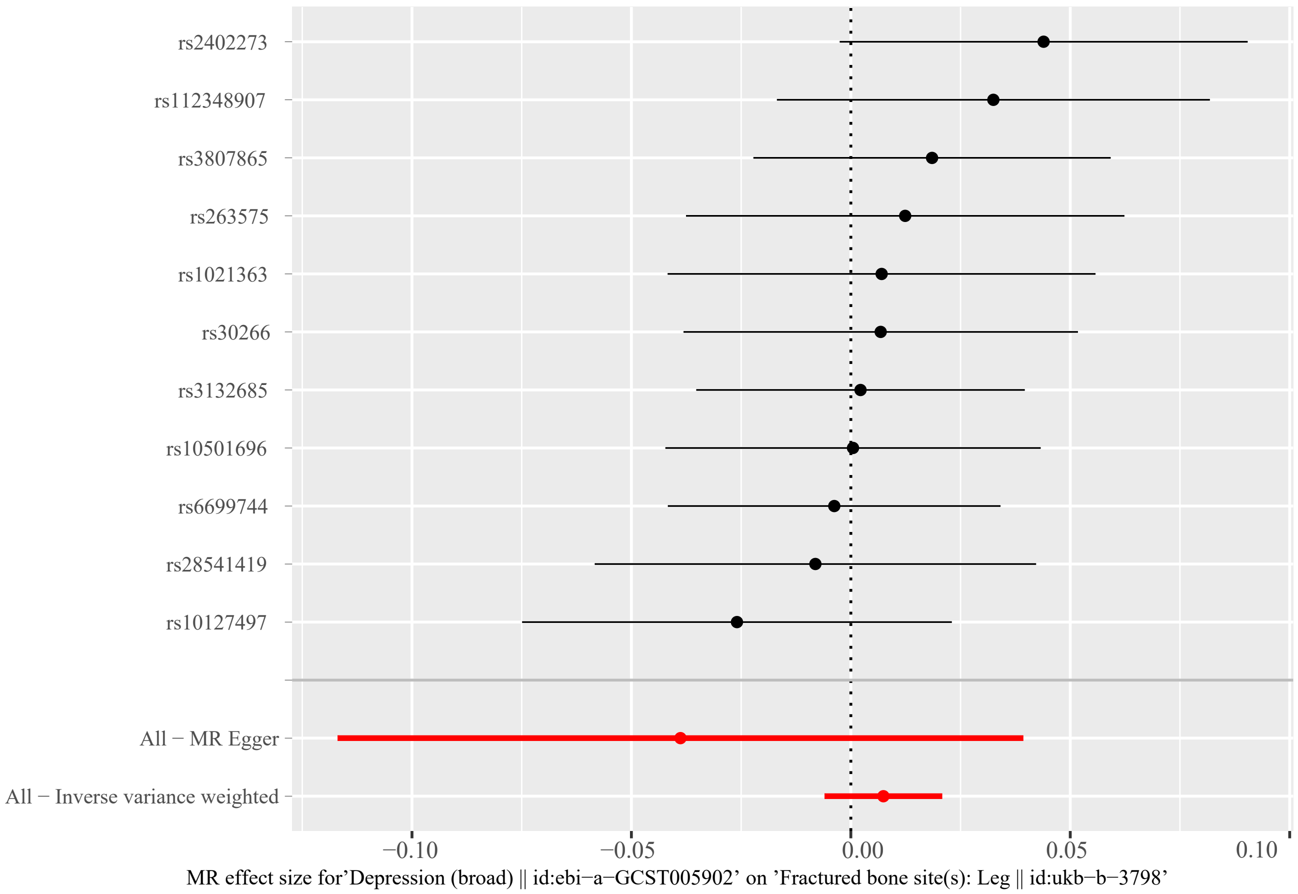
rs2402273
rs112348907
rs3807865
rs263575
rs1021363
rs30266
rs3132685
rs10501696
rs6699744
rs28541419
rs10127497
All − MR Egger
All − Inverse variance weighted
−0.10
−0.05
0.00
0.05
0.10
MR effect size for'Depression (broad) || id:ebi−a−GCST005902' on 'Fractured bone site(s): Leg || id:ukb−b−3798'

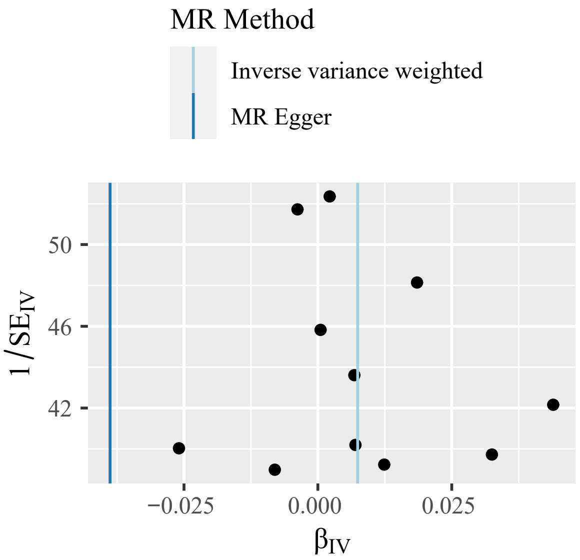

MR Method
Inverse variance weighted
MR Egger
50
46
42
$1/SE_{IV}$
−0.025
0.000
0.025
β_{IV}

Figure S9. Leave-one-out analysis, MR effect size and funnel plot for MDD on AF.

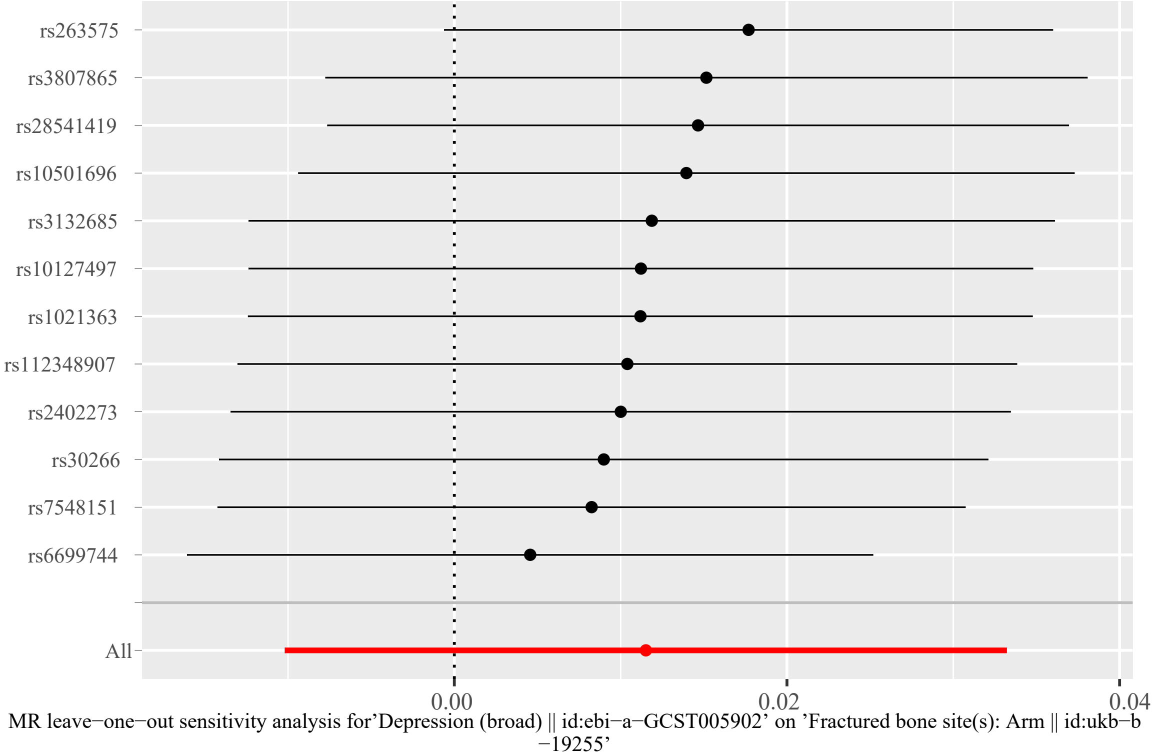

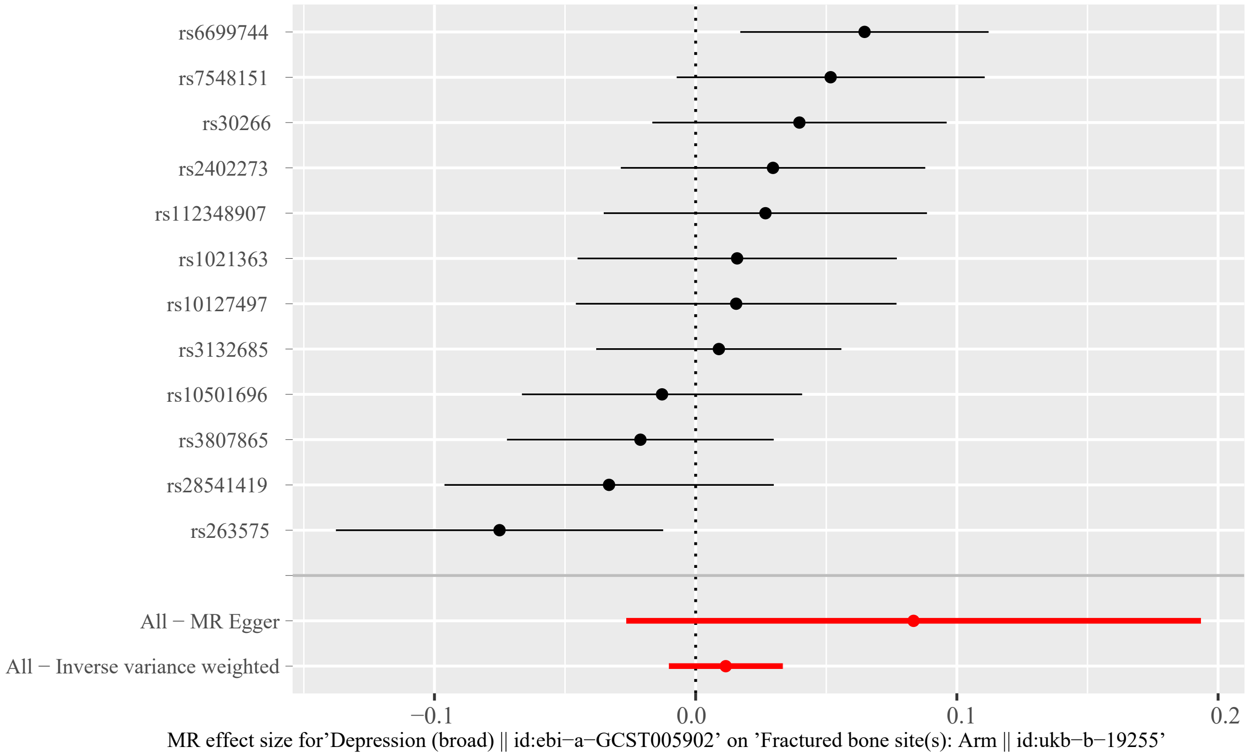
rs6699744
rs7548151
rs30266
rs2402273
rs112348907
rs1021363
rs10127497
rs3132685
rs10501696
rs3807865
rs28541419
rs263575
All − MR Egger
All − Inverse variance weighted
−0.1
0.0
0.1
0.2
MR effect size for'Depression (broad) || id:ebi−a−GCST005902' on 'Fractured bone site(s): Arm || id:ukb−b−19255'

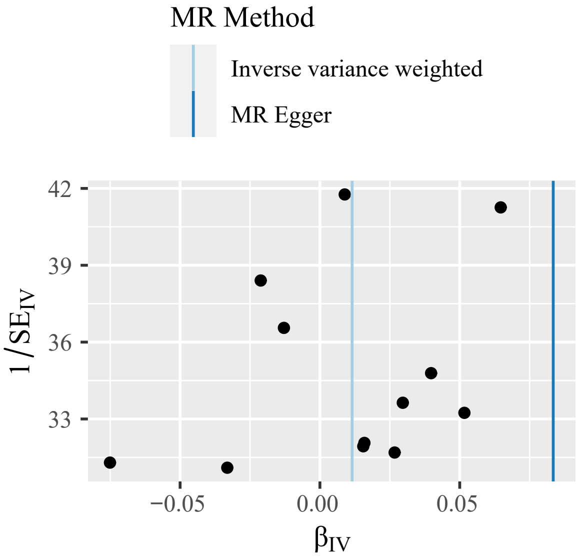
MR Method
Inverse variance weighted
MR Egger
42
39
36
33
$1/SE_{IV}$
−0.05
0.00
0.05
β_{IV}

Figure S10. Leave-one-out analysis, MR effect size and funnel plot for MDD on SF.

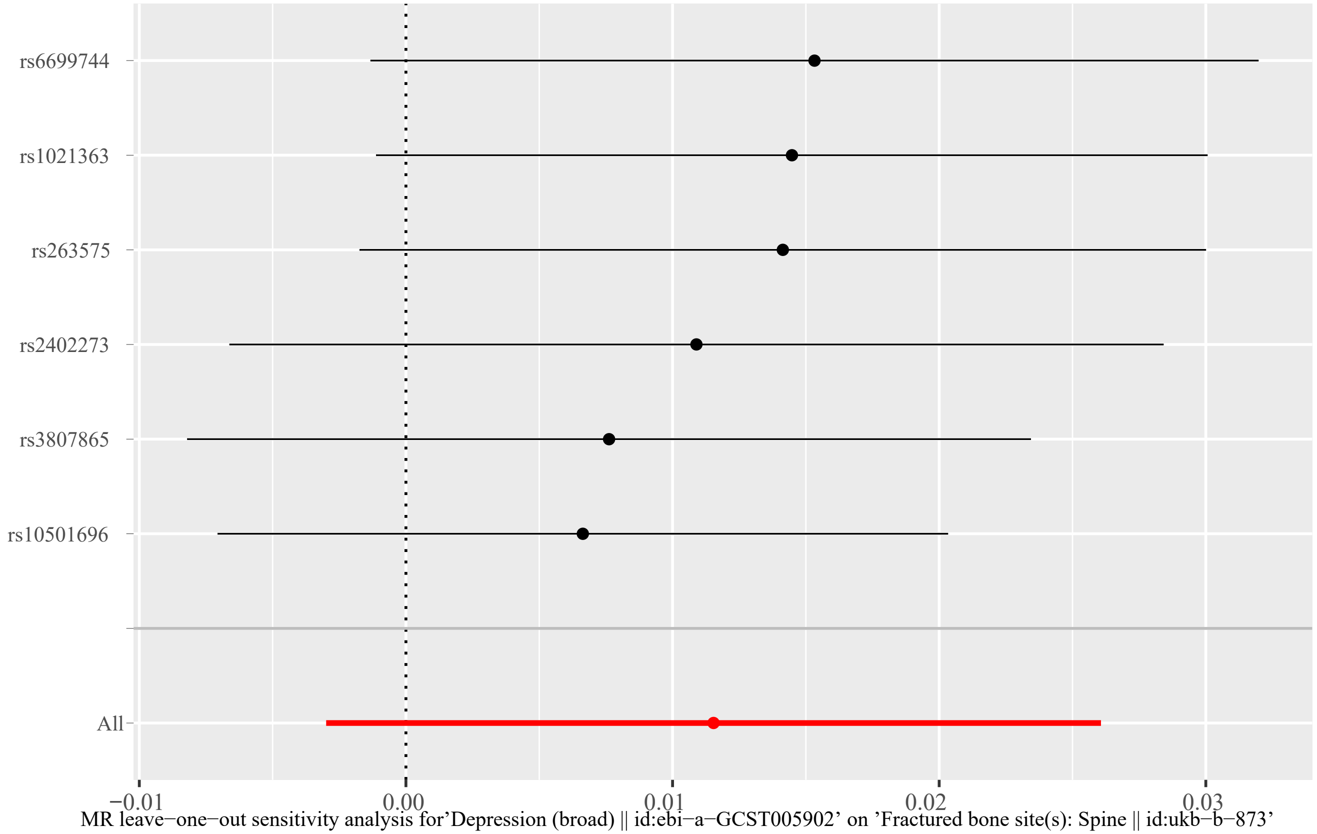

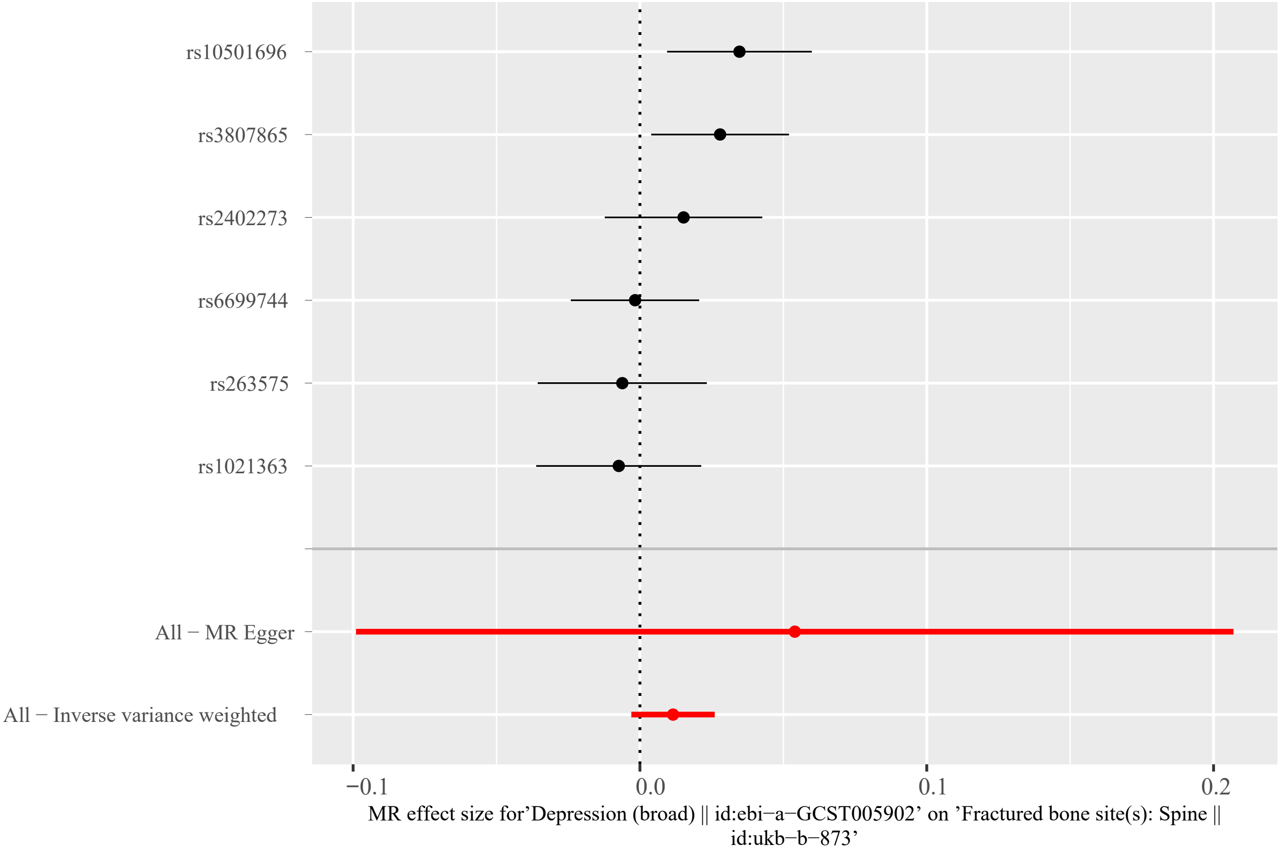
rs10501696
rs3807865
rs2402273
rs6699744
rs263575
rs1021363
All − MR Egger
All − Inverse variance weighted
−0.1
0.0
0.1
0.2
MR effect size for'Depression (broad) || id:ebi−a−GCST005902' on 'Fractured bone site(s): Spine || id:ukb−b−873'

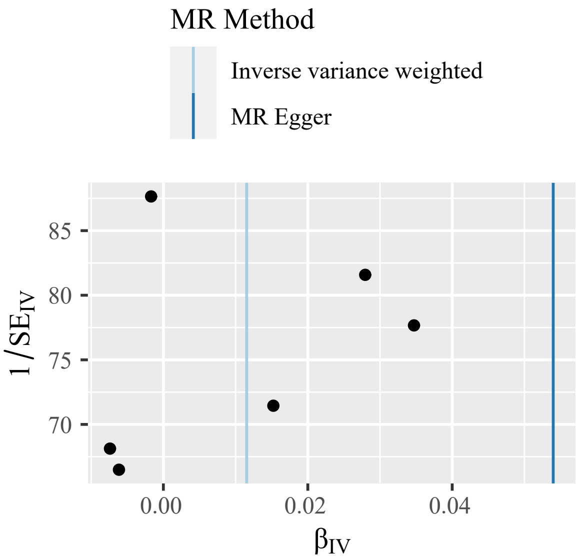

MR Method
Inverse variance weighted
MR Egger
85
80
75
70
0.00
0.02
0.04
1/SE_IV
β_IV

Figure S11. Leave-one-out analysis, MR effect size and funnel plot for MDD on HF.

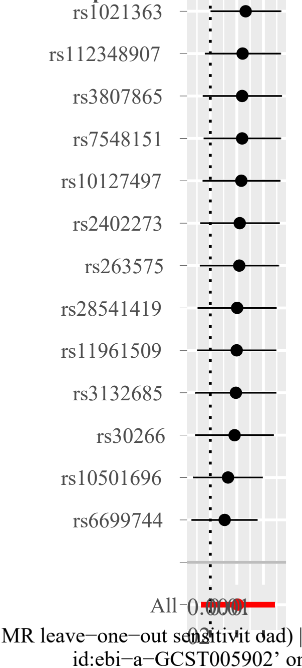

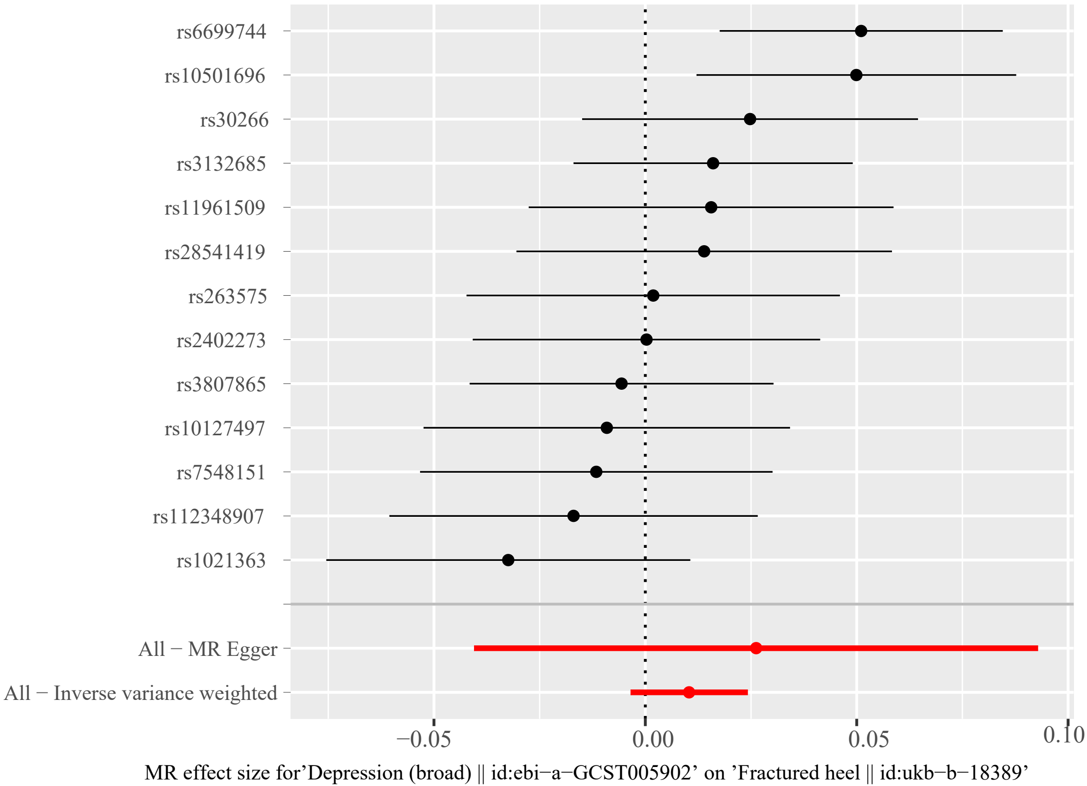
rs6699744
rs10501696
rs30266
rs3132685
rs11961509
rs28541419
rs263575
rs2402273
rs3807865
rs10127497
rs7548151
rs112348907
rs1021363
All − MR Egger
All − Inverse variance weighted
−0.05
0.00
0.05
0.10
MR effect size for'Depression (broad) || id:ebi−a−GCST005902' on 'Fractured heel || id:ukb−b−18389'

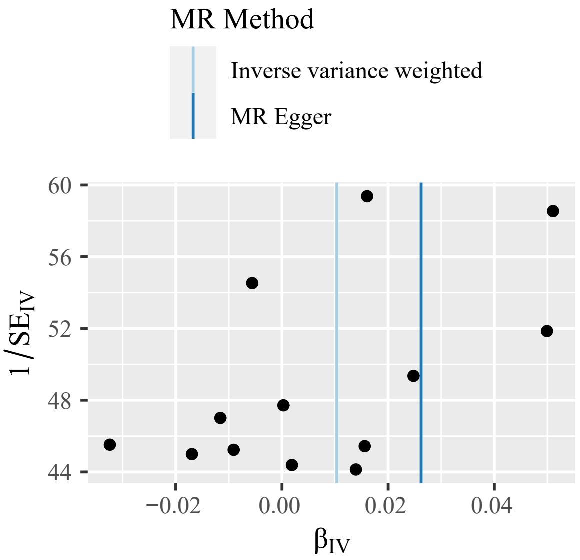

MR Method
Inverse variance weighted
MR Egger
60
56
52
48
44
1/SE_IV
−0.02
0.00
0.02
0.04
β_IV
