# Supplementary material for: Mendelian randomization analysis does not reveal a causal influence of mental diseases on osteoporosis
Source: Front Endocrinol (Lausanne). 2023 Apr 20;14:1125427. doi: 10.3389/fendo.2023.1125427 (PMC10157183; doi:10.3389/fendo.2023.1125427)
Supplement: Supplementary Material 1 — Instrumental variables SNPs. [file DataSheet_1.zip › Supplementary Material/Supplementary Material 5.pdf]

Figure S1 Leave-one-out analysis, MR effect size and funnel plot for AD on OP.

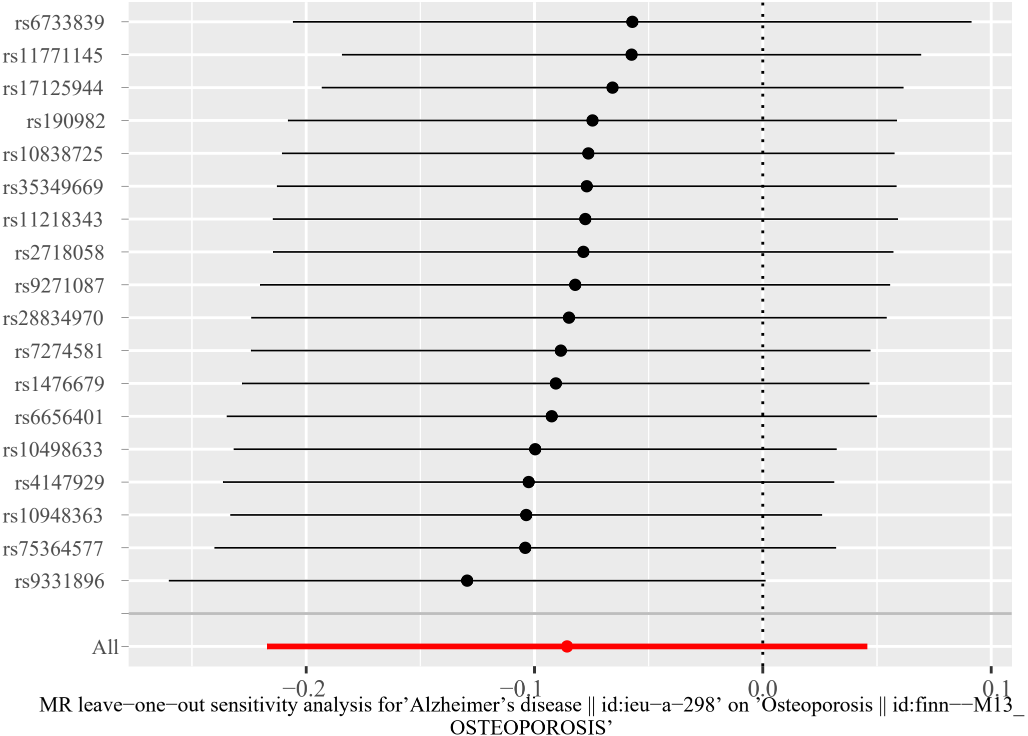

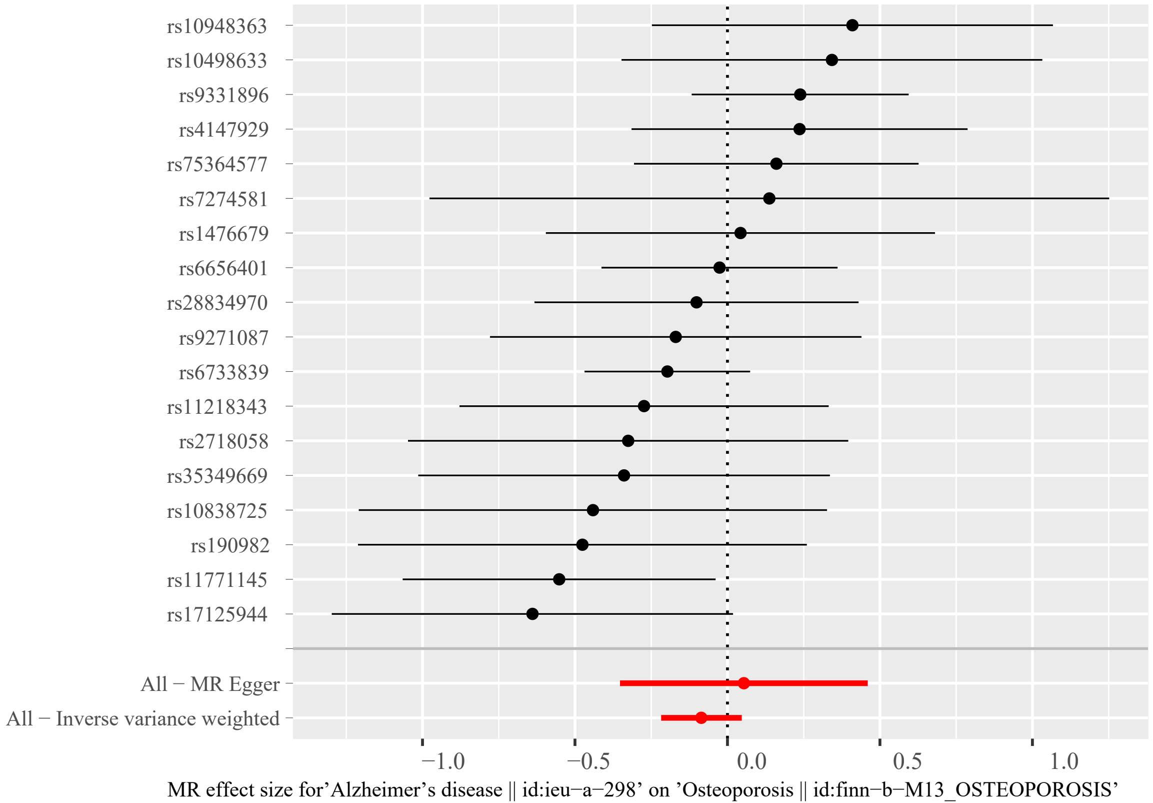
rs10948363
rs10498633
rs9331896
rs4147929
rs75364577
rs7274581
rs1476679
rs6656401
rs28834970
rs9271087
rs6733839
rs11218343
rs2718058
rs35349669
rs10838725
rs190982
rs11771145
rs17125944
All − MR Egger
All − Inverse variance weighted
−1.0
−0.5
0.0
0.5
1.0
MR effect size for'Alzheimer's disease || id:ieu−a−298' on 'Osteoporosis || id:finn−b−M13_OSTEOPOROSIS'

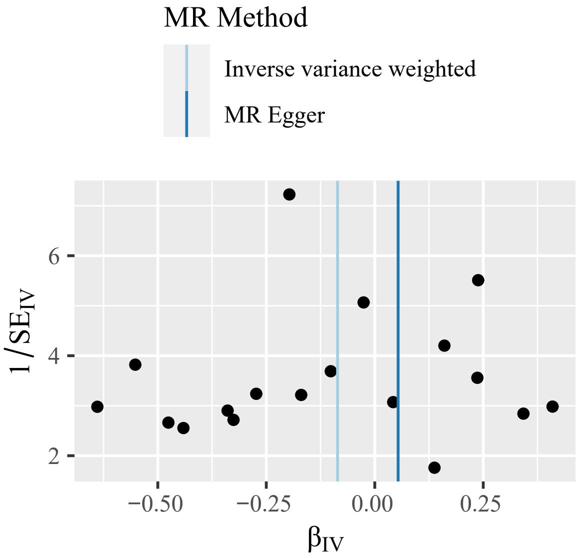

MR Method
Inverse variance weighted
MR Egger
6
4
2
1/SE_IV
−0.50
−0.25
0.00
0.25
β_IV

Figure S2 Leave-one-out analysis, MR effect size and funnel plot for AD on OPF.

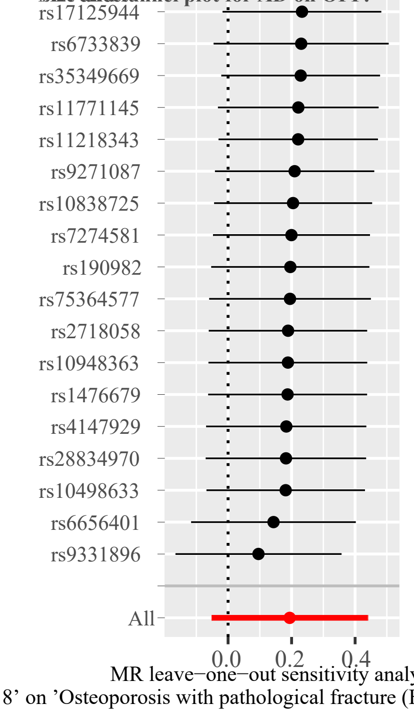

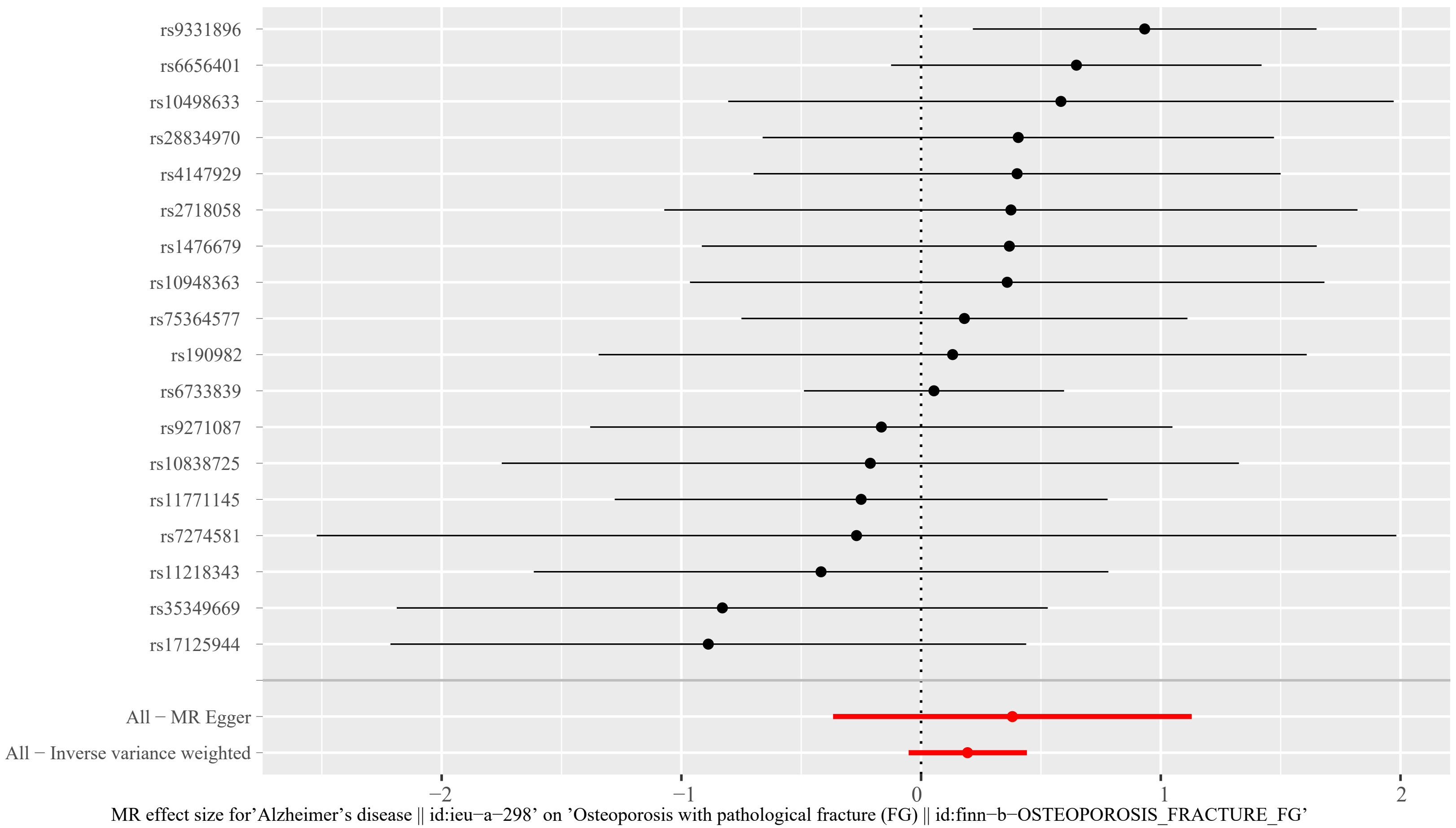
rs9331896
rs6656401
rs10498633
rs28834970
rs4147929
rs2718058
rs1476679
rs10948363
rs75364577
rs190982
rs6733839
rs9271087
rs10838725
rs11771145
rs7274581
rs11218343
rs35349669
rs17125944
All − MR Egger
All − Inverse variance weighted
−2
−1
0
1
2
MR effect size for'Alzheimer's disease || id:ieu−a−298' on 'Osteoporosis with pathological fracture (FG) || id:finn−b−OSTEOPOROSIS_FRACTURE_FG'

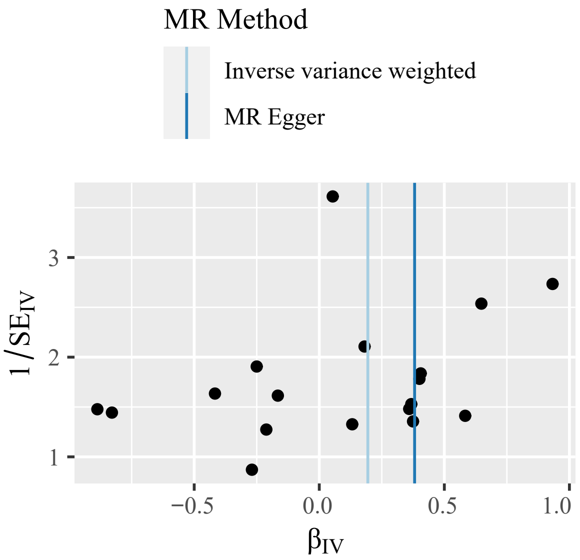
MR Method
Inverse variance weighted
MR Egger
1/SE_IV
3
2
1
−0.5
0.0
0.5
1.0
β_IV

Figure S3. Leave-one-out analysis, MR effect size and funnel plot for AD on TB-BMD.

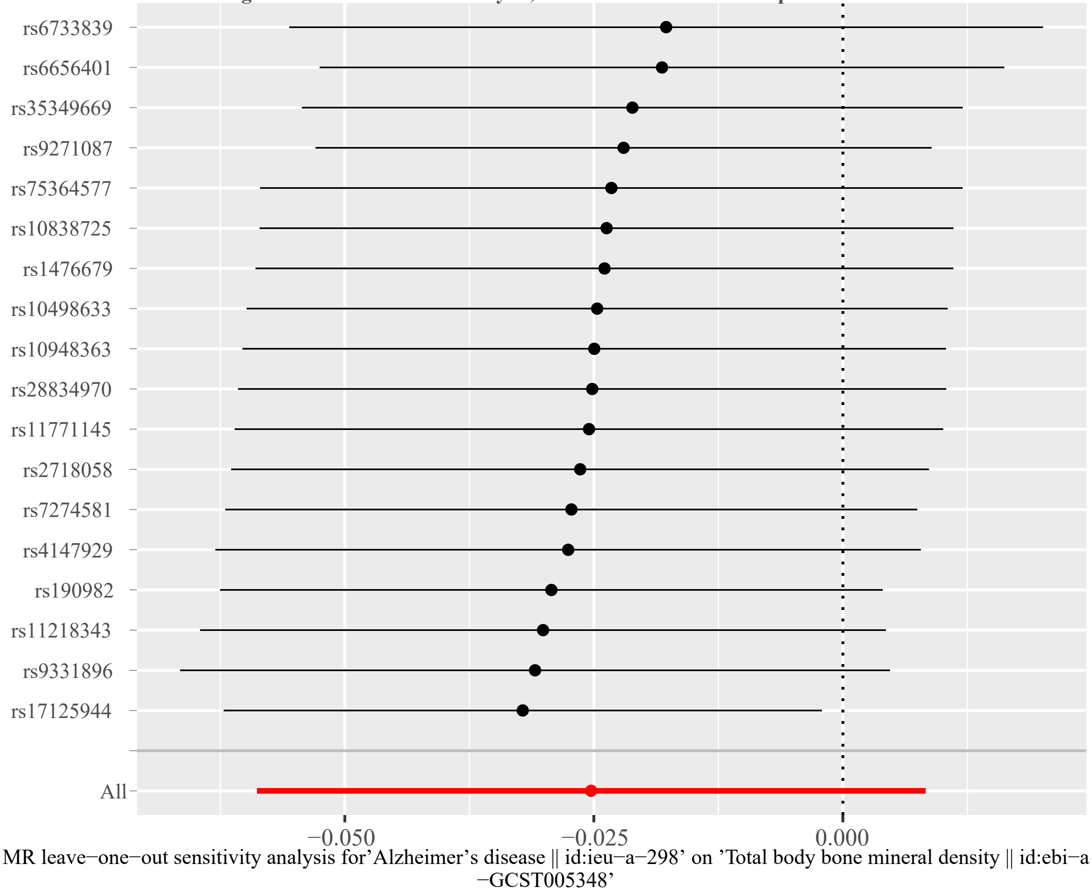

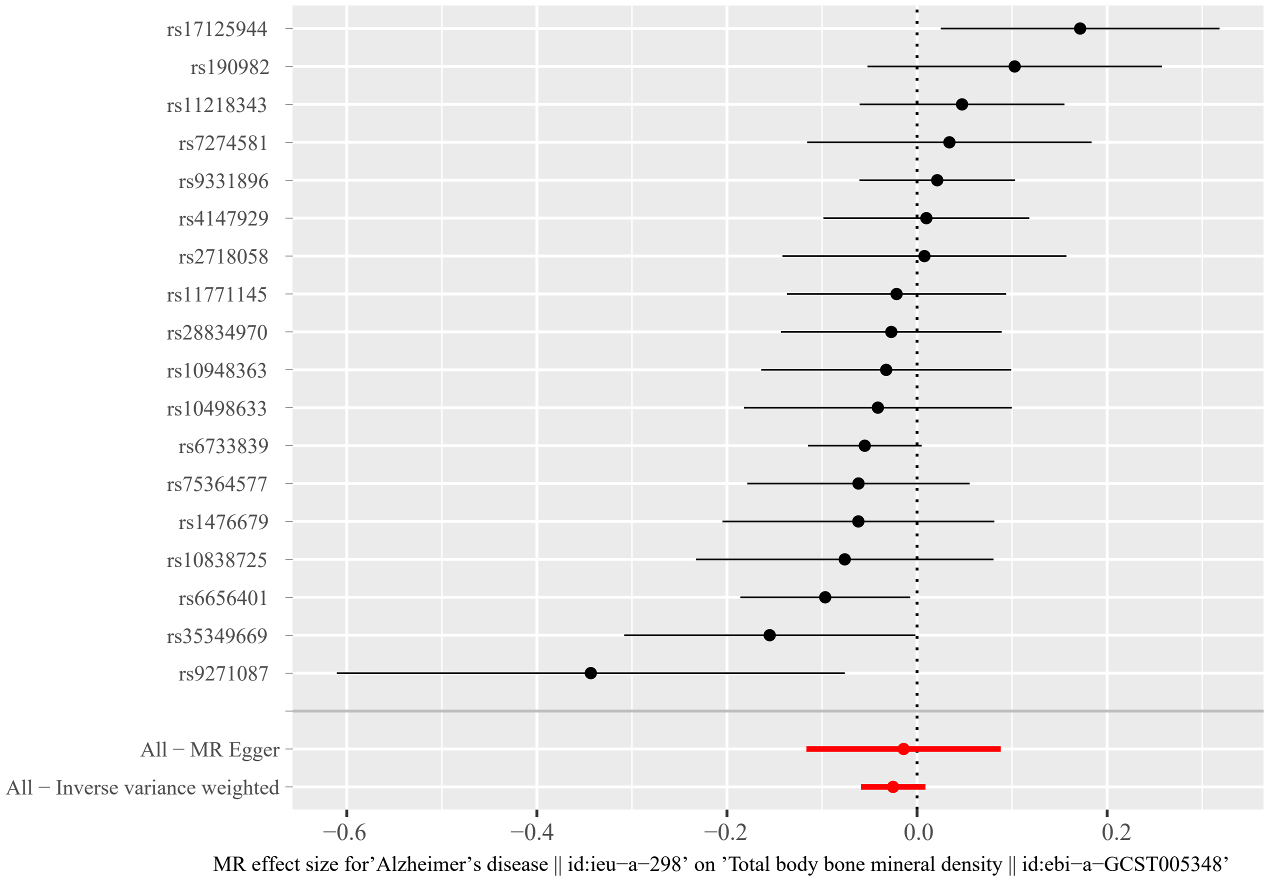
rs17125944
rs190982
rs11218343
rs7274581
rs9331896
rs4147929
rs2718058
rs11771145
rs28834970
rs10948363
rs10498633
rs6733839
rs75364577
rs1476679
rs10838725
rs6656401
rs35349669
rs9271087
All − MR Egger
All − Inverse variance weighted
−0.6
−0.4
−0.2
0.0
0.2
MR effect size for'Alzheimer's disease || id:ieu−a−298' on 'Total body bone mineral density || id:ebi−a−GCST005348'

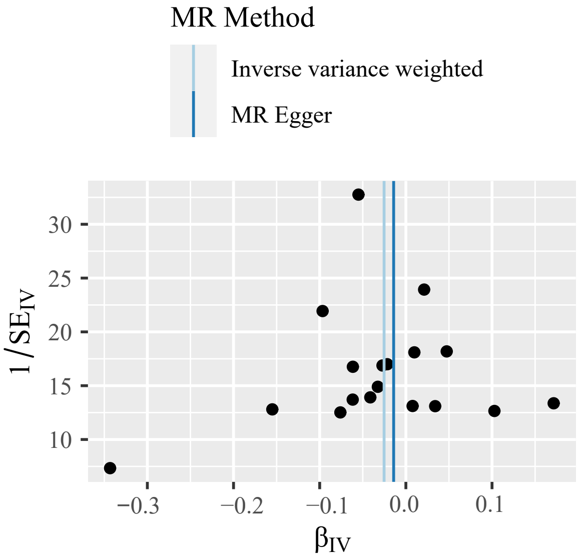

MR Method
Inverse variance weighted
MR Egger
30
25
20
15
10
1/SE$_{IV}$
−0.3
−0.2
−0.1
0.0
0.1
β$_{IV}$

Figure S4. Leave-one-out analysis, MR effect size and funnel plot for AD on FN-BMD.

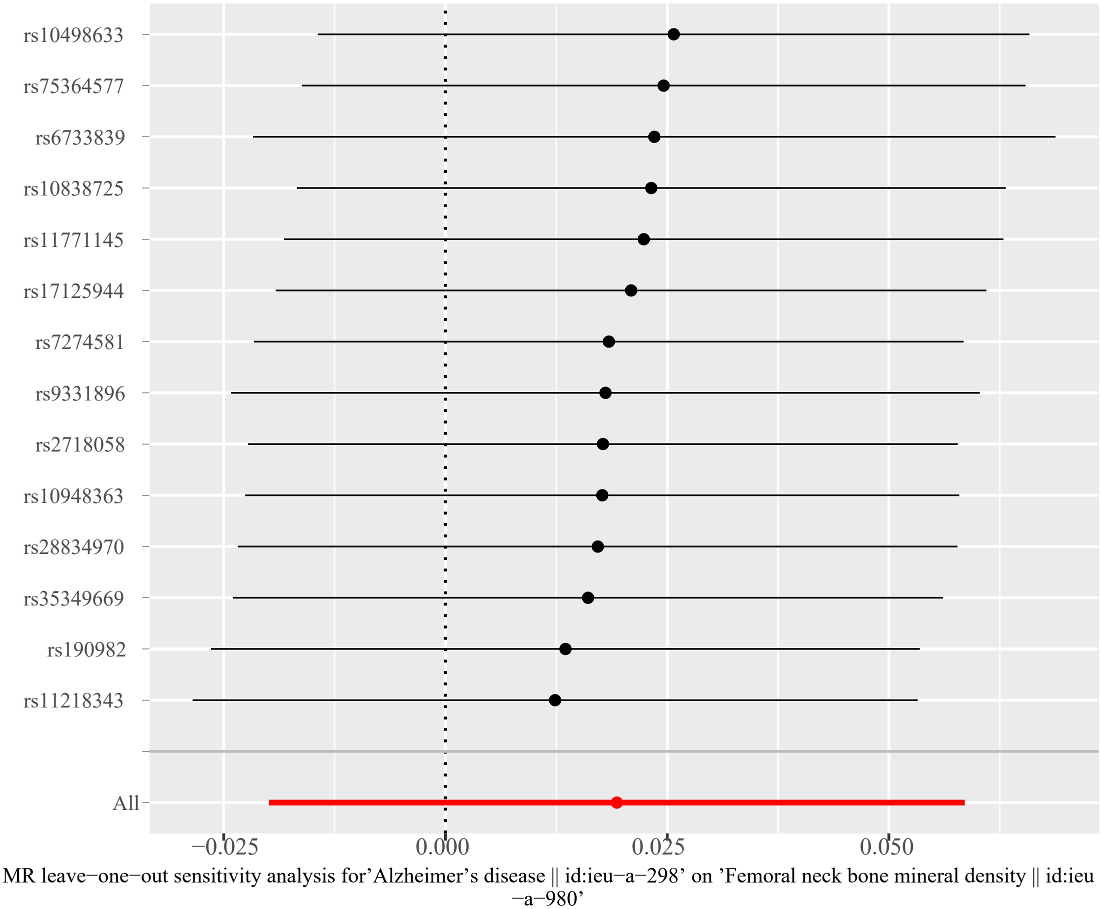

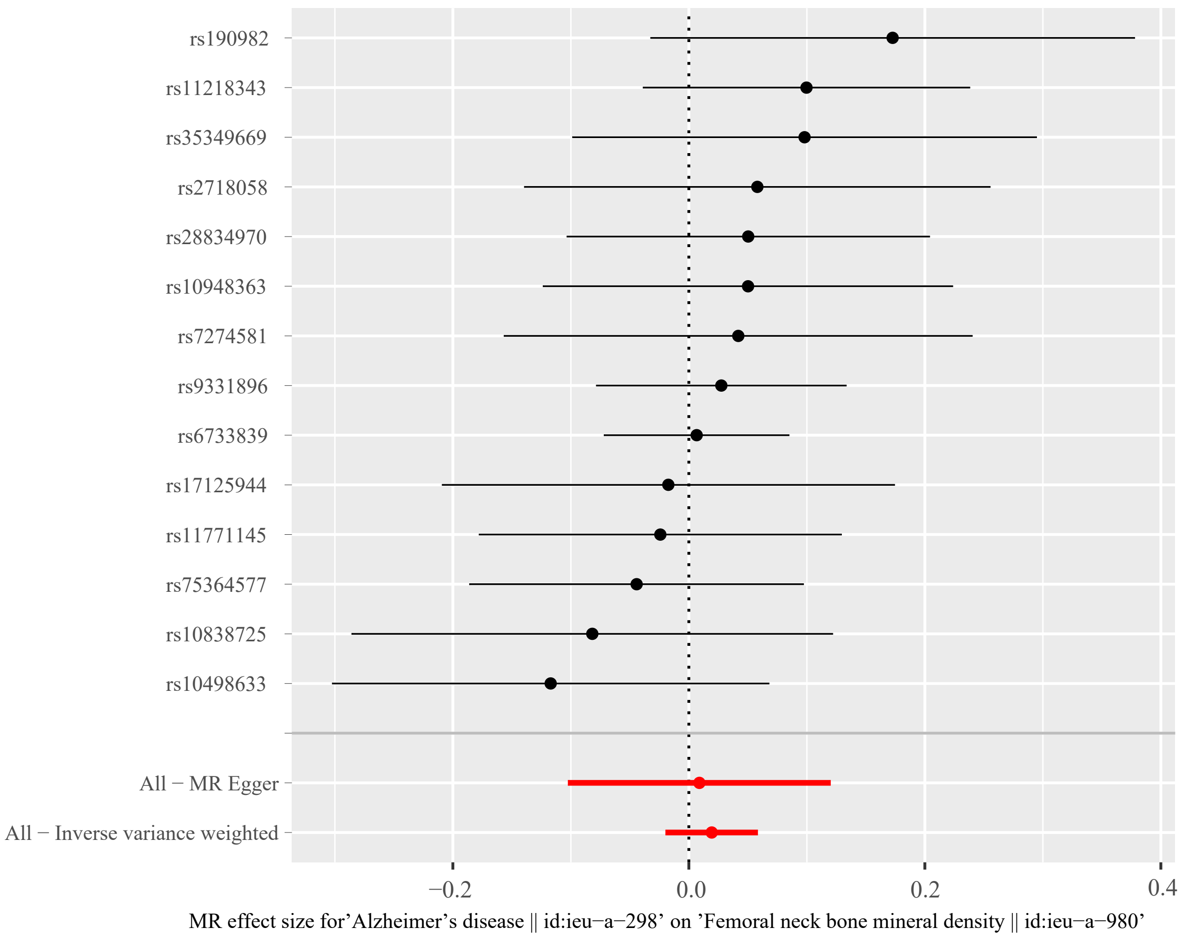
rs190982
rs11218343
rs35349669
rs2718058
rs28834970
rs10948363
rs7274581
rs9331896
rs6733839
rs17125944
rs11771145
rs75364577
rs10838725
rs10498633
All − MR Egger
All − Inverse variance weighted
−0.2
0.0
0.2
0.4
MR effect size for'Alzheimer's disease || id:ieu−a−298' on 'Femoral neck bone mineral density || id:ieu−a−980'

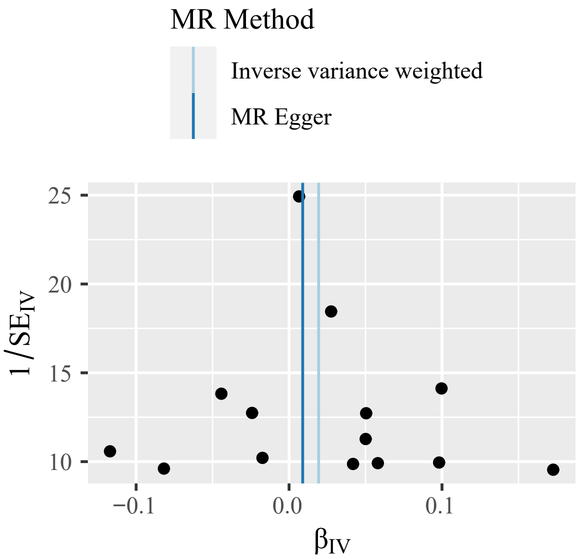
MR Method
Inverse variance weighted
MR Egger
25
20
15
10
−0.1
0.0
0.1
$1/SE_{IV}$
β_{IV}

Figure S5. Leave-one-out analysis, MR effect size and funnel plot for AD on LS-BMD.

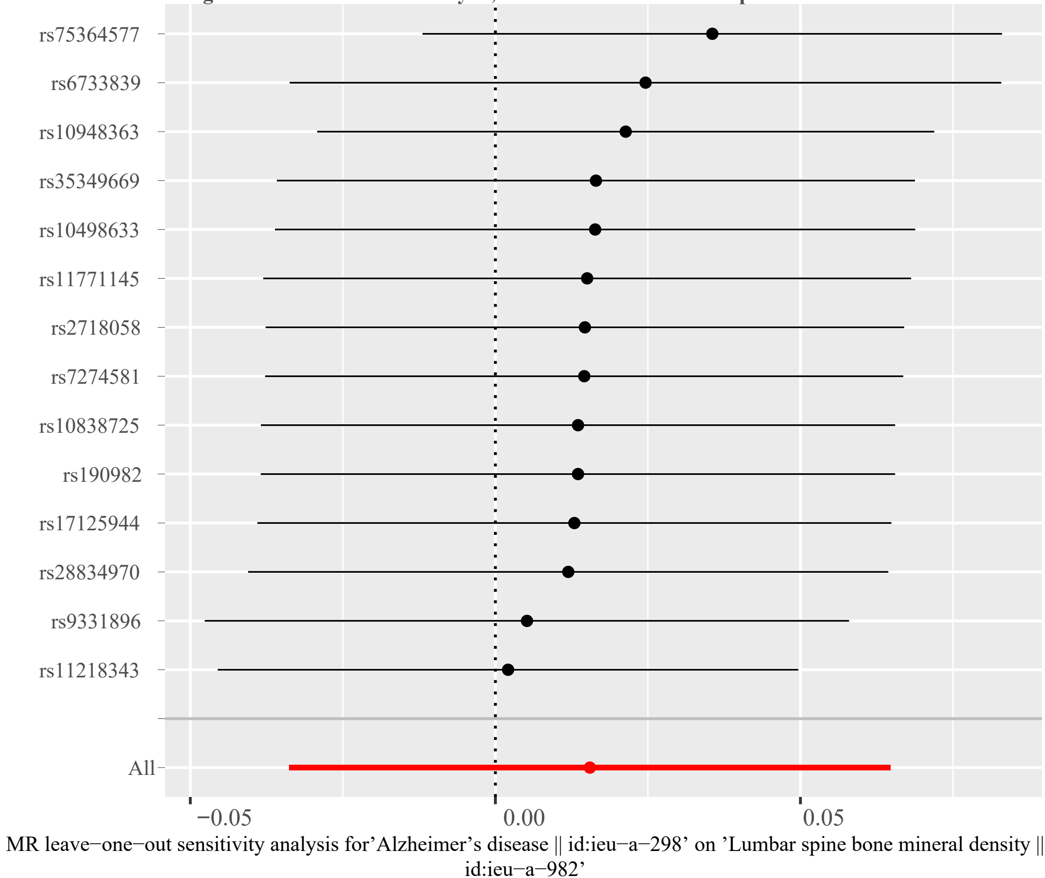

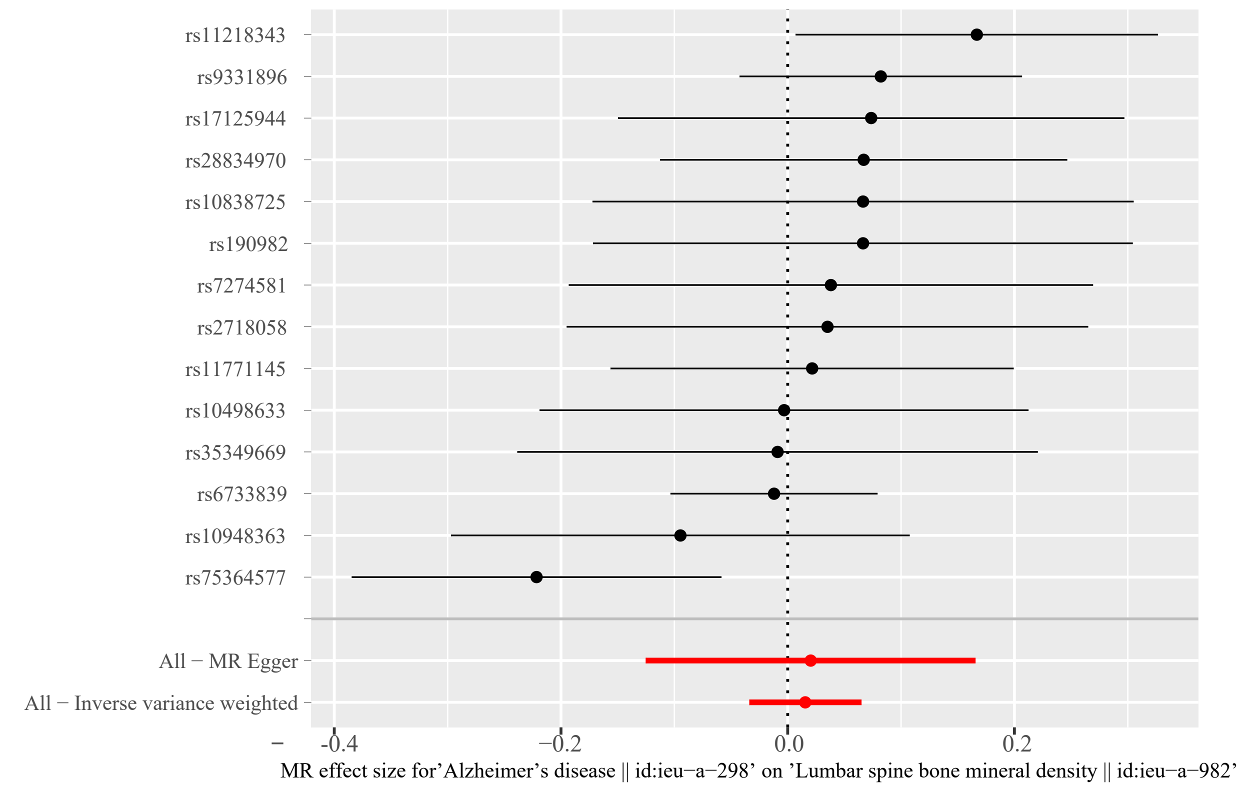
rs11218343
rs9331896
rs17125944
rs28834970
rs10838725
rs190982
rs7274581
rs2718058
rs11771145
rs10498633
rs35349669
rs6733839
rs10948363
rs75364577
All − MR Egger
All − Inverse variance weighted
-0.4
−0.2
0.0
0.2
MR effect size for'Alzheimer's disease || id:ieu−a−298' on 'Lumbar spine bone mineral density || id:ieu−a−982'

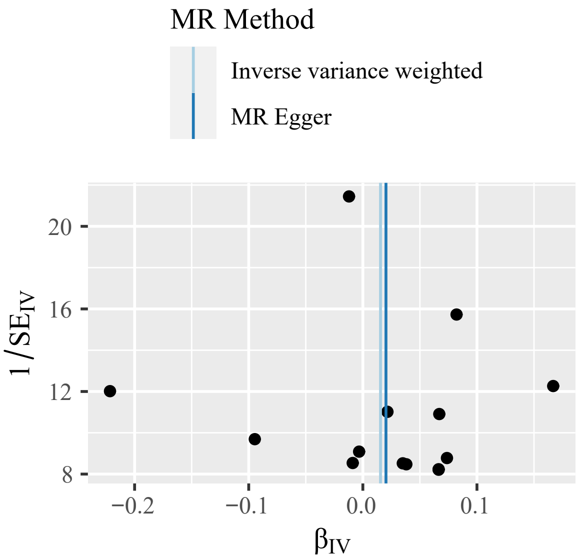
MR Method
Inverse variance weighted
MR Egger
20
16
12
8
$1/SE_{IV}$
−0.2
−0.1
0.0
0.1
β_{IV}

Figure S6. Leave-one-out analysis, MR effect size and funnel plot for AD on FA-BMD

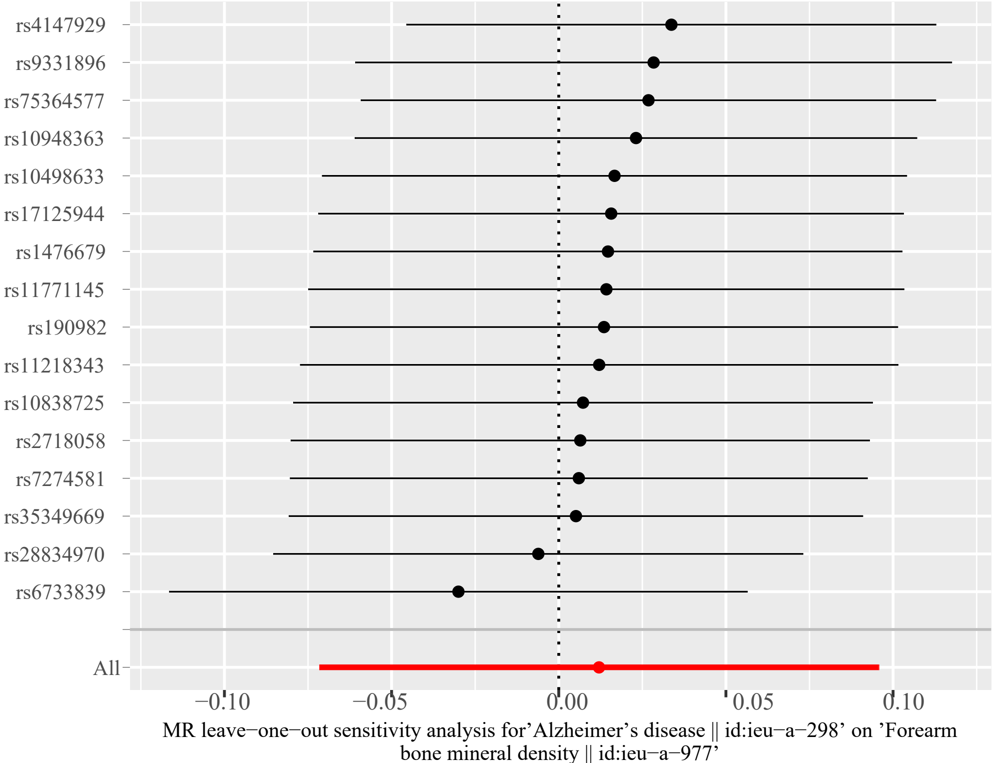

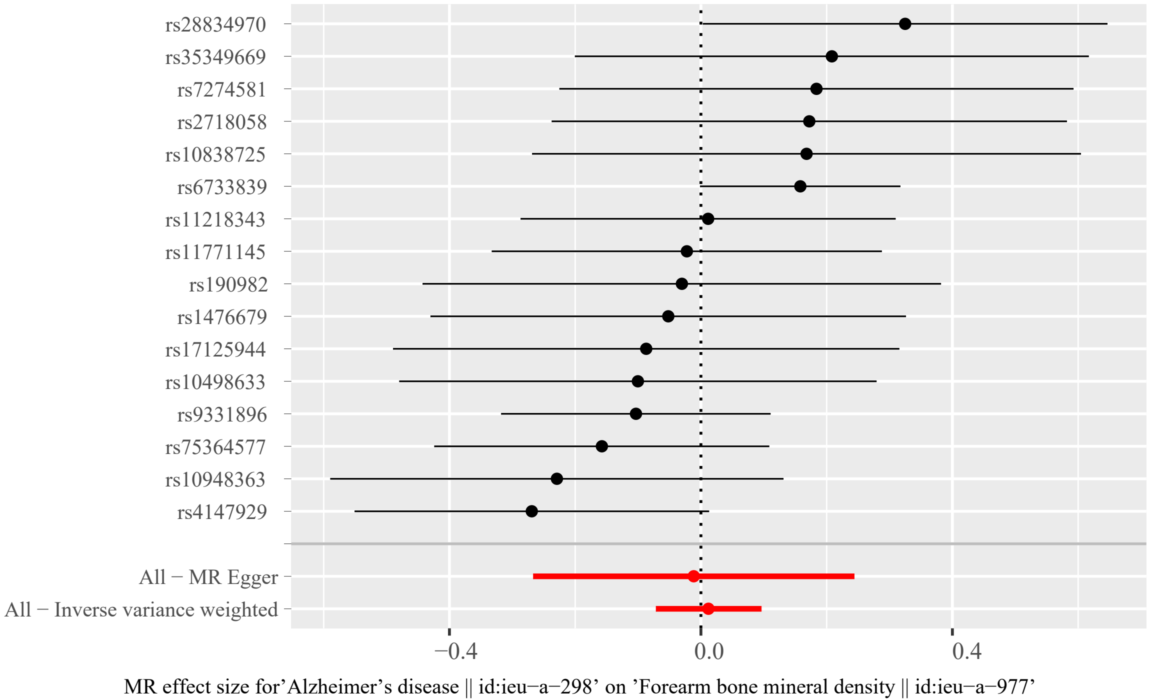
rs28834970
rs35349669
rs7274581
rs2718058
rs10838725
rs6733839
rs11218343
rs11771145
rs190982
rs1476679
rs17125944
rs10498633
rs9331896
rs75364577
rs10948363
rs4147929
All − MR Egger
All − Inverse variance weighted
−0.4
0.0
0.4
MR effect size for'Alzheimer's disease || id:ieu−a−298' on 'Forearm bone mineral density || id:ieu−a−977'

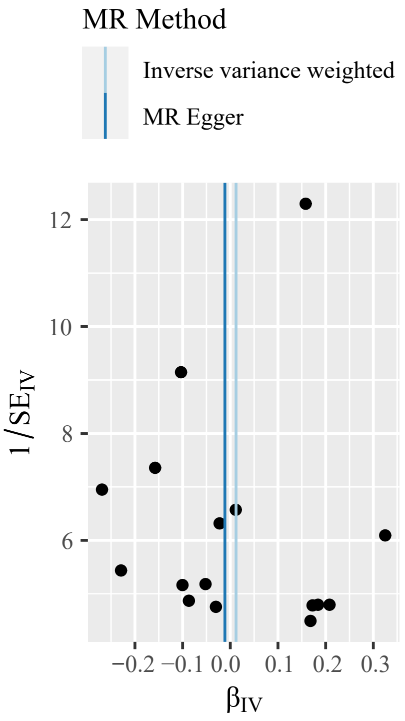
MR Method
Inverse variance weighted
MR Egger
12
10
8
6
1/SE_IV
−0.2
−0.1
0.0
0.1
0.2
0.3
β_IV

Figure S7. Leave-one-out analysis, MR effect size and funnel plot for AD on eBMD

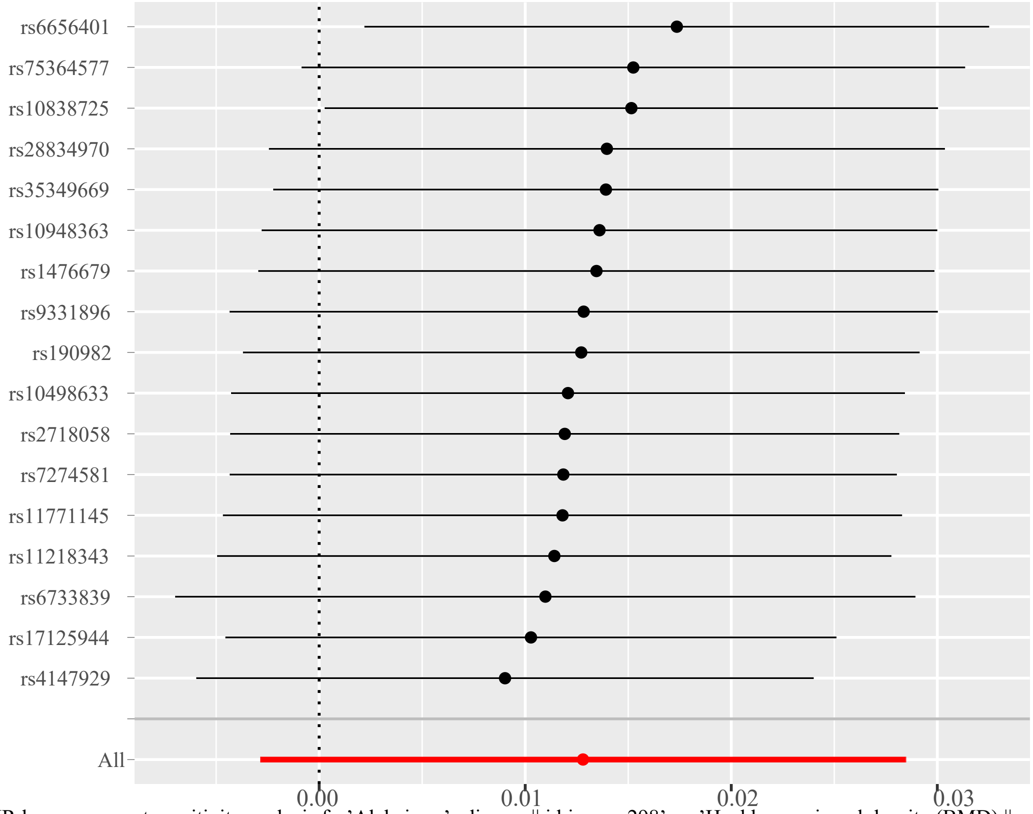

MR leave−one−out sensitivity analysis for'Alzheimer's disease || id:ieu−a−298' on 'Heel bone mineral density (BMD) || id:ukb−b−8875'

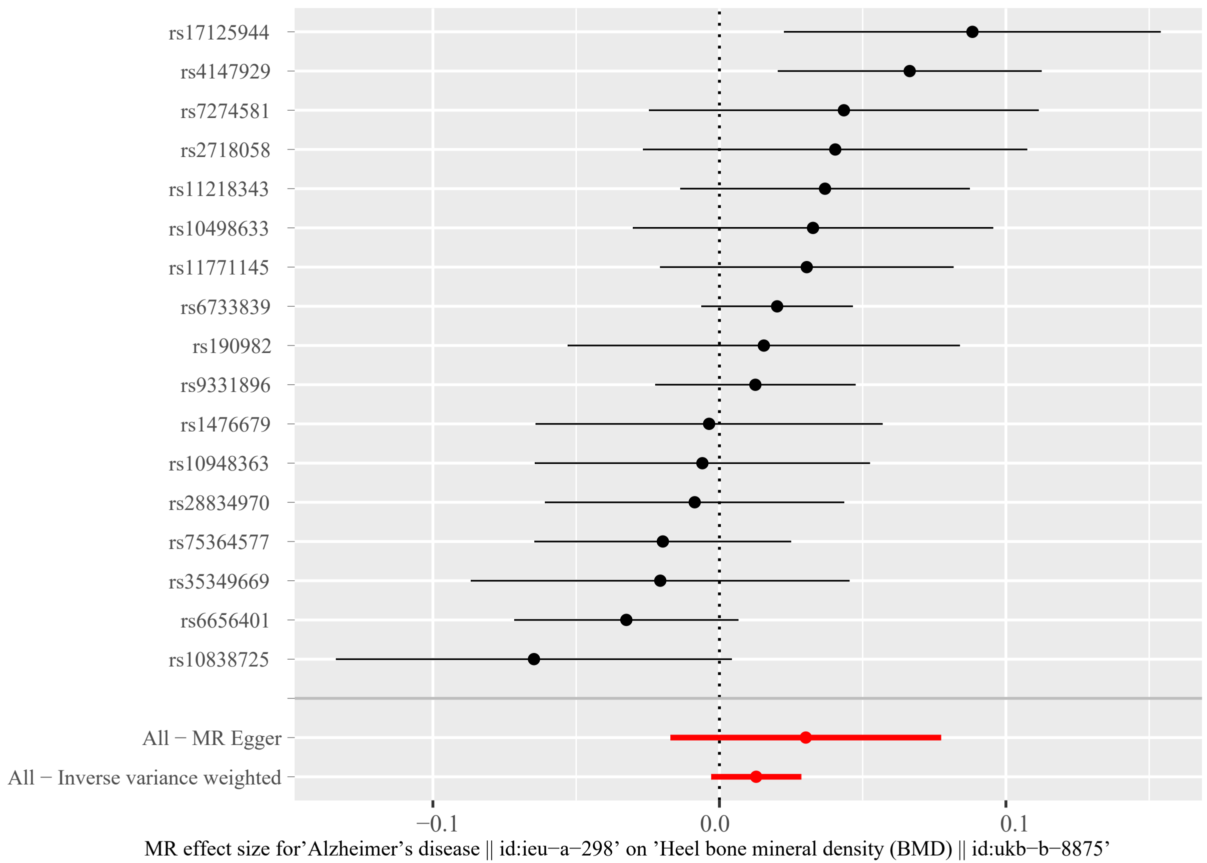

rs17125944
rs4147929
rs7274581
rs2718058
rs11218343
rs10498633
rs11771145
rs6733839
rs190982
rs9331896
rs1476679
rs10948363
rs28834970
rs75364577
rs35349669
rs6656401
rs10838725
All − MR Egger
All − Inverse variance weighted
−0.1
0.0
0.1
MR effect size for'Alzheimer's disease || id:ieu−a−298' on 'Heel bone mineral density (BMD) || id:ukb−b−8875'

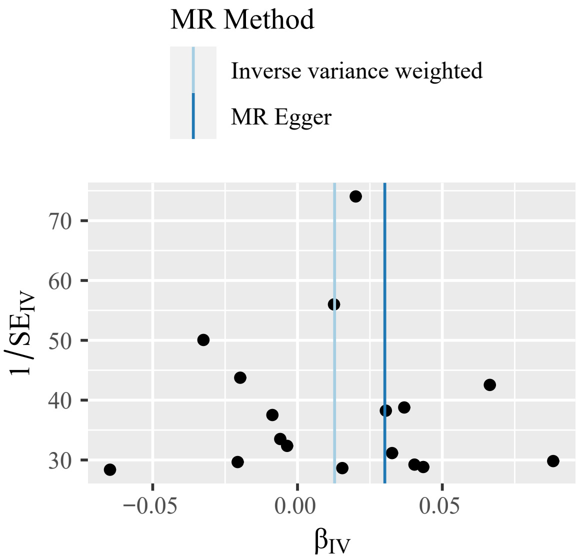

MR Method
Inverse variance weighted
MR Egger
70
60
50
40
30
1/SE$_{IV}$
−0.05
0.00
0.05
β$_{IV}$

Figure S8. Leave-one-out analysis, MR effect size and funnel plot for AD on LF.

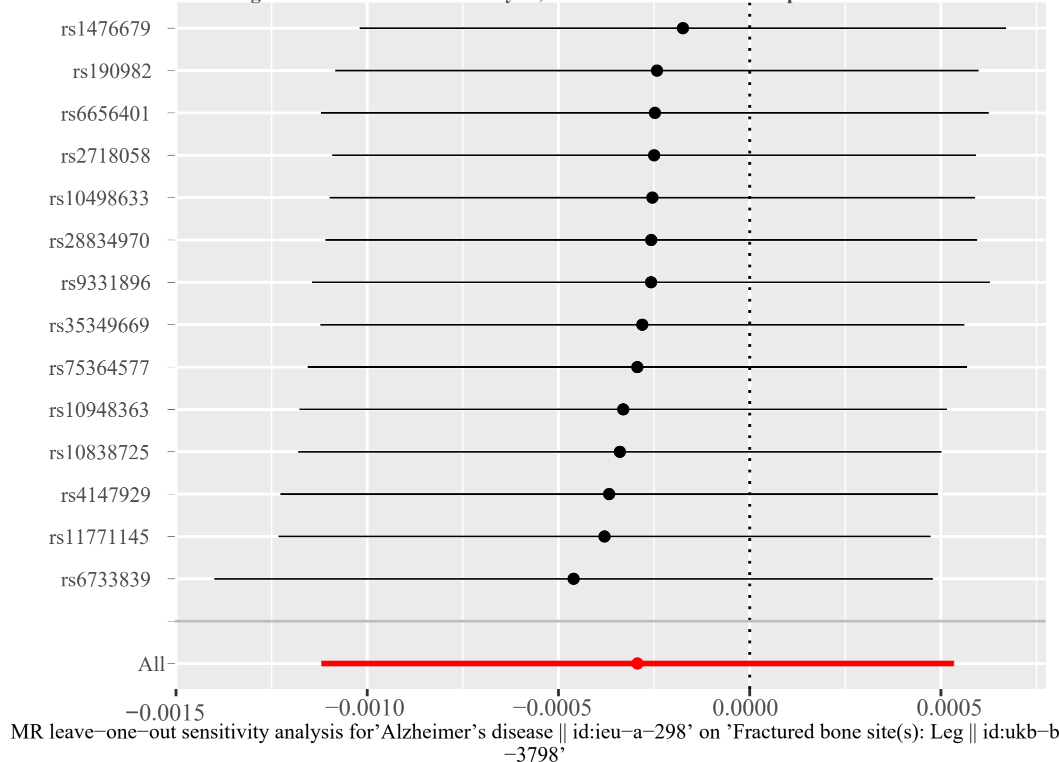

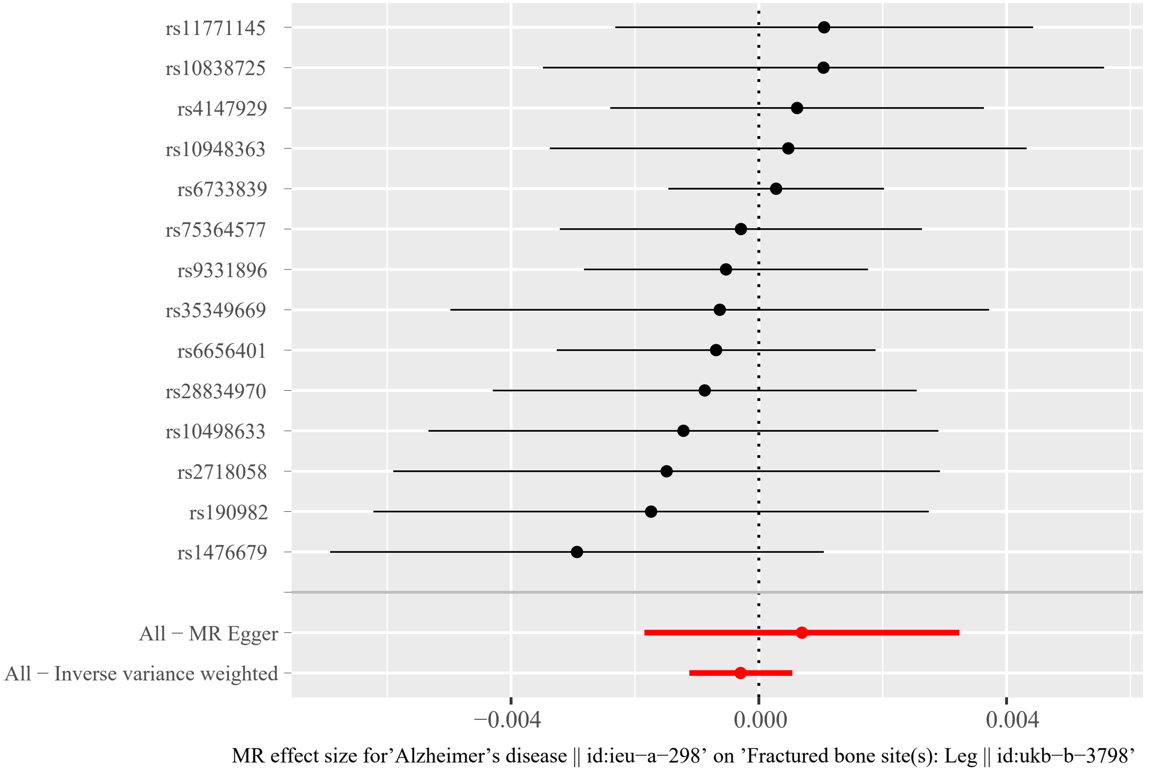
rs11771145
rs10838725
rs4147929
rs10948363
rs6733839
rs75364577
rs9331896
rs35349669
rs6656401
rs28834970
rs10498633
rs2718058
rs190982
rs1476679
All − MR Egger
All − Inverse variance weighted
−0.004
0.000
0.004
MR effect size for'Alzheimer's disease || id:ieu−a−298' on 'Fractured bone site(s): Leg || id:ukb−b−3798'

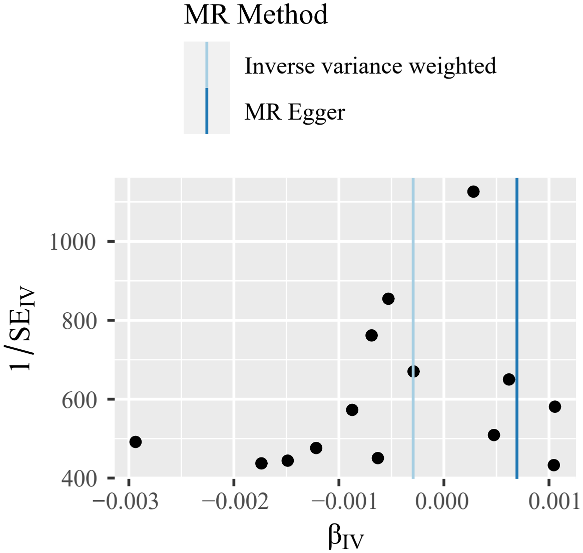

MR Method
Inverse variance weighted
MR Egger
1000
800
600
400
$1/SE_{IV}$
−0.003
−0.002
−0.001
0.000
0.001
β_{IV}

Figure S9. Leave-one-out analysis, MR effect size and funnel plot for AD on AF.

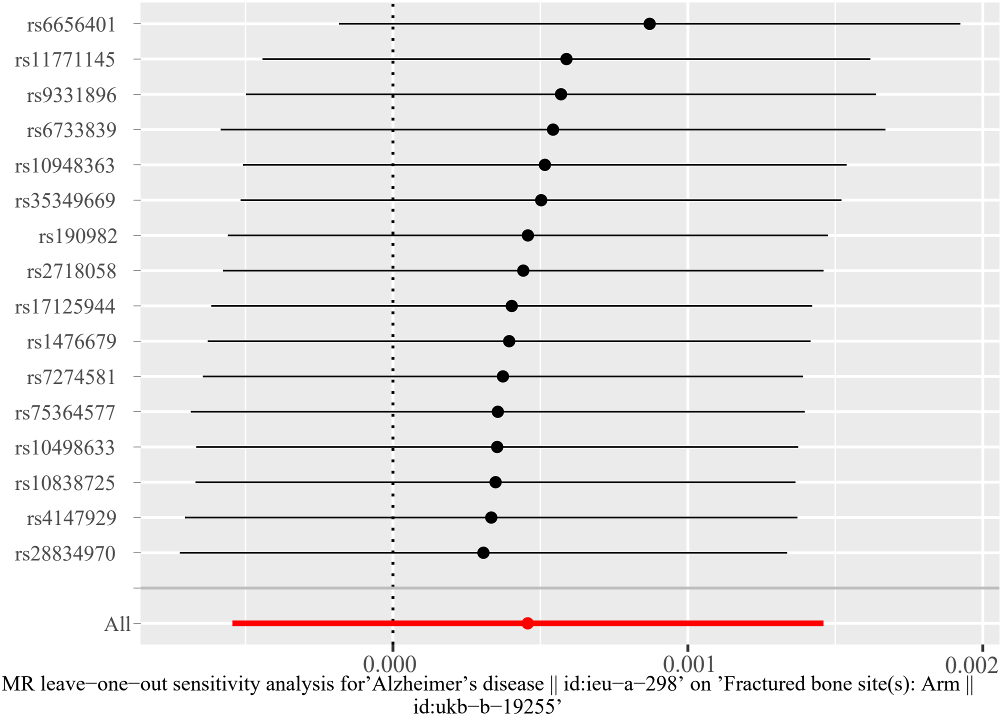

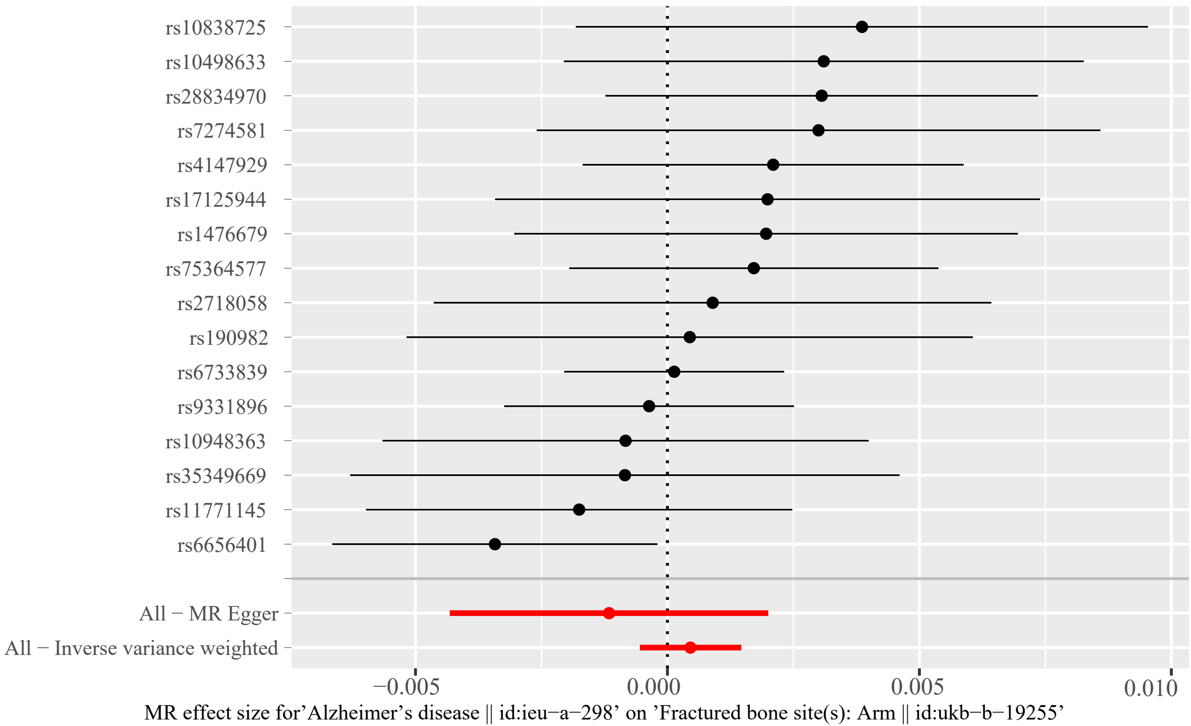
rs10838725
rs10498633
rs28834970
rs7274581
rs4147929
rs17125944
rs1476679
rs75364577
rs2718058
rs190982
rs6733839
rs9331896
rs10948363
rs35349669
rs11771145
rs6656401
All − MR Egger
All − Inverse variance weighted
−0.005
0.000
0.005
0.010
MR effect size for’Alzheimer’s disease || id:ieu−a−298’ on ’Fractured bone site(s): Arm || id:ukb−b−19255’

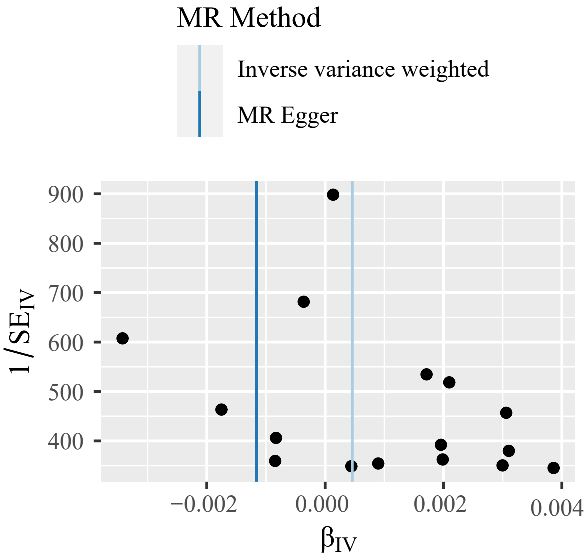

MR Method
Inverse variance weighted
MR Egger
900
800
700
600
500
400
$1/SE_{IV}$
−0.002
0.000
0.002
0.004
β_{IV}

Figure S10 . Leave-one-out analysis, MR effect size and funnel plot for AD on SF.

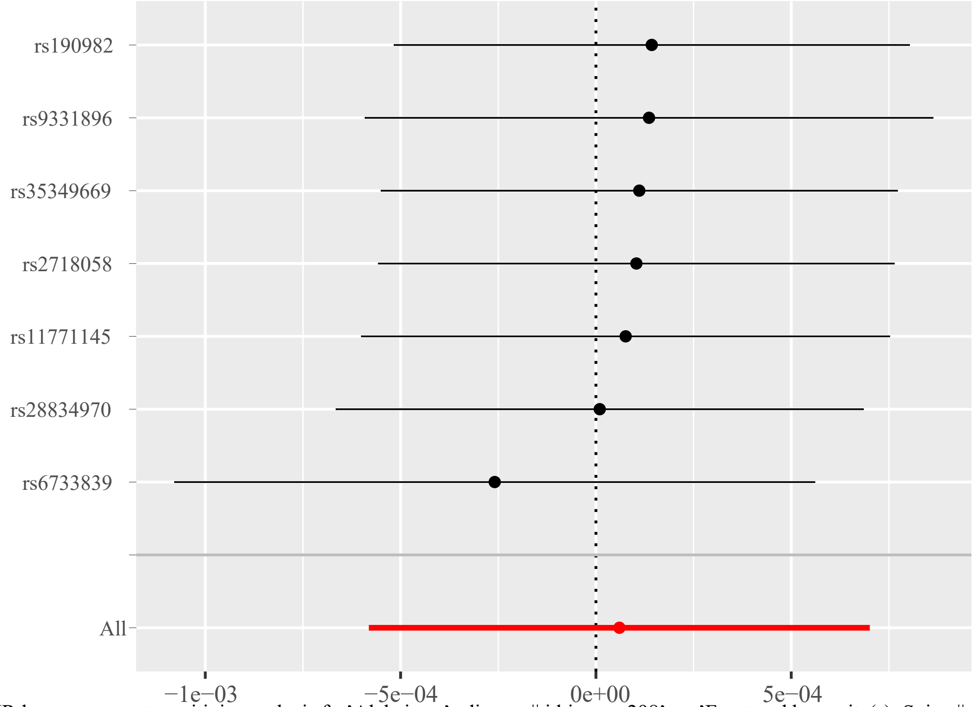

MR leave−one−out sensitivity analysis for’Alzheimer’s disease || id:ieu−a−298’ on ’Fractured bone site(s): Spine || id:ukb−b−873’

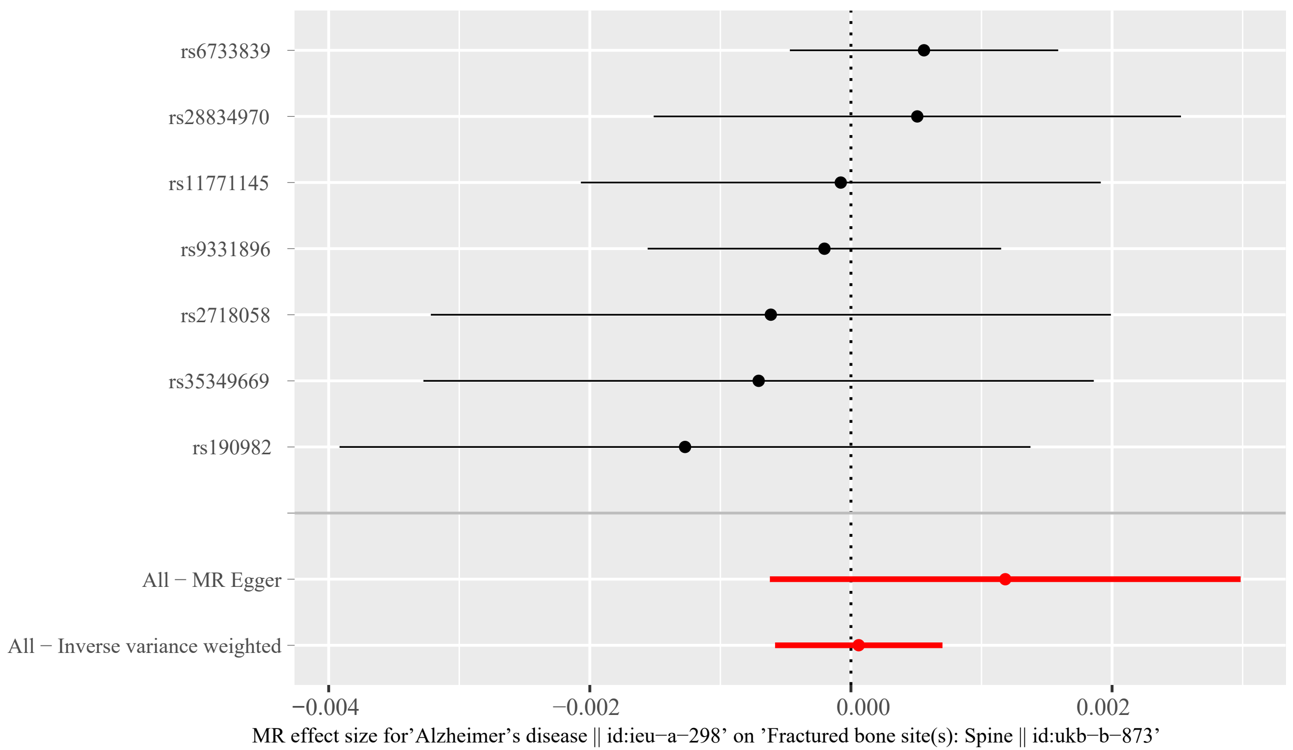
rs6733839
rs28834970
rs11771145
rs9331896
rs2718058
rs35349669
rs190982
All − MR Egger
All − Inverse variance weighted
−0.004
−0.002
0.000
0.002
MR effect size for'Alzheimer's disease || id:ieu−a−298' on 'Fractured bone site(s): Spine || id:ukb−b−873'

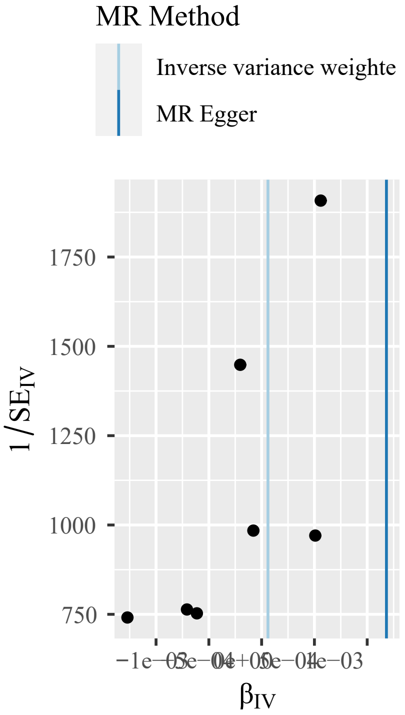
MR Method
Inverse variance weighte
MR Egger
1750
1500
1250
1000
750
1/SE_IV
β_IV

Figure S11. Leave-one-out analysis, MR effect size and funnel plot for AD on HF.

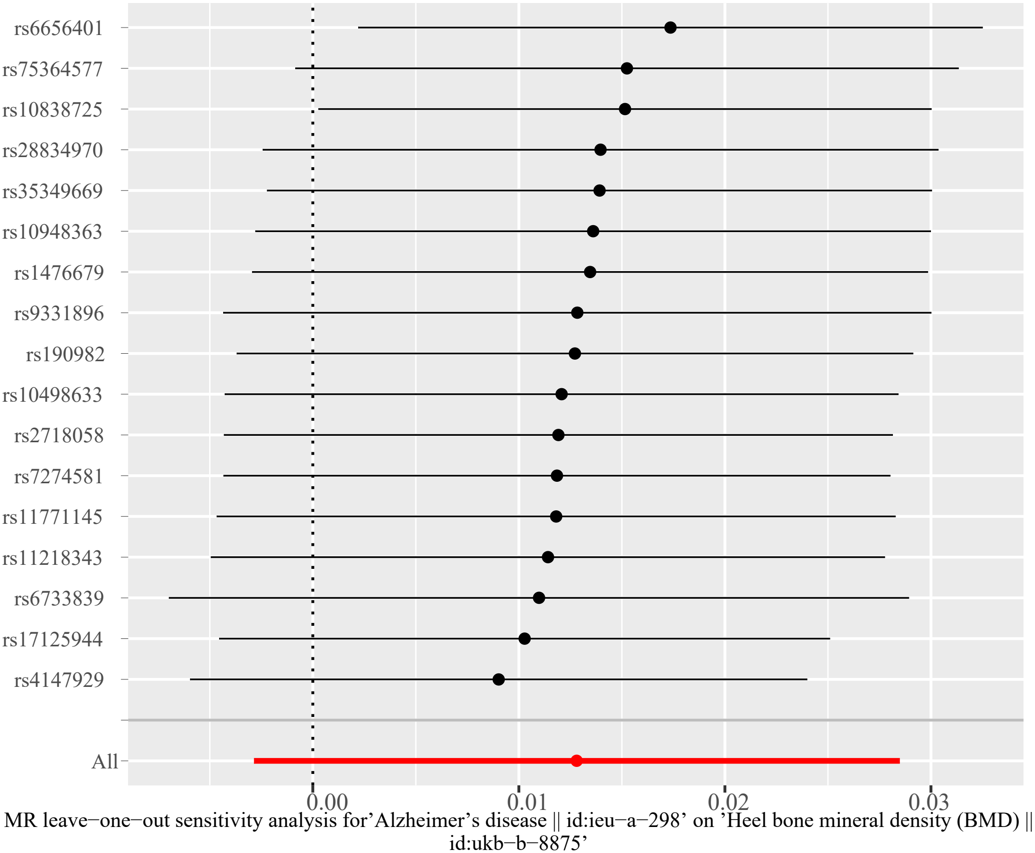

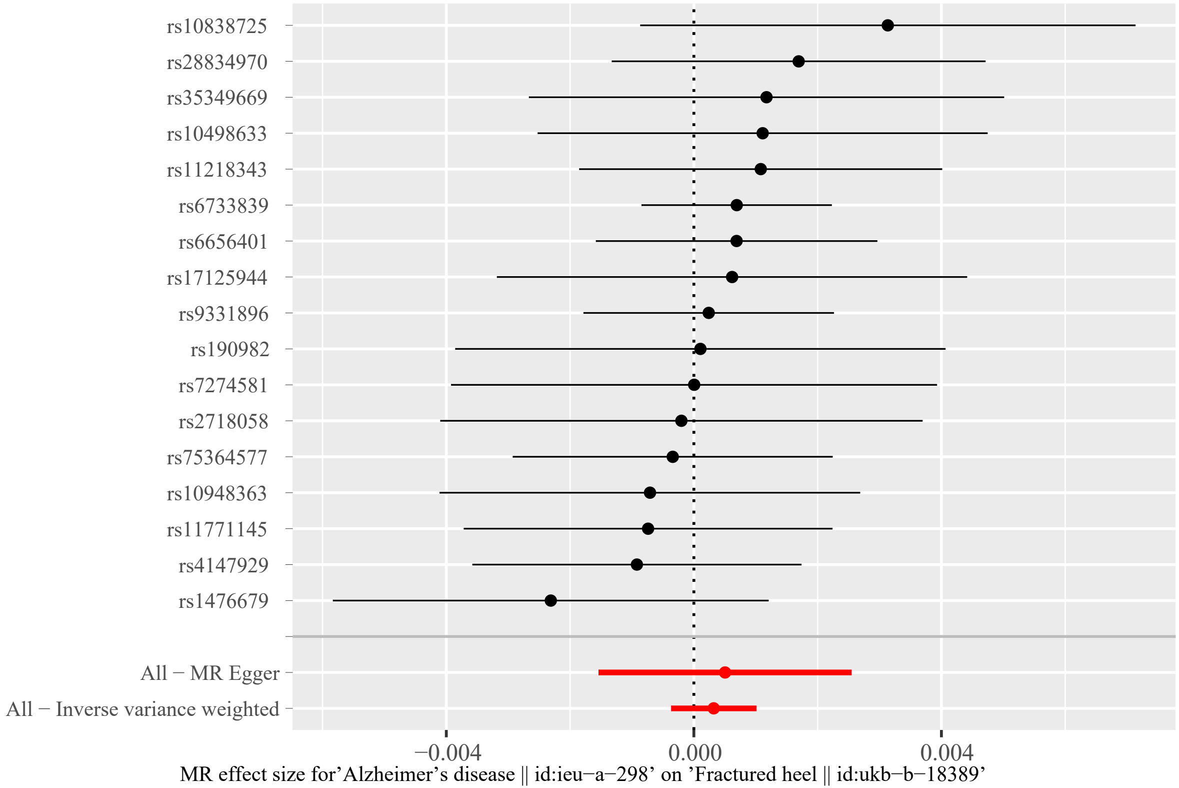
rs10838725
rs28834970
rs35349669
rs10498633
rs11218343
rs6733839
rs6656401
rs17125944
rs9331896
rs190982
rs7274581
rs2718058
rs75364577
rs10948363
rs11771145
rs4147929
rs1476679
All − MR Egger
All − Inverse variance weighted
−0.004
0.000
0.004
MR effect size for'Alzheimer's disease || id:ieu−a−298' on 'Fractured heel || id:ukb−b−18389'

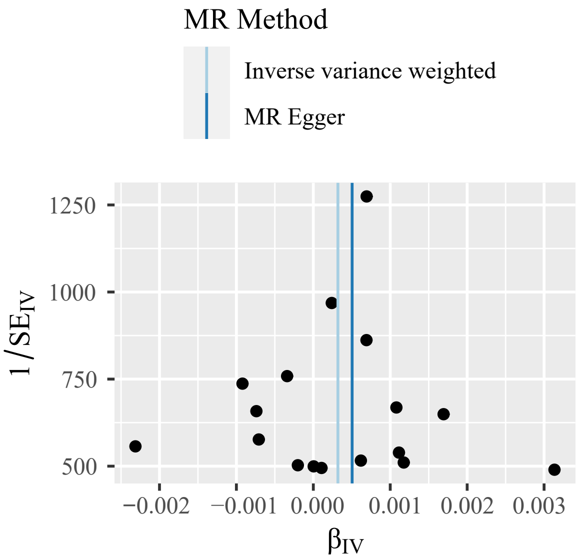

MR Method
Inverse variance weighted
MR Egger
1250
1000
750
500
$1/SE_{IV}$
−0.002
−0.001
0.000
0.001
0.002
0.003
β_{IV}
