# Supplementary material for: Mendelian randomization analysis does not reveal a causal influence of mental diseases on osteoporosis
Source: Front Endocrinol (Lausanne). 2023 Apr 20;14:1125427. doi: 10.3389/fendo.2023.1125427 (PMC10157183; doi:10.3389/fendo.2023.1125427)
Supplement: Supplementary Material 1 — Instrumental variables SNPs. [file DataSheet_1.zip › Supplementary Material/Supplementary Material 6.pdf]

Figure S1 Leave-one-out analysis, MR effect size and funnel plot for PD on OP.

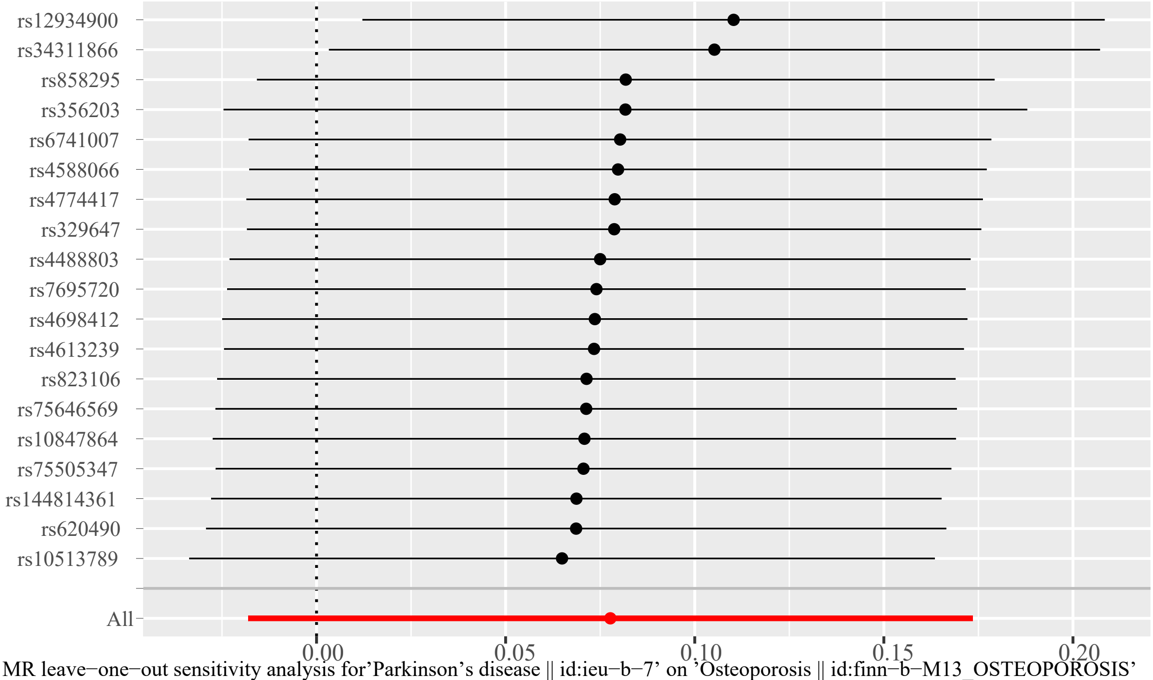

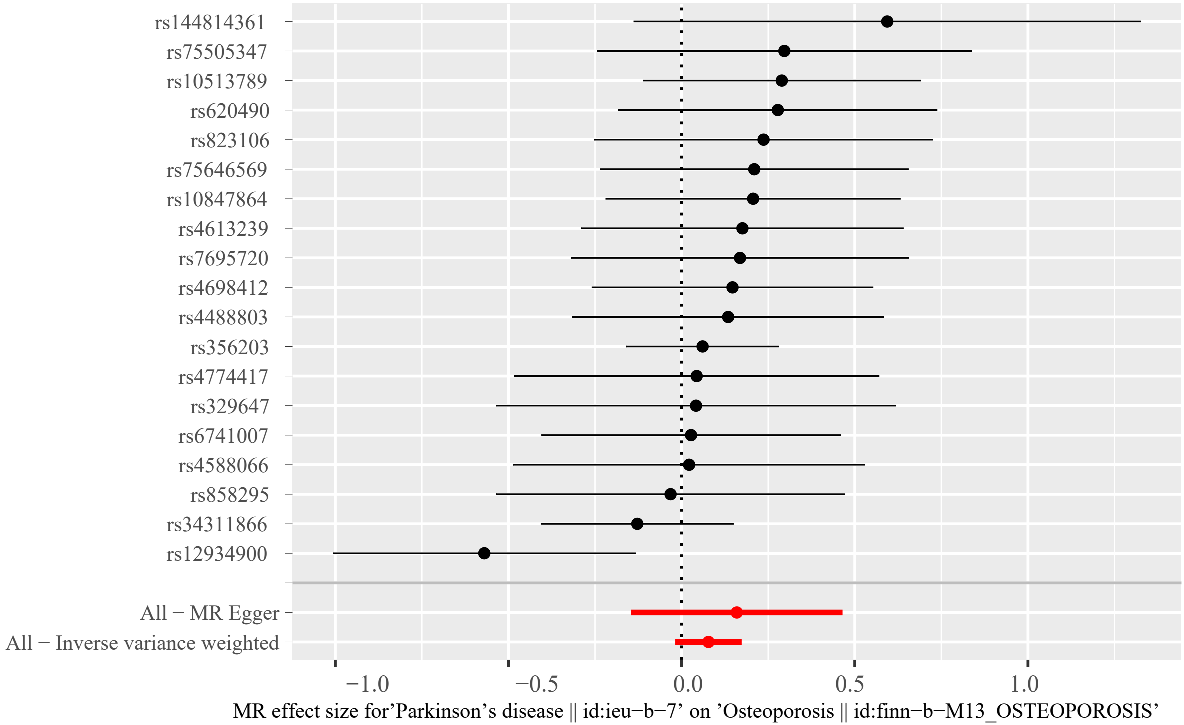
rs144814361
rs75505347
rs10513789
rs620490
rs823106
rs75646569
rs10847864
rs4613239
rs7695720
rs4698412
rs4488803
rs356203
rs4774417
rs329647
rs6741007
rs4588066
rs858295
rs34311866
rs12934900
All − MR Egger
All − Inverse variance weighted
−1.0
−0.5
0.0
0.5
1.0
MR effect size for'Parkinson's disease || id:ieu−b−7' on 'Osteoporosis || id:finn−b−M13_OSTEOPOROSIS'

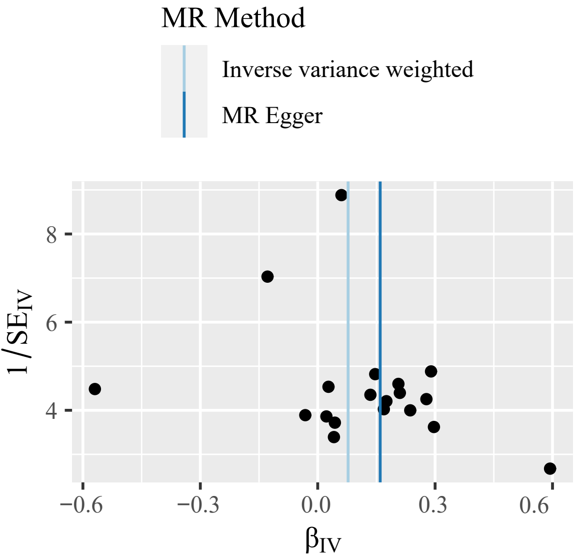
MR Method
Inverse variance weighted
MR Egger
$1/SE_{IV}$
8
6
4
−0.6
−0.3
0.0
0.3
0.6
β_{IV}

Figure S2. Leave-one-out analysis, MR effect size and funnel plot for PD on OPF.

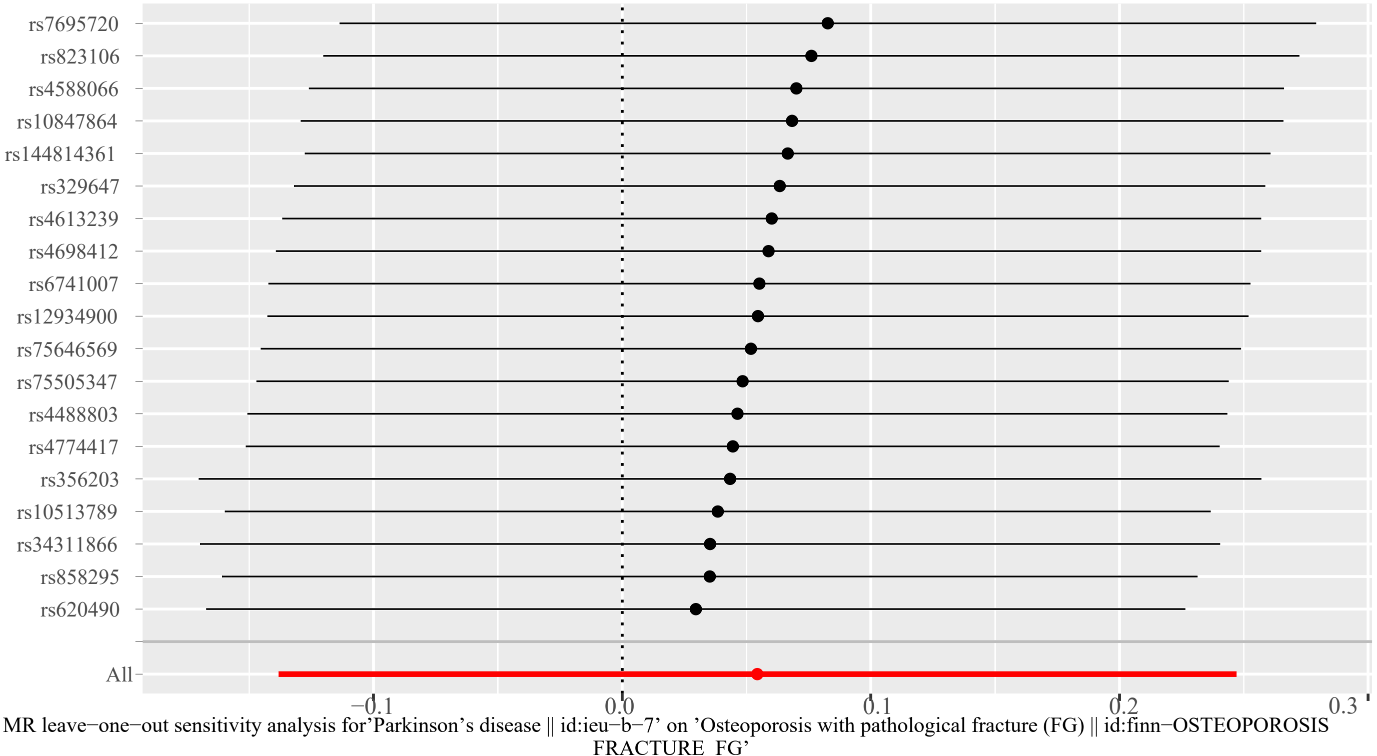

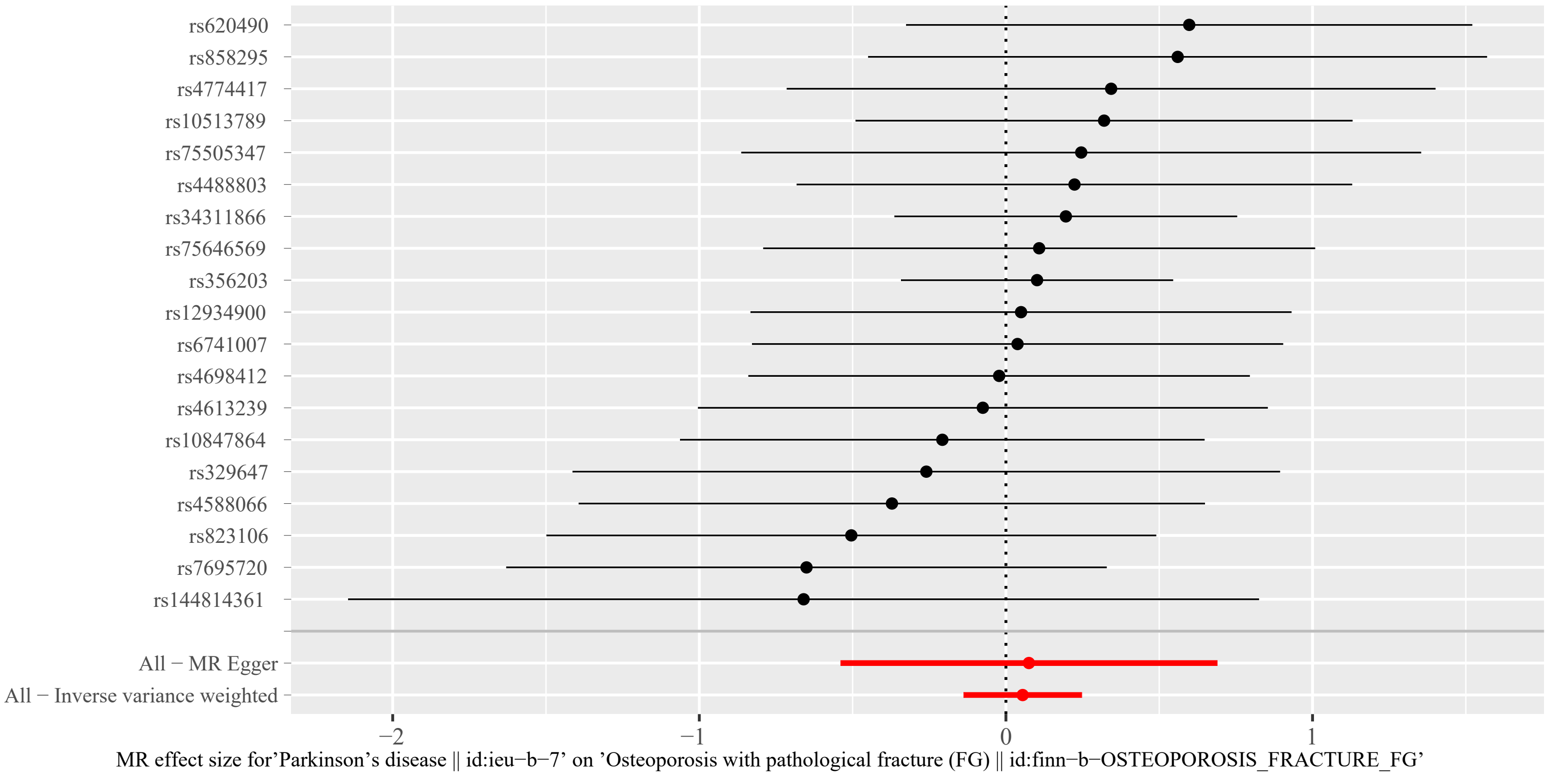

rs620490
rs858295
rs4774417
rs10513789
rs75505347
rs4488803
rs34311866
rs75646569
rs356203
rs12934900
rs6741007
rs4698412
rs4613239
rs10847864
rs329647
rs4588066
rs823106
rs7695720
rs144814361
All − MR Egger
All − Inverse variance weighted
−2
−1
0
1
MR effect size for'Parkinson's disease || id:ieu−b−7' on 'Osteoporosis with pathological fracture (FG) || id:finn−b−OSTEOPOROSIS_FRACTURE_FG'

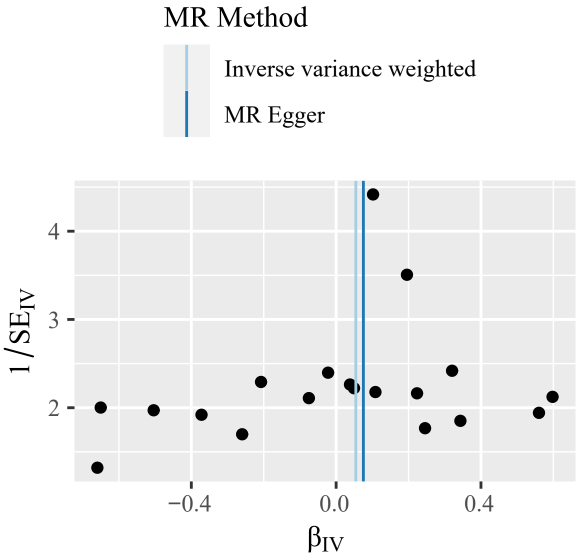
MR Method
Inverse variance weighted
MR Egger
$1/SE_{IV}$
4
3
2
−0.4
0.0
0.4
β_{IV}

Figure S3. Leave-one-out analysis, MR effect size and funnel plot for PD on TB-BMD.

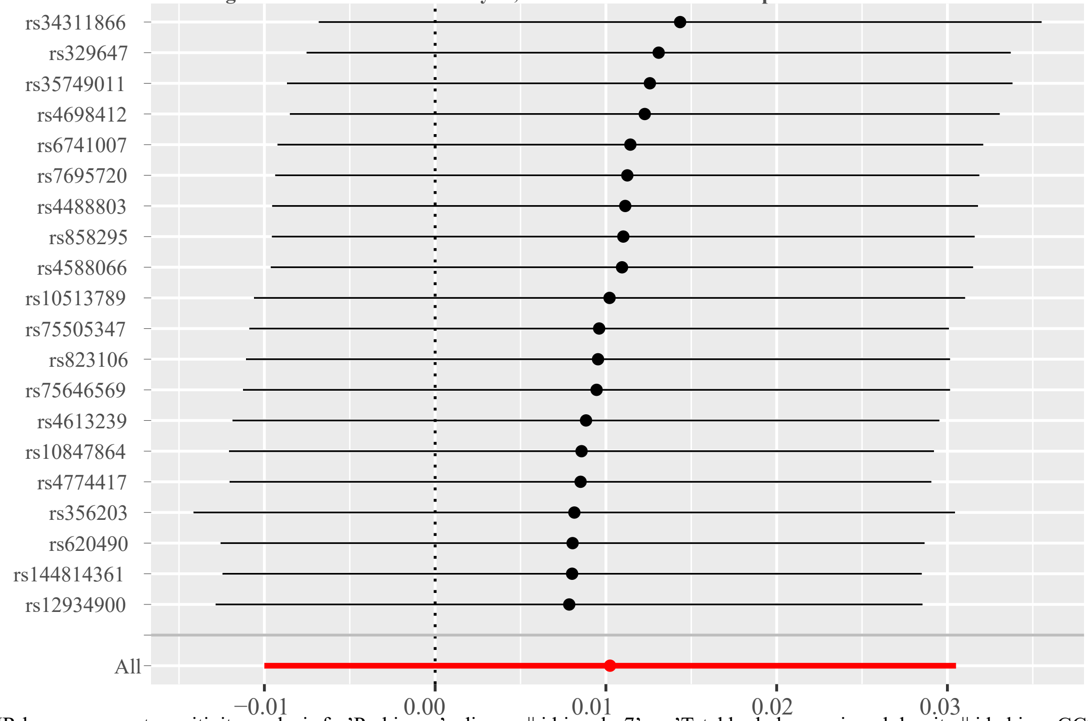

MR leave−one−out sensitivity analysis for'Parkinson's disease || id:ieu−b−7' on 'Total body bone mineral density || id:ebi−a−GCS 005348'

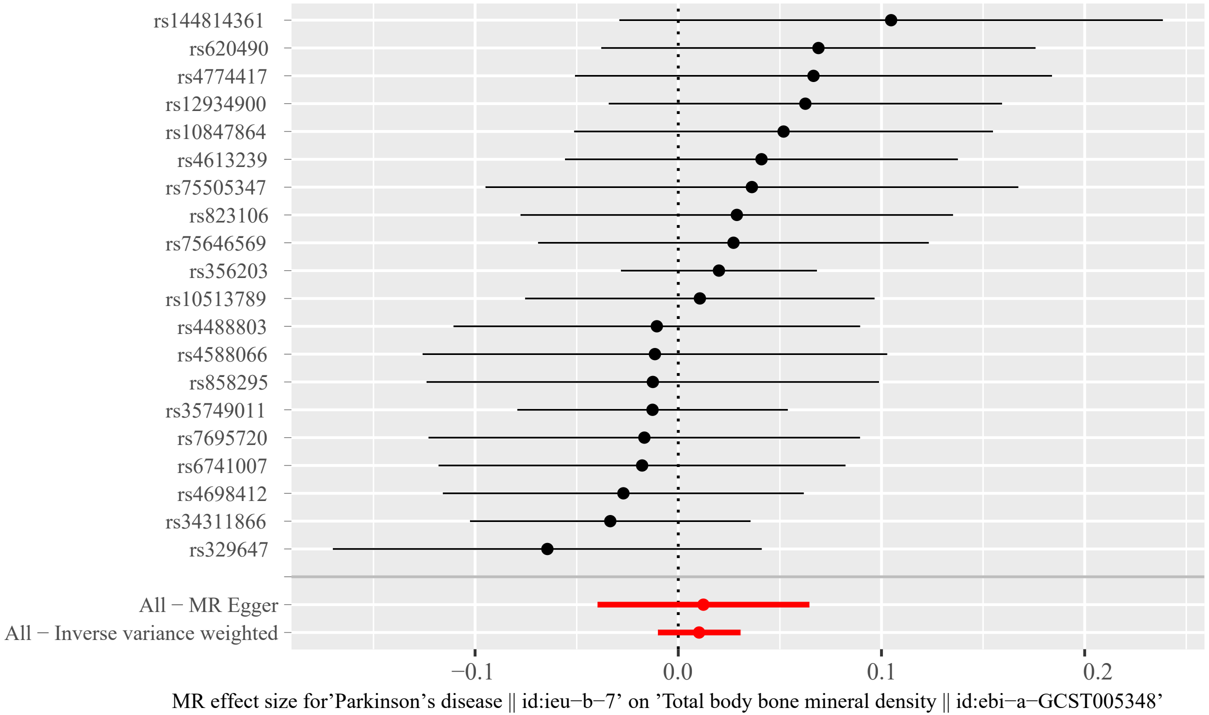
rs144814361
rs620490
rs4774417
rs12934900
rs10847864
rs4613239
rs75505347
rs823106
rs75646569
rs356203
rs10513789
rs4488803
rs4588066
rs858295
rs35749011
rs7695720
rs6741007
rs4698412
rs34311866
rs329647
All − MR Egger
All − Inverse variance weighted
−0.1
0.0
0.1
0.2
MR effect size for’Parkinson’s disease || id:ieu−b−7’ on ’Total body bone mineral density || id:ebi−a−GCST005348’

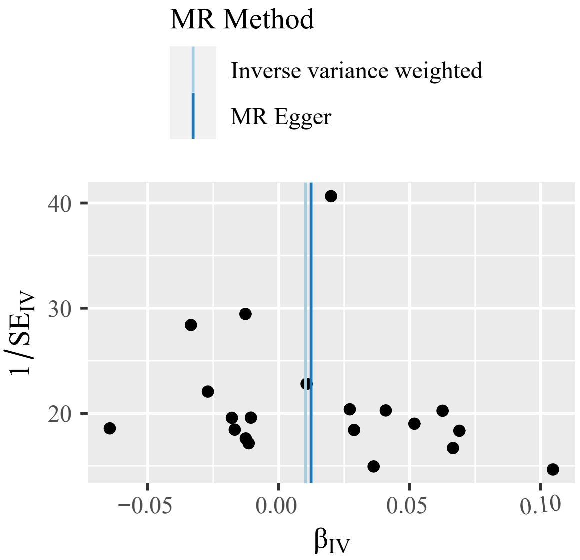
MR Method
Inverse variance weighted
MR Egger
40
30
20
$1/SE_{IV}$
−0.05
0.00
0.05
0.10
β_{IV}

Figure S4. Leave-one-out analysis, MR effect size and funnel plot for PD on FN-BMD.

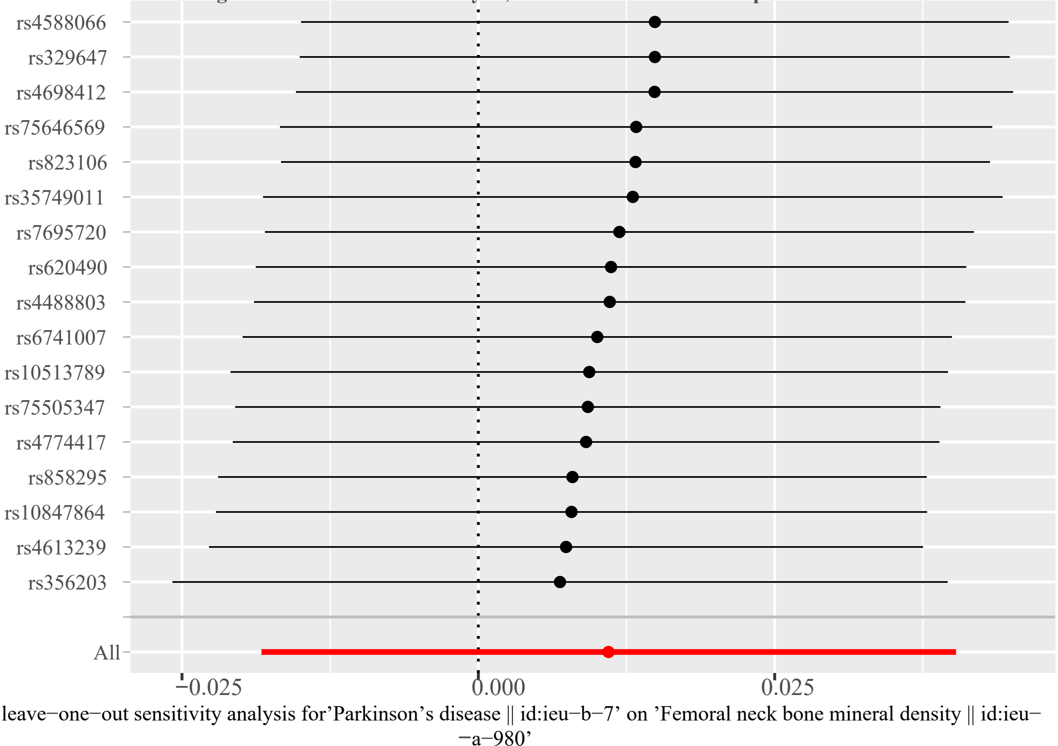

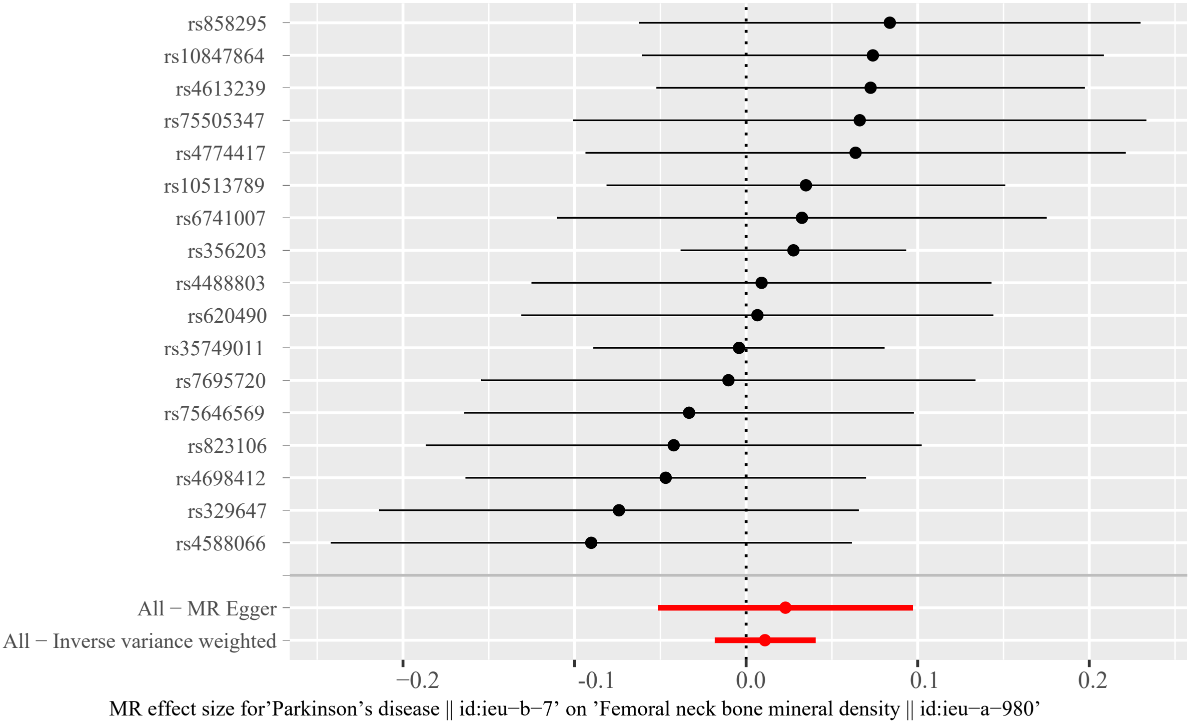
rs858295
rs10847864
rs4613239
rs75505347
rs4774417
rs10513789
rs6741007
rs356203
rs4488803
rs620490
rs35749011
rs7695720
rs75646569
rs823106
rs4698412
rs329647
rs4588066
All − MR Egger
All − Inverse variance weighted
−0.2
-0.1
0.0
0.1
0.2
MR effect size for'Parkinson's disease || id:ieu−b−7' on 'Femoral neck bone mineral density || id:ieu−a−980'

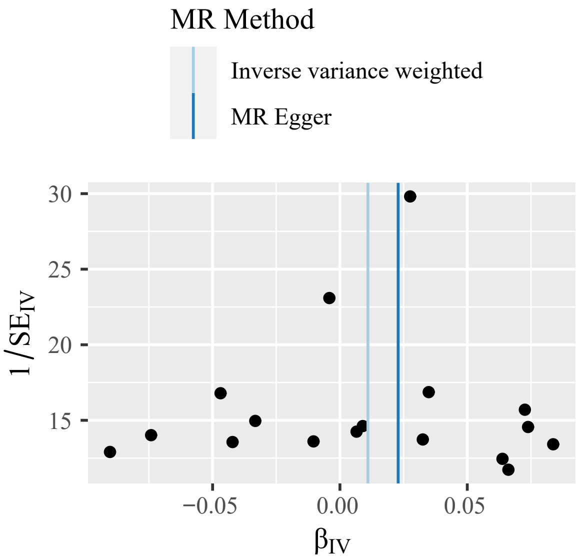
MR Method
Inverse variance weighted
MR Egger
30
25
20
15
1/SE_IV
−0.05
0.00
0.05
β_IV

Figure S5. Leave-one-out analysis, MR effect size and funnel plot for PD on LS-BMD.

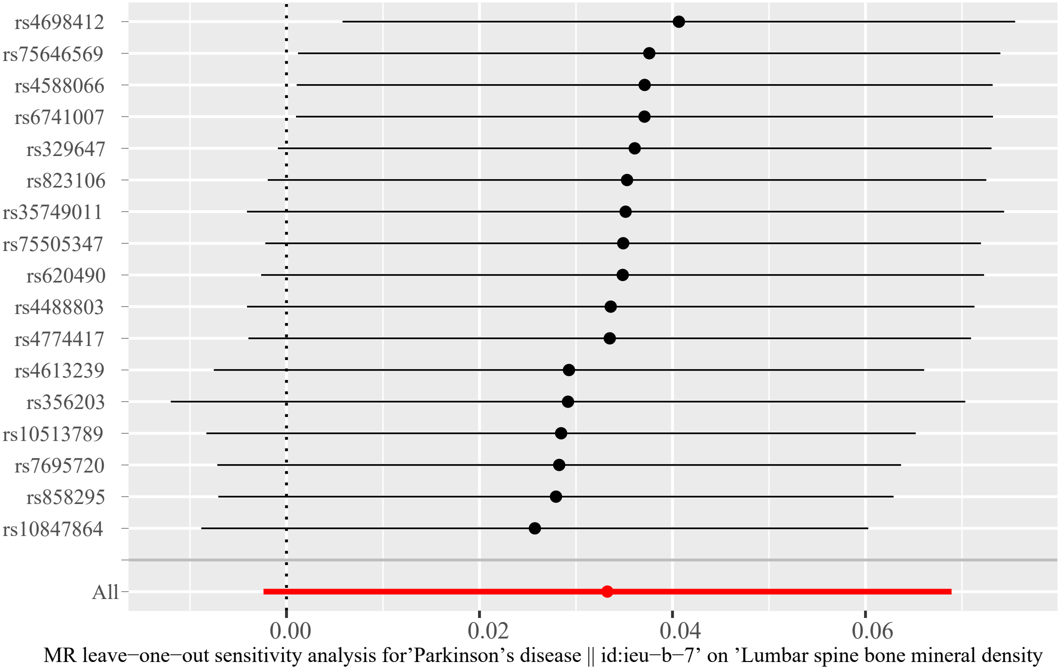

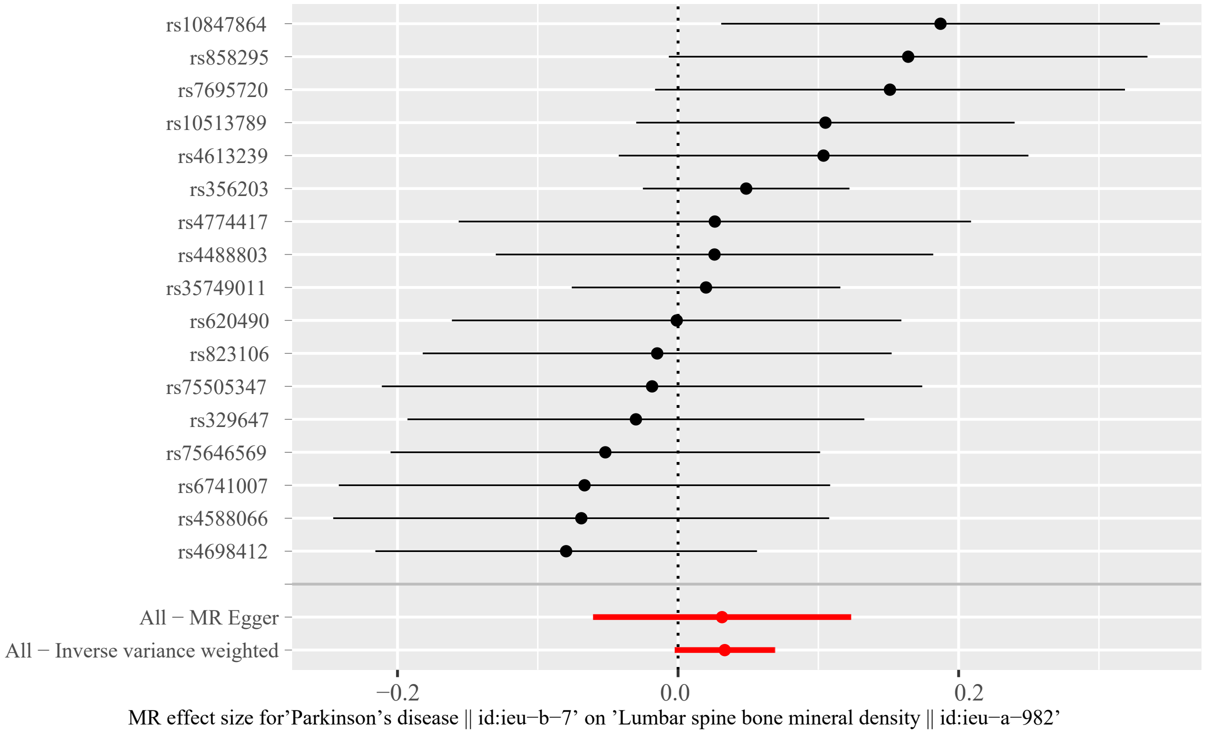
rs10847864
rs858295
rs7695720
rs10513789
rs4613239
rs356203
rs4774417
rs4488803
rs35749011
rs620490
rs823106
rs75505347
rs329647
rs75646569
rs6741007
rs4588066
rs4698412
All − MR Egger
All − Inverse variance weighted
−0.2
0.0
0.2
MR effect size for'Parkinson's disease || id:ieu−b−7' on 'Lumbar spine bone mineral density || id:ieu−a−982'

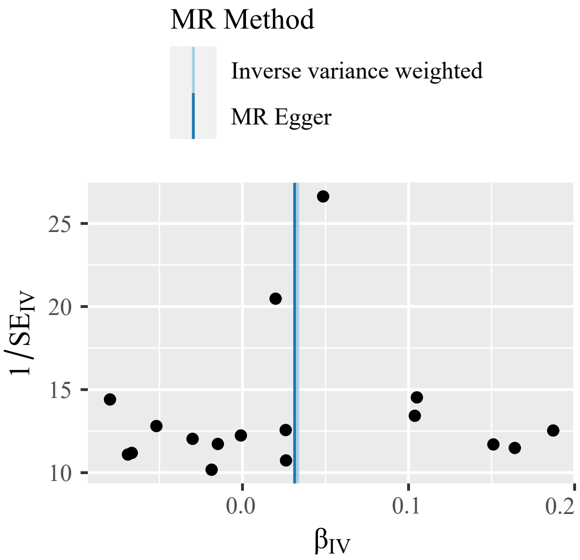

MR Method
Inverse variance weighted
MR Egger
25
20
15
10
0.0
0.1
0.2
1/SE_IV
β_IV

Figure S6. Leave-one-out analysis, MR effect size and funnel plot for PD on FA-BMD.

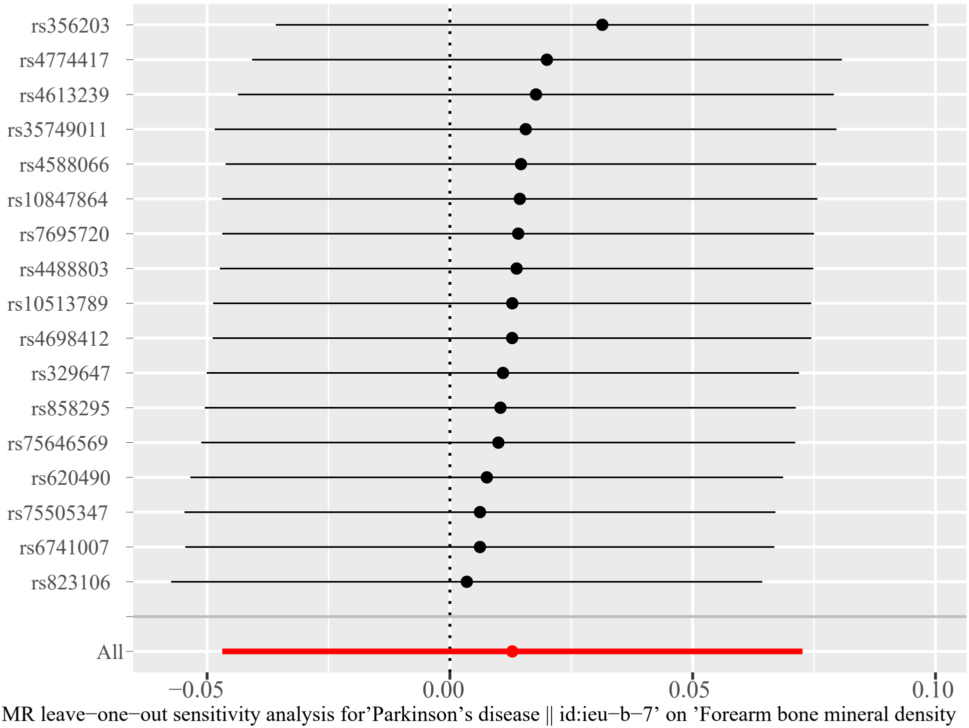

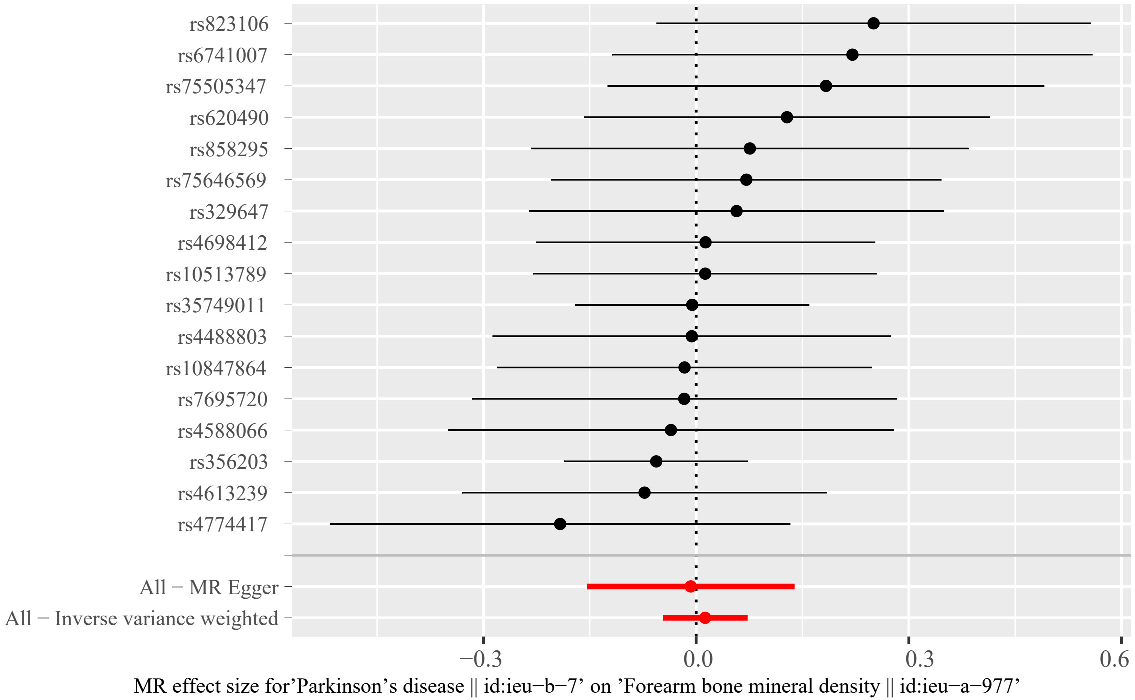
rs823106
rs6741007
rs75505347
rs620490
rs858295
rs75646569
rs329647
rs4698412
rs10513789
rs35749011
rs4488803
rs10847864
rs7695720
rs4588066
rs356203
rs4613239
rs4774417
All − MR Egger
All − Inverse variance weighted
−0.3
0.0
0.3
0.6
MR effect size for'Parkinson's disease || id:ieu−b−7' on 'Forearm bone mineral density || id:ieu−a−977'

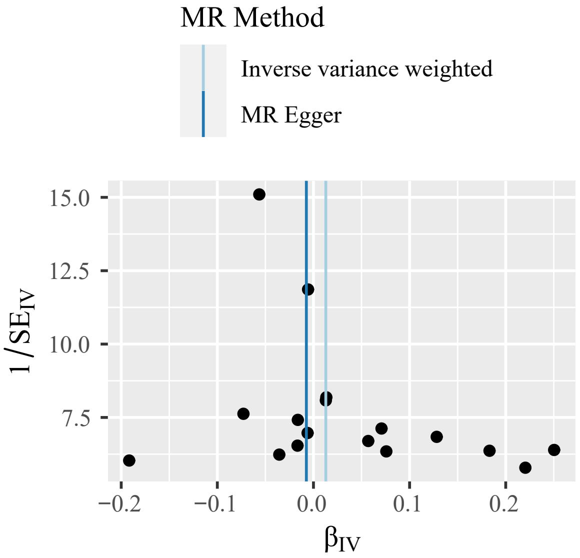
MR Method
Inverse variance weighted
MR Egger
15.0
12.5
10.0
7.5
1/SE_IV
−0.2
−0.1
0.0
0.1
0.2
β_IV

Figure S7. Leave-one-out analysis, MR effect size and funnel plot for PD on eBMD.

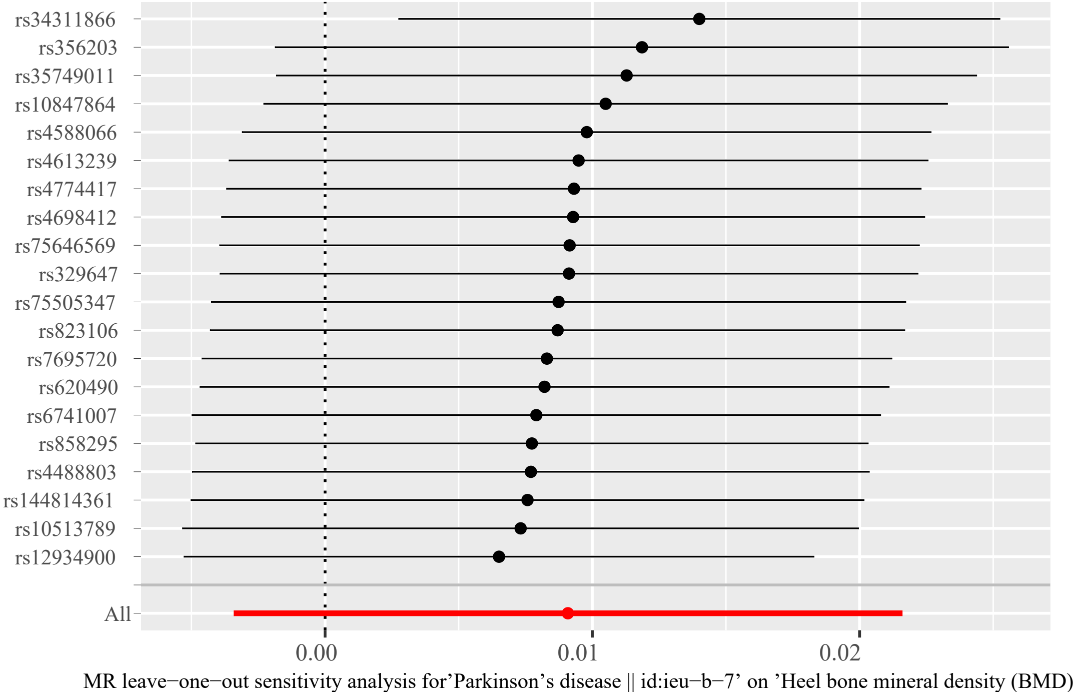

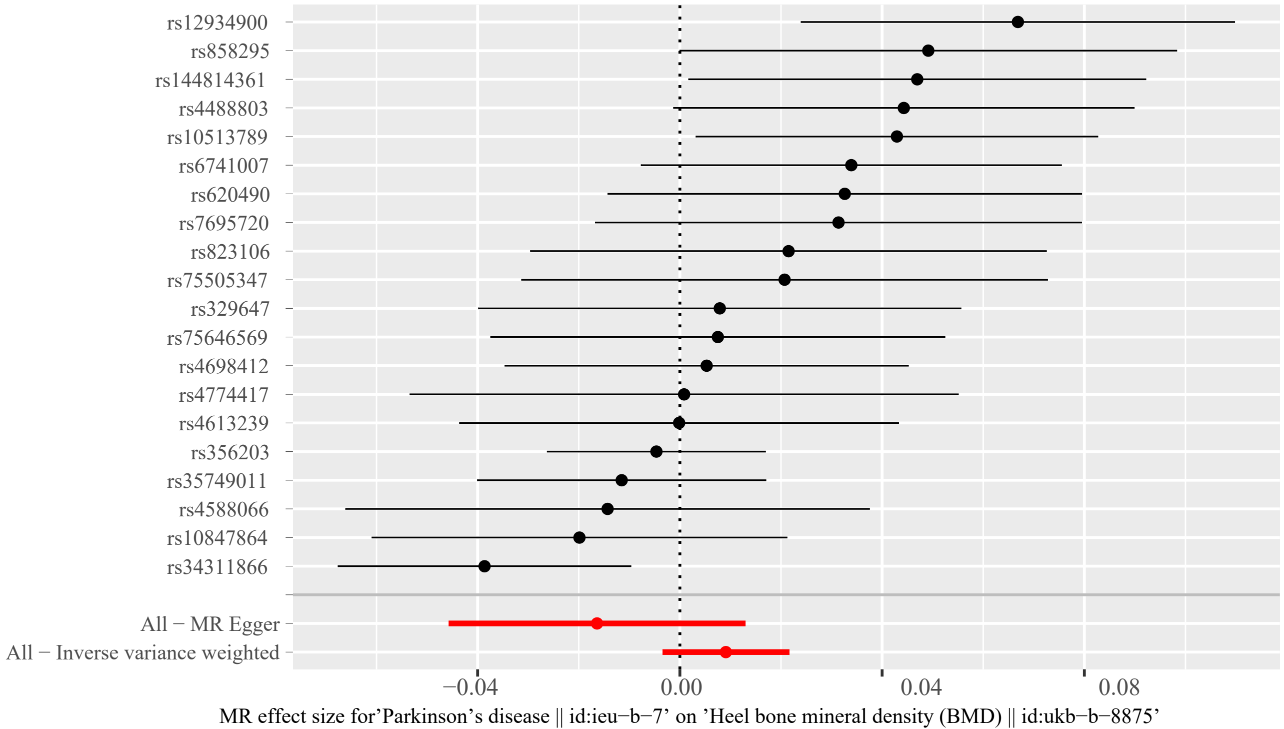
rs12934900
rs858295
rs144814361
rs4488803
rs10513789
rs6741007
rs620490
rs7695720
rs823106
rs75505347
rs329647
rs75646569
rs4698412
rs4774417
rs4613239
rs356203
rs35749011
rs4588066
rs10847864
rs34311866
All − MR Egger
All − Inverse variance weighted
−0.04
0.00
0.04
0.08
MR effect size for'Parkinson's disease || id:ieu−b−7' on 'Heel bone mineral density (BMD) || id:ukb−b−8875'

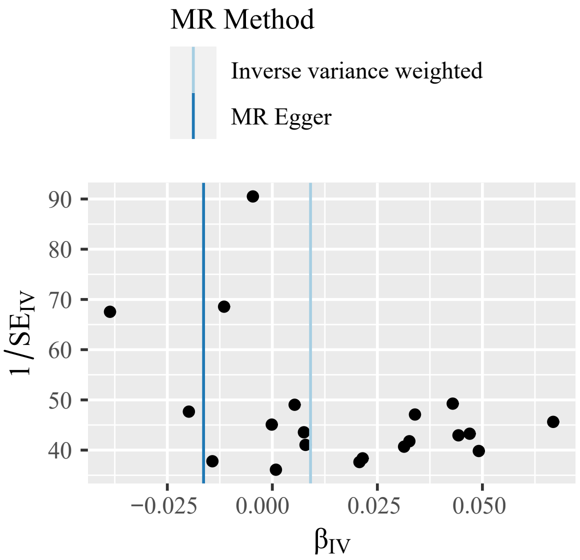
MR Method
Inverse variance weighted
MR Egger
90
80
70
60
50
40
$1/SE_{IV}$
−0.025
0.000
0.025
0.050
β_{IV}

Figure S8. Leave-one-out analysis, MR effect size and funnel plot for PD on LF.

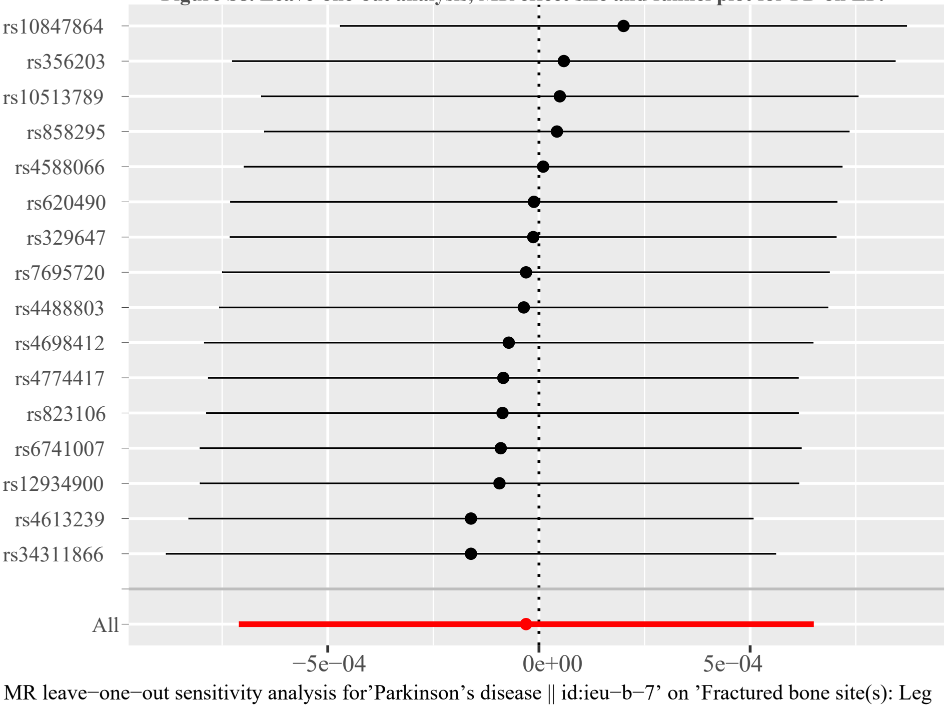

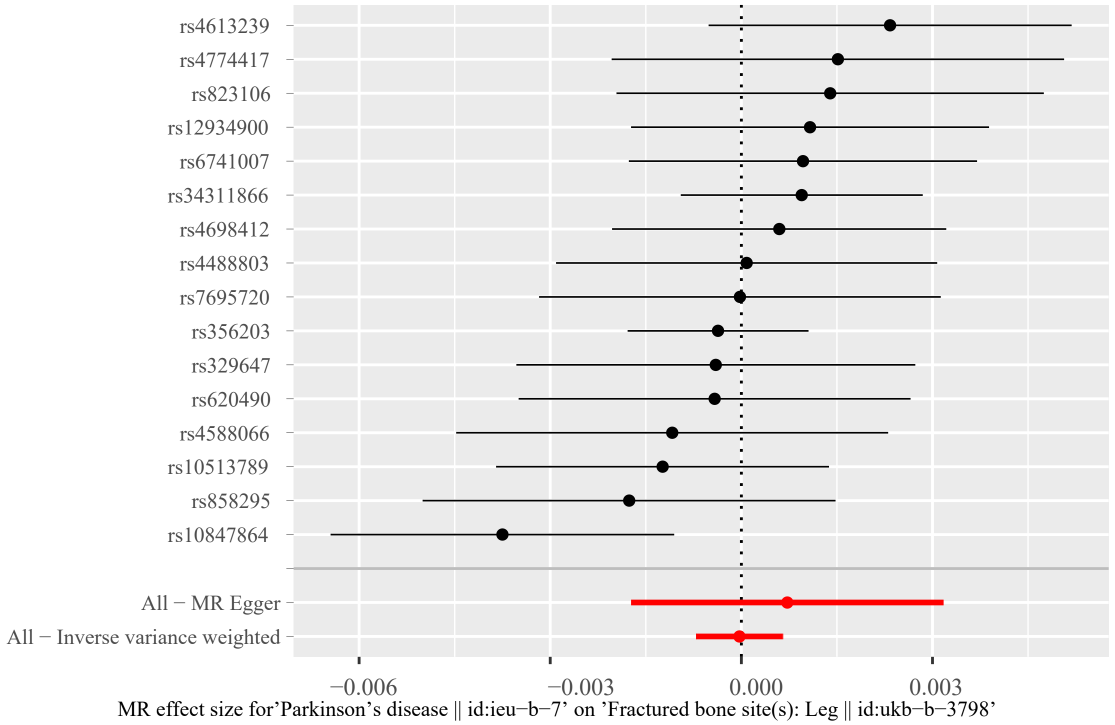
rs4613239
rs4774417
rs823106
rs12934900
rs6741007
rs34311866
rs4698412
rs4488803
rs7695720
rs356203
rs329647
rs620490
rs4588066
rs10513789
rs858295
rs10847864
All − MR Egger
All − Inverse variance weighted
−0.006
−0.003
0.000
0.003
MR effect size for'Parkinson's disease || id:ieu−b−7' on 'Fractured bone site(s): Leg || id:ukb−b−3798'

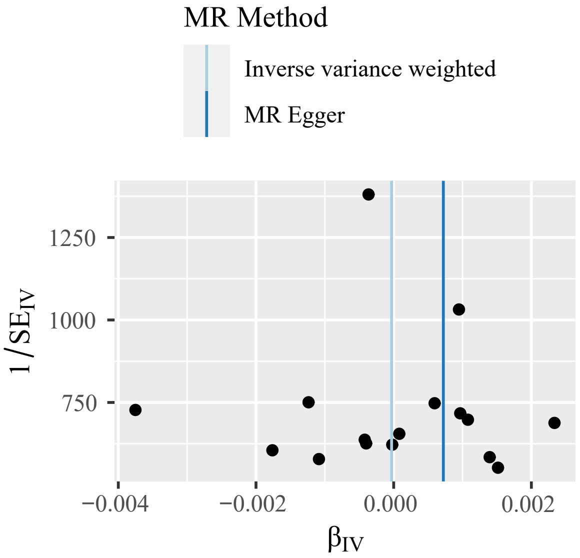

MR Method
Inverse variance weighted
MR Egger
1250
1000
750
1/SE_IV
−0.004
−0.002
0.000
0.002
β_IV

Figure S9. Leave-one-out analysis, MR effect size and funnel plot for PD on AF.

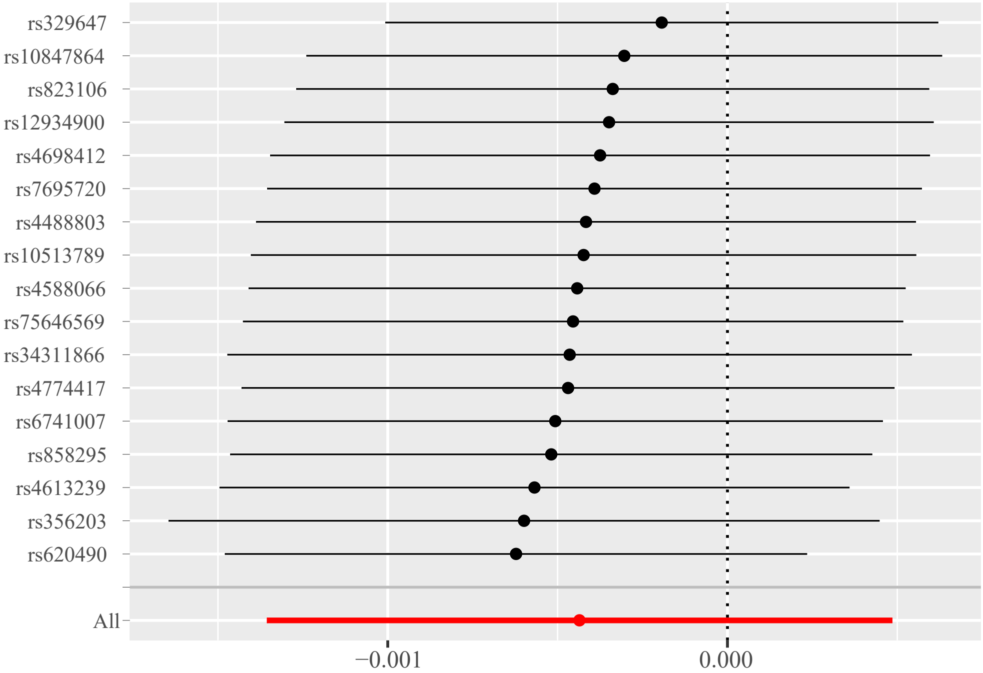

MR leave−one−out sensitivity analysis for'Parkinson's disease || id:ieu−b−7' on 'Fractured bone site(s): Arm

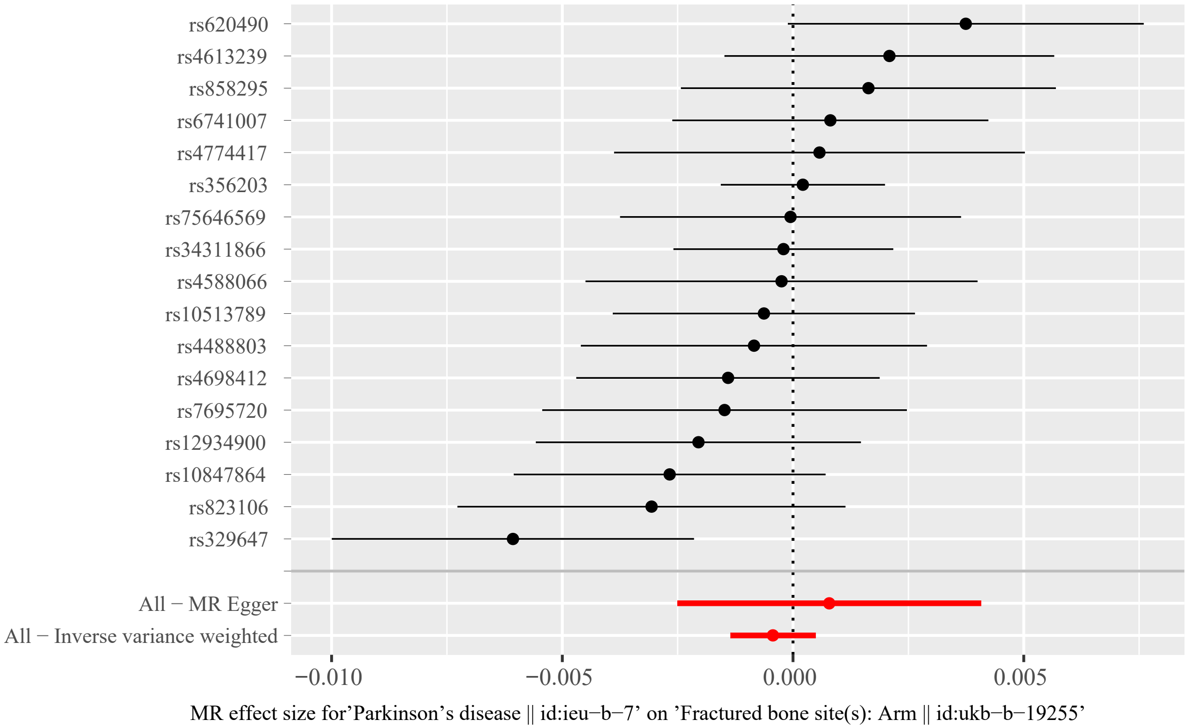
rs620490
rs4613239
rs858295
rs6741007
rs4774417
rs356203
rs75646569
rs34311866
rs4588066
rs10513789
rs4488803
rs4698412
rs7695720
rs12934900
rs10847864
rs823106
rs329647
All − MR Egger
All − Inverse variance weighted
−0.010
−0.005
0.000
0.005
MR effect size for'Parkinson's disease || id:ieu−b−7' on 'Fractured bone site(s): Arm || id:ukb−b−19255'

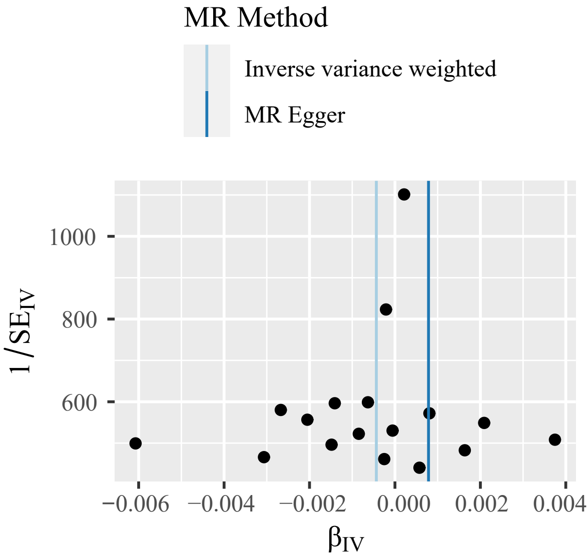
MR Method
Inverse variance weighted
MR Egger
1000
800
600
1/SE_IV
−0.006
−0.004
−0.002
0.000
0.002
0.004
β_IV

Figure S10. Leave-one-out analysis, MR effect size and funnel plot for PD on SF.

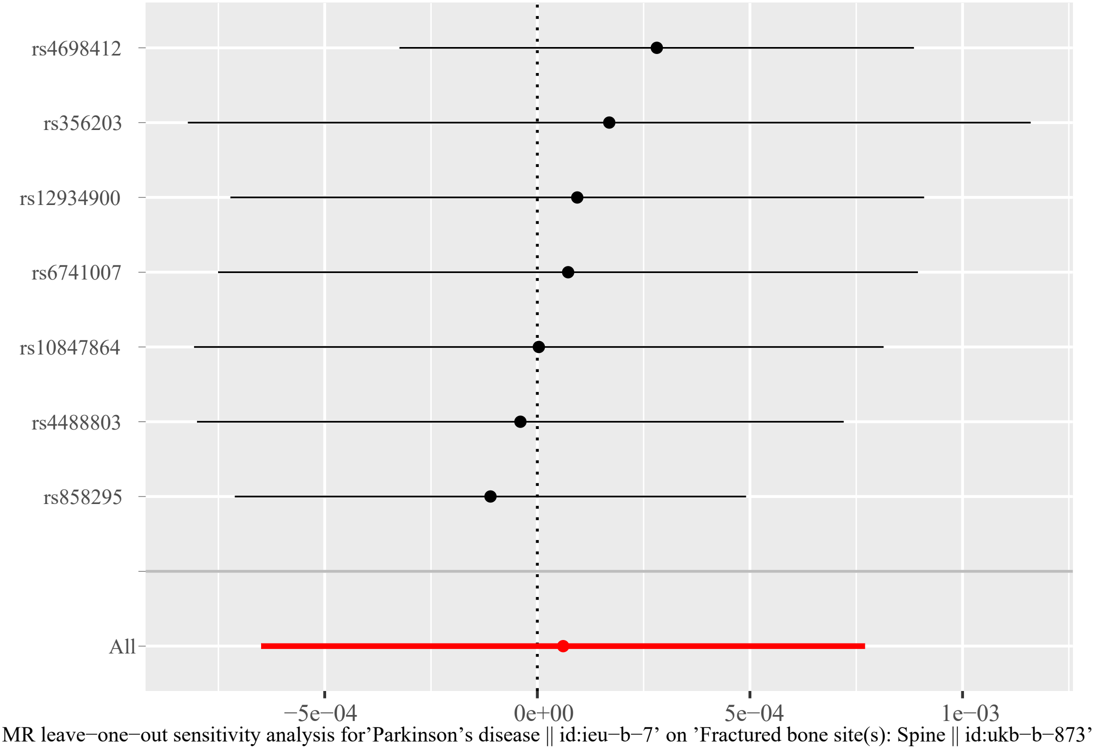

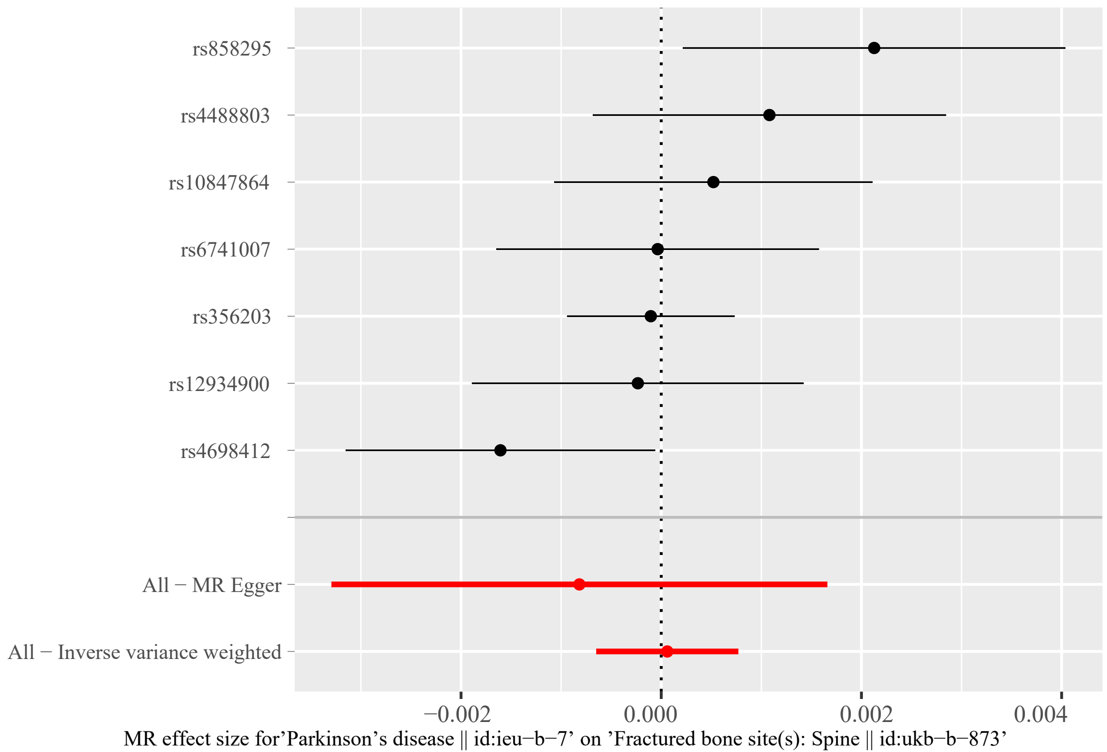

rs858295
rs4488803
rs10847864
rs6741007
rs356203
rs12934900
rs4698412
All − MR Egger
All − Inverse variance weighted
−0.002
0.000
0.002
0.004
MR effect size for’Parkinson’s disease || id:ieu−b−7’ on ’Fractured bone site(s): Spine || id:ukb−b−873’

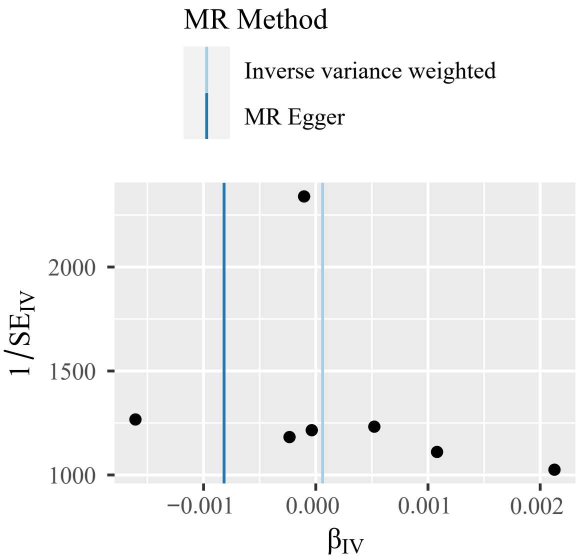

MR Method
Inverse variance weighted
MR Egger
2000
1500
1000
$1/SE_{IV}$
−0.001
0.000
0.001
0.002
β_{IV}

Figure S11. Leave-one-out analysis, MR effect size and funnel plot for PD on HF.

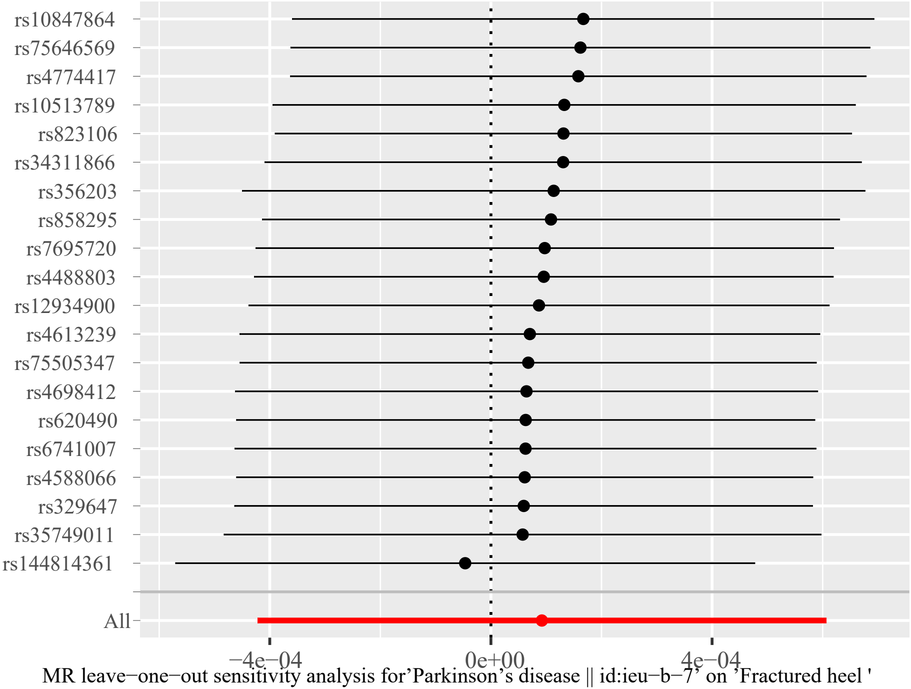

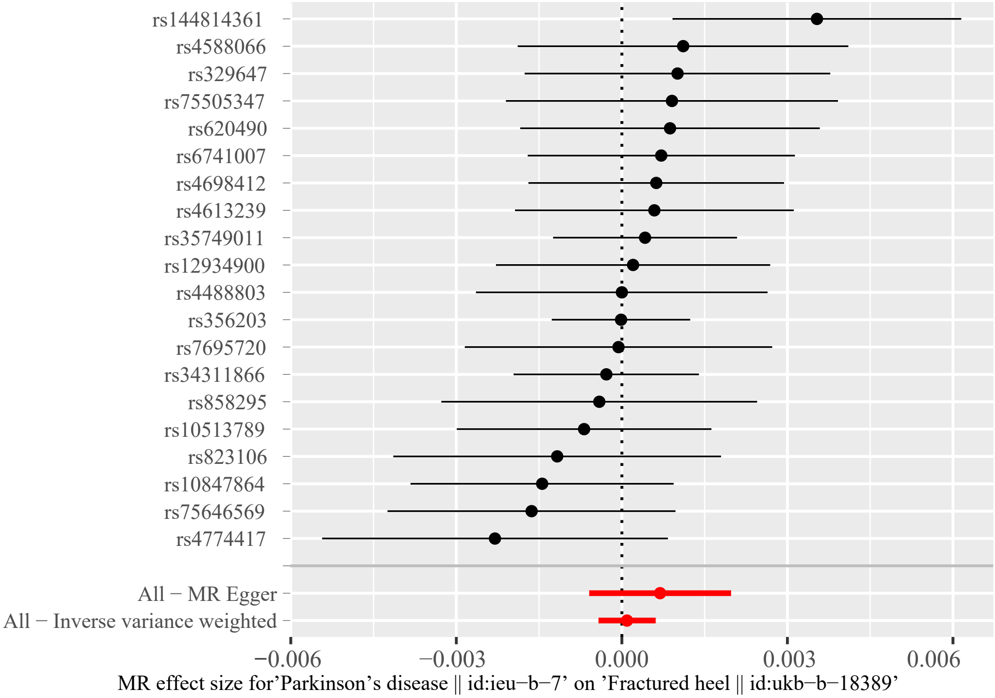

rs144814361
rs4588066
rs329647
rs75505347
rs620490
rs6741007
rs4698412
rs4613239
rs35749011
rs12934900
rs4488803
rs356203
rs7695720
rs34311866
rs858295
rs10513789
rs823106
rs10847864
rs75646569
rs4774417
All − MR Egger
All − Inverse variance weighted
−0.006
−0.003
0.000
0.003
0.006
MR effect size for'Parkinson's disease || id:ieu−b−7' on 'Fractured heel || id:ukb−b−18389'

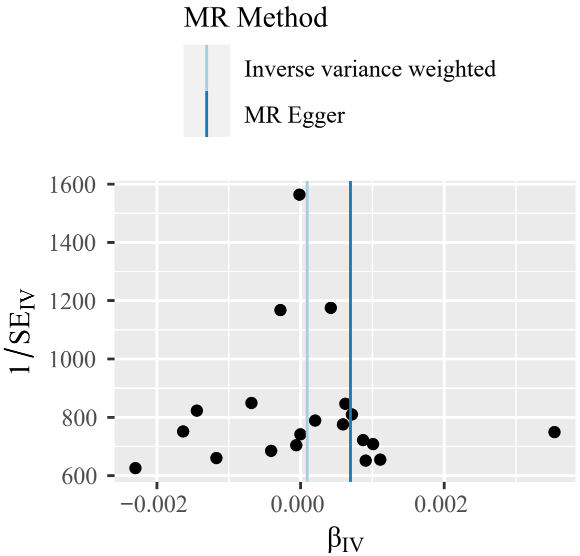

MR Method
Inverse variance weighted
MR Egger
1600
1400
1200
1000
800
600
−0.002
0.000
0.002
$1/SE_{IV}$
β_{IV}
