# Supplementary material for: Mendelian randomization analysis does not reveal a causal influence of mental diseases on osteoporosis
Source: Front Endocrinol (Lausanne). 2023 Apr 20;14:1125427. doi: 10.3389/fendo.2023.1125427 (PMC10157183; doi:10.3389/fendo.2023.1125427)
Supplement: Supplementary Material 1 — Instrumental variables SNPs. [file DataSheet_1.zip › Supplementary Material/Supplementary Material 7.pdf]

Figure S1. Leave-one-out analysis, MR effect size and funnel plot for EP on OP.

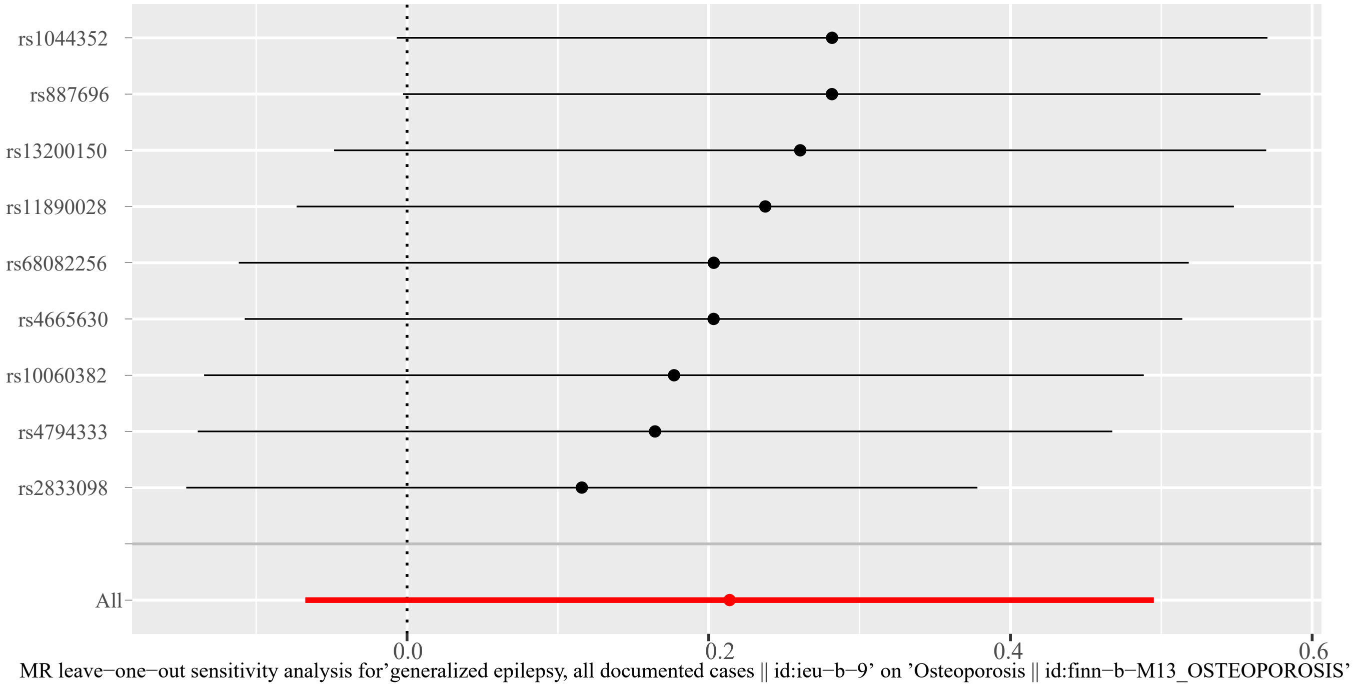

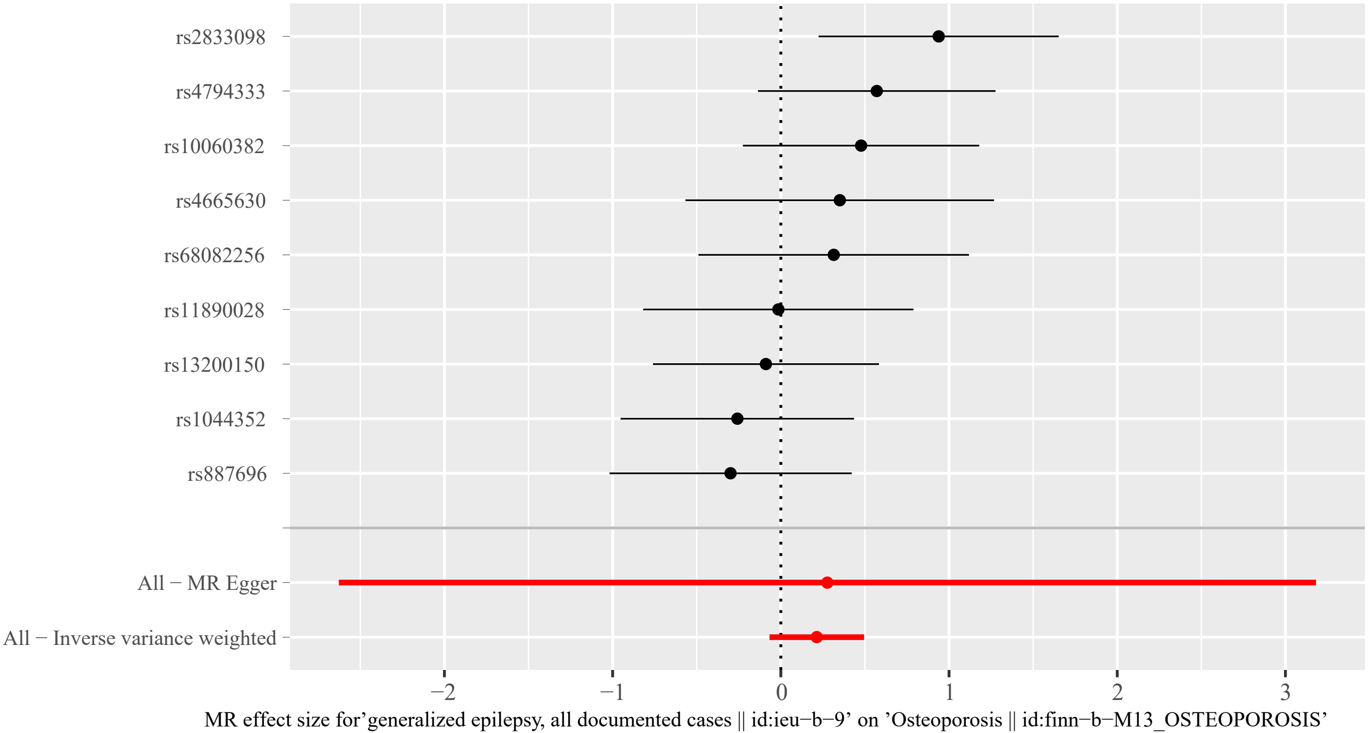
rs2833098
rs4794333
rs10060382
rs4665630
rs68082256
rs11890028
rs13200150
rs1044352
rs887696
All − MR Egger
All − Inverse variance weighted
−2
−1
0
1
2
3
MR effect size for'generalized epilepsy, all documented cases || id:ieu−b−9' on 'Osteoporosis || id:finn−b−M13_OSTEOPOROSIS'

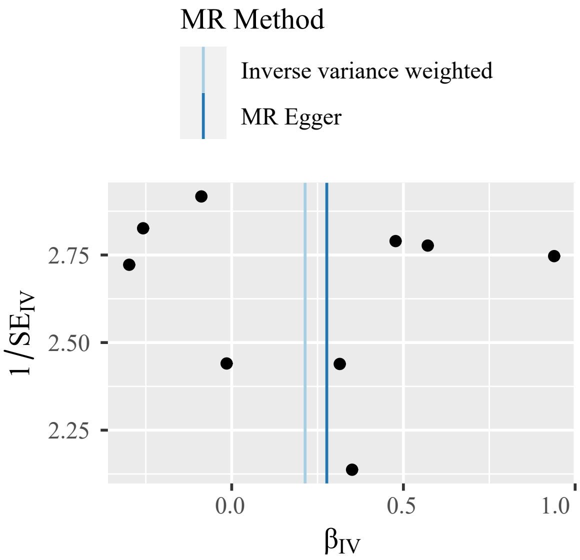

MR Method
Inverse variance weighted
MR Egger
2.75
2.50
2.25
$1/SE_{IV}$
0.0
0.5
1.0
β_{IV}

Figure S2. Leave-one-out analysis, MR effect size and funnel plot for EP on OPF.

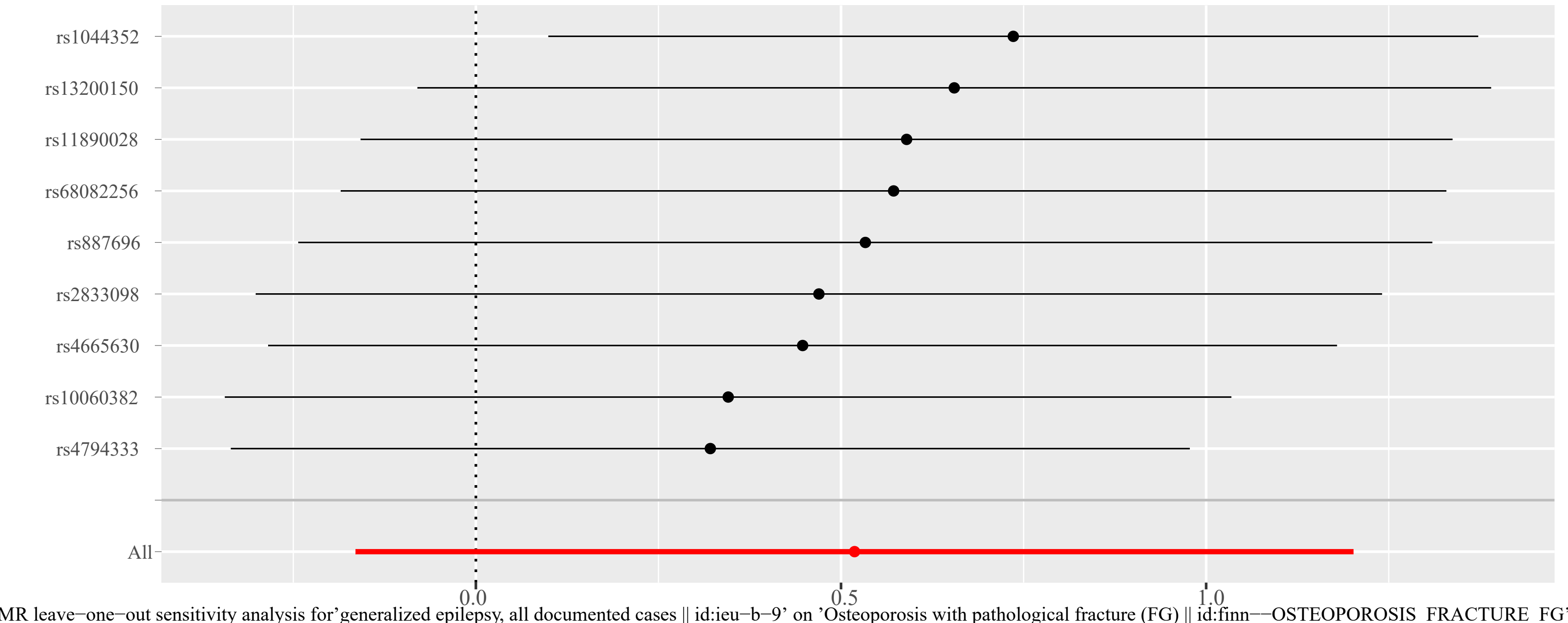

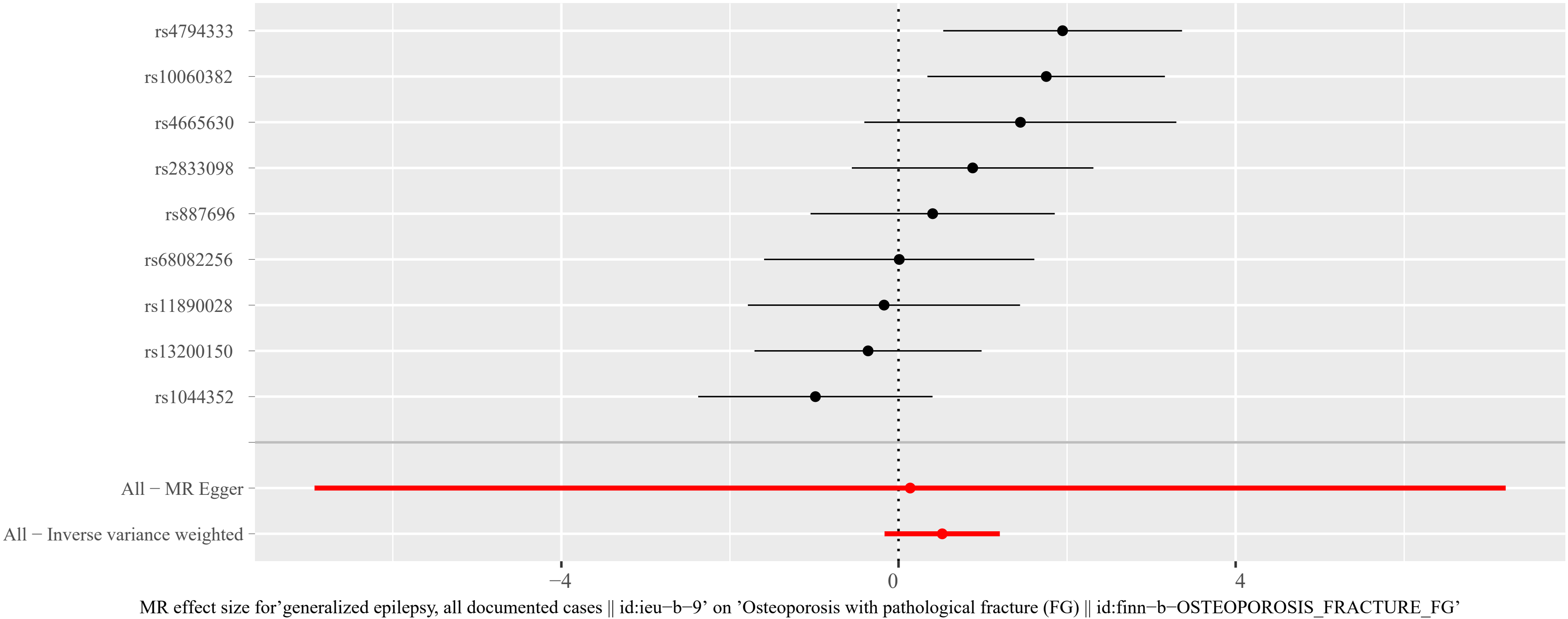
rs4794333
rs10060382
rs4665630
rs2833098
rs887696
rs68082256
rs11890028
rs13200150
rs1044352
All − MR Egger
All − Inverse variance weighted
−4
0
4
MR effect size for'generalized epilepsy, all documented cases || id:ieu−b−9' on 'Osteoporosis with pathological fracture (FG) || id:finn−b−OSTEOPOROSIS_FRACTURE_FG'

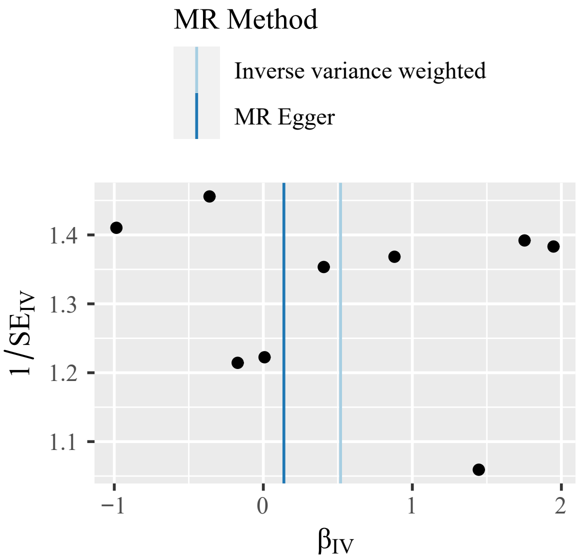
MR Method
Inverse variance weighted
MR Egger
1.4
1.3
1.2
1.1
−1
0
1
2
$1/SE_{IV}$
β_{IV}

Figure S3. Leave-one-out analysis, MR effect size and funnel plot for EP on TB-BMD.

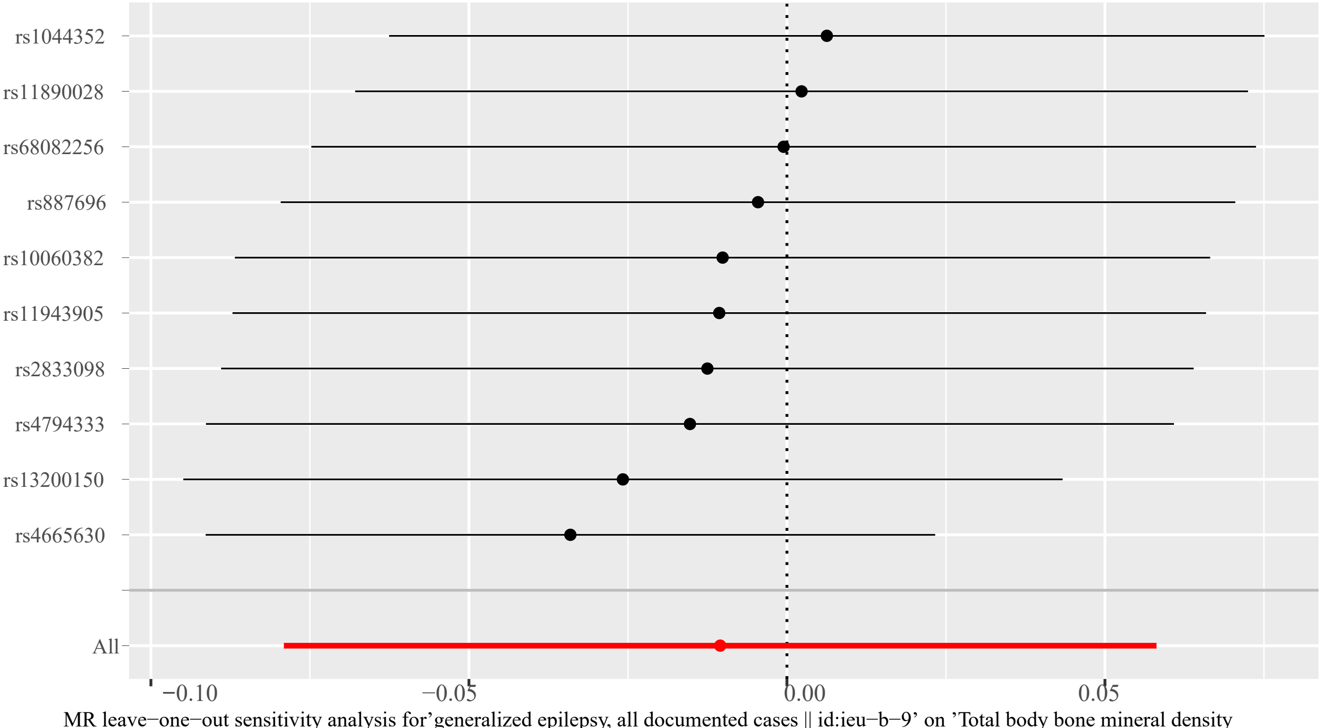

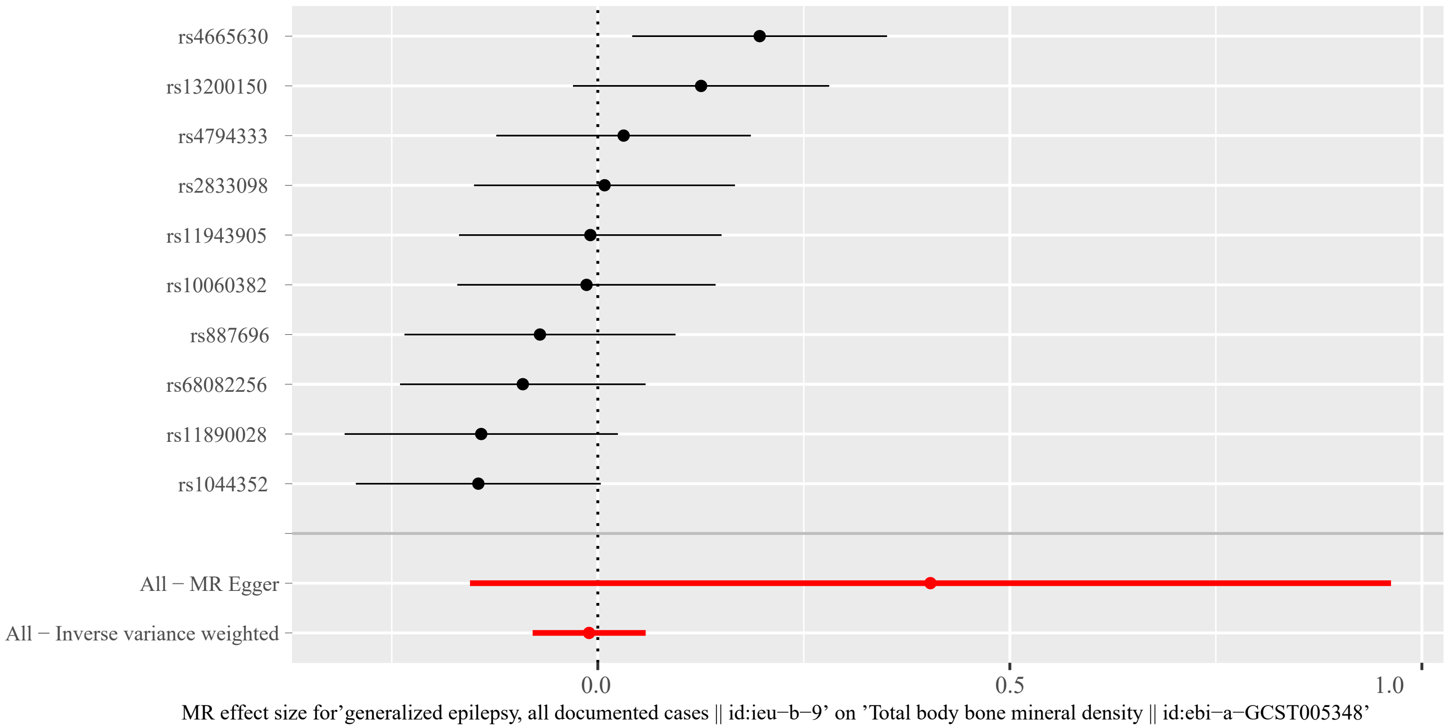
rs4665630
rs13200150
rs4794333
rs2833098
rs11943905
rs10060382
rs887696
rs68082256
rs11890028
rs1044352
All − MR Egger
All − Inverse variance weighted
0.0
0.5
1.0
MR effect size for'generalized epilepsy, all documented cases || id:ieu−b−9' on 'Total body bone mineral density || id:ebi−a−GCST005348'

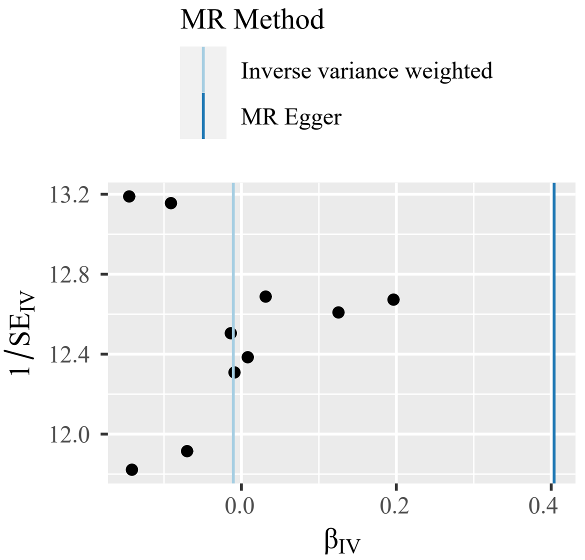

MR Method
Inverse variance weighted
MR Egger
13.2
12.8
12.4
12.0
$1/SE_{IV}$
0.0
0.2
0.4
β_{IV}

Figure S4. Leave-one-out analysis, MR effect size and funnel plot for EP on FN-BMD.

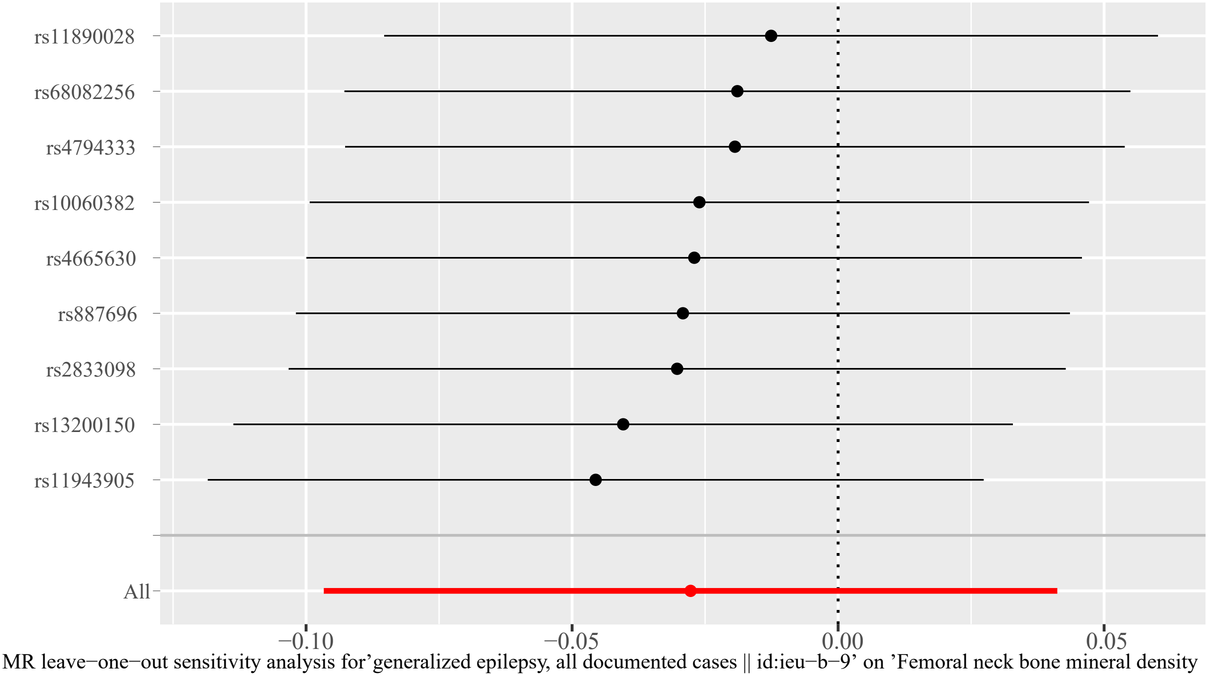

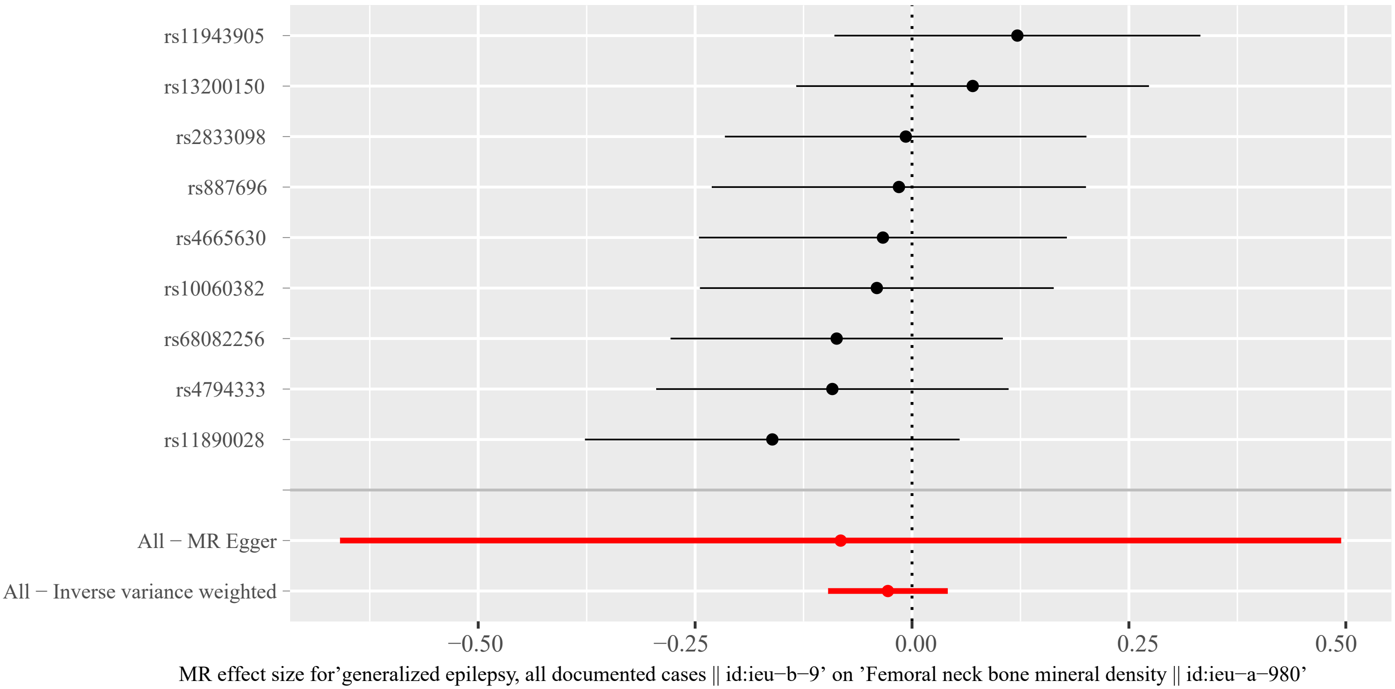

rs11943905
rs13200150
rs2833098
rs887696
rs4665630
rs10060382
rs68082256
rs4794333
rs11890028
All − MR Egger
All − Inverse variance weighted
−0.50
−0.25
0.00
0.25
0.50
MR effect size for'generalized epilepsy, all documented cases || id:ieu−b−9' on 'Femoral neck bone mineral density || id:ieu−a−980'

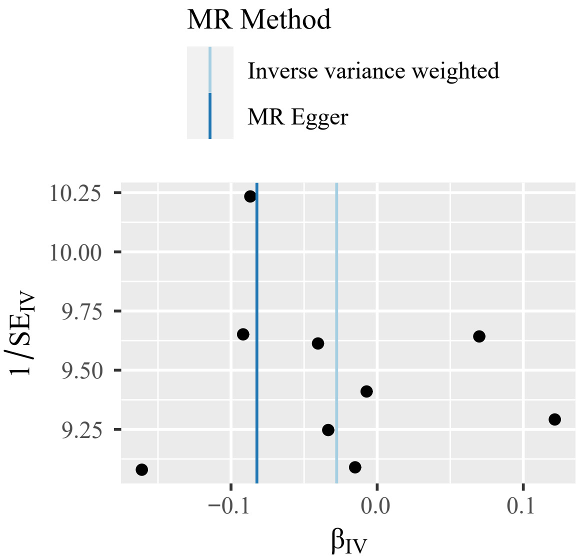
MR Method
Inverse variance weighted
MR Egger
10.25
10.00
9.75
9.50
9.25
−0.1
0.0
0.1
1/SE_IV
β_IV

Figure S5. Leave-one-out analysis, MR effect size and funnel plot for EP on LS-BMD.

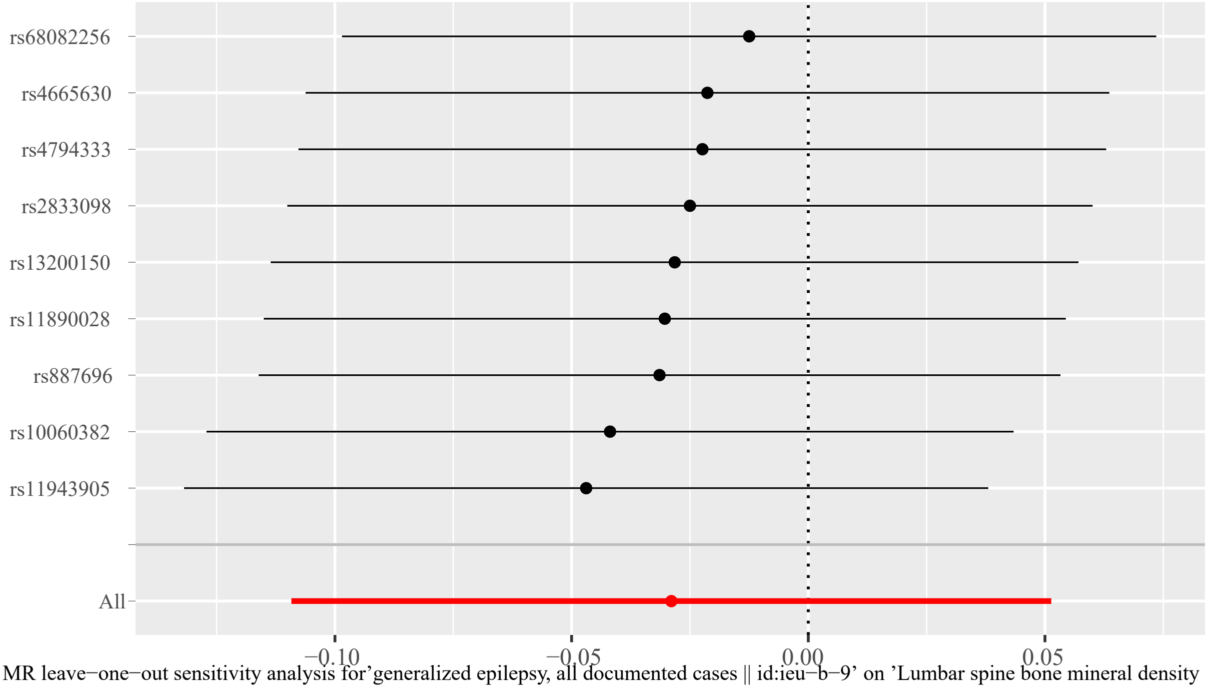

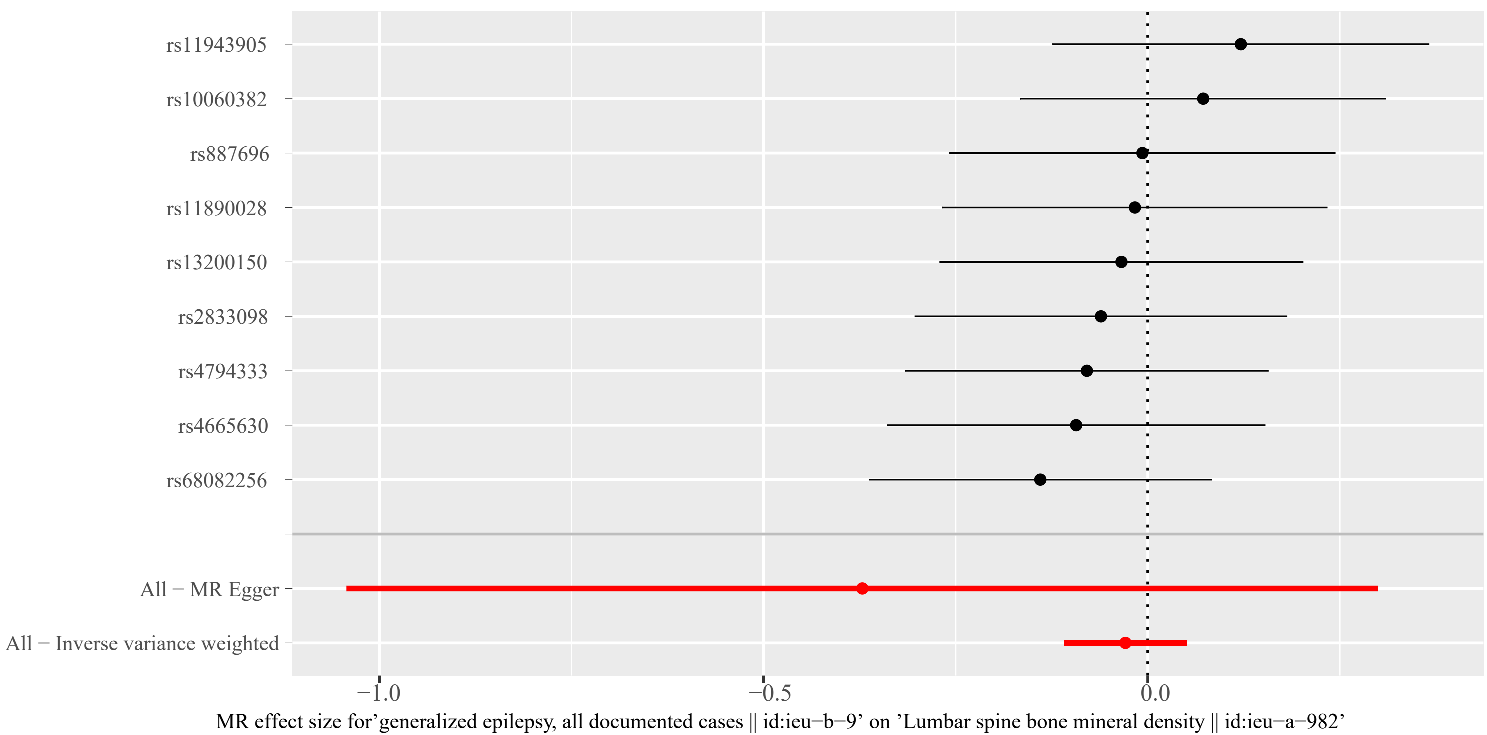
rs11943905
rs10060382
rs887696
rs11890028
rs13200150
rs2833098
rs4794333
rs4665630
rs68082256
All − MR Egger
All − Inverse variance weighted
−1.0
−0.5
0.0
MR effect size for'generalized epilepsy, all documented cases || id:ieu−b−9' on 'Lumbar spine bone mineral density || id:ieu−a−982'

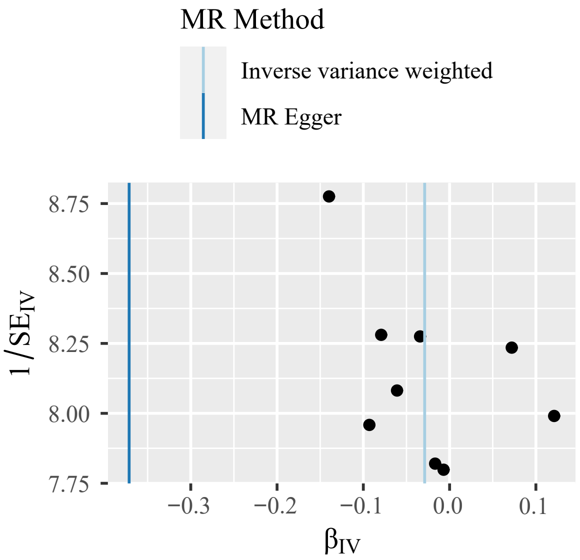

MR Method
Inverse variance weighted
MR Egger
8.75
8.50
8.25
8.00
7.75
1/SE_IV
−0.3
−0.2
−0.1
0.0
0.1
β_IV

Figure S6. Leave-one-out analysis, MR effect size and funnel plot for EP on FA-BMD.

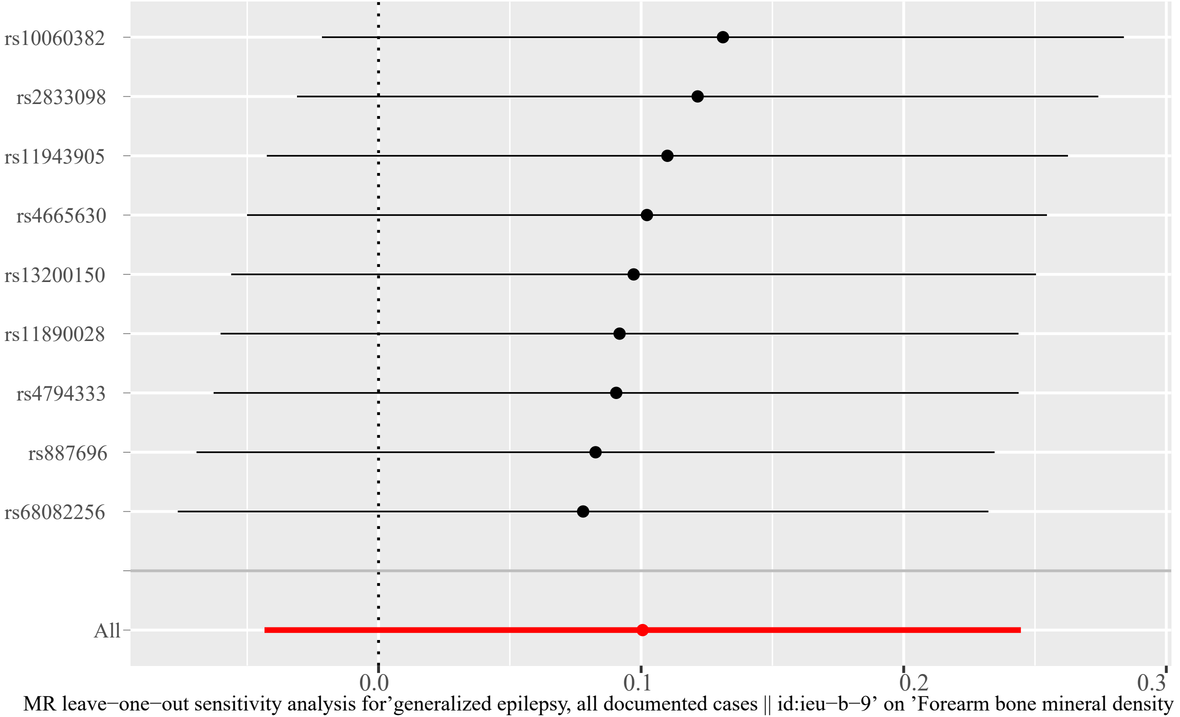

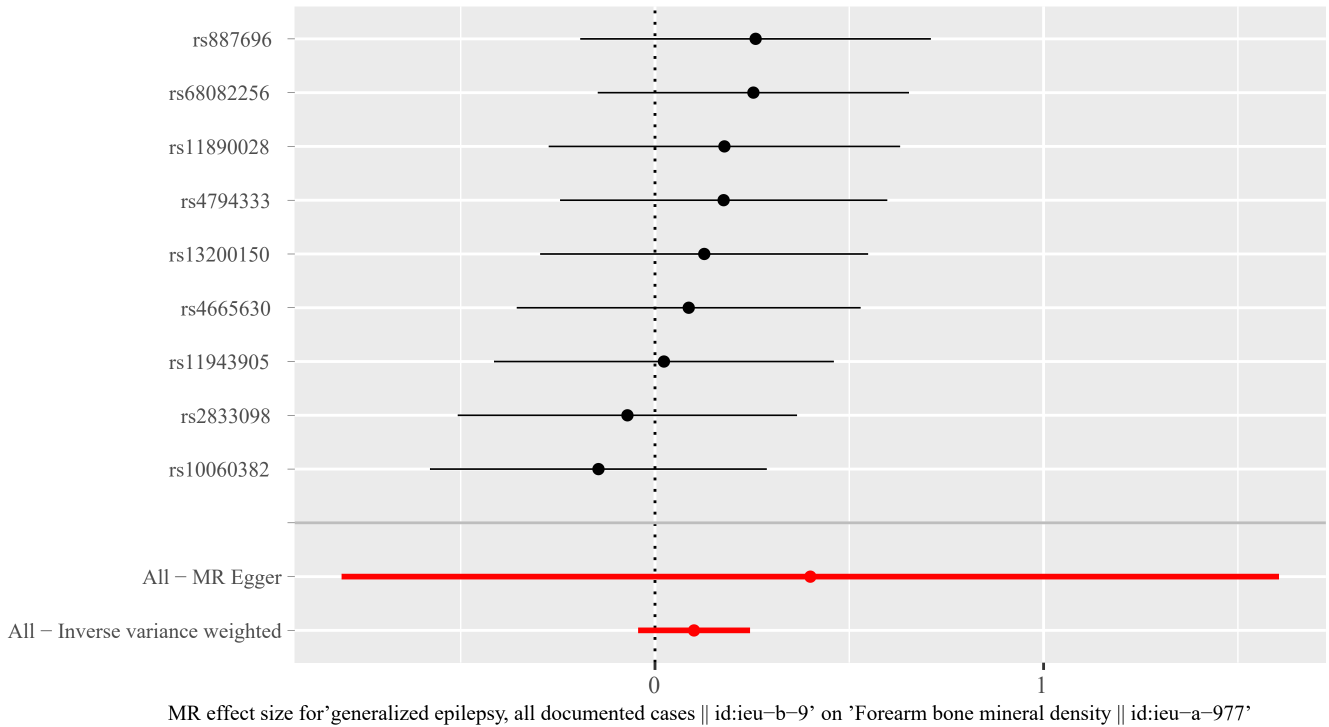
rs887696
rs68082256
rs11890028
rs4794333
rs13200150
rs4665630
rs11943905
rs2833098
rs10060382
All − MR Egger
All − Inverse variance weighted
0
1
MR effect size for'generalized epilepsy, all documented cases || id:ieu−b−9' on 'Forearm bone mineral density || id:ieu−a−977'

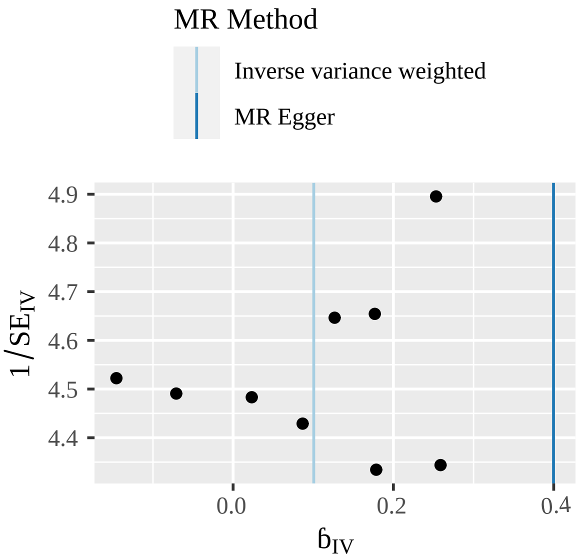

MR Method
Inverse variance weighted
MR Egger
4.9
4.8
4.7
4.6
4.5
4.4
$1/SE_{IV}$
0.0
0.2
0.4
$β_{IV}$

Figure S7. Leave-one-out analysis, MR effect size and funnel plot for EP on eBMD.

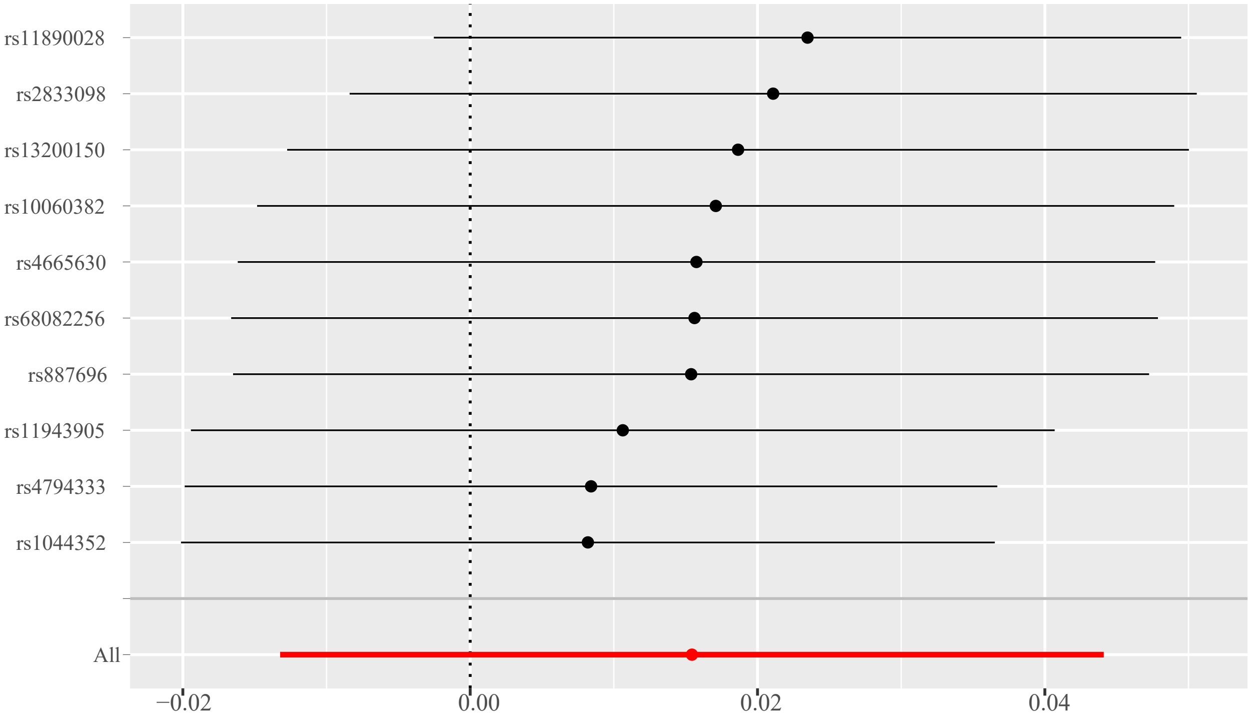

MR leave−one−out sensitivity analysis for’generalized epilepsy, all documented cases || id:ieu−b−9’ on ’Heel bone mineral density (BMD)

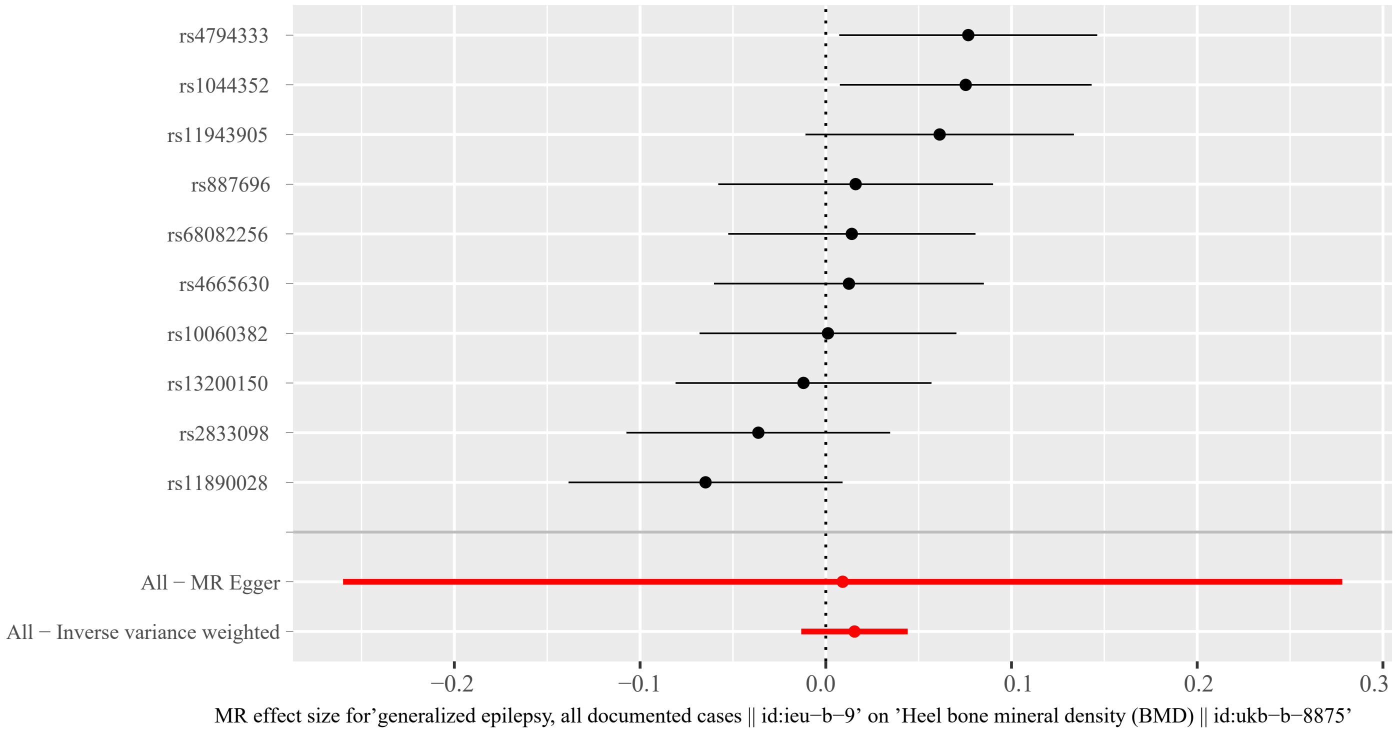
rs4794333
rs1044352
rs11943905
rs887696
rs68082256
rs4665630
rs10060382
rs13200150
rs2833098
rs11890028
All − MR Egger
All − Inverse variance weighted
−0.2
−0.1
0.0
0.1
0.2
0.3
MR effect size for'generalized epilepsy, all documented cases || id:ieu−b−9' on 'Heel bone mineral density (BMD) || id:ukb−b−8875'

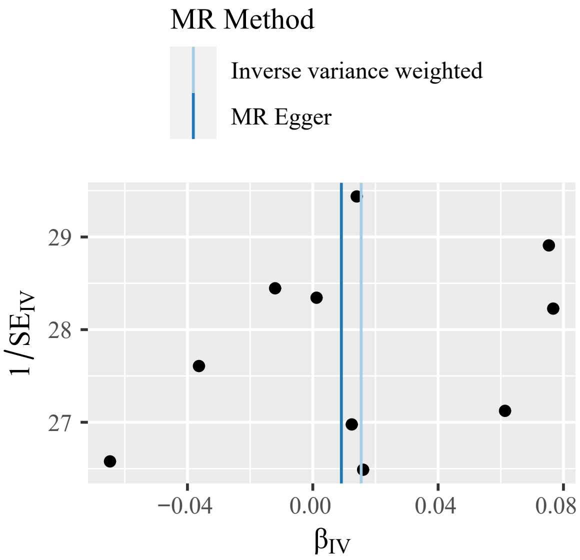

MR Method
Inverse variance weighted
MR Egger
29
28
27
1/SE_IV
−0.04
0.00
0.04
0.08
β_IV

Figure S8. Leave-one-out analysis, MR effect size and funnel plot for EP on LF.

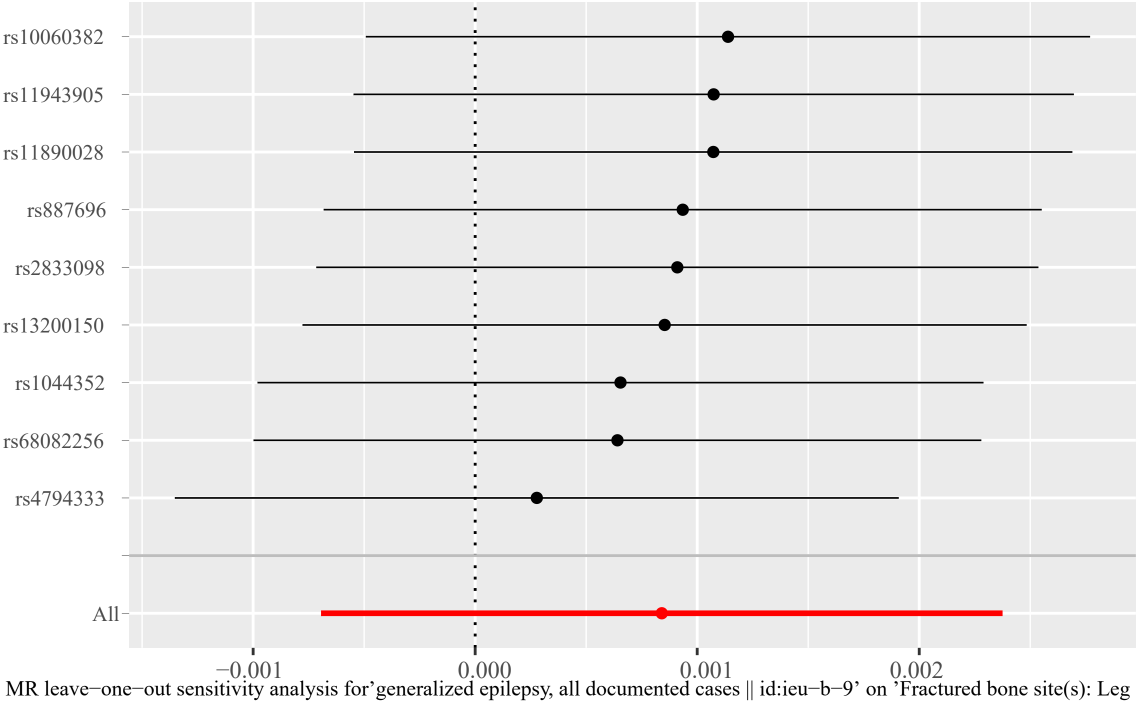

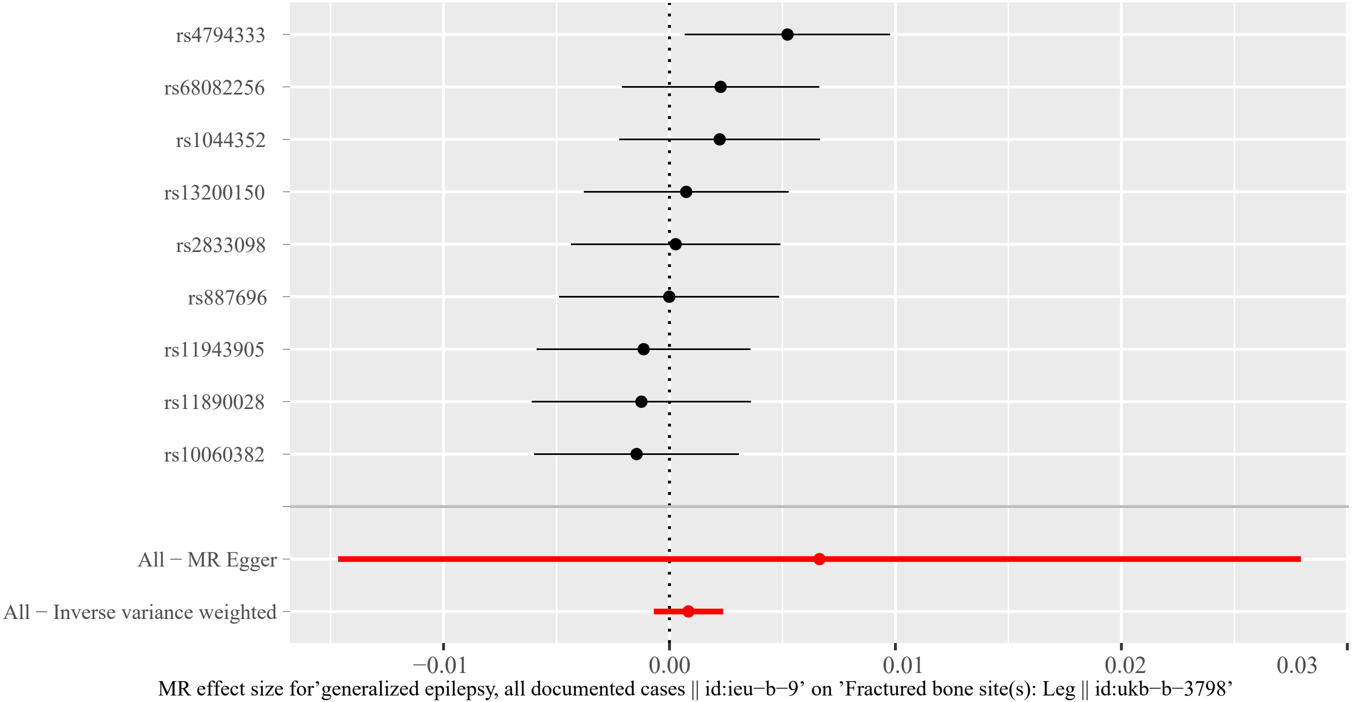
rs4794333
rs68082256
rs1044352
rs13200150
rs2833098
rs887696
rs11943905
rs11890028
rs10060382
All − MR Egger
All − Inverse variance weighted
−0.01
0.00
0.01
0.02
0.03
MR effect size for'generalized epilepsy, all documented cases || id:ieu−b−9' on 'Fractured bone site(s): Leg || id:ukb−b−3798'

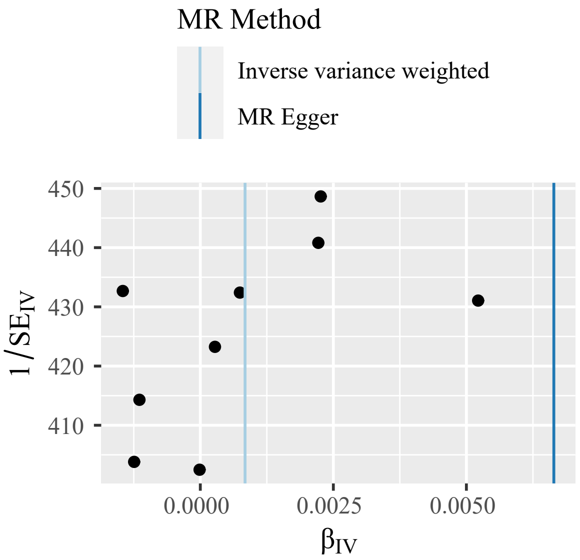
MR Method
Inverse variance weighted
MR Egger
450
440
430
420
410
0.0000
0.0025
0.0050
1/SE_IV
β_IV

Figure S9. Leave-one-out analysis, MR effect size and funnel plot for EP on AF.

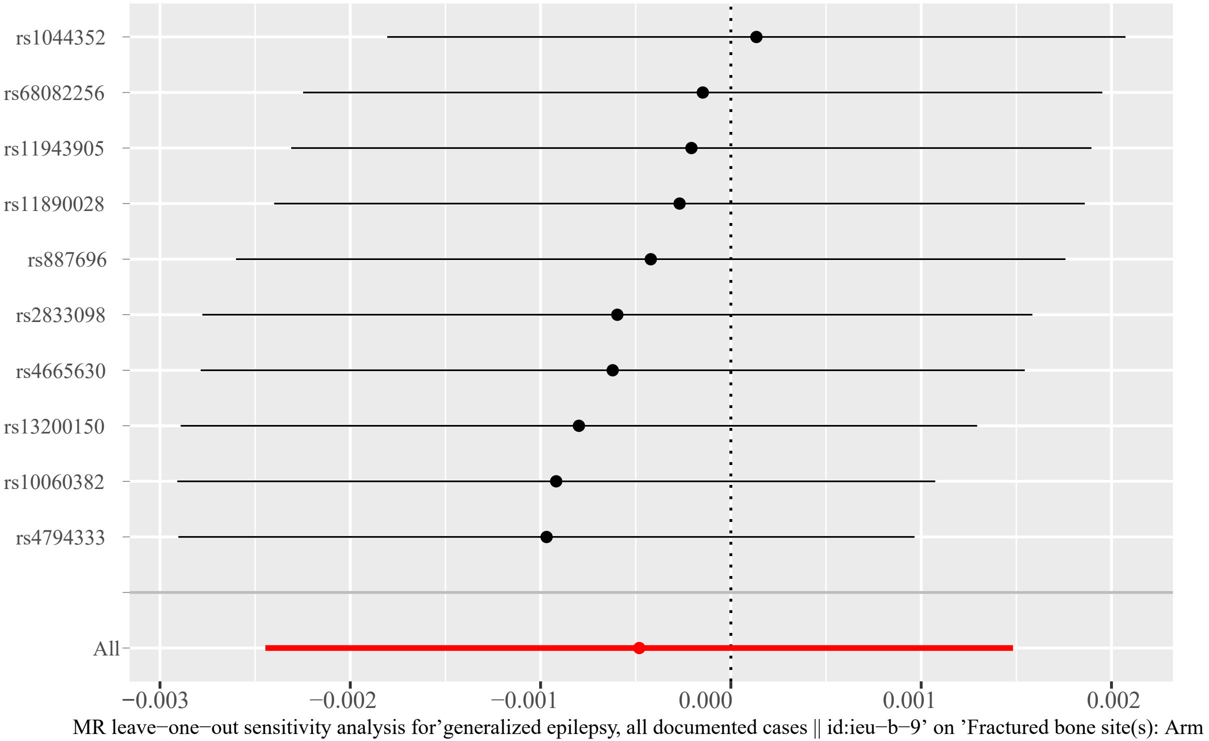

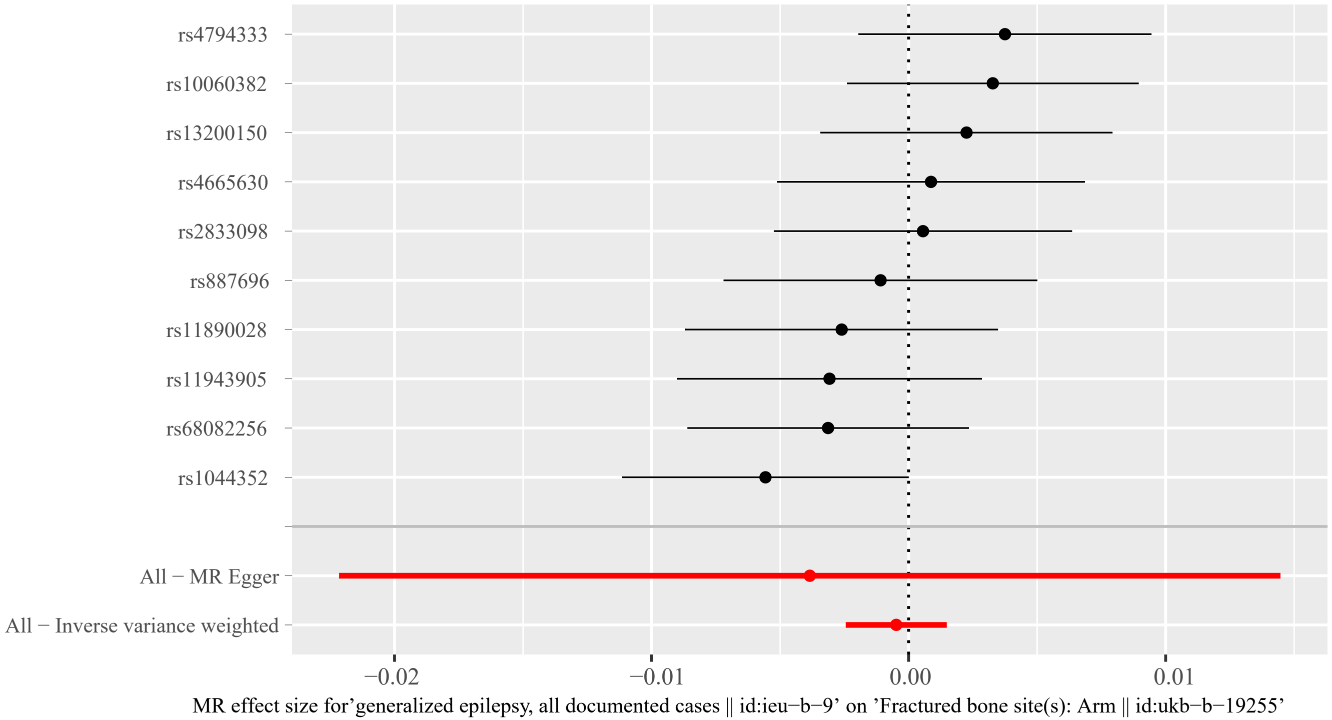

rs4794333
rs10060382
rs13200150
rs4665630
rs2833098
rs887696
rs11890028
rs11943905
rs68082256
rs1044352
All − MR Egger
All − Inverse variance weighted
−0.02
−0.01
0.00
0.01
MR effect size for'generalized epilepsy, all documented cases || id:ieu−b−9' on 'Fractured bone site(s): Arm || id:ukb−b−19255'

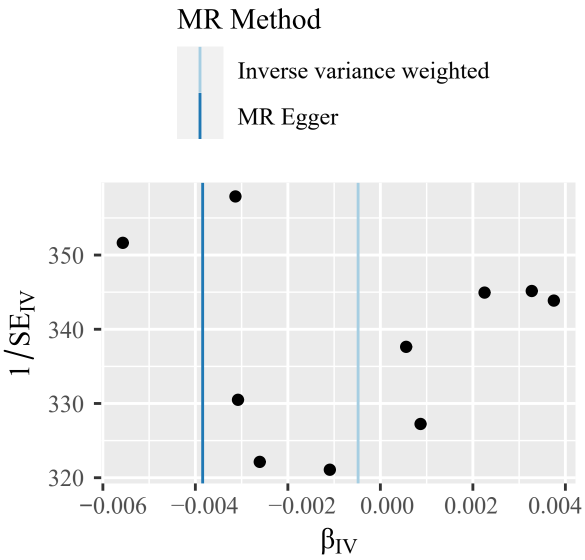

MR Method
Inverse variance weighted
MR Egger
350
340
330
320
1/SE_IV
−0.006
−0.004
−0.002
0.000
0.002
0.004
β_IV

Figure S10. Leave-one-out analysis, MR effect size and funnel plot for EP on HF.

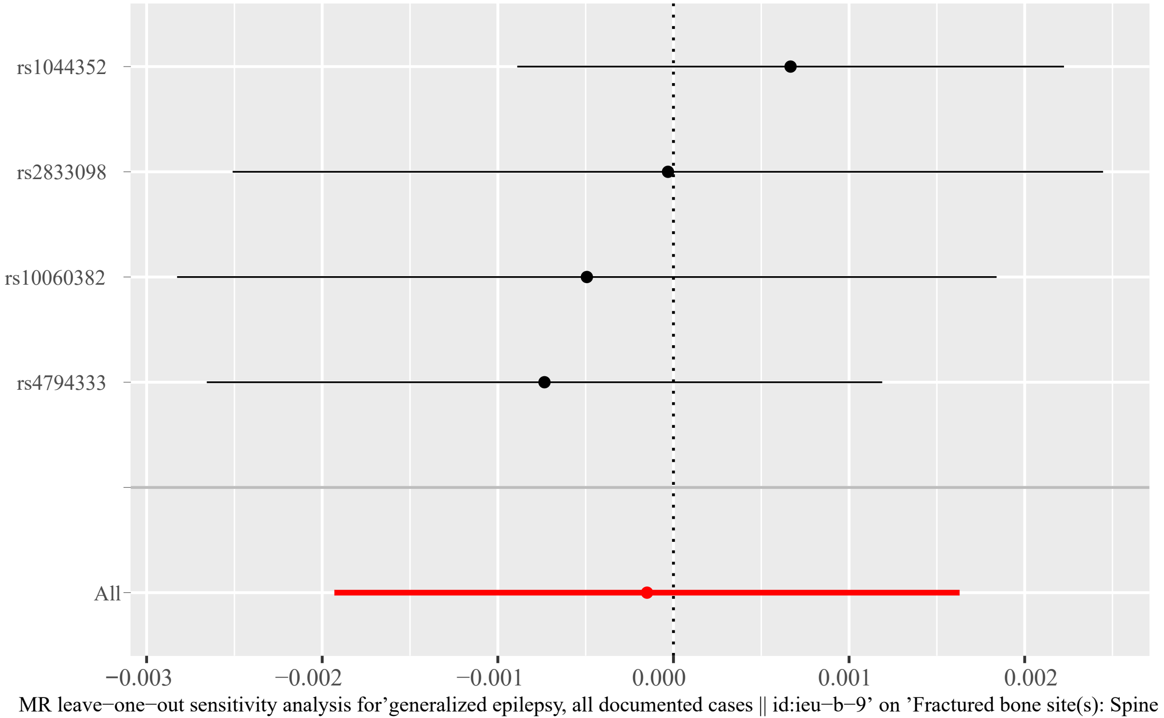

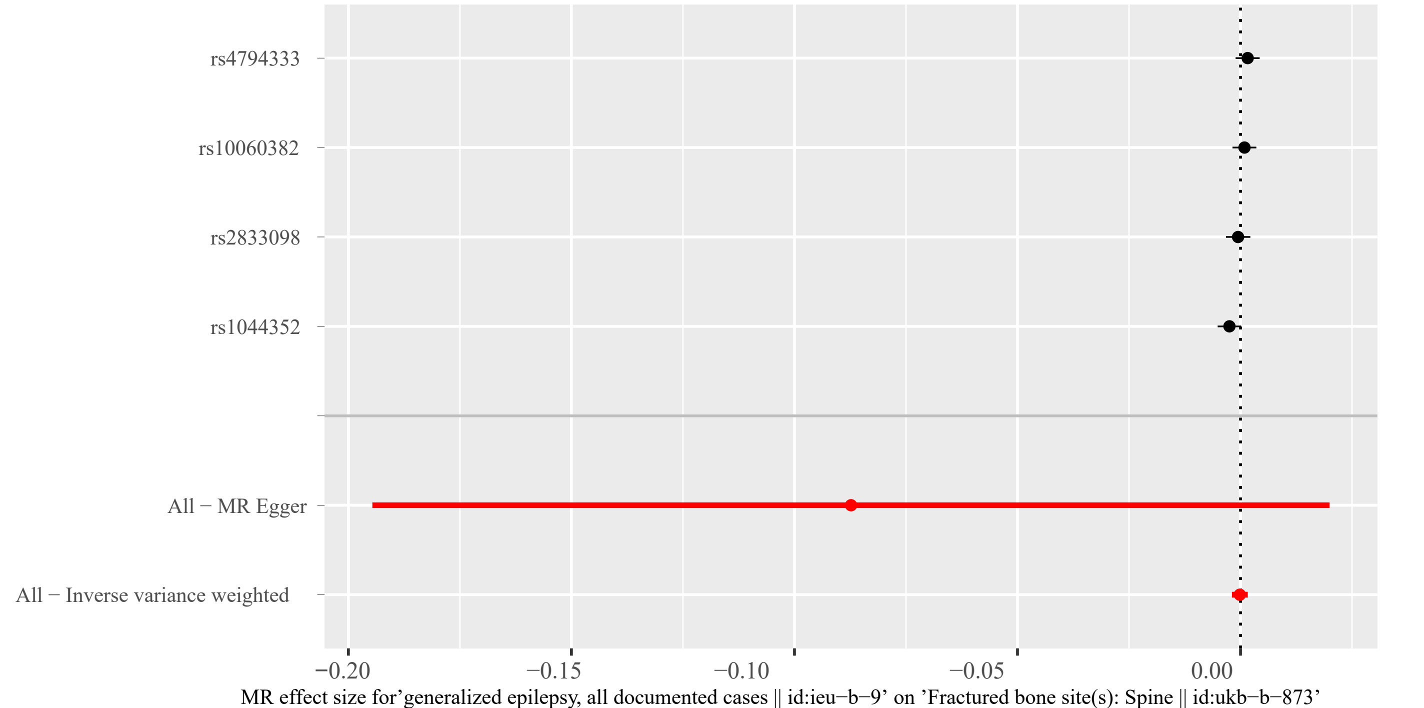
rs4794333
rs10060382
rs2833098
rs1044352
All − MR Egger
All − Inverse variance weighted
−0.20
−0.15
−0.10
−0.05
0.00
MR effect size for'generalized epilepsy, all documented cases || id:ieu−b−9' on 'Fractured bone site(s): Spine || id:ukb−b−873'

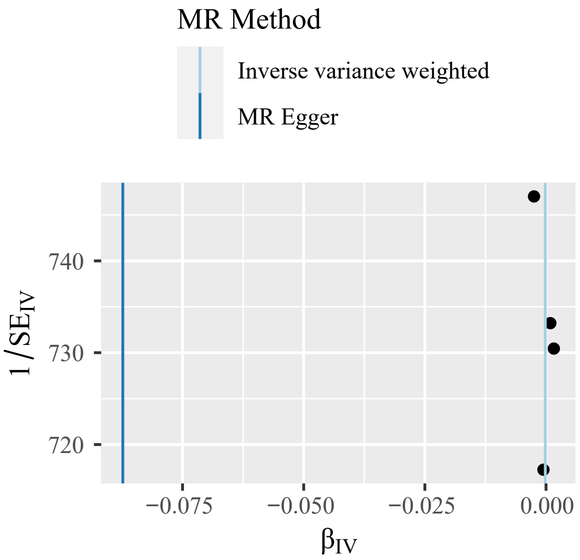
MR Method
Inverse variance weighted
MR Egger
740
730
720
1/SE_IV
−0.075
−0.050
−0.025
0.000
β_IV

Figure S11. Leave-one-out analysis, MR effect size and funnel plot for EP on HF.

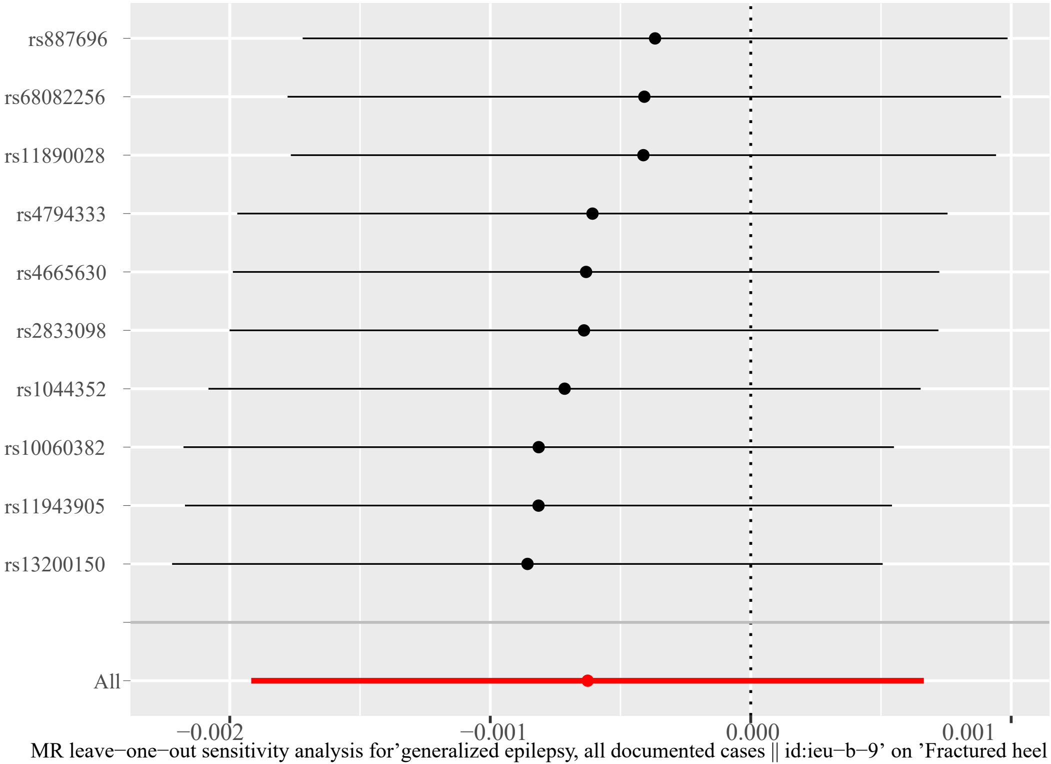

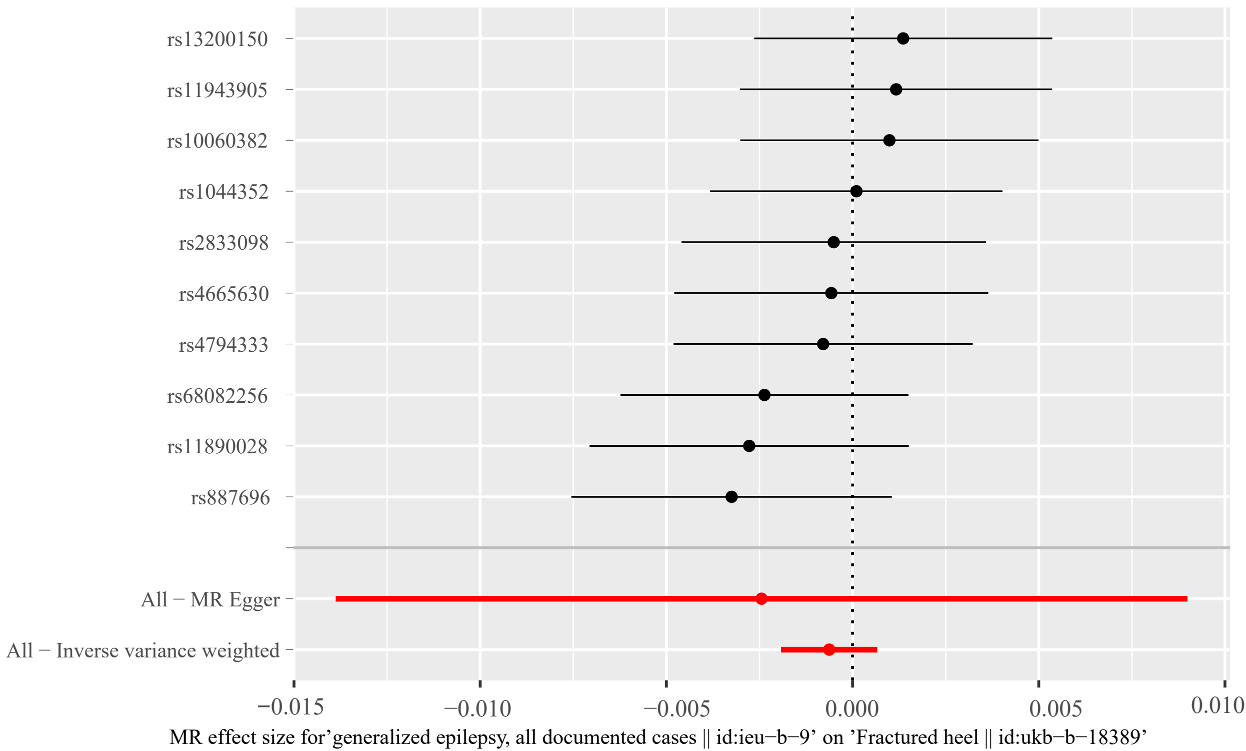
rs13200150
rs11943905
rs10060382
rs1044352
rs2833098
rs4665630
rs4794333
rs68082256
rs11890028
rs887696
All − MR Egger
All − Inverse variance weighted
−0.015
−0.010
−0.005
0.000
0.005
0.010
MR effect size for'generalized epilepsy, all documented cases || id:ieu−b−9' on 'Fractured heel || id:ukb−b−18389'

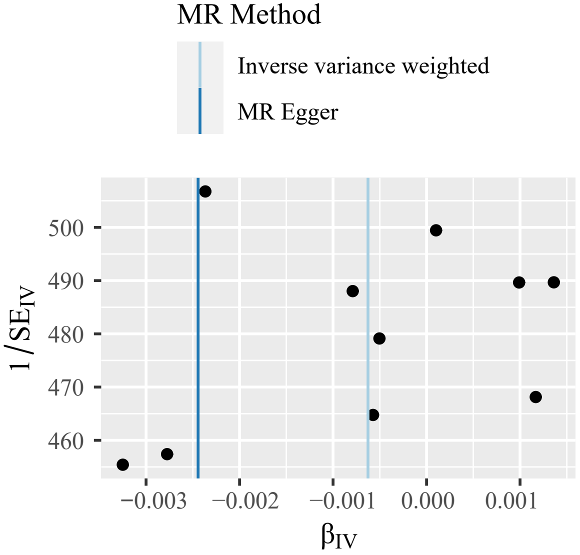

MR Method
Inverse variance weighted
MR Egger
500
490
480
470
460
$1/SE_{IV}$
−0.003
−0.002
−0.001
0.000
0.001
β_{IV}
