# Supplementary material for: Mendelian randomization analysis does not reveal a causal influence of mental diseases on osteoporosis
Source: Front Endocrinol (Lausanne). 2023 Apr 20;14:1125427. doi: 10.3389/fendo.2023.1125427 (PMC10157183; doi:10.3389/fendo.2023.1125427)
Supplement: Supplementary Material 1 — Instrumental variables SNPs. [file DataSheet_1.zip › Supplementary Material/Supplementary Material 9.pdf]

Code:

```
library(TwoSampleMR)

aaa <- extract_instruments(outcomes=' ieu-a-22', clump=TRUE, r2=0.001,kb=10000,access_token= NULL )

abc <- extract_outcome_data(
  snps=aaa$SNP,
  outcomes='ieu-b-4965',
  proxies = FALSE,
  maf_threshold = 0.01,
  access_token = NULL)


Mydata <- harmonise_data(
        exposure_dat=aaa,
        outcome_dat=abc,
        action= 2
```

```
)

mr_presso(BetaOutcome ="beta.outcome", BetaExposure = "beta.exposure", SdOutcome ="se.outcome", SdExposure = "se.exposure", OUTLIERtest = TRUE,DISTORTIONtest = TRUE, data =Mydata, NbDistribution = 1000, SignifThreshold = 0.05)

mr(Mydata, method_list=c("mr_ivw", "mr_ivw_fe", "mr_two_sample_ml", "mr_egger_regression", "mr_weighted_median", "mr_penalised_weighted_median", "mr_simple_mode", "mr_weighted_mode"))

mr_heterogeneity(Mydata, method_list=c("mr_egger_regression", "mr_ivw"))

pleio <- mr_pleiotropy_test(Mydata)
pleio
```

```
single <- mr_leaveoneout(Mydata)
  mr_leaveoneout_plot(single)

mr_scatter_plot(res,Mydata)

res_single <- mr_singlesnp(Mydata)
mr_forest_plot(res_single)

mr_funnel_plot(res_single)

generate_odds_ratios(res)
```
